# Limiting Absorption Principles and Linear Inviscid Damping in the Euler–Boussinesq System in the Periodic Channel

**DOI:** 10.1007/s00220-024-05224-y

**Published:** 2025-02-17

**Authors:** Michele Coti Zelati, Marc Nualart

**Affiliations:** 1 Department of Mathematics, Imperial College London, London, SW7 2AZ UK; 2 Consejo Superior de Investigaciones Científicas, Instituto de Ciencias Matemáticas, 28049 Madrid, Spain

## Abstract

We consider the long-time behavior of solutions to the two dimensional non-homogeneous Euler equations under the Boussinesq approximation posed on a periodic channel. We study the linearized system near a linearly stratified Couette flow and prove inviscid damping of the perturbed density and velocity field for any positive Richardson number, with optimal rates. Our methods are based on time-decay properties of oscillatory integrals obtained using a limiting absorption principle, and require a careful understanding of the asymptotic expansion of the generalized eigenfunction near the critical layer. As a by-product of our analysis, we provide a precise description of the spectrum of the linearized operator, which, for sufficiently large Richardson number, consists of an essential spectrum (as expected according to classical hydrodynamic problems) as well as discrete neutral eigenvalues (giving rise to oscillatory modes) accumulating towards the endpoints of the essential spectrum.

## Introduction

Under the Boussinesq approximation, the motion of an incompressible, non-homogeneous, inviscid fluid is described by the Euler equations

1.1 $$\begin{aligned} \begin{aligned} (\partial _t+{\tilde{\varvec{v}}}\cdot \nabla ){\tilde{{\omega }}}&= -\mathfrak {g}\partial _x{\tilde{\rho }},\\ (\partial _t+{\tilde{\varvec{v}}}\cdot \nabla ){\tilde{\rho }}&=0, \end{aligned} \end{aligned}$$

where ${\tilde{\varvec{v}}}=\nabla ^\perp \Delta ^{-1}{\tilde{{\omega }}}$ denotes the velocity field of the fluid with vorticity ${\tilde{{\omega }}}=\nabla ^\perp \cdot {\tilde{\varvec{v}}}$ and density ${\tilde{\rho }}$, and $\mathfrak {g}$ is the gravity constant.

In the periodic channel ${{\mathbb {T}}}\times [0,1]$, we are interested in the linear asymptotic stability of the special equilibrium solution

1.2 $$\begin{aligned} \bar{\varvec{v}}=(y,0), \qquad \bar{\rho }(y)=1-\vartheta y, \qquad \partial _y p=-\mathfrak {g}\bar{\rho }(y), \end{aligned}$$

which describes a Couette flow that is linearly stratified by a density with slope $\vartheta >0$. We introduce the perturbed velocity $\tilde{\varvec{v}}=\bar{\varvec{v}}+\varvec{v}$ and density profile $\tilde{\rho }=\bar{\rho }+\vartheta \rho$, and define the corresponding vorticity perturbation ${\omega }=\nabla ^\perp \cdot \varvec{v}$. After neglecting the nonlinear terms, the linearized Euler–Boussinesq system (1.1) near (1.2) can be written as

1.3 $$\begin{aligned} {\left\{ \begin{array}{ll} \partial _t{\omega }+ y\partial _x{\omega }=-\beta ^2\partial _x\rho \\ \partial _t\rho + y\partial _x\rho =\partial _x\psi ,\\ {\Delta }\psi ={\omega }, \end{array}\right. } \end{aligned}$$

with $\psi$ being the streamfunction and $\beta =\sqrt{\vartheta \mathfrak {g}} >0$. The understanding of the long-time dynamics of solutions to (1.3) is very much related to the spectral properties of the associated linear operator

1.4 $$\begin{aligned} {\mathcal {L}}=\begin{pmatrix} y\partial _x &  \beta ^2\partial _x \\ -{\Delta }^{-1}\partial _x &  y\partial _x \end{pmatrix}. \end{aligned}$$

In the setting of the periodic channel, ${\mathcal {L}}$ can have quite interesting features: it has both continuous and point spectrum, with a sequence of eigenvalues accumulating to the endpoint of the spectrum. As a consequence, any asymptotic stability result requires well-prepared initial data, whose projection onto the point spectrum vanishes.

We summarize the main result of this article in the following theorem. There a few key assumptions on the initial data that we informally state in the theorem and comment on right after.

### Theorem 1

Let $\beta >0$ and assume that the initial data $({\omega }^0,\rho ^0)$ vanish on the physical boundaries, has trivial projection onto the subspace generated by the eigenfunctions of ${\mathcal {L}}$, satisfy an orthogonality condition at the endpoint of the essential spectrum and

1.5 $$\begin{aligned} \int _{{\mathbb {T}}}{\omega }^0(x,y)\textrm{d}x = \int _{{\mathbb {T}}}\rho ^0(x,y)\textrm{d}x =0. \end{aligned}$$

Let $\varvec{v}=(v^x,v^y)=\nabla ^\perp \psi =(-\partial _y\psi ,\partial _x\psi )$ be the corresponding velocity field. We have the following estimates.

- If $\beta ^2\ne 1/4$, let $\mu =\textrm{Re}\sqrt{1/4-\beta ^2}$ and $\nu =\textrm{Im}\sqrt{1/4-\beta ^2}$. Then,
  1.6 $$\begin{aligned} \Vert v^x(t) \Vert _{L^2}&\lesssim \frac{1}{t^{\frac{1}{2}-\mu }}\left( \Vert \rho ^0 \Vert _{L^2_xH^3_y} + \Vert {\omega }^0 \Vert _{L^2_xH^3_y}\right) , \end{aligned}$$
  1.7 $$\begin{aligned} \Vert v^y(t) \Vert _{L^2}&\lesssim \frac{1}{t^{\frac{3}{2}-\mu }}\left( \Vert \rho ^0 \Vert _{L^2_xH^4_y} + \Vert {\omega }^0 \Vert _{L^2_xH^4_y}\right) , \end{aligned}$$
  1.8 $$\begin{aligned} \Vert \rho (t) \Vert _{L^2}&\lesssim \frac{1}{t^{\frac{1}{2}-\mu }}\left( \Vert \rho ^0 \Vert _{H^1_xH^3_y} + \Vert {\omega }^0 \Vert _{H^1_xH^3_y}\right) , \end{aligned}$$
  for all $$t\ge 1$$.
- If $$\beta ^2=1/4$$, then
  1.9 $$\begin{aligned} \Vert v^x(t) \Vert _{L^2}&\lesssim \frac{1+\log (t)}{t^\frac{1}{2}}\left( \Vert \rho ^0 \Vert _{L^2_xH^3_y} + \Vert {\omega }^0 \Vert _{L^2_xH^3_y}\right) , \end{aligned}$$
  1.10 $$\begin{aligned} \Vert v^y(t) \Vert _{L^2}&\lesssim \frac{1+\log (t)}{t^\frac{3}{2}}\left( \Vert \rho ^0 \Vert _{L^2_xH^4_y} + \Vert {\omega }^0 \Vert _{L^2_xH^4_y}\right) , \end{aligned}$$
  1.11 $$\begin{aligned} \Vert \rho (t) \Vert _{L^2}&\lesssim \frac{1+\log (t)}{t^\frac{1}{2}}\left( \Vert \rho ^0 \Vert _{H^1_xH^3_y} + \Vert {\omega }^0 \Vert _{H^1_xH^3_y}\right) , \end{aligned}$$
  for all $$t\ge 1$$.

### Remark 1.1

*(Assumptions on data)*. The assumptions on the initial data are completely natural. The vanishing at the boundary points $y\in \{0,1\}$ is a typical requirement [20, 23], while (1.5) is inessential, as the *x*-average is a constant of motion for (1.3). The null projection of the data to the eigenfunctions of $\mathcal {L}$ is needed to avoid oscillatory, non-decaying modes (which are present for $\beta ^2>1/4$, see Sect. 2.5). Lastly, the precise meaning of the spectral assumption at the endpoints of the essential spectrum $\sigma _{ess}(\mathcal {L})=[0,1]$ is in condition (H) in Sect. 2.8 below. It requires orthogonality to certain generalized eigenfunctions that appear at $\partial \sigma _{ess}(\mathcal {L})=\{0,1\}$.

For initial data with no assumptions on its spectral projection on the discrete eigenvalues, the solution to the linearized dynamics can be decomposed into countably many non-decaying oscillatory waves associated to the discrete eigenvalues, and an additional component that experiences inviscid damping with time-decay rates given by (1.6)–(1.11).

The inviscid damping estimates (1.6)–(1.11) encode the asymptotic stability of (1.3) and precisely describe the long-time dynamics. The decay is due to a combination of *mixing* (due to the background Couette flow) and *stratification* (due to the background density). The former has been extensively studied in the homogeneous Euler equations both at the linear level [2, 8, 13, 22, 23, 28–30, 35–38] and at the nonlinear level [3, 19–21, 24].

In the presence of stratification, the spectral stability of the Euler–Boussinesq system has been address in the classical work of Miles [25] and Howard [17]. See [32, Section 3.2.3] for a survey on the literature regarding the spectral problem. The first work in the direction of asymptotic stability dates back to Hartman [16] in 1975, in which (1.3) on ${{\mathbb {T}}}\times {{\mathbb {R}}}$ was solved explicitly on the Fourier side using hypergeometric functions. Moreover, it was predicted the vorticity should be unstable in $L^2$, with a growth proportional to $\sqrt{t}$. This approach was used in [33] to prove decay rates analogous to those in Theorem 1 in ${{\mathbb {T}}}\times {{\mathbb {R}}}$. In this spatial setting, a different approach based on an energy method in Fourier space was used in [4] to prove both inviscid damping and instability in the spectrally stable regime $\beta ^2>1/4$, confirming the predictions of [16]. The analysis has been extended in the full nonlinear setting in [1]. A third proof of linear inviscid damping on ${{\mathbb {T}}}\times {{\mathbb {R}}}$ can be found in our companion article [7], in which the methods developed here can be used to provide explicit solutions in physical variables to (1.3).

Our article constitutes the first result of (linear) asymptotic stability of a stably stratified shear flow for the Euler–Boussinesq equations in the periodic channel, as well as the first rigorous characterization of the spectrum of the linearized operator (1.4) and in particular the existence of discrete neutral eigenvalues for $\beta ^2>1/4$. From a technical standpoint, the main difficulty lies in the stratification of the background density ${\bar{\rho }}$. This manifests itself in the equation that rules the underlying spectral problem (the Taylor–Goldstein equation, see (TG) below), which becomes more singular than the usual Rayleigh equation for inviscid homogeneous fluids.

This work also connects with the global well-posedness for the Euler–Boussinesq equations and, by extension, to the axisymmetric 3d Euler equations. Certain solutions to the Euler–Boussinesq and 3d Euler equations are known to blow up in finite time, see the ground-breaking work of Elgindi [9], and related works [5, 10, 11]. On the other hand, there are examples where inviscid damping plays a key role in proving global well-posedness for the 3d Euler equations and for the inhomogeneous 2d Euler equations, see [6, 14, 34], respectively. We remark here that the absence of gravity in [34] results in the linearised dynamics being governed by a modified version of the Rayleigh equation, the solutions of which retain sufficient regularity so that the inviscid damping time-decay rates coincide with those for the homogeneous Euler equations. In the case of Euler–Boussinesq near stratified shear flows, a long-time existence result relying on inviscid damping estimates can be found in [1].

## Main Ideas and Outline of the Article

In this section, we give a brief account of the strategy of proof of Theorem 1, recording the main steps that will be then expanded in the subsequent sections, and providing a quick reasoning behind the assumptions of Theorem 1 on the initial data. We focus on the case $\beta ^2\ne 1/4$ for the sake of clarity. When $\beta ^2=1/4$, the strategy is the same, but the statements of the main results typically differ by a logarithmic correction, and we prefer to postpone them in the relevant Sect. 5. We also set some of the notation and assumptions that will be used throughout the manuscript.

### Fourier decomposition and spectral representation

The setting of the periodic channel ${{\mathbb {T}}}\times [0,1]$ considered in this article poses new challenges as it forbids the use of Fourier methods in the vertical direction *y*. However, we can decouple (1.3) in Fourier modes in $x\in {{\mathbb {T}}}$, writing

$$\begin{aligned} {\omega }=\sum _{m\in {{\mathbb {Z}}}}{\omega }_m(t,y)\textrm{e}^{imx}, \qquad \rho =\sum _{m\in {{\mathbb {Z}}}}\rho _m(t,y)\textrm{e}^{imx}, \qquad \psi =\sum _{m\in {{\mathbb {Z}}}}\psi _m(t,y)\textrm{e}^{imx}, \end{aligned}$$

so that

$$\begin{aligned} (\partial _t+imy){\omega }_m=-im\beta ^2\rho _m, \qquad (\partial _t+imy)\rho _m=im\psi _m, \end{aligned}$$

for each $m\in {{\mathbb {Z}}}$, with

$$\begin{aligned} {\left\{ \begin{array}{ll} {\Delta }_m\psi _m ={\omega }_m, \\ \psi _m|_{y=0,1}=0, \end{array}\right. } \qquad {\Delta }_m:= \partial _y^2-m^2. \end{aligned}$$

The modes corresponding to the *x*-average, namely when $m=0$, are clearly conserved and therefore we will not consider them further (cf. (1.5)). Moreover, since ${\omega }$ and $\rho$ are real-valued, we necessarily have that $\overline{{\omega }_{-m}}={\omega }_m$ and $\overline{\rho _{-m}}=\rho _m$. Without loss of generality, we take $m\ge 1$.

For our purposes, it is more convenient to write (1.3) in the compact stream-function formulation

$$\begin{aligned} \partial _t \begin{pmatrix} \psi _m \\ \rho _m\end{pmatrix}+imL_m\begin{pmatrix} \psi _m \\ \rho _m \end{pmatrix}=0, \end{aligned}$$

and directly obtain its solution as

$$\begin{aligned} \begin{pmatrix} \psi _m \\ \rho _m\end{pmatrix}=\textrm{e}^{-imL_mt}\begin{pmatrix} \psi _m^0 \\ \rho _m^0 \end{pmatrix} \end{aligned}$$

where $L_m$ is the linear operator defined by

2.1 $$\begin{aligned} L_m =\begin{pmatrix} {\Delta }_m^{-1}(y{\Delta }_m) &  \beta ^2{\Delta }_m^{-1} \\ -1 &  y \end{pmatrix} \end{aligned}$$

Using Dunford’s formula [12, 27], we have that

2.2 $$\begin{aligned} \begin{pmatrix} \psi _m(t,y) \\ \rho _m(t,y) \end{pmatrix} = \frac{1}{2\pi i} \int _{\partial {\Omega }}\textrm{e}^{-imct} (c-L_m)^{-1}\begin{pmatrix} \psi _m^0(y) \\ \rho _m^0(y) \end{pmatrix} \,\textrm{d}c, \end{aligned}$$

where here ${\Omega }$ is any domain containing the spectrum $\sigma (L_m)$. Under suitable conditions on the initial data (see Proposition 6.1 below), we can reduce the contour of integration to

2.3 $$\begin{aligned}  &   \begin{pmatrix} \psi _m(t,y) \\ \rho _m(t,y) \end{pmatrix} =\frac{1}{2\pi i }\lim _{\varepsilon \rightarrow 0}\int _0^1 \textrm{e}^{-imy_0t}\left[ (-y_0-i\varepsilon +L_m)^{-1}-(-y_0+i\varepsilon +L_m)^{-1}\right] \nonumber \\  &   \quad \begin{pmatrix} \psi _m^0 \\ \rho _m^0 \end{pmatrix}\, \textrm{d}y_0. \end{aligned}$$

In particular, the contour integral along the essential spectrum of $L_m$, $\sigma _{ess}(L_m)=[0,1]$ is the only non-trivial contribution from $\sigma (L_m)$ to the Dunford’s formula. For $\varepsilon >0$, we denote

2.4 $$\begin{aligned} \begin{pmatrix} \psi ^{\pm }_{m,\varepsilon }(y,y_0) \\ \rho ^\pm _{m,\varepsilon }(y,y_0) \end{pmatrix}:=\left( -y_0\pm i\varepsilon +L_m\right) ^{-1}\begin{pmatrix} \psi _m^0(y) \\ \rho _m^0(y) \end{pmatrix} \end{aligned}$$

and obtain the coupled system of equations

$$\begin{aligned} \begin{aligned} {\omega }_m^0(y)&=(y-y_0\pm i\varepsilon ){\Delta }_m\psi ^\pm _{m,\varepsilon }(y,y_0)+\beta ^2 \rho ^\pm _{m,\varepsilon }(y,y_0), \\ \rho _m^0(y)&=(y-y_0\pm i\varepsilon )\rho ^\pm _{m,\varepsilon }(y,y_0) -\psi ^\pm _{m,\varepsilon }(y,y_0). \end{aligned} \end{aligned}$$

We first solve

2.5 $$\begin{aligned} \rho ^\pm _{m,\varepsilon }(y,y_0)=\frac{1}{y-y_0\pm i\varepsilon }\left( \rho _m^0(y)+\psi ^\pm _{m,\varepsilon }(y,y_0)\right) \end{aligned}$$

and from there we obtain the following inhomogeneous *Taylor–Goldstein equation* for $\psi ^\pm _{m,\varepsilon }$,

TG $$\begin{aligned} {\Delta }_m\psi ^{\pm }_{m,\varepsilon }+\beta ^2\frac{\psi ^\pm _{m,\varepsilon }}{(y-y_0\pm i\varepsilon )^2}=\frac{{\omega }_m^0}{y-y_0\pm i\varepsilon }-\beta ^2\frac{\rho _m^0}{(y-y_0\pm i\varepsilon )^2}, \end{aligned}$$

along with homogeneous Dirichlet boundary conditions at $y=0,1$.

### Notation and conventions

Throughout the manuscript, we assume $\beta >0$ and $m\ge 1$. We say that $A\lesssim B$ when there exists $C>0$ such that $A\le CB$. Also, for $j\ge 0$ we define

$$\begin{aligned} Q_{j,m}=\Vert \rho _m^0 \Vert _{H^{j+2}_y} + \Vert {\omega }_m^0 \Vert _{H^{j+2}_y}, \end{aligned}$$

to quantify the regularity requirements on the initial data.

### Green’s function for the Taylor–Goldstein equation

Solutions to (TG) are fundamental objects of study of this work. They can be constructed via the classical method of Green’s functions, by first solving the *homogeneous* Taylor–Goldstein equation

TGh $$\begin{aligned} \textsc {TG}_{m,\varepsilon }^\pm \phi =0, \qquad \textsc {TG}_{m,\varepsilon }^\pm := {\Delta }_m +\frac{\beta ^2}{(y-y_0\pm i\varepsilon )^2}, \end{aligned}$$

for $y\in (0,1)$. We refer to $\textsc {TG}_{m,\varepsilon }^\pm$ as to the *Taylor–Goldstein operator*. As in the statement of Theorem 1, we define throughout the article the numbers

2.6 $$\begin{aligned} \mu =\textrm{Re}\left( \sqrt{1/4-\beta ^2}\right) , \qquad \nu =\textrm{Im}\left( \sqrt{1/4-\beta ^2}\right) , \end{aligned}$$

and we denote by ${\mathcal {G}}^\pm _{m,\varepsilon }(y,y_0,z)$ the Green’s function of the Taylor–Goldstein equation, which satisfies

2.7 $$\begin{aligned} \textsc {TG}_{m,\varepsilon }^\pm {\mathcal {G}}^\pm _{m,\varepsilon }(y,y_0,z)=\delta (y-z). \end{aligned}$$

While ${\mathcal {G}}^\pm _{m,\varepsilon }(y,y_0,z)$ has an explicit expression, reported in Proposition 3.1, we record its important properties as the key result.

#### Theorem 2

Let $\beta ^2\ne 1/4$. There exists $\varepsilon _0>0$ such that for all $\varepsilon \in (0, \varepsilon _0)$ and for all $y,y_0\in [0,1]$ such that $m|y-y_0|\le 3\beta$, we have

$$\begin{aligned} |y-y_0+i\varepsilon |^{-\frac{1}{2}+\mu } \Vert {\mathcal {G}}^\pm _{m,\varepsilon }(y,y_0,\cdot )\Vert _{L^2_z}+ |y-y_0+i\varepsilon |^{\frac{1}{2}+\mu } \Vert \partial _y{\mathcal {G}}^\pm _{m,\varepsilon }(y,y_0,\cdot )\Vert _{L^2_z}\lesssim \frac{1}{m^{1+\mu }}. \end{aligned}$$

The theorem provides sharp bounds on the Green’s function near the *critical layer* $y=y_0$, where (TGh) is singular and (TG) has a regular singular point. The scale of the problem is crucially determined by $\beta$ and *m*.

The proof of Theorem 2 is carried out in Sect. 4, while the analogous result for $\beta ^2=1/4$ is stated in Theorem 5 and proven in Sect. 5. They are based on the asymptotic properties of Whittaker functions [31], whose main properties can be found in Appendix A.

### Regularization of the generalized stream-functions

The source term of (TG) is, a priori, too singular for $\psi _{m,\varepsilon }^\pm$ to be obtained as an application of the Green’s function on (TG). However, the singularity of the source term is no worse than $\frac{\beta ^2}{(y-y_0\pm i\varepsilon )^2}$, which is precisely the potential of the Taylor–Goldstein operator (TGh). Then, (TG) may be written as

$$\begin{aligned} \text {TG}_{m,\varepsilon }^\pm \psi _m^\pm =\text {TG}_{m,\varepsilon }^\pm \left( \frac{1}{\beta ^2}(y-y_0\pm i\varepsilon ){\omega }_m^0 -\rho _m^0\right) + {\Delta }_m\big (\rho _m^0(y)-\frac{1}{\beta ^2}(y-y_0\pm i\varepsilon ){\omega }_m^0(y)\big ). \end{aligned}$$

Hence, for $z,y_0\in [0,1]$ and $0\le \varepsilon \le 1$, define

2.8 $$\begin{aligned} F_{m,\varepsilon }^\pm (z,y_0):={\Delta }_m\rho _m^0(z)-\frac{1}{\beta ^2}{\Delta }_m\big ((z-y_0\pm i\varepsilon ){\omega }_m^0(z)\big ) \end{aligned}$$

and note that, since the pair of initial data vanish on the physical boundaries $y=0$ and $y=1$, the solution $\psi ^\pm _{m,\varepsilon }(y,y_0)$ to (TG) is given by

2.9 $$\begin{aligned} \begin{aligned} \psi ^\pm _{m,\varepsilon }(y,y_0)&= \frac{1}{\beta ^2}(y-y_0\pm i\varepsilon ){\omega }_m^0(y) -\rho _m^0(y) + \varphi _{m,\varepsilon }^\pm (y,y_0), \end{aligned} \end{aligned}$$

while

2.10 $$\begin{aligned} \rho _{m,\varepsilon }^\pm (y,y_0)=\frac{1}{\beta ^2}{\omega }_m^0(y) +\frac{1}{y-y_0\pm i\varepsilon } \varphi _{m,\varepsilon }^\pm (y,y_0). \end{aligned}$$

Here, $$\varphi _{m,\varepsilon }^\pm $$ solves

2.11 $$\begin{aligned} \textsc {TG}_{m,\varepsilon }^\pm \varphi _{m,\varepsilon }^\pm = F_{m,\varepsilon }^\pm \end{aligned}$$

and is given by

2.12 $$\begin{aligned} \varphi _{m,\varepsilon }^\pm (y,y_0)=\int _0^1 {\mathcal {G}}^\pm _{m,\varepsilon }(y,y_0,z) F_{m,\varepsilon }^\pm (z,y_0)\textrm{d}z. \end{aligned}$$

The main reason to write $\psi _{m,\varepsilon }^\pm$ and $\rho _{m,\varepsilon }^\pm$ using (2.9) and (2.10) is that now $F_{m,\varepsilon }^\pm \in L^2_z$ and we can use the bounds on the Green’s function ${\mathcal {G}}_{m,\varepsilon }^\pm$ from Theorem 2 in (2.12) to estimate $\varphi _{m,\varepsilon }^\pm$, and thus $\psi _{m,\varepsilon }^\pm$ and $\rho _{m,\varepsilon }^\pm$, near the critical layer. The introduction of $F_{m,\varepsilon }^\pm$ constitutes a first example of the underlying *motif* of inviscid damping, namely that *decay costs regularity*.

### Spectral picture

The main assumption of Theorem 1 consists in requiring that the initial data are orthogonal to the subspace generated by the eigenfunctions of $L_m$. Generically speaking, (embedded) eigenvalues may constitute an obstruction to damping phenomena, as they can give rise to oscillatory modes or even growing (hence unstable) modes. The spectral picture here is quite intriguing and drastically different compared to the case of the periodic strip. The main result on the spectrum of $L_m$ is below.

#### Theorem 3

Let $\beta >0$. Then the essential spectrum of $L_m$ is $\sigma _{ess}(L_m)=[0,1]$. Moreover,

- any eigenvalue $c\in {{\mathbb {C}}}$ such that $\left| \textrm{Re}(c)-1/2\right| \ge 1/2$, must have $\textrm{Im}(c)= 0$;
- for $$\beta ^2>1/4$$,
  - there are no eigenvalues $c\in {{\mathbb {C}}}$ such that $\textrm{Im}(c)\ne 0$ and $\textrm{Re}(c)\in (0,1)$.
  - there are no real eigenvalues $c\in {{\mathbb {R}}}$ such that $c<-\beta /m$ or $c>1+\beta /m$.
  - there is a countably infinite number of discrete eigenvalues $c\in {{\mathbb {C}}}$, with $\textrm{Im}(c)=0$ and $\textrm{Re}(c)\in \left( -\beta /m,0\right) \cup \left( 1,1+\beta /m\right)$. Moreover, they accumulate exponentially fast towards 0 and 1.
- for $$\beta ^2\le 1/4$$,
  - there is no eigenvalue $c\in {{\mathbb {C}}}$ such that $\textrm{Re}(c)\le 0$ or $\textrm{Re}(c)\ge 1$.
  - there is no eigenvalue $c\in {{\mathbb {C}}}$ such that $\left| \textrm{Im}(c)\right| \ge \beta /m$ or $\left| \textrm{Im}(c)\right| \le \varepsilon _0$.

**Fig. 1:**
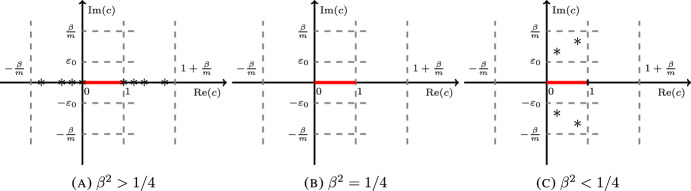
The essential spectrum $\sigma _{ess}(L_m)=[0,1]$ is in red. Eigenvalues are denoted by $*$. Theorem 3 shows their existence for $\beta ^2> 1/4$, while when $\beta ^2<1/4$ we can only discern that they do not exist close to the essential spectrum

The three cases outlined above are depicted in Fig. 1. Unstable eigenmodes can be ruled out by the classical Miles-Howard stability criterion [17, 25] when $\beta ^2\ge 1/4$, so that any eigenvalue $c\in {{\mathbb {C}}}$ of $L_m$ must have $\textrm{Im}(c)=0$. However, spectral stability is typically not sufficient to deduce asymptotic stability. This is particularly clear when $\beta ^2>1/4$, for which infinitely many eigenvalues exist, corresponding to neutral (oscillatory) modes. This is a specific feature of the problem in the *periodic channel*. The same problem on the periodic strip does not have any of these modes, as the essential spectrum is the whole real line, and hence eigenvalues are “pushed away to infinity”. In the periodic channel, each of these discrete eigenvalues are found to be zeroes of the Wronskian of the Green’s function and this is precisely how we characterize them in Proposition 6.3.

When $\beta ^2<1/4$, we are able to rule out the existence of eigenvalues in the proximity of the essential spectrum, which is a consequence of suitable lower bounds on the Wronskian. Nonetheless, isolated unstable eigenvalues in an intermediate region may exist in this case, although their presence does not affect the conclusion of Theorem 1 if the data are orthogonal to them. The proof of their existence is an interesting open question.

The proof of Theorem 3 is postponed to Sect. 6. It requires an extensive analysis of the resolvent operator $(c-L_m)^{-1}$ and of spectral integrals of the form (2.2), where the domain of integration containing the essential spectrum is carefully designed.

### Solutions to the inhomogeneous Taylor–Goldstein equation

Once the Green’s function is established and (TG) is regularized due to the introduction of $F_{m,\varepsilon }^\pm$ and $\varphi _{m,\varepsilon }^\pm$, most of the analysis on $\psi _{m,\varepsilon }^\pm$ will follow from the properties of generic solutions $\Phi _{m,\varepsilon }^\pm$ to the general inhomogeneous Taylor–Goldstein equation

TGf $$\begin{aligned} \textsc {TG}_{m,\varepsilon }^\pm \Phi _{m,\varepsilon }^\pm = f, \end{aligned}$$

for some $f\in L^2$ and with boundary conditions $\Phi _{m,\varepsilon }^\pm (0,y_0)=\Phi _{m,\varepsilon }^\pm (1,y_0)=0$. To formally quantify the distance to the critical layer, for $y_0\in [0,1]$ and $n\ge 1$ we introduce the nested sets

$$\begin{aligned} J_n=\lbrace y\in [0,1]: m|y-y_0|\le n\beta \rbrace \end{aligned}$$

and $J_n^c=[0,1]\setminus J_n$. A direct consequence of Theorem 2 are the asymptotic expansions of $\Phi _{m,\varepsilon }^\pm$ near the critical layer. That is, for all $y\in J_3$ we have

2.13 $$\begin{aligned} |y-y_0\pm i\varepsilon |^{-\frac{1}{2}+\mu } |\Phi _{m,\varepsilon }^\pm (y,y_0)|+ |y-y_0\pm i\varepsilon |^{\frac{1}{2}+\mu } |\partial _y \Phi _{m,\varepsilon }^\pm (y,y_0)|\lesssim \frac{1}{m^{1+\mu }}\Vert f \Vert _{L^2_y}. \nonumber \\ \end{aligned}$$

Using the entanglement inequality

2.14 $$\begin{aligned} \Vert \partial _y \Phi _{m,\varepsilon }^\pm \Vert _{L^2_y(J_3^c)}^2 + m^2 \Vert \Phi _{m,\varepsilon }^\pm \Vert _{L^2_y(J_3^c)}^2 \lesssim m^2 \Vert \Phi _{m,\varepsilon }^\pm \Vert _{L^2_y(J_2^c\cap J_3)}^2 + \frac{1}{m^2}\Vert f \Vert _{L^2_y(J_2^c)}^2, \qquad \end{aligned}$$

which is inspired from [18] and proved in Lemma 7.1, the localised asymptotic expansions (2.13) provide integral estimates on $\Phi _{m,\varepsilon }^\pm$ away from the critical layer,

2.15 $$\begin{aligned} \Vert \partial _y \Phi _{m,\varepsilon }^\pm (y,y_0)\Vert _{L^2_y(J_3^c)} + m\Vert \Phi _{m,\varepsilon }^\pm (y,y_0)\Vert _{L^2_y(J_3^c)}\lesssim \frac{1}{m}\Vert f \Vert _{L^2_y}. \end{aligned}$$

The precise statements and proofs of (2.13) and (2.15), as well as the corresponding versions for $\beta ^2=1/4$, can be found in Proposition 7.2 in Sect. 7.

### Inviscid damping estimates through the limiting absorption principle

The last step in the proof of Theorem 1 is a stationary phase argument to deduce decay of $\psi _m$ and $\rho _m$ in (2.3). As customary, it involves an integration by parts in the spectral variable $y_0$ to gain time-decay from the oscillatory phase. The amount of decay that can be obtained is linked to the regularity of the generalized streamfunctions $\psi ^{\pm }_{m,\varepsilon }$ in (2.4), and even more crucially to their asymptotic expansion at the critical layer (matching that of the Green’s function in Theorem 2, as can be seen from (2.9) and (2.12)). Moreover, the integration leads to boundary terms at the endpoint of the spectrum that need to be treated *ad hoc*.

To obtain the asymptotic expansions of $\psi _{m,\varepsilon }^\pm$ near the critical layer, in Proposition 3.5 we observe that $\partial _y + \partial _{y_0}$ commutes with the Taylor–Goldstein operator (TGh) and we deduce formulas for $\partial _{y_0}\psi _{m,\varepsilon }^\pm$, and several other derivatives with respect to both *y* and $y_0$. These formulas involve solutions $\Phi _{m,\varepsilon }^\pm$ to (TGf) for source terms *f* given by derivatives of $F_{m,\varepsilon }^\pm$. As is clear from (2.13), the asymptotic expansions of $\Phi _{m,\varepsilon }^\pm$, and in turn of $\partial _{y_0}\psi _{m,\varepsilon }^\pm$ and related derivatives, are conditional to the $L^2$ boundedness of derivatives of $F_{m,\varepsilon }^\pm$, constituting a further example of the fact that decay costs regularity.

Some formulas from Proposition 3.5 involve as well terms related to $\partial _y\varphi _{m,\varepsilon }^\pm (z,y_0)$, and higher derivatives, evaluated at the physical boundaries $z=0$ and $z=1$. In general, these boundary terms arise when the Taylor–Goldstein operator (TGh) acting on the $\partial _y$ derivative of solutions to (TGf) is inverted, and usually they do not vanish. See Proposition 3.5 for more details. Near the critical layer, these boundary terms are studied in Sect. 8 and some require (H) to be sufficiently regular, see Proposition 8.3 for more details.

Once the asymptotic expansions for $\psi _{m,\varepsilon }^\pm$ near the critical layer are established via Proposition 3.5 and Proposition 7.2, these are used through the entanglement inequality (2.14) to derive the regularity estimates of $\psi _{m,\varepsilon }^\pm$ away from the critical layer. Additionally, asymptotic expansions and regularity estimates for $\rho _{m,\varepsilon }^\pm$ are deduced accordingly thanks to (2.10). The precise statements and proofs are found in Sect. 9. Both the asymptotic expansions and the regularity estimates are uniform in $\varepsilon$ sufficiently small, so that the limiting functions in (2.3) retain the same properties.

### Limiting absorption principle for spectral boundary terms

The stationary phase argument employed in the proof of Theorem 1 requires an integration by parts in the spectral variable $y_0$ in (2.3) regarding $\psi _m$ that involves spectral boundary terms evaluated at $y_0=0$ and $y_0=1$. These boundary terms are

2.16 $$\begin{aligned} \begin{aligned} -\frac{1}{2\pi i}\frac{1}{imt}\lim _{\varepsilon \rightarrow 0}\Big [ \textrm{e}^{-imy_0t}\left( \psi _{m,\varepsilon }^-(y,y_0)-\psi _{m,\varepsilon }^+(y,y_0)\right) \Big ]_{y_0=0}^{y_0=1}. \end{aligned} \end{aligned}$$

For $y_0=0$, from (2.9) and (2.12) we note that

2.17 $$\begin{aligned} \psi _{m,\varepsilon }^-(y,0)-\psi _{m,\varepsilon }^+(y,0)= &   -\frac{2i\varepsilon }{\beta ^2}{\omega }_m^0 +\int _0^1 \left( {\mathcal {G}}_{m,\varepsilon }^-(y,0,z)-{\mathcal {G}}_{m,\varepsilon }^+(y,0,z)\right) F_{m}(z,0)\textrm{d}z \nonumber \\  &   +\frac{i\varepsilon }{\beta ^2}\int _0^1 \left( {\mathcal {G}}_{m,\varepsilon }^-(y,0,z)+{\mathcal {G}}_{m,\varepsilon }^+(y,0,z)\right) {\Delta }_m{\omega }_m^0\textrm{d}z, \end{aligned}$$

where $F_m(z,0)=F_{m,0}^\pm (z,0)$. Moreover, for $\beta ^2>\frac{1}{4}$, from Lemma 6.15, there exists $\varepsilon _0>0$ and $C_\varepsilon \ge C_0>0$ such that

2.18 $$\begin{aligned} \left| {\mathcal {G}}_{m,\varepsilon }^-(y,0,z)-{\mathcal {G}}_{m,\varepsilon }^+(y,0,z)-C_\varepsilon \phi _{u,m}(y)\phi _{u,m}(z)\right| \lesssim \varepsilon ^\frac{1}{2}, \end{aligned}$$

for all $\varepsilon \le \varepsilon _0$ and uniformly in $y,z\in [0,1]$. Here, $\phi _{u,m}$, given by (3.7), denotes the generalized eigenfunction associated to the generalized eigenvalue $y_0=0$. Analogous expressions to (2.17) and (2.18) can be deduced for the boundary term associated to $y_0=1$, now involving $\phi _{l,m}$, the generalized eigenfunction associated to the generalized eigenvalue $y_0=1$ and given by (3.8).

In view of (2.18), for (2.16) to vanish we require the initial data $({\omega }_m^0, \rho _m^0)$ to be such that

H $$\begin{aligned} \int _0^1 \phi _{u,m}(z)F_m(z,0)\textrm{d}z=\int _0^1 \phi _{l,m}(z)F_m(z,1)\textrm{d}z=0. \end{aligned}$$

This is the key orthogonality assumption at the endpoint of the essential spectrum, which was discussed in Remark 1.1. Then, we are able to show

#### Theorem 4

We have that

$$\begin{aligned} \lim _{\varepsilon \rightarrow 0}\left\| \psi _{m,\varepsilon }^-(\cdot ,y_0)-\psi _{m,\varepsilon }^+(\cdot ,y_0)\right\| _{L^2_y}=0, \qquad y_0\in \{ 0, 1\}. \end{aligned}$$

The proof of Theorem 4 is carried out in Sect. 6, where (2.18) is shown in Lemma 6.15 for $\beta ^2>1/4$. For the case $\beta ^2\le 1/4$, the difference of Green’s functions at $y_0=0$ and $y_0=1$ vanish as $\varepsilon \rightarrow 0$ and no orthogonality conditions are needed, see Lemma 6.21 and Lemma 6.26 for more details.

## Explicit Solutions to the Taylor–Goldstein Equation

The first step towards the proof of Theorem 1 is to derive the expression of the Green’s function associated to (TG). The building block consists of the so-called Whittaker functions [31], a modified form of hypergeometric functions that solve equations of the form

3.1 $$\begin{aligned} \partial ^2_{\zeta } M_{\kappa ,\gamma }+\left( -{\frac{1}{4}}+{\frac{\kappa }{\zeta }}+{\frac{1/4- \gamma ^{2}}{\zeta ^{2}}}\right) M_{\kappa ,\gamma }=0, \qquad \zeta \in {{\mathbb {C}}}, \end{aligned}$$

for parameters $\kappa ,\gamma \in {{\mathbb {C}}}$. Their properties are reported in Appendix A. We believe that a precise understanding of the solutions to the Taylor–Goldstein equation for the Couette flow may shed some light on the analysis of the Taylor–Goldstein equation corresponding to other monotone shear flow configurations.

### The case $\beta ^2\ne 1/4$

We use Whittaker functions with $\gamma =\pm (\mu +i\nu )=\pm \sqrt{1/4-\beta ^2}$ and $b=0$, see (2.6), and denote by $M_\pm (\zeta ):=M_{0,\pm (\mu +i\nu )}(2m\zeta )$ the solution to the rescaled Whittaker equation

3.2 $$\begin{aligned} \partial ^2_{\zeta } M_\pm +\left( -{\frac{1}{4}} +{\frac{1/4- (1/4-\beta ^2)}{4m^2\zeta ^{2}}}\right) M_\pm =0, \qquad \zeta \in {{\mathbb {C}}}. \end{aligned}$$

The construction of the Green’s function is contained in the following result.

#### Proposition 3.1

Let $\varepsilon \in (0,1)$ and $\beta ^2\ne 1/4$. The Green’s function ${\mathcal {G}}_{m,\varepsilon }^\pm$ of $\textsc {TG}_{m,\varepsilon }^\pm$ is given by

3.3 $$\begin{aligned} {\mathcal {G}}_{m,\varepsilon }^\pm (y,y_0,z)=\frac{1}{{\mathcal {W}}_{m,\varepsilon }^\pm (y_0)} {\left\{ \begin{array}{ll} \phi _{u,m,\varepsilon }^\pm (y,y_0)\phi _{l,m,\varepsilon }^\pm (z,y_0), \quad & 0\le z\le y\le 1,\\ \phi _{u,m,\varepsilon }^\pm (z,y_0)\phi _{l,m,\varepsilon }^\pm (y,y_0), \quad & 0\le y\le z\le 1, \end{array}\right. } \end{aligned}$$

where $\phi _{u,m,\varepsilon }^\pm (\cdot ,y_0)$ and $\phi _{l,m,\varepsilon }^\pm (\cdot ,y_0)$ are two homogeneous solutions to TGh) such that $\phi _{u,m,\varepsilon }^\pm (1,y_0)=0$ and $\phi _{l,m,\varepsilon }^\pm (0,y_0)=0$, respectively, for all $y_0\in [0,1]$. They are explicitly given by

3.4 $$\begin{aligned} \phi _{u,m,\varepsilon }^\pm (y,y_0):=M_+(1-y_0\pm i \varepsilon )M_-(y-y_0\pm i\varepsilon )-M_-(1-y_0\pm i\varepsilon )M_+(y-y_0\pm i\varepsilon ) \nonumber \\ \end{aligned}$$

and

3.5 $$\begin{aligned} \phi _{l,m,\varepsilon }^\pm (y,y_0):=M_+(-y_0\pm i \varepsilon )M_-(y-y_0\pm i\varepsilon )-M_-(-y_0\pm i\varepsilon )M_+(y-y_0\pm i\varepsilon ), \nonumber \\ \end{aligned}$$

with Wronskian

3.6 $$\begin{aligned} {\mathcal {W}}_{m,\varepsilon }^\pm (y_0)&{:=}&4 (\mu +i\nu ) m\Big (M_+(-y_0\pm i\varepsilon )M_-(1-y_0\pm i\varepsilon )\nonumber \\  &   \quad -M_-(-y_0\pm i\varepsilon )M_+(1-y_0\pm i\varepsilon )\Big ). \end{aligned}$$

Furthermore, we have the relation ${\mathcal {G}}_{m,\varepsilon }^+(y,y_0,z)=\overline{{\mathcal {G}}_{m,\varepsilon }^-(y,y_0,z)}$, for all $y,y_0,z\in [0,1]$.

#### Proof

We introduce the variables $\tilde{y}_\pm =2m(y-y_0\pm i\varepsilon )$ and $\tilde{z}_\pm =2m(z-y_0\pm i\varepsilon )$ and we write ${\mathcal {G}}_{m,\varepsilon }^\pm (y,y_0,z)={\mathcal {G}}(\tilde{y}_\pm ,\tilde{z}_\pm )$ and rewrite (2.7) as

$$\begin{aligned} \partial ^2_{\tilde{y}_\pm }{\mathcal {G}}+ \left( -\frac{1}{4}+\frac{1/4-(1/4-\beta ^2)}{\tilde{y}_\pm ^2}\right) {\mathcal {G}}=\frac{1}{4m^2}\delta \left( \frac{1}{2m}\left( \tilde{y}_\pm -\tilde{z}_\pm \right) \right) . \end{aligned}$$

The left-hand side above has precisely the form of (3.2), and therefore the general solution is given in terms of the homogeneous solutions in (3.4)–(3.5) by

$$\begin{aligned} {\mathcal {G}}_{m,\varepsilon }^\pm (y,y_0,z)= {\left\{ \begin{array}{ll}C_1(\widetilde{z}_\pm )\phi _{u,m,\varepsilon }^\pm (y,y_0), & 0\le z\le y\le 1,\\ C_2(\widetilde{z}_\pm )\phi _{l,m,\varepsilon }^\pm (y,y_0), & 0\le y\le z\le 1, \end{array}\right. } \end{aligned}$$

where $C_i$ are constants to be determined. Imposing the continuity and jump conditions of the Green’s function, together with basic properties of the Whittaker functions [26], we obtain the desired result. $\square$

We also record the following proposition regarding homogeneous solutions to (TGh).

#### Proposition 3.2

The unique solutions to the homogeneous TGh) for $\varepsilon =0$ and $y_0=0,1$ with homogeneous Dirichlet boundary conditions at $y=0,1$ are given by

3.7 $$\begin{aligned} \phi _{u,m}(y):=M_+(1)M_-(y)-M_-(1)M_+(y) \end{aligned}$$

and

3.8 $$\begin{aligned} \phi _{l,m}(y):=M_+(1)M_-(1-y)-M_-(1)M_+(1-y). \end{aligned}$$

### The case $\beta ^2= 1/4$

We next provide the Green’s function to the Taylor–Goldstein equation in the case $\beta ^2=1/4$. In this case, the Whittaker equation (3.1) has to be taken for $a=b=0$, and $M_0(\zeta ):=M_{0,0}(2m\zeta )$ satisfies

3.9 $$\begin{aligned} \partial ^2_{\zeta } M_0+\left( -{\frac{1}{4}} +{\frac{1/4}{4m^2\zeta ^{2}}}\right) M_0=0, \qquad \zeta \in {{\mathbb {C}}}. \end{aligned}$$

The second independent homogeneous solution from which we build the Green’s function is given by $W_0(\zeta ):=W_{0,0}(2m\zeta )$, defined to be the unique solution to (3.9) such that

$$\begin{aligned} W_{0,0}(\zeta )=\sqrt{\tfrac{\zeta }{\pi }}\left( 2\log (2) + \varsigma -\log (\zeta )\right) + O\left( \zeta ^\frac{3}{2}\log (\zeta )\right) , \end{aligned}$$

as $\zeta \rightarrow 0$, where $\varsigma$ denotes the Euler constant. Apart from the introduction of $W_0$, the result here is similar to that in Proposition 3.1.

#### Proposition 3.3

Let $\varepsilon \in (0,1)$. The Green’s function ${\mathcal {G}}_{m,\varepsilon }^\pm$ of ${\textsc {TG}}_{m,\varepsilon }^\pm$ is given by

3.10 $$\begin{aligned} {\mathcal {G}}_{m,\varepsilon }^\pm (y,y_0,z)=\frac{1}{{\mathcal {W}}_{m,\varepsilon }^\pm (y_0)} {\left\{ \begin{array}{ll}\phi _{u,m,\varepsilon }^\pm (y,y_0)\phi _{l,m,\varepsilon }^\pm (z,y_0)& 0\le z\le y\le 1,\\ \phi _{u,m,\varepsilon }^\pm (z,y_0)\phi _{l,m,\varepsilon }^\pm (y,y_0)& 0\le y\le z\le 1,\end{array}\right. } \end{aligned}$$

where $\phi _{u,m,\varepsilon }^\pm (\cdot ,y_0)$ and $\phi _{l,m,\varepsilon }^\pm (\cdot ,y_0)$ are two homogeneous solutions to TGh) such that $\phi _{u,m,\varepsilon }^\pm (1,y_0)=0$ and $\phi _{l,m,\varepsilon }^\pm (0,y_0)=0$, respectively, for all $y_0\in [0,1]$. They are explicitly given by

3.11 $$\begin{aligned} \phi _{u,m,\varepsilon }^\pm (y,y_0):=W_0(1-y_0\pm i \varepsilon )M_0(y-y_0\pm i\varepsilon )-M_0(1-y_0\pm i\varepsilon )W_0(y-y_0\pm i\varepsilon ) \nonumber \\ \end{aligned}$$

and

3.12 $$\begin{aligned} \phi _{l,m,\varepsilon }^\pm (y,y_0):=W_0(-y_0\pm i \varepsilon )M_0(y-y_0\pm i\varepsilon )-M_0(-y_0\pm i\varepsilon )W_0(y-y_0\pm i\varepsilon ). \nonumber \\ \end{aligned}$$

with Wronskian

3.13 $$\begin{aligned} {\mathcal {W}}_{m,\varepsilon }^\pm (y_0):=\frac{2m}{\sqrt{\pi }}\Big (W_0(-y_0\pm i\varepsilon )M_0(1-y_0\pm i\varepsilon )-M_0(-y_0\pm i\varepsilon )W_0(1-y_0\pm i\varepsilon )\Big ).\nonumber \\ \end{aligned}$$

Furthermore, we have the relation ${\mathcal {G}}_{m,\varepsilon }^+(y,y_0,z)=\overline{{\mathcal {G}}_{m,\varepsilon }^-(y,y_0,z)}$, for all $y,y_0,z\in [0,1]$.

Similarly, we state the following proposition regarding homogeneous solutions to (TGh) when $\beta ^2=1/4$.

#### Proposition 3.4

The unique solutions to the homogeneous TGh) for $\varepsilon =0$ and $y_0=0,1$ with homogeneous Dirichlet boundary conditions at $y=0,1$ are given by

3.14 $$\begin{aligned} \phi _{u,m}(y):=W_0(1)M_0(y)-M_0(1)W_0(y) \end{aligned}$$

and

3.15 $$\begin{aligned} \phi _{l,m}(y):=W_0(1)M_0(1-y)-M_0(1)W_0(1-y). \end{aligned}$$

### Derivative formulae for solutions to the Taylor–Goldstein equation

We finish this section by exhibiting the following useful expressions for various derivatives of $\psi _{m,\varepsilon }^\pm$ and $\rho _{m,\varepsilon }^\pm$.

#### Proposition 3.5

Let $$\varepsilon \in (0,1)$$. Then,

3.16 $$\begin{aligned} \begin{aligned} \partial _{y_0}\psi _{m,\varepsilon }^\pm (y,y_0)&=-\frac{1}{\beta ^2}{\omega }_m^0(y) + \Big . {\mathcal {B}}_{m,\varepsilon }^\pm (y,y_0,z)\Big ]_{z=0}^{z=1} -\int _0^1\partial _y{\mathcal {G}}_{m,\varepsilon }^\pm (y,y_0,z)F_{m,\varepsilon }^\pm (z,y_0)\textrm{d}z \\&\quad + \int _0^1{\mathcal {G}}_{m,\varepsilon }^\pm (y,y_0,z)\left( \partial _zF_{m,\varepsilon }^\pm (z,y_0)+\partial _{y_0}F_{m,\varepsilon }^\pm (z,y_0)\right) \textrm{d}z, \end{aligned} \nonumber \\ \end{aligned}$$

where $${\mathcal {B}}_{m,\varepsilon }^\pm (y,y_0):=(\partial _y+\partial _{y_0})^2\varphi _{m,\varepsilon }^\pm (y,y_0)$$. Moreover,

3.17 $$\begin{aligned} \begin{aligned} \partial ^2_{y_0}\psi _{m,\varepsilon }(y,y_0)&= F_{m,\varepsilon }^\pm (y,y_0) -2\Big . \partial _y {\mathcal {B}}_{m,\varepsilon }^\pm (y,y_0,z)\Big ]_{z=0}^{z=1} + \Big . \widetilde{{\mathcal {B}}_{m,\varepsilon }^\pm }(y,y_0,z)\Big ]_{z=0}^{z=1}\\&\quad + \left( m^2-\frac{\beta ^2}{(y-y_0\pm i\varepsilon )^2}\right) \int _0^1{\mathcal {G}}_{m,\varepsilon }^\pm (y,y_0,z)F_{m,\varepsilon }^\pm (z,y_0)\textrm{d}z \\&\quad - 2\int _0^1\partial _y{\mathcal {G}}_{m,\varepsilon }^\pm (y,y_0,z)\left( \partial _z+\partial _{y_0}\right) F_{m,\varepsilon }^\pm (z,y_0) \textrm{d}z \\&\quad +\int _0^1{\mathcal {G}}_{m,\varepsilon }^\pm (y,y_0,z)\left( \partial _z+\partial _{y_0}\right) ^2F_{m,\varepsilon }^\pm (z,y_0) \textrm{d}z, \end{aligned} \nonumber \\ \end{aligned}$$

where $$\widetilde{{\mathcal {B}}_{m,\varepsilon }^\pm }(y,y_0,z):=\partial _z{\mathcal {G}}_{m,\varepsilon }^\pm (y,y_0,z)\left( \partial _z + \partial _{y_0}\right) ^2\varphi _{m,\varepsilon }^\pm (z,y_0)$$. Additionally,

3.18 $$\begin{aligned} \partial _{y}\psi _{m,\varepsilon }^\pm (y,y_0)\!=\!\frac{1}{\beta ^2}\left( {\omega }_m^0(y)+(y-y_0\pm i\varepsilon )\partial _y{\omega }_m^0(y)\right) -\partial _y\rho _m^0(y)\!+\! \partial _y\varphi _{m,\varepsilon }^\pm (y,y_0) \nonumber \\ \end{aligned}$$

and

3.19 $$\begin{aligned} \begin{aligned} \partial _{y_0,y}^2\psi _{m,\varepsilon }^\pm (y,y_0)&=-\frac{1}{\beta ^2}\partial _y{\omega }_m^0(y) - F_{m,\varepsilon }^\pm (y,y_0) +\partial _y {\mathcal {B}}_{m,\varepsilon }^\pm (y,y_0,z)\Big ]_{z=0}^{z=1} \\&\quad + \int _0^1\partial _y{\mathcal {G}}_{m,\varepsilon }^\pm (y,y_0,z)\left( \partial _zF_{m,\varepsilon }^\pm (z,y_0)+\partial _{y_0}F_{m,\varepsilon }^\pm (z,y_0)\right) \textrm{d}z \\&\quad -\left( m^2-\frac{\beta ^2}{(y-y_0\pm i\varepsilon )^2}\right) \int _0^1{\mathcal {G}}_{m,\varepsilon }^\pm (y,y_0,z)F_{m,\varepsilon }^\pm (z,y_0)\textrm{d}z. \end{aligned} \nonumber \\ \end{aligned}$$

#### Proof

The formula for $\partial _y\psi _{m,\varepsilon }^\pm$ follows from taking a $\partial _y$ derivative in (2.9). Similarly, once $\partial _{y_0}\psi _{m,\varepsilon }^\pm$ is established, the expression for $\partial _{y_0,y}\psi _{m,\varepsilon }^\pm$ follows from taking a $\partial _y$ derivative in (3.16) and noting that ${\mathcal {G}}_{m,\varepsilon }^\pm$ is the Green’s function of the Taylor–Goldstein operator. As for $\partial _{y_0}\psi _{m,\varepsilon }^\pm$ and $\partial _{y_0}^2\psi _{m,\varepsilon }$ we show these expressions using the Taylor–Goldstein equation and taking $y_0$ and *y* derivatives there. More precisely, note that $\partial _y+\partial _{y_0}$ commutes with the Taylor–Goldstein operator (TGh). As such, ${\textsc {TG}}_{m,\varepsilon }^\pm \left( \partial _y + \partial _{y_0}\right) \varphi _{m,\varepsilon }^\pm =\left( \partial _y + \partial _{y_0}\right) F_{m,\varepsilon }^\pm$ and the first part of the lemma follows, upon noting that

$$\begin{aligned} \partial _{y_0}\psi _{m,\varepsilon }^\pm =-\frac{1}{\beta ^2}{\omega }_m^0+\partial _{y_0}\varphi _{m,\varepsilon }^\pm \end{aligned}$$

and that

$$\begin{aligned}  &   \int _0^1 {\mathcal {G}}_{m,\varepsilon }^\pm (y,y_0,z){\textsc {TG}}_{m,\varepsilon }^\pm \partial _z\varphi _{m,\varepsilon }^\pm (z,y_0)\textrm{d}z \\  &   \quad = {-\left. \partial _z{\mathcal {G}}_{m,\varepsilon }^\pm (y,y_0,z)\partial _z\varphi _{m,\varepsilon }^\pm (z,y_0)\right| _{z=0}^{z=1}} + \partial _y\varphi _{m,\varepsilon }^\pm (y,y_0). \end{aligned}$$

As for the second part of the lemma, ${\textsc {TG}}_{m,\varepsilon }^\pm \left( \partial _y + \partial _{y_0}\right) ^2 \varphi _{m,\varepsilon }^\pm =\left( \partial _y + \partial _{y_0}\right) ^2F_{m,\varepsilon }^\pm$, from which we deduce that

$$\begin{aligned} \partial _{y_0}\left( \partial _y + \partial _{y_0}\right) \varphi _{m,\varepsilon }^\pm&=-\partial _{y}\left( \partial _y + \partial _{y_0}\right) \varphi _{m,\varepsilon }^\pm +\Big [ \partial _z {\mathcal {G}}_{m,\varepsilon }^\pm (y,y_0,z)(\partial _{y_0}+\partial _z)^2\varphi _{m,\varepsilon }^\pm (z,y_0)\Big ]_{z=0}^{z=1} \\&\quad + \int _0^1{\mathcal {G}}_{m,\varepsilon }^\pm (y,y_0,z)\left( \partial _z+\partial _{y_0}\right) ^2F_{m,\varepsilon }^\pm (z,y_0)\textrm{d}z. \end{aligned}$$

Now, since $(\partial _{y_0}+\partial _y)^2\varphi _{m,\varepsilon }^\pm =\partial _{y_0}^2\varphi _{m,\varepsilon }^\pm +2\partial _{y_0}\partial _y\varphi _{m,\varepsilon }^\pm + \partial _y^2\varphi _{m,\varepsilon }^\pm$, we observe that for $y=0$ and $y=1$,

$$\begin{aligned} \begin{aligned} (\partial _{y_0}+\partial _y)^2\varphi _{m,\varepsilon }^\pm (y,y_0)&=-\frac{2}{\beta ^2}\partial _y{\omega }_m^0(y) -F_{m,\varepsilon }^\pm (y,y_0) \\&\quad +2\partial _y\Big [ \partial _z{\mathcal {G}}_{m,\varepsilon }^\pm (y,y_0,z)\partial _z\varphi _{m,\varepsilon }^\pm (z,y_0)\Big ]_{z=0}^{z=1}\\&\quad +2 \int _0^1\partial _y{\mathcal {G}}_{m,\varepsilon }^\pm (y,y_0,z)\left( \partial _z+\partial _{y_0}\right) F_{m,\varepsilon }^\pm (z,y_0)\textrm{d}z \\ \end{aligned} \end{aligned}$$

Moreover, from ${\textsc {TG}}_{m,\varepsilon }^\pm \left( \partial _y + \partial _{y_0}\right) \varphi _{m,\varepsilon }^\pm =\left( \partial _y+\partial _{y_0}\right) F_{m,\varepsilon }^\pm$ we can also obtain

$$\begin{aligned} \left( \partial _y + \partial _{y_0}\right) \varphi _{m,\varepsilon }^\pm= &   \Big [ \partial _z{\mathcal {G}}_{m,\varepsilon }^\pm (y,y_0,z)\partial _y\varphi _{m,\varepsilon }^\pm (y,y_0)\Big ]_{z=0}^{z=1}\\  &   \quad + \int _0^1 {\mathcal {G}}_{m,\varepsilon }^\pm (y,y_0,z)(\partial _{z}+\partial _{y_0})F_{m,\varepsilon }^\pm (z,y_0)\textrm{d}z, \end{aligned}$$

so that

$$\begin{aligned} \partial _y \left( \partial _y + \partial _{y_0}\right) \varphi _{m,\varepsilon }^\pm&=\partial _y\Big [ \partial _z{\mathcal {G}}_{m,\varepsilon }^\pm (y,y_0,z)\partial _y\varphi _{m,\varepsilon }^\pm (y,y_0)\Big ]_{z=0}^{z=1} \\&\quad + \int _0^1 \partial _y{\mathcal {G}}_{m,\varepsilon }^\pm (y,y_0,z)(\partial _{z}+\partial _{y_0})F_{m,\varepsilon }^\pm (z,y_0)\textrm{d}z. \end{aligned}$$

We finish with the observation that $(\partial _{y_0}-\partial _y)\left( \partial _y + \partial _{y_0}\right) \varphi _{m,\varepsilon }^\pm =(\partial _{y_0}^2-\partial _y^2)\varphi _{m,\varepsilon }^\pm$, that is,

$$\begin{aligned} \partial _{y_0}^2\varphi _{m,\varepsilon }^\pm&=\partial _y^2\varphi _{m,\varepsilon }^\pm + (\partial _{y_0}-\partial _y)\left( \partial _y + \partial _{y_0}\right) \varphi _{m,\varepsilon }^\pm \\&=\left( m^2 -\beta ^2\frac{1}{(y-y_0+i\varepsilon )^2}\right) \varphi _{m,\varepsilon }^\pm + F_{m,\varepsilon }^\pm + \partial _{y_0}\left( \partial _y + \partial _{y_0}\right) \varphi _{m,\varepsilon }^\pm - \partial _y \left( \partial _y + \partial _{y_0}\right) \varphi _{m,\varepsilon }^\pm . \end{aligned}$$

Gathering the previously obtained terms, the second part of the lemma follows, since $\partial ^2_{y_0}\psi _{m,\varepsilon }^\pm =\partial ^2_{y_0}\varphi _{m,\varepsilon }^\pm$. $\square$

With the same ideas as above, we can also find useful expressions for $\partial _{y_0}\rho _{m,\varepsilon }^\pm$ and $\partial _y\rho _{m,\varepsilon }^\pm$, thanks again to (2.5).

#### Corollary 3.6

Let $$\varepsilon \in (0,1)$$. Then,

3.20 $$\begin{aligned} \begin{aligned} \partial _{y_0}\rho _{m,\varepsilon }^\pm (y,y_0)&= \frac{1}{(y-y_0\pm i\varepsilon )^2}\int _0^1 {\mathcal {G}}^\pm _{m,\varepsilon }(y,y_0,z) F_{m,\varepsilon }^\pm (z,y_0)\textrm{d}z\\&\quad + \frac{1}{y-y_0\pm i\varepsilon }\Big . {\mathcal {B}}_{m,\varepsilon }^\pm (y,y_0,z)\Big ]_{z=0}^{z=1}\\&\quad + \frac{1}{y-y_0\pm i\varepsilon }\int _0^1 {\mathcal {G}}^\pm _{m,\varepsilon }(y,y_0,z) \left( \partial _zF_{m,\varepsilon }^\pm (z,y_0) + \partial _{y_0}F_{m,\varepsilon }^\pm (z,y_0)\right) \textrm{d}z \\&\quad - \frac{1}{y-y_0\pm i\varepsilon }\int _0^1 \partial _y{\mathcal {G}}^\pm _{m,\varepsilon }(y,y_0,z) F_{m,\varepsilon }^\pm (z,y_0)\textrm{d}z, \end{aligned} \nonumber \\ \end{aligned}$$

and

3.21 $$\begin{aligned} \begin{aligned} \partial _{y}\rho _{m,\varepsilon }^\pm (y,y_0)&= \frac{1}{\beta ^2}\partial _y{\omega }_m^0(y) -\frac{1}{(y-y_0\pm i\varepsilon )^2}\int _0^1 {\mathcal {G}}^\pm _{m,\varepsilon }(y,y_0,z) F_{m,\varepsilon }^\pm (z,y_0)\textrm{d}z \\&\quad + \frac{1}{y-y_0\pm i\varepsilon }\int _0^1 \partial _y{\mathcal {G}}^\pm _{m,\varepsilon }(y,y_0,z) F_{m,\varepsilon }^\pm (z,y_0)\textrm{d}z. \end{aligned} \nonumber \\ \end{aligned}$$

## Bounds on the Green’s Function for $\beta ^{2} \ne 1/4$

This section is devoted to the proof of Theorem 2, which provides $L^2$ bounds on the Green’s function ${\mathcal {G}}_{m,\varepsilon }^\pm$. We separate the estimates into bounds near the critical layer (Sect. 4.1) and away from the critical layer (Sect. 4.2). We wrap up the proof in Sect. 4.3.

### Pointwise bounds near the critical layer

The aim is to provide pointwise bounds for the Green’s function and its $\partial _y$ derivative when both *y* and *z* variables are close to the spectral variable $y_0$.

#### Proposition 4.1

Let $y,y_0,z\in [0,1]$ such that $m|y-y_0+i\varepsilon |\le 10\beta$ and $m|z-y_0+i\varepsilon |\le 10\beta$. There exists $\varepsilon _0>0$ such that

$$\begin{aligned} |{\mathcal {G}}_{m,\varepsilon }^+(y,y_0,z)|\lesssim m^{-2\mu } |y-y_0+i\varepsilon |^{\frac{1}{2}-\mu }|z-y_0+i\varepsilon |^{\frac{1}{2}-\mu } \end{aligned}$$

and

$$\begin{aligned} |\partial _y{\mathcal {G}}_{m,\varepsilon }^+(y,y_0,z)|\lesssim m^{-2\mu } |y-y_0+i\varepsilon |^{-\frac{1}{2}-\mu }|z-y_0+i\varepsilon |^{\frac{1}{2}-\mu }, \end{aligned}$$

for all $$\varepsilon \le \varepsilon _0$$.

The proofs depend heavily on the Wronskian associated to the Green’s function ${\mathcal {G}}_{m,\varepsilon }^\pm (y,y_0,z)$ and whether $\beta ^2>1/4$ or not. We begin with the case in which $\beta ^2>1/4$, for which $\mu =0$ and $\nu >0$.

#### Proposition 4.2

Let $\beta ^2>1/4$. Within the assumptions of Proposition 4.1, there exists $\varepsilon _0>0$ such that

$$\begin{aligned} |{\mathcal {G}}_{m,\varepsilon }^+(y,y_0,z)|\lesssim |y-y_0+i\varepsilon |^{\frac{1}{2}}|z-y_0+i\varepsilon |^{\frac{1}{2}} \end{aligned}$$

and

$$\begin{aligned} |\partial _y{\mathcal {G}}_{m,\varepsilon }^+(y,y_0,z)|\lesssim |y-y_0+i\varepsilon |^{-\frac{1}{2}}|z-y_0+i\varepsilon |^{\frac{1}{2}}, \end{aligned}$$

for all $$\varepsilon \le \varepsilon _0$$.

#### Proof

Let us assume that $y\le z$. Then (3.3) tells us that

4.1 $$\begin{aligned} {\mathcal {G}}_{m,\varepsilon }^+(y,y_0,z)=\frac{1}{W_{m,\varepsilon }^\pm (y_0)}\phi _{u,m,\varepsilon }^+(z,y_0)\phi _{l,m,\varepsilon }^+(y,y_0) \end{aligned}$$

and we have from Lemma A.3 that

$$\begin{aligned} |\phi _{u,m,\varepsilon }^+(z,y_0)|\lesssim m^\frac{1}{2}|z-y_0+i\varepsilon |^\frac{1}{2}\big ( |M_-(1-y_0+i\varepsilon )| + |M_+(1-y_0+i\varepsilon )|\big ), \end{aligned}$$

while

$$\begin{aligned} |\phi _{l,m,\varepsilon }^+(y,y_0)|\lesssim m^\frac{1}{2}|y-y_0+i\varepsilon |^\frac{1}{2}\big ( |M_-(y_0-i\varepsilon )| + |M_+(y_0-i\varepsilon )|\big ). \end{aligned}$$

The proof follows once we show that

4.2 $$\begin{aligned} |{\mathcal {W}}_{m,\varepsilon }^+(y_0)|\ge C_\nu m|M_-(y_0-i\varepsilon )||M_+(1-y_0+i\varepsilon )| \end{aligned}$$

and

4.3 $$\begin{aligned} \frac{|M_+(y_0-i\varepsilon )|}{|M_-(y_0-i\varepsilon )|}+ \frac{|M_-(1-y_0+i\varepsilon )|}{|M_+(1-y_0+i\varepsilon )|}\lesssim 1. \end{aligned}$$

To prove the lower bound on the Wronskian, we begin by writing out a suitable expression for ${\mathcal {W}}^+_{m,\varepsilon }(y_0)$, where we have used the analytic continuation properties of the Whittaker functions $M_\pm$:

$$\begin{aligned} \begin{aligned} {\mathcal {W}}^+_{m,\varepsilon }(y_0)&=4i\nu m\Big ( \textrm{e}^{\nu \pi }M_-(y_0-i\varepsilon )M_+(1-y_0+i\varepsilon ) -\textrm{e}^{-\nu \pi }M_+(y_0-i\varepsilon )M_-(1-y_0+i\varepsilon )\Big ) \\&=4i\nu mM_-(y_0-i\varepsilon )M_+(1-y_0+i\varepsilon ) \\&\quad \left( \textrm{e}^{\nu \pi } - \textrm{e}^{-\nu \pi }\frac{M_+(y_0-i\varepsilon )}{M_-(y_0-i\varepsilon )}\frac{M_-(1-y_0+i\varepsilon )}{M_+(1-y_0+i\varepsilon )}\right) . \end{aligned} \end{aligned}$$

The proof depends on the location of $y_0\in [0,1]$ as well as on the smallness of *m*. In this direction, let $N_\nu >0$ given in Lemma A.6.

$\bullet$ **Case 1:** $m< N_\nu .$ Assume that $y_0\le 1/2$ (otherwise we would have $1-y_0\le 1/2$ and the proof would carry over unaltered). Therefore, it follows that $2\,m y_0 < N_\nu$ and $m\le 2\,m(1-y_0) < 2N_\nu$. Hence, there exists $\varepsilon _0>0$ such that from Lemma A.7

$$\begin{aligned} \left| \frac{M_+(y_0-i\varepsilon )}{M_+(y_0+i\varepsilon )}\right| \le \textrm{e}^{\frac{5}{4}\nu \pi }, \end{aligned}$$

and from Lemma A.8

$$\begin{aligned} \frac{|M_-(1-y_0+i\varepsilon )|}{|M_+(1-y_0+i\varepsilon )|}\le \textrm{e}^{\frac{1}{4}\nu \pi }, \end{aligned}$$

for all $\varepsilon \le \varepsilon _0$. Moreover,

$$\begin{aligned} |{\mathcal {W}}_{m,\varepsilon }^+(y_0)|\ge C_\nu m|M_-(y_0-i\varepsilon )||M_+(1-y_0+i\varepsilon )| \end{aligned}$$

for $$C_\nu =4\nu (\textrm{e}^{\nu \pi }-\textrm{e}^{\nu \pi /2})$$.

$\bullet$ **Case 2:** $m\ge N_\nu .$ Assume now that $2m y_0\le N_\nu$. Then, since $m\ge N_\nu$ we have that $2m(1-y_0)\ge N_\nu$. The other case is completely analogous and $m\ge N_\nu$ ensures that $2my_0<N_\nu$ and $2\,m(1-y_0)<N_\nu$ cannot hold simultaneously for any $y_0\in [0,1]$. Therefore, it follows from Lemma A.7 that

$$\begin{aligned} \left| \frac{M_+(y_0-i\varepsilon )}{M_+(y_0+i\varepsilon )}\right| \le \textrm{e}^{\frac{5}{4}\nu \pi }, \end{aligned}$$

while from Lemma A.6 we obtain

$$\begin{aligned} \left| \frac{M_-(1-y_0+i\varepsilon )}{M_+(1-y_0+i\varepsilon )}\right| \le \textrm{e}^{\frac{1}{4}\nu \pi }, \end{aligned}$$

for all $\varepsilon \le \varepsilon _0$, for some $\varepsilon _0>0$. The lower bound on $|{\mathcal {W}}^+_{m,\varepsilon }(y_0)|$ holds for the same $C_\nu$ as above. $\square$

We next consider the case $\beta ^2<1/4$, for which $\nu =0$ and $0<\mu <1/2$.

#### Proposition 4.3

Let $\beta ^2<1/4$. Within the assumptions of Proposition 4.1, there exists $\varepsilon _0>0$ such that

$$\begin{aligned} |{\mathcal {G}}_{m,\varepsilon }^+(y,y_0,z)|\lesssim m^{-2\mu } |y-y_0+i\varepsilon |^{\frac{1}{2}-\mu }|z-y_0+i\varepsilon |^{\frac{1}{2}-\mu } \end{aligned}$$

and

$$\begin{aligned} |\partial _y{\mathcal {G}}_{m,\varepsilon }^+(y,y_0,z)|\lesssim m^{-2\mu } |y-y_0+i\varepsilon |^{-\frac{1}{2}-\mu }|z-y_0+i\varepsilon |^{\frac{1}{2}-\mu }, \end{aligned}$$

for all $$\varepsilon \le \varepsilon _0$$.

#### Proof

Let us assume that $y\le z$ and deal with the expression in (4.1). From Lemma A.3 we have

$$\begin{aligned} |\phi _{u,m,\varepsilon }^+(z,y_0)|\lesssim m^{\frac{1}{2}-\mu }|z-y_0+i\varepsilon |^{\frac{1}{2}-\mu }\big ( |M_-(1-y_0+i\varepsilon )| + |M_+(1-y_0+i\varepsilon )|\big ), \end{aligned}$$

while

$$\begin{aligned} |\phi _{l,m,\varepsilon }^+(y,y_0)|\lesssim m^{\frac{1}{2}-\mu }|y-y_0+i\varepsilon |^{\frac{1}{2}-\mu }\big ( |M_-(y_0-i\varepsilon )| + |M_+(y_0-i\varepsilon )|\big ), \end{aligned}$$

both following from the observation that $(2m|y-y_0+i\varepsilon |)^{2\mu }\le 10$. Using the analytic continuation properties of the Whittaker functions $M_\pm$ we obtain

$$\begin{aligned} \begin{aligned}&{\mathcal {W}}_{m,\varepsilon }^+(y_0)\\&\quad =4\mu m\left( \textrm{e}^{i\mu \pi }M_+(y_0-i\varepsilon )M_-(1-y_0+i\varepsilon )-\textrm{e}^{-i\mu \pi }M_-(y_0-i\varepsilon )M_+(1-y_0+i\varepsilon )\right) \\&\quad =-4\mu m\left( \textrm{e}^{-i\mu \pi }M_-(y_0-i\varepsilon )M_+(1-y_0+i\varepsilon ) - \textrm{e}^{i\mu \pi }M_+(y_0-i\varepsilon )M_-(1-y_0+i\varepsilon )\right) \end{aligned} \end{aligned}$$

One needs to obtain suitable estimates on several quotients. This is again done considering the location of $y_0\in [0,1]$ and the smallness of *m*. Thus, let $N_{\mu ,0}>0$ given in Lemma A.15.

$\bullet$ **Case 1:** $m\le N_{\mu ,0}.$ Assume initially that $y_0\le \frac{1}{2}$. Then, $2my_0\le N_{\mu ,0}$ and $m\le 2\,m(1-y_0)\le 2N_{\mu ,0}$. Assume further that $2my_0\le \delta _{\mu ,1}$ as given in Lemma A.16. From Lemma A.17 choosing $N_{\mu ,1}:=m$ and Lemma A.5 we have that

$$\begin{aligned} \frac{3}{4} \le \left| \frac{M_+(1-y_0+i\varepsilon )}{M_+(1-y_0)} \right| \le \frac{5}{4}, \end{aligned}$$

and from Lemma A.16

$$\begin{aligned} \left| \frac{M_-(1-y_0+i\varepsilon )}{M_+(1-y_0)} \right| = \left| \frac{M_-(1-y_0+i\varepsilon )}{M_-(1-y_0)}\right| \left| \frac{M_-(1-y_0)}{M_+(1-y_0)} \right| \le \frac{5}{4} M\left( \frac{1}{2}-\mu ,1-2\mu ,2N_{\mu ,0}\right) , \end{aligned}$$

for $\varepsilon \le \varepsilon _0$ small enough. Additionally, since $2my_0\le \delta _{\mu ,1}$, we have from Lemma A.16 that

$$\begin{aligned} \left| \frac{M_+(y_0-i\varepsilon )}{M_-(y_0-i\varepsilon )} \right| \le \frac{1}{5M\left( \frac{1}{2}-\mu ,1-2\mu ,2N_1\right) }. \end{aligned}$$

With the above comparison estimates at hand, we note that

$$\begin{aligned} \begin{aligned}&\textrm{e}^{-i\mu \pi } M_-(y_0-i\varepsilon )M_+(1-y_0+i\varepsilon ) - \textrm{e}^{i\mu \pi }M_+(y_0-i\varepsilon )M_-(1-y_0+i\varepsilon ) \\&\quad =\textrm{e}^{-i\mu \pi }M_-(y_0-i\varepsilon )M_+(1-y_0)\\&\qquad \left( \frac{M_+(1-y_0+i\varepsilon )}{M_+(1-y_0)} - \textrm{e}^{2i\mu \pi }\frac{M_+(y_0-i\varepsilon )}{M_-(y_0-i\varepsilon )}\frac{M_-(1-y_0+i\varepsilon )}{M_+(1-y_0)} \right) \\ \end{aligned} \end{aligned}$$

and therefore we can lower bound

$$\begin{aligned} |{\mathcal {W}}_{m,\varepsilon }^+(y_0)|\ge 2\mu m M_+(1-y_0)|M_-(y_0-i\varepsilon )|. \end{aligned}$$

The bounds on the Green’s functions follow from the lower bound on the Wronskian and the comparison estimates stated above.

Assume now that $2my_0 > \delta _{\mu ,1}$. Then, due to Lemma A.17 we have both

$$\begin{aligned} \left| M_\pm (y_0-i\varepsilon )-M_\pm (y_0)\right| \le \frac{\sin \mu \pi }{4}\left| M_\pm (y_0)\right| \end{aligned}$$

and

$$\begin{aligned} \left| M_\pm (1-y_0+i\varepsilon )-M_\pm (1-y_0)\right| \le \frac{\sin \mu \pi }{4}\left| M_\pm (1-y_0)\right| , \end{aligned}$$

for all $\varepsilon \le \varepsilon _0$. With the observation that

$$\begin{aligned} \begin{aligned}&\left| \textrm{e}^{-i\mu \pi }M_-(y_0)M_+(1-y_0) - \textrm{e}^{i\mu \pi }M_+(y_0)M_-(1-y_0) \right| \\&\qquad \ge \sin \mu \pi \big ( M_-(y_0)M_+(1-y_0) + M_+(y_0)M_-(1-y_0) \big ), \end{aligned} \end{aligned}$$

and the expansion

$$\begin{aligned} \begin{aligned}&M_-(y_0-i\varepsilon )M_+(1-y_0+i\varepsilon ) = M_-(y_0)M_+(1-y_0)\\&\quad +\big ( M_-(y_0-i\varepsilon ) - M_-(y_0) \big )M_+(1-y_0) \\&\quad + \big ( M_-(y_0-i\varepsilon ) - M_-(y_0) \big ) \big ( M_+(1-y_0+i\varepsilon )-M_+(1-y_0)\big ) \\&\quad + M_-(y_0) \big ( M_+(1-y_0+i\varepsilon )-M_+(1-y_0)\big ), \end{aligned} \end{aligned}$$

one can lower bound

$$\begin{aligned} |{\mathcal {W}}_{m,\varepsilon }^+(y_0)|\ge \mu m\sin \mu \pi \big ( M_-(y_0)M_+(1-y_0) + M_+(y_0)M_-(1-y_0) \big ). \end{aligned}$$

As before, we obtain the bound on the Green’s function combing the lower bound on the Wronskian with the above comparison estimates.

$\bullet$ **Case 2:** $m\ge N_{\mu ,0}.$ Assume $2my_0<N_{\mu ,0}$. Since $m\ge N_{\mu ,0}$ then $2m(1-y_0)\ge N_{\mu ,0}$. Assume further that $2my_0\le \delta _{\mu ,1}$ as given in Lemma A.16 and let $C_\mu :=2^{-4\mu }\frac{\Gamma (1-\mu )}{\Gamma (1+\mu )}$. Then, from Lemma A.7 we have that

$$\begin{aligned} \left| \frac{M_+(y_0-i\varepsilon )}{M_-(y_0-i\varepsilon )} \right| \le \frac{1}{3} C_\mu ^{-1}, \end{aligned}$$

while from Lemma A.15,

$$\begin{aligned} \left| \frac{M_-(1-y_0+i\varepsilon )}{M_+(1-y_0+i\varepsilon )}\right| \le \frac{3}{2} C_\mu . \end{aligned}$$

Since we can write

$$\begin{aligned}  &   {\mathcal {W}}_{m,\varepsilon }^+(y_0)=-4\mu m\textrm{e}^{-i\mu \pi }M_-(y_0-i\varepsilon )M_+(1-y_0+i\varepsilon )\\  &   \quad \left( 1 - \textrm{e}^{2i\mu \pi }\frac{M_+(y_0-i\varepsilon )}{M_-(y_0-i\varepsilon )}\frac{M_-(1-y_0+i\varepsilon )}{M_+(1-y_0+i\varepsilon )} \right) \end{aligned}$$

we are able to lower bound

$$\begin{aligned} |{\mathcal {W}}_{m,\varepsilon }^+(y_0)|\ge 2\mu m |M_-(y_0-i\varepsilon )||M_+(1-y_0+i\varepsilon )|, \end{aligned}$$

and the estimates on the Green’s function follow directly.

On the other hand, if $2my_0\ge \delta _{\mu ,1}$, we shall write

$$\begin{aligned} \begin{aligned} \textrm{e}^{-i\mu \pi }&M_-(y_0-i\varepsilon )M_+(1-y_0+i\varepsilon ) - \textrm{e}^{i\mu \pi }M_+(y_0-i\varepsilon )M_-(1-y_0+i\varepsilon ) \\&=\textrm{e}^{-i\mu \pi }M_-(y_0)M_+(1-y_0+i\varepsilon ) - \textrm{e}^{i\mu \pi }M_+(y_0)M_-(1-y_0+i\varepsilon ) \\&\quad + \textrm{e}^{-i\mu \pi }\big (M_-(y_0-i\varepsilon ) -M_-(y_0)\big )M_+(1-y_0+i\varepsilon ) \\&\quad - \textrm{e}^{i\mu \pi }\big (M_+(y_0-i\varepsilon ) -M_+(y_0)\big )M_-(1-y_0+i\varepsilon ) \\&= T_1 + T_2 + T_3. \end{aligned} \end{aligned}$$

and we note that

$$\begin{aligned} T_1=M_+(1-y_0+i\varepsilon )\left( \textrm{e}^{-i\mu \pi }M_-(y_0) - \textrm{e}^{i\mu \pi }M_+(y_0)\frac{M_-(1-y_0+i\varepsilon )}{M_+(1-y_0+i\varepsilon )} \right) , \end{aligned}$$

with

$$\begin{aligned} \begin{aligned}&\textrm{Im}\left( \textrm{e}^{-i\mu \pi }M_-(y_0) - \textrm{e}^{i\mu \pi }M_+(y_0)\frac{M_-(1-y_0+i\varepsilon )}{M_+(1-y_0+i\varepsilon )} \right) \\&\quad = -\sin \mu \pi \left( M_-(y_0) + M_+(y_0)\textrm{Re}\left( \frac{M_-(1-y_0+i\varepsilon )}{M_+(1-y_0+i\varepsilon )} \right) \right. \\&\qquad \left. + \frac{1}{\tan \mu \pi }M_+(y_0)\textrm{Im}\left( \frac{M_-(1-y_0+i\varepsilon )}{M_+(1-y_0+i\varepsilon )}\right) \right) . \end{aligned} \end{aligned}$$

Once again, from Lemma A.15, we have that

$$\begin{aligned} \left| \textrm{Re}\left( \frac{M_-(1-y_0+i\varepsilon )}{M_+(1-y_0+i\varepsilon )} \right) - C_\mu \right| + \left| \frac{1}{\tan \mu \pi }\textrm{Im}\left( \frac{M_-(1-y_0+i\varepsilon )}{M_+(1-y_0+i\varepsilon )} \right) \right| \le \frac{C_\mu }{4}, \end{aligned}$$

so that we can lower bound

$$\begin{aligned} |T_1| \ge \sin \mu \pi |M_+(1-y_0+i\varepsilon )|\left( M_-(y_0) + \frac{C_\mu }{2} M_+(y_0) \right) . \end{aligned}$$

Next, we shall see that the terms $T_2$ and $T_3$ are sufficiently small so that they can be absorbed by $T_1$. To this end, from Lemma A.17 we have that

$$\begin{aligned} |T_2|\le \frac{\sin \mu \pi }{2}|M_+(1-y_0+i\varepsilon )|M_-(y_0), \end{aligned}$$

and, combined with Lemma A.15, we also have that

$$\begin{aligned} |T_3|\le \sin \mu \pi \frac{C_\mu }{4}M_+(y_0) |M_+(1-y_0+i\varepsilon )|, \end{aligned}$$

for all $\varepsilon \le \varepsilon _0$ small enough. Hence, we conclude that

$$\begin{aligned} |T_1+T_2+T_3|\ge \sin \mu \pi \left( \frac{1}{2}M_-(y_0) +\frac{C_\mu }{4}M_+(y_0)\right) |M_+(1-y_0+i\varepsilon )| \end{aligned}$$

and we lower bound

$$\begin{aligned} |{\mathcal {W}}_{m,\varepsilon }^+(y_0)|\ge \mu m\sin \mu \pi \left( 2M_-(y_0) + C_\mu M_+(y_0)\right) |M_+(1-y_0+i\varepsilon )|. \end{aligned}$$

The bounds on the Green’s function are a straightforward consequence of the above lower bound ${\mathcal {W}}_{m,\varepsilon }^+(y_0)$ and the comparison estimates. $\square$

### Estimates for ${\mathcal {G}}_{m,\varepsilon }$ away from the critical layer

Throughout this section, let $\varepsilon _0$ be given by Proposition 4.1 and assume that $m>8\beta$. Hence, both $y_0<\tfrac{4\beta }{m}$ and $y_0>1-\tfrac{4\beta }{m}$ cannot hold simultaneously and through the section we assume without loss of generality that $y0<1-\tfrac{4\beta }{m}$.

The proof of the following results combines an entanglement inequality inspired by [18] and the estimates from Proposition 4.1. Firstly we obtain estimates when *z* is far from the critical layer, but *y* is still near the spectral variable $y_0$.

#### Lemma 4.4

Let $y_0\in [0,1]$ and $0<\varepsilon \le \varepsilon _0$. For all $z\in [0,1]$ such that $m|z-y_0|\le 9\beta$ we have the following.

$$\begin{aligned} \Vert \partial _y{\mathcal {G}}_{m,\varepsilon }^\pm (\cdot ,y_0,z)\Vert _{L^2_y(J_3^c)}^2 + m^2\Vert {\mathcal {G}}_{m,\varepsilon }^\pm (\cdot ,y_0,z)\Vert _{L^2_y(J_3^c)}^2 \lesssim m^{-2\mu }|z-y_0\pm i\varepsilon |^{1-2\mu }. \end{aligned}$$

#### Proof

Assume without loss of generality that $y_0<1-\frac{3\beta }{m}$. Let $y_2=y_0+\frac{2\beta }{m}$ and take $\eta \in C_p^1([y_2,1])$, the space of piecewise continuously differentiable functions. To ease notation, we denote $h(y):={\mathcal {G}}_{m,\varepsilon }^+(y,y_0,z)$. Hence *h*(*y*) solves

$$\begin{aligned} \left( {\Delta }_m +\beta ^2\frac{1}{(y-y_0+i\varepsilon )^2}\right) h=\delta (y-z). \end{aligned}$$

Multiplying the equation by $\overline{h}\eta ^2$ and integrating from $y_2$ to 1, we find that

$$\begin{aligned} -\overline{h}(z)\eta ^2(z)\mathcal {H}(z-y_2)=\int _{y_2}^1 |\partial _y h|^2\eta ^2 + 2\partial _yh \overline{h}\partial _y\eta \eta +m^2|h|^2\eta ^2 -\beta ^2\frac{|h|^2\eta ^2}{(y-y_0+i\varepsilon )^2} \, \textrm{d}y \end{aligned}$$

and thus

$$\begin{aligned} |\overline{h}(z)\eta ^2(z)\mathcal {H}(z-y_2)| \ge \int _{y_2}^1 \frac{1}{2}|\partial _yh|^2\eta ^2 +\left( \frac{m^2}{2}\eta ^2-2(\partial _y\eta )^2\right) |h|^2\, \textrm{d}y, \end{aligned}$$

where we have used Young’s inequality and $m|y-y_0+i\varepsilon |\ge 2\beta$, for all $y\ge y_2$. Here, $\mathcal {H}$ represents the Heavyside function. Now, we shall choose $\eta$ as follows:

$$\begin{aligned} \eta (y)= {\left\{ \begin{array}{ll} \frac{m}{\beta }(y-y_2),& y\in (y_2,y_2+\frac{\beta }{m}),\\ 1,& y\in (y_2+\frac{\beta }{m}, 1)\end{array}\right. }. \end{aligned}$$

Note that $\eta$ is a piecewise $C^1$ function such that it is a linear function in $(y_2,y_2+\frac{\beta }{m})$ and it is constant in $(y_2+\frac{\beta }{m}, 1)$. Hence,

$$\begin{aligned} |h(z)|+\frac{m^2}{\beta ^2}\int _{y_2}^{y_2+\frac{\beta }{m}}|h|^2\textrm{d}y \ge \frac{1}{2} \int _{y_2+\frac{\beta }{m}}^1 \left( |\partial _y h|^2 + m^2 |h|^2\right) \textrm{d}y. \end{aligned}$$

Using Proposition 4.1, we can estimate

$$\begin{aligned} \begin{aligned} |h(z)| + \frac{m^2}{\beta ^2}\int _{y_2}^{y_2+\frac{\beta }{m}}|h(y,y_0,z)|^2\textrm{d}y&\lesssim m^{-4\mu }|z-y_0+i\varepsilon |^{1-2\mu }\\&\quad \left( 1+ \frac{m^2}{\beta ^2}\int _{y_2}^{y_2+\frac{\beta }{m}}|y-y_0+i\varepsilon |^{1-2\mu }\textrm{d}y\right) \\&\lesssim m^{-2\mu }|z-y_0+i\varepsilon |^{1-2\mu }. \end{aligned} \end{aligned}$$

Therefore, since $y_2=y_0+\frac{2\beta }{m}$ we have the bound

$$\begin{aligned} \int _{y_0+\frac{3\beta }{m}}^1 \left( |\partial _y h|^2 + m^2 |h|^2\right) \textrm{d}y \lesssim m^{-2\mu }|z-y_0+i\varepsilon |^{1-2\mu }. \end{aligned}$$

and the Lemma follows. $\square$

We shall now deduce estimates for $\partial _y {\mathcal {G}}_{m,\varepsilon }^\pm (y,y_0,z)$ when *y* is still near $y_0$ but *z* is away from the critical layer. To this end, we shall use the symmetry of the Green’s function and the following result.

#### Lemma 4.5

Let $y_0\in [0,1]$ and $0<\varepsilon \le \varepsilon _0$. For all $z\in [0,1]$ such that $m|z-y_0|\le 3\beta$ we have the following.

$$\begin{aligned} \Vert \partial _y\partial _z{\mathcal {G}}_{m,\varepsilon }^\pm (\cdot ,y_0,z)\Vert _{L^2_y(J_4^c)}^2 + \Vert \partial _z{\mathcal {G}}_{m,\varepsilon }^\pm (\cdot ,y_0,z)\Vert _{L^2_y(J_4^c)}^2 \lesssim m^{-2\mu }|z-y_0\pm i\varepsilon |^{-1-2\mu }. \end{aligned}$$

#### Proof

We assume without loss of generality that $y_0\le 1-\frac{4\beta }{m}$. For any $y>z$, we have that $g(y):=\partial _z{\mathcal {G}}_{m,\varepsilon }^+(y,y_0,z)$ solves

$$\begin{aligned} \left( {\Delta }_m +\beta ^2\frac{1}{(y-y_0+i\varepsilon )^2}\right) g=0, \end{aligned}$$

with $g(1)=0$. Multiplying the equation by $\overline{g}\eta ^2$ and integrating from $y_2=y_0+\frac{7\beta }{2m}>z$ to 1, we find that

$$\begin{aligned} \begin{aligned} 0&=\int _{y_2}^1 |\partial _y g|^2\eta ^2 + 2\partial _yh \overline{g}\partial _y\eta \eta +m^2|g|^2\eta ^2 -\beta ^2\frac{|g|^2\eta ^2}{(y-y_0+i\varepsilon )^2} \, \textrm{d}y \\&\ge \int _{y_2}^1 \frac{1}{2}|\partial _yg|^2\eta ^2 +\left( \frac{m^2}{2}\eta ^2-2(\partial _y\eta )^2\right) |g|^2\, \textrm{d}y, \end{aligned} \end{aligned}$$

where we have used Young’s inequality and $m|y-y_0|\ge 2\beta$, for all $y\ge y_2$. For

$$\begin{aligned} \eta (y)= {\left\{ \begin{array}{ll}\frac{2m}{\beta }(y-y_2),& y\in (y_2,y_2+\frac{\beta }{2m}),\\ 1,& y\in (y_2+\frac{\beta }{2m}, 1),\end{array}\right. }. \end{aligned}$$

we get

$$\begin{aligned} \frac{m^2}{\beta ^2}\int _{y_2}^{y_2+\frac{\beta }{2m}}|g|^2\textrm{d}y \ge \frac{1}{2} \int _{y_2+\frac{\beta }{2m}}^1 \left( |\partial _y g|^2 + m^2 |g|^2\right) \textrm{d}y. \end{aligned}$$

Using Proposition 4.1, we can estimate

$$\begin{aligned} \begin{aligned} \frac{m^2}{\beta ^2}\int _{y_2}^{y_2+\frac{\beta }{2m}}|g(y,y_0,z)|^2\textrm{d}y&\lesssim m^{-4\mu }|z-y_0+i\varepsilon |^{-1-2\mu }\frac{m^2}{\beta ^2}\int _{y_2}^{y_2+\frac{\beta }{2m}}|y-y_0+i\varepsilon |^{1-2\mu }\textrm{d}y\\&\lesssim m^{-2\mu }|z-y_0+i\varepsilon |^{-1-2\mu }. \end{aligned} \end{aligned}$$

Now, $y_2 +\frac{\beta }{2m}=y_0+\frac{4\beta }{m}$ so that

$$\begin{aligned} \int _{y_0+\frac{4\beta }{m}}^1 \left( |\partial _y g|^2 + m^2 |g|^2\right) \textrm{d}y \lesssim m^{-2\mu }|z-y_0+i\varepsilon |^{-1-2\mu }. \end{aligned}$$

The proof is finished. $\square$

The next corollary is a direct consequence of the above Lemma together with the observation that once the estimate for $\partial _z{\mathcal {G}}_{m,\varepsilon }^\pm (y,y_0,z)$ is established, the estimate of $\partial _y{\mathcal {G}}_{m,\varepsilon }^\pm (y,y_0,z)$ follows from the fact that, since ${\mathcal {G}}_{m,\varepsilon }^\pm (y,y_0,z)={\mathcal {G}}_{m,\varepsilon }^\pm (z,y_0,y)$, then $(\partial _y{\mathcal {G}}_{m,\varepsilon }^\pm )(y,y_0,z)=(\partial _z{\mathcal {G}}_{m,\varepsilon }^\pm )(z,y_0,y)$.

#### Corollary 4.6

Let $y_0\in [0,1]$ and $0<\varepsilon \le \varepsilon _0$. For all $y\in [0,1]$ such that $m|y-y_0|\le 3\beta$ we have that.

$$\begin{aligned} \Vert \partial _y{\mathcal {G}}_{m,\varepsilon }^\pm (y,y_0,\cdot )\Vert _{L^2_z(J_4^c)}\lesssim \frac{1}{m^{1+\mu }}|y-y_0\pm i\varepsilon |^{-\frac{1}{2}-\mu }. \end{aligned}$$

### Proof of Theorem 2

Let $0<\varepsilon \le {\varepsilon _0}\le \frac{\beta }{m}$ and assume that $m|y-y_0|\le 3\beta$. For $m\le 8\beta$, the Theorem follows directly from Proposition 4.1. Hence, we consider for $m>8\beta$ and we note that

$$\begin{aligned} \Vert {\mathcal {G}}_{m,\varepsilon }^\pm \Vert _{L^2_z}\le \Vert {\mathcal {G}}_{m,\varepsilon }^\pm \Vert _{L^2_z(J_3)} + \Vert {\mathcal {G}}_{m,\varepsilon }^\pm \Vert _{L^2_z(J_3^c)}, \quad \Vert \partial _y{\mathcal {G}}_{m,\varepsilon }^\pm \Vert _{L^2_z}\le \Vert \partial _y{\mathcal {G}}_{m,\varepsilon }^\pm \Vert _{L^2_z(J_4)} + \Vert \partial _y{\mathcal {G}}_{m,\varepsilon }^\pm \Vert _{L^2_z(J_4^c)}. \end{aligned}$$

Now, the bounds for $\Vert {\mathcal {G}}_{m,\varepsilon }^\pm \Vert _{L^2_z(J_3)}$ and $\Vert \partial _y{\mathcal {G}}_{m,\varepsilon }^\pm \Vert _{L^2_z(J_4)}$ follow from Proposition 4.1, while the estimate for $\Vert {\mathcal {G}}_{m,\varepsilon }^\pm \Vert _{L^2_z(J_3^c)}$ is given in Lemma 4.4 due to the *y*, *z* symmetry of the Green’s function and the estimate for $\Vert \partial _y{\mathcal {G}}_{m,\varepsilon }^\pm \Vert _{L^2_z(J_4^c)}$ is given by Corollary 4.6. The theorem follows.

## Bounds on the Green’s Function for $\beta ^2$ = 1/4

This section studies and obtains $L^2$ bounds on the Green’s function for the case $\beta ^2=1/4$. Most of the results and proof are analogous to the ones presented in Sect. 4 above, so we limit ourselves to present the statements we use and comment on the main ingredients of the proof.

### Theorem 5

There exists $\varepsilon _0>0$ such that for all $\varepsilon \in (0, \varepsilon _0)$ and for all $y,y_0\in [0,1]$ such that $m|y-y_0|\le 3\beta$, we have

$$\begin{aligned}  &   |y-y_0 \pm i\varepsilon |^{-\frac{1}{2}} \Vert {\mathcal {G}}^\pm _{m,\varepsilon }(y,y_0,\cdot )\Vert _{L^2_z}+ |y-y_0 \pm i\varepsilon |^\frac{1}{2} \Vert \partial _y{\mathcal {G}}^\pm _{m,\varepsilon }(y,y_0,\cdot )\Vert _{L^2_z}\\  &   \quad \lesssim \frac{1}{m}\left( 1 + \big | \log m\left| y-y_0 \pm i\varepsilon \right| \big |\right) \end{aligned}$$

In comparison with Theorem 2, we have a logarithmic correction to the behavior near the critical layer. The proof is omitted as it is analogous to that of Theorem 2, once all the intermediate steps are established. The rest of this section is devoted to the proof of such steps, to be compared with the analogous one of Sect. 4.

### Estimates near the critical layer

Using the analytic continuation properties from Lemma A.2, we can write the Wronskian as

$$\begin{aligned} \begin{aligned} {\mathcal {W}}_{m,\varepsilon }^+(y_0)&=\frac{2im}{\sqrt{\pi }}\big ( M_0(1-y_0+i\varepsilon )W_0(y_0-i\varepsilon )-W_0(1-y_0+i\varepsilon )M_0(y_0-i\varepsilon )\big ) \\&\quad + 2m M_0(1-y_0+i\varepsilon )M_0(y_0-i\varepsilon ). \end{aligned} \end{aligned}$$

We then have the following.

#### Proposition 5.1

Let $y,y_0,z\in [0,1]$ such that $m|y-y_0+i\varepsilon |\le 10\beta$ and $m|z-y_0+i\varepsilon |\le 10\beta$. There exists $\varepsilon _0>0$ such that

$$\begin{aligned}  &   |{\mathcal {G}}_{m,\varepsilon }^+(y,y_0,z)|\lesssim |y-y_0+i\varepsilon |^{\frac{1}{2}}|z-y_0+i\varepsilon |^{\frac{1}{2}}\left( 1 + \big |\log (m|y-y_0+i\varepsilon )|\big |\right) \\  &   \quad \left( 1 + \big |\log (m|z-y_0+i\varepsilon )|\big |\right) \end{aligned}$$

and

$$\begin{aligned}  &   |\partial _y{\mathcal {G}}_{m,\varepsilon }^+(y,y_0,z)|\lesssim |y-y_0+i\varepsilon |^{-\frac{1}{2}}|z-y_0+i\varepsilon |^{\frac{1}{2}}\left( 1 + \big |\log (m|y-y_0+i\varepsilon )|\big |\right) \\  &   \quad \left( 1 + \big |\log (m|z-y_0+i\varepsilon )|\big |\right) \end{aligned}$$

for all $$\varepsilon \le \varepsilon _0$$.

#### Proof

Assume without loss of generality that $y\le z$. From the asymptotic expansions given by Lemma A.3, we have that

$$\begin{aligned}  &   |\phi _{u,m,\varepsilon }^+(z,y_0)|\lesssim (2m|z-y_0+i\varepsilon |)^\frac{1}{2}\left( 1+ \log (2m|z-y_0+i\varepsilon )|\right) \\  &   \quad \big (|W_0(1-y_0+i\varepsilon )|+|M_0(1-y_0+i\varepsilon )|\big ), \end{aligned}$$

while

$$\begin{aligned}  &   |\phi _{l,m,\varepsilon }^+(y,y_0)|\lesssim (2m|y-y_0+i\varepsilon |)^\frac{1}{2}\left( 1+ \log (2m|y-y_0+i\varepsilon )|\right) \\  &   \quad \big (|W_0(y_0-i\varepsilon )|+|M_0(y_0-i\varepsilon )|\big ). \end{aligned}$$

The proposition follows from the estimates on the Wronskian given in the lemma below.

$$\square $$

#### Lemma 5.2

Let $y_0\in [0,1]$. There exists $0<\varepsilon _0\le \frac{\beta }{m}$ and $C>0$ such that

$$\begin{aligned} |{\mathcal {W}}_{m,\varepsilon }^+(y_0)|\ge C_\nu m|M_0(y_0-i\varepsilon )||M_0(1-y_0+i\varepsilon )|, \end{aligned}$$

for all $$\varepsilon \le \varepsilon _0$$.

#### Proof

The proof follows from treating the next two cases. Let $N_0>0$ be given as in Lemma A.10.

$\bullet$ **Case 1:** $m<N_0.$ Assume that $y_0\le \frac{1}{2}$. Then $2my_0< N_0$ and $m\le 2\,m(1-y_0)< 2N_0$. Assume further that $2my_0\le \delta _1$ given by Lemma A.11. Then,

$$\begin{aligned} {\mathcal {W}}_{m,\varepsilon }^+(y_0)= &   \frac{2mi}{\sqrt{\pi }}M_0(1-y_0+i\varepsilon )W_0(y_0-i\varepsilon )\\  &   \quad \times \left( 1-\frac{W_0(1-y_0+i\varepsilon )}{M_0(1-y_0+i\varepsilon )}\frac{M_0(y_0-i\varepsilon )}{W_0(y_0-i\varepsilon )} -i \sqrt{\pi }\frac{M_0(y_0-i\varepsilon )}{W_0(y_0-i\varepsilon )}\right) \end{aligned}$$

and from Lemma A.12 and Lemma A.11 we have

$$\begin{aligned} \left| \frac{W_0(1-y_0+i\varepsilon )}{M_0(1-y_0+i\varepsilon )} \right| \le C_0, \quad \left| \frac{M_0(y_0-i\varepsilon )}{W_0(y_0-i\varepsilon )} \right| \le \frac{1}{2(C_0+\sqrt{\pi })}, \end{aligned}$$

for all $\varepsilon \le \varepsilon _0$, from which the lower bounds on the Wronskian follows.

Similarly, assume now that $\delta _1<2my_0< N_0$, in this case we write

$$\begin{aligned} \begin{aligned} {\mathcal {W}}_{m,\varepsilon }^+(y_0)&=2m M_0(1-y_0+i\varepsilon )M_0(y_0-i\varepsilon )\\&\quad \times \left( 1 + \frac{i}{\sqrt{\pi }} \left( \frac{W_0(y_0-i\varepsilon )}{M_0(y_0-i\varepsilon )} - \frac{W_0(1-y_0+i\varepsilon )}{M_0(1-y_0+i\varepsilon )}\right) \right) \end{aligned} \end{aligned}$$

and we further note that, for all $\varepsilon \le \varepsilon _0$,

$$\begin{aligned} \begin{aligned}&\left| 1 + \frac{i}{\sqrt{\pi }}\left( \frac{W_0(y_0-i\varepsilon )}{M_0(y_0-i\varepsilon )} - \frac{W_0(1-y_0+i\varepsilon )}{M_0(1-y_0+i\varepsilon }\right) \right| \\&\qquad \ge 1 - \frac{1}{\sqrt{\pi }}\left( \left| \textrm{Im}\left( \frac{W_0(y_0-i\varepsilon )}{M_0(y_0-i\varepsilon )} \right) \right| + \left| \textrm{Im}\left( \frac{W_0(1-y_0+i\varepsilon )}{M_0(1-y_0+i\varepsilon }\right) \right| \right) \\&\qquad \ge \frac{1}{2}, \end{aligned} \end{aligned}$$

due to the estimates from Lemma A.12. The lower bound on the Wronskian follows as before.

$\bullet$ **Case 2:** $m\ge N_0.$ Under the assumption that $2m(1-y_0)\ge N_0$ and that $2m(y_0-i\varepsilon )\le \delta _1$ we can write

$$\begin{aligned} {\mathcal {W}}_{m,\varepsilon }^+(y_0)= &   \frac{2mi}{\sqrt{\pi }}M_0(1-y_0+i\varepsilon )W_0(y_0-i\varepsilon )\\  &   \quad \times \left( 1-\frac{W_0(1-y_0+i\varepsilon )}{M_0(1-y_0+i\varepsilon )}\frac{M_0(y_0-i\varepsilon )}{W_0(y_0-i\varepsilon )} -i \sqrt{\pi }\frac{M_0(y_0-i\varepsilon )}{W_0(y_0-i\varepsilon )}\right) \end{aligned}$$

and we have that

$$\begin{aligned} \left| \frac{W_0(1-y_0+i\varepsilon )}{M_0(1-y_0+i\varepsilon )} \right| \le \sqrt{\pi }, \quad \left| \frac{M_0(y_0-i\varepsilon )}{W_0(y_0-i\varepsilon )} \right| \le \frac{1}{4\sqrt{\pi }}, \end{aligned}$$

from which we obtain the lower bound

$$\begin{aligned} |{\mathcal {W}}_{m,\varepsilon }^+(y_0)|\ge \frac{4m}{\sqrt{\pi }}|M_0(1-y_0+i\varepsilon )||W_0(y_0-i\varepsilon )|. \end{aligned}$$

Now, when $2my_0\ge \delta _1$, we write

$$\begin{aligned} \begin{aligned} {\mathcal {W}}_{m,\varepsilon }^+(y_0)&=2m M_0(1-y_0+i\varepsilon )M_0(y_0-i\varepsilon )\\&\quad \times \left( 1 + \frac{i}{\sqrt{\pi }} \left( \frac{W_0(y_0-i\varepsilon )}{M_0(y_0-i\varepsilon )} - \frac{W_0(1-y_0+i\varepsilon )}{M_0(1-y_0+i\varepsilon )}\right) \right) \end{aligned} \end{aligned}$$

and we further note that, for all $\varepsilon \le \varepsilon _0$,

$$\begin{aligned} \begin{aligned}&\left| 1 + \frac{i}{\sqrt{\pi }}\left( \frac{W_0(y_0-i\varepsilon )}{M_0(y_0-i\varepsilon )} - \frac{W_0(1-y_0+i\varepsilon )}{M_0(1-y_0+i\varepsilon }\right) \right| \\&\qquad \qquad \ge 1 - \frac{1}{\sqrt{\pi }}\left( \left| \textrm{Im}\left( \frac{W_0(y_0-i\varepsilon )}{M_0(y_0-i\varepsilon )} \right) \right| + \left| \frac{W_0(1-y_0+i\varepsilon )}{M_0(1-y_0+i\varepsilon }\right| \right) \\&\qquad \qquad \ge \frac{1}{2}, \end{aligned} \end{aligned}$$

due to the estimates from Lemma A.12 and Lemma A.10. $\square$

### Estimates for ${\mathcal {G}}_{m,\varepsilon }$ away from the critical layer

Throughout this section, let $\varepsilon _0$ be given by Lemma 5.2 and let $m>8\beta$.

#### Lemma 5.3

Let $y_0\in [0,1]$ and $0<\varepsilon \le \varepsilon _0$. For all $z\in [0,1]$ such that $m|z-y_0|\le 9\beta$ we have

$$\begin{aligned}  &   \Vert \partial _y{\mathcal {G}}_{m,\varepsilon }^\pm (\cdot ,y_0,z)\Vert _{L^2_y(J_3^c)}^2 + m^2\Vert {\mathcal {G}}_{m,\varepsilon }^\pm (\cdot ,y_0,z)\Vert _{L^2_y(J_3^c)}^2 \\  &   \quad \lesssim |z-y_0\pm i\varepsilon |\left( 1 + \big | \log (m|z-y_0 \pm i\varepsilon |)\big |\right) ^2. \end{aligned}$$

#### Proof

We comment the case $y_0<1-\frac{3\beta }{m}$. The proof goes on the same spirit as the one for Lemma 4.4. For $y_2=y_0+\frac{2\beta }{m}$ and $h(y)={\mathcal {G}}_{m,\varepsilon }^+(y,y_0,z)$, introducing a suitable cut-off function we have that

$$\begin{aligned} |h(z)|+\frac{m^2}{\beta ^2}\int _{y_2}^{y_2+\frac{\beta }{m}}|h|^2\textrm{d}y \ge \frac{1}{2} \int _{y_2+\frac{\beta }{m}}^1 \left( |\partial _y h|^2 + m^2 |h|^2\right) \textrm{d}y. \end{aligned}$$

Using Proposition 5.1, we estimate

$$\begin{aligned} \begin{aligned}&|h(z)| + \frac{m^2}{\beta ^2}\int _{y_2}^{y_2+\frac{\beta }{m}}|h(y,y_0,z)|^2\textrm{d}y \\&\quad \lesssim \frac{m^2}{\beta ^2}|z-y_0+i\varepsilon |\left( 1 + \big | \log (m|z-y_0+i\varepsilon |)\big |\right) ^2\\&\qquad \int _{y_2}^{y_2+\frac{\beta }{m}}|y-y_0+i\varepsilon |\left( 1 + \big | \log (m|y-y_0+i\varepsilon |)\big |\right) ^2\textrm{d}y \\&\qquad +|z-y_0+i\varepsilon |\left( 1 + \big | \log (m|z-y_0+i\varepsilon |)\big |\right) ^2 \\&\quad \lesssim |z-y_0+i\varepsilon |\left( 1 + \big | \log (m|z-y_0+i\varepsilon |)\big |\right) ^2, \end{aligned} \end{aligned}$$

since $1\le m|y-y_0+i\varepsilon |\le 2$, for all $y\in \left[ y_2, y_2+\frac{\beta }{m}\right]$. Therefore, recalling $y_2=y_0+\frac{2\beta }{m}$ we have the bound

$$\begin{aligned} \int _{y_0+\frac{3\beta }{m}}^1 \left( |\partial _y h|^2 + m^2 |h|^2\right) \textrm{d}y \lesssim |z-y_0+i\varepsilon |\left( 1 + \big | \log (m|z-y_0+i\varepsilon |)\big |\right) ^2 \end{aligned}$$

and the proof follows. $\square$

We next provide an intermediate result towards estimates for $\Vert \partial _y{\mathcal {G}}_{m,\varepsilon }^\pm (y,y_0,\cdot )\Vert _{L^2_z(J_4^c)}$.

#### Lemma 5.4

Let $y_0\in [0,1]$ and $0<\varepsilon \le \varepsilon _0$. For all $z\in [0,1]$ such that $m|z-y_0|\le 3\beta$ we have

$$\begin{aligned}  &   \Vert \partial _y\partial _z{\mathcal {G}}_{m,\varepsilon }^\pm (\cdot ,y_0,z)\Vert _{L^2_y(J_4^c)}^2 + \Vert \partial _z{\mathcal {G}}_{m,\varepsilon }^\pm (\cdot ,y_0,z)\Vert _{L^2_y(J_4^c)}^2 \\  &   \quad \lesssim |z-y_0\pm i\varepsilon |^{-1}\left( 1 + \big | \log (m|z-y_0+i\varepsilon |)\big |\right) ^2. \end{aligned}$$

#### Proof

From the proof of Lemma 4.5, for $g(y):=\partial _z{\mathcal {G}}_{m,\varepsilon }^+(y,y_0,z)$ we have that

$$\begin{aligned} \frac{m^2}{\beta ^2}\int _{y_2}^{y_2+\frac{\beta }{2m}}|g|^2\textrm{d}y \ge \frac{1}{2} \int _{y_2+\frac{\beta }{2m}}^1 \left( |\partial _y g|^2 + m^2 |g|^2\right) \textrm{d}y. \end{aligned}$$

Using Proposition 5.1 we estimate

$$\begin{aligned} \begin{aligned} \frac{m^2}{\beta ^2}\int _{y_2}^{y_2+\frac{\beta }{2m}}|g(y,y_0,z)|^2\textrm{d}y&\lesssim |z-y_0+i\varepsilon |^{-1}\left( 1 + \big | \log (m|z-y_0+i\varepsilon |)\big |\right) ^2. \end{aligned} \end{aligned}$$

Therefore, since $y_2 +\frac{\beta }{2m}=y_0+\frac{4\beta }{m}$ we have the bound

$$\begin{aligned} \int _{y_0+\frac{4\beta }{m}}^1 \left( |\partial _y g|^2 + m^2 |g|^2\right) \textrm{d}y \lesssim |z-y_0+i\varepsilon |^{-1}\left( 1 + \big | \log (m|z-y_0+i\varepsilon )|\big |\right) ^2, \end{aligned}$$

and the lemma follows. $\square$

We finish the section with the estimates for $\Vert \partial _y{\mathcal {G}}_{m,\varepsilon }^\pm (y,y_0,\cdot )\Vert _{L^2_z(J_4^c)}$, which are deduce using the symmetry properties of the Green’s function as in Corollary 4.6 and are given in the next result.

#### Corollary 5.5

Let $y_0\in [0,1]$ and $0<\varepsilon \le \varepsilon _0$. For all $y\in [0,1]$ such that $m|y-y_0|\le 3\beta$ we have

$$\begin{aligned} \Vert \partial _y{\mathcal {G}}_{m,\varepsilon }^\pm (y,y_0,\cdot )\Vert _{L^2_z(J_4^c)}\lesssim \frac{1}{m}|y-y_0\pm i\varepsilon |^{-\frac{1}{2}}\left( 1 + \big | \log (m|y-y_0+i\varepsilon |)\big |\right) . \end{aligned}$$

$$\dagger $$

## Contour Integral Reduction

In this section, we study the contour integration that is present in the Dunford’s formula (see (2.2))

6.1 $$\begin{aligned} \begin{pmatrix} \psi _m(t,y) \\ \rho _m(t,y) \end{pmatrix} = \frac{1}{2\pi i} \int _{\partial {\Omega }}\textrm{e}^{-imct} \mathcal {R}(c,L_m)\begin{pmatrix} \psi _m^0 \\ \rho _m^0 \end{pmatrix} \,\textrm{d}c, \quad {\mathcal {R}(c,L_m):=(c-L_m)^{-1}},\nonumber \\ \end{aligned}$$

where ${\Omega }$ is any domain containing $\sigma (L_m)$, the spectrum of the linearized operator $L_m$ in (2.1). The main goal of this section is, under suitable conditions on the initial data, to reduce the above contour integration to a much simpler integration along the essential spectrum $\sigma _{ess}(L_m)=[0,1]$.

As the domain of integration we take the rectangle $\Omega = [-\beta /m,1+\beta /m]\times [-\beta /m,\beta /m]$, and further consider the inner rectangular region,

$$\begin{aligned} \begin{aligned} R_*:=\left\{ c=y_0 + is\in {{\mathbb {C}}}: y_0\in \left[ y_*,1-y_*\right] , \, s\in [-\varepsilon _*, \varepsilon _*] \right\} , \end{aligned} \end{aligned}$$

for some $y_*<0$ and $\varepsilon _*>0$ to be determined later on. Further, we decompose

$$\begin{aligned} \partial R_* = R_*^0 \cup R_*^{ess} \cup R_*^1, \end{aligned}$$

where

$$\begin{aligned} R_*^0&= \left( \lbrace y_* \rbrace \times [-\varepsilon _*,\varepsilon _*]\right) \cup \left( [y_*, 0 ]\times \lbrace \varepsilon _* \rbrace \right) \cup \left( [y_*, 0 ]\times \lbrace -\varepsilon _* \rbrace \right) , \\ R_*^{ess}&= \left( [ 0, 1 ]\times \lbrace \varepsilon _* \rbrace \right) \cup \left( [0, 1 ]\times \lbrace -\varepsilon _* \rbrace \right) , \\ R_*^1&= \left( \lbrace 1-y_* \rbrace \times [-\varepsilon _*,\varepsilon _*]\right) \cup \left( [1,1-y_*]\times \lbrace \varepsilon _* \rbrace \right) \cup \left( [1,1-y_*]\times \lbrace -\varepsilon _* \rbrace \right) . \end{aligned}$$

**Fig. 2:**
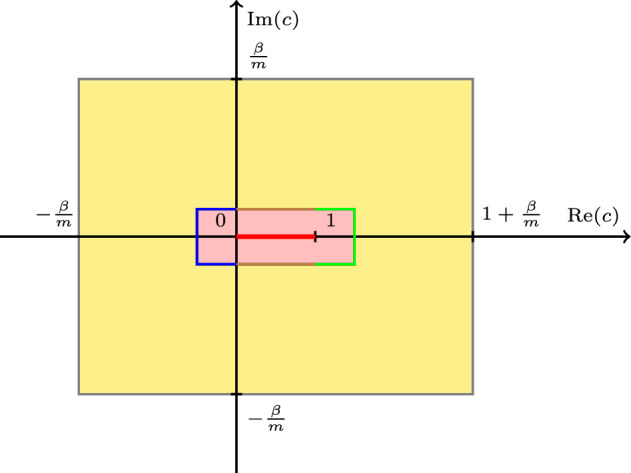
The domain of integration  and the smaller rectangular sub-domain , which is bounded by ,  and

The decomposition of $\Omega$ is depicted in Fig. 2 below.

The goal of the next three sections is to show the following result, which amounts to reduce our contour integration to $R_*^{ess}$, in the limit as $\varepsilon _*\rightarrow 0$, as in (2.3).

### Proposition 6.1

Assume that the pair of initial data $({\omega }_m^0, \rho _m^0)$ is orthogonal to the subspace generated by the eigenfunctions of $L_m$. Then,

$$\begin{aligned} \begin{aligned} \int _{\partial \Omega }\textrm{e}^{-imct}\mathcal {R}(c,L_m)\textrm{d}c&=\frac{1}{2\pi i }\lim _{\varepsilon \rightarrow 0}\int _0^1 \textrm{e}^{-imy_0t}\big ((-y_0-i\varepsilon +L_m)^{-1}\big . \\&\qquad \big .-(-y_0+i\varepsilon +L_m)^{-1}\big )\begin{pmatrix} \psi _m^0 \\ \rho _m^0 \end{pmatrix}\, \textrm{d}y_0. \end{aligned} \end{aligned}$$

The description of the spectrum in Theorem 3 will then be clear from the following three sections. As a first step towards proving Proposition 6.1 we show that $\sigma (L_m)\subset \Omega$.

### Lemma 6.2

Let $c\in {{\mathbb {C}}}{\setminus } \Omega$. Then, $\left( c - L_m\right) ^{-1}$ exists and

$$\begin{aligned} \left\| \left( c - L_m\right) ^{-1}\begin{pmatrix} \psi _m^0 \\ \rho _m^0 \end{pmatrix}\right\| _{L^2}\lesssim \Vert {\omega }_m^0\Vert _{L^2_y} + \Vert \rho _m^0\Vert _{L^2_y} \end{aligned}$$

### Proof

For any $c\in {{\mathbb {C}}}\setminus \Omega$, we note that $\tfrac{1}{|y-c|}\le \tfrac{m}{\beta }$, for all $y\in [0,1]$ and we define $\psi _m(y,c)$ as the unique solution to

$$\begin{aligned} {\Delta }_m\psi _{m}+\beta ^2\frac{\psi _{m}}{(y-c)^2}=\frac{{\omega }_m^0}{y-c}-\beta ^2\frac{\rho _m^0}{(y-c)^2}. \end{aligned}$$

with homogeneous boundary conditions $\psi _m(0,c)=\psi _m(1,c)=0$ given by standard ODE theory. We also define

$$\begin{aligned} \rho _{m}(y,c)=\frac{1}{y-c}\left( \rho _m^0(y)+\psi _{m}(y,c)\right) \end{aligned}$$

and it is straightforward to see that

6.2 $$\begin{aligned} ( -c+L_m)\begin{pmatrix} \psi _{m}(y,c) \\ \rho _{m}(y,c) \end{pmatrix}=\begin{pmatrix} \psi _m^0(y) \\ \rho _m^0(y) \end{pmatrix}. \end{aligned}$$

Therefore, $(-c+L_m)$ is invertible and the desired resolvent estimates follow from usual energy estimates on the equation, that is, multiply the equation by $\overline{\psi _m(y,c)}$ integrate by parts and absorb the potential term. $\square$

In order to show that the only contributions that remain are those given by (6.2), we study the resolvent operator for the cases $\beta ^2>1/4$, $\beta ^2=1/4$ and $\beta ^2<1/4$ separately.

### Integral reduction for $\beta ^2>1/4$: discrete eigenvalues

The classical Miles-Howard stability criterion [17, 25] rules out the existence of unstable modes when $\beta ^2\ge 1/4$. That is, any eigenvalue $c\in {{\mathbb {C}}}$ of $L_m$ must have $\textrm{Im}(c)=0$.

#### Discrete eigenvalues of $L_m$

In this subsection we find and characterize the discrete set of real isolated eigenvalues that accumulate towards the end-points of the essential spectrum, that is, towards 0 and 1. Our study involves a precise understanding of the Wronskian when $\varepsilon =0$. For this, we denote

6.3 $$\begin{aligned} {\mathcal {W}}_m(c):=M_+(c)M_-(1+c)-M_-(c)M_+(1+c) \end{aligned}$$

for all $c>0$ and we note from (3.6) that ${\mathcal {W}}_{m,0}^\pm (-c)=4i\nu m{\mathcal {W}}_m(c)$. We state the following.

##### Proposition 6.3

There exists sequences $\lbrace p_k\rbrace _{k\ge 1}$ and $\lbrace q_k\rbrace _{k\ge 1}$ of strictly positive real numbers such that $p_k,q_k\rightarrow 0$ as $k\rightarrow \infty$ and

$$\begin{aligned} |{\mathcal {W}}_m(p_k)|=2|M_+(p_k)||M_+(1+p_k)|, \quad {\mathcal {W}}_m(q_k)=0, \end{aligned}$$

for all $$k\ge 1$$.

##### Proof

For any $c>0$, from (6.3) we have that

$$\begin{aligned} {\mathcal {W}}_m(c)= -2i\textrm{Im}\big (M_-(c)M_+(1+c)\big ), \end{aligned}$$

where further $M_-(c)=\overline{M_+(c)}=|M_+(c)|\textrm{e}^{-i\text {Arg}(M_+(c))}$ and $M_+(1+c)=|M_+(1+c)|\textrm{e}^{i\text {Arg}(M_+(1+c))}$. For $x>0$, we define $\Theta (x)=\text {Arg}(M_+(x))$ and we write

$$\begin{aligned} {\mathcal {W}}_m(c)=-2i|M_+(c)||M_+(1+c)|\sin \left( \Theta (1+c)-\Theta (c)\right) . \end{aligned}$$

The proposition follows if we can find some integer $k_0\ge 0$ and two sequences $\lbrace p_k\rbrace _{k\ge 1}$ and $\lbrace q_k\rbrace _{k\ge 1}$ of strictly positive real numbers such that

$$\begin{aligned} \Theta (1+p_k) - \Theta (p_k) = \left( k+k_0+\frac{1}{2}\right) \pi , \qquad \Theta (1+q_k) - \Theta (q_k) = (k+k_0)\pi , \end{aligned}$$

for all $k\ge 1$. To this end, given the Wronskian properties of the pair $M_+(x)$ and $M_-(x)$ from [26], we note that for all $x>0$

$$\begin{aligned} \begin{aligned} -2i\nu&= M_+(x)M_-'(x) - M_+'(x)M_-(x) \\&= |M_+(x)|\textrm{e}^{i\Theta (x)}\left( |M_+(x)|'\textrm{e}^{-i\Theta (x)} -i\Theta '(x)|M_+(x)|\textrm{e}^{-i\Theta (x)}\right) \\&\quad - |M_+(x)|\textrm{e}^{-i\Theta (x)}\left( |M_+(x)|'\textrm{e}^{i\Theta (x)} + i\Theta '(x)|M_+(x)|\textrm{e}^{i\Theta (x)}\right) \\&=-2i\Theta '(x)|M_+(x)|^2, \end{aligned} \end{aligned}$$

and thus, $\Theta '(x)=\frac{\nu }{|M_+(x)|^2}>0$. Hence, for all $c>0$ we define

$$\begin{aligned} r(c):=\Theta (1+c)-\Theta (c) = \nu \int _c^{1+c}\frac{1}{|M_+(x)|^2}\textrm{d}x. \end{aligned}$$

Note that *r*(*c*) is continuous for all $c>0$ and strictly decreasing. This follows from $|M_+(x)|$ being strictly increasing, see Lemma A.5. Moreover, also from Lemma A.5, we have that

$$\begin{aligned} r(c)\gtrsim _\nu \int _c^{1+c} \frac{1}{x}\textrm{d}x \gtrsim _\nu \ln \left( \frac{1+c}{c}\right) , \end{aligned}$$

which diverges as $c\rightarrow 0$, while from Lemma A.6 and since $|M_+(x)|$ is an increasing function of $x\ge 0$, we have

$$\begin{aligned} r(c)\le \frac{\nu }{|M_+(c)|^2}\lesssim \textrm{e}^{-c} \end{aligned}$$

for *c* sufficiently large. Therefore, $r(c):(0,+\infty )\rightarrow (0,+\infty )$ is a bijection and we conclude the existence of two sequences of strictly positive real numbers $\lbrace p_k\rbrace _{k\ge 1}$ and $\lbrace q_k\rbrace _{k\ge 1}$ such that $q_{k+1}<p_k<q_{k}$, for all $k\ge 1$ and

$$\begin{aligned} r(p_k)=\left( k+\frac{1}{2}\right) \pi , \qquad r(q_k) = k\pi , \end{aligned}$$

with the further property that $p_k,q_k\rightarrow 0$ as $k\rightarrow \infty$. $\square$

##### Corollary 6.4

There are infinitely many eigenvalues $c_k:=-q_k<0$ and $d_k:=1-c_k>1$ of $L_m$ associated to the eigenfunctions

$$\begin{aligned} \begin{pmatrix} \omega _{c_k} \\ \rho _{c_k} \end{pmatrix} = \begin{pmatrix} -\beta ^2\frac{\phi _{c_k}}{(y-c_k)^2} \\ \frac{\phi _{c_k}}{y-c_k} \end{pmatrix} \quad \text { and }\quad \begin{pmatrix} \omega _{d_k} \\ \rho _{d_k} \end{pmatrix} = \begin{pmatrix} -\beta ^2\frac{\phi _{d_k}}{(y-d_k)^2} \\ \frac{\phi _{d_k}}{y-d_k} \end{pmatrix}, \end{aligned}$$

where

$$\begin{aligned} \phi _{c_k}(y)=M_+(1-c_k)M_-(y-c_k) - M_-(1-c_k)M_+(y-c_k) \end{aligned}$$

and

$$\begin{aligned} \phi _{d_k}(y)=M_+(d_k)M_-(d_k-y) - M_-(d_k)M_+(d_k-y), \end{aligned}$$

respectively. Moreover, there exists some $C_0>0$ such that

$$\begin{aligned} |c_k| \lesssim \frac{1}{\textrm{e}^{C_0k}-1}, \end{aligned}$$

for all $$k\ge 1$$.

##### Proof

Any eigenvalue $c<0$ of $L_m$ is such that there exists a non-trivial solution $\phi _c(y)$ to

$$\begin{aligned} {\Delta }_m\phi _c + \beta ^2\frac{\phi _c}{(y-c)^2}=0 \end{aligned}$$

satisfying the boundary conditions $\phi _c(0)=\phi _c(1)=0$. We can write such solution as

$$\begin{aligned} \phi _c(y)=AM_+(y-c) + BM_-(y-c) \end{aligned}$$

and since $c<0$, it is smooth. Imposing the boundary conditions, we have non-trivial coefficients $A,B\in {{\mathbb {C}}}$ if and only if ${\mathcal {W}}_m(-c)=M_+(-c)M_-(1-c)-M_-(-c)M_+(1-c)$ vanishes. This is the case for the sequence $\lbrace q_k\rbrace _{k\in {{\mathbb {N}}}}$. These $c_k:=-q_k$ are the discrete eigenvalues of $L_m$ and from Proposition 6.3 and Lemma A.5, there exist some $C=C(\nu ,m)>0$ such that

$$\begin{aligned} k\pi = r(q_k)=\nu \int _{q_k}^{1+q_k}\frac{1}{|M_+(x)|^2}\textrm{d}x \le C\int _{q_k}^{1+q_k}\frac{1}{x}\textrm{d}x =C \ln \left( \frac{1+q_k}{q_k}\right) , \end{aligned}$$

from which the estimate on $c_k$ follows. Similarly, any $d>1$ is an eigenvalue of $L_m$ if there exists a non-zero solution $\phi _d(y)$ to

$$\begin{aligned} {\Delta }_m\phi _d + \beta ^2\frac{\phi _d}{(d-y)^2}=0 \end{aligned}$$

such that $\phi _d(0)=\phi _d(1)=0$ As before, the candidate solution is $\phi _d(y)=AM_+(d-y) + BM_-(d-y)$ and the homogeneous boundary conditions are non-trivially satisfied provided the Wronskian vanishes. Now, letting $d_k=1-c_k$ we see that

$$\begin{aligned} \mathcal {W}_m(d_k)&= M_+(d_k)M_-(d_k-1)-M_-(d_k)M_+(d_k-1) \\&= M_+(1-c_k)M_-(-c_k)-M_-(1-c_k)M_+(-c_k) \\&= 0. \end{aligned}$$

Thus, $\phi _{d_k}(y)=M_+(d_k)M_-(d_k-y)-M_-(d_k)M_+(d_k-y)$ is a non-zero solution to the equation and $d_k$ is an eigenvalue of $L_m$. $\square$

#### Contour Integrals of the Resolvent Operator

We shall next obtain suitable estimates on the contour integral of the resolvent. In this direction, we write

$$\begin{aligned} \int _{\partial \Omega }\textrm{e}^{-imct}\mathcal {R}(c,L_m)\textrm{d}c = \int _{\partial R_*}\textrm{e}^{-imct}\mathcal {R}(c,L_m)\textrm{d}c + \int _{\partial (\Omega \setminus R_*)}\textrm{e}^{-imct}\mathcal {R}(c,L_m)\textrm{d}c, \end{aligned}$$

where we recall $R_*=\left\{ c=y_0 + is\in {{\mathbb {C}}}: y_0\in \left[ y_*, 1-y_*\right] , \, s\in [-\varepsilon _*, \varepsilon _*] \right\}$, for some $y_*<0$ and $\varepsilon _*>0$ that will be determined later. Exploiting the decomposition $\partial R_* = R_*^0\cup R_*^{ess} \cup R_*^1$, we shall the that the contributions of both $R_*^0$ and $R_*^1$ can be made arbitrarily small. We shall prove this for $R_*^0$, since the arguments and computations for $R_*^1$ are the same. Now, we recall (2.4) and we the note that for $R_*^0$, we can write

$$\begin{aligned} \begin{aligned}&\left\| \int _{ R_*^0}\textrm{e}^{-imct}\mathcal {R}(c,L_m)\textrm{d}c \right\| _{L^2_y}\\&\quad \le \left\| \int _{y_*}^0 \textrm{e}^{-imy_0t}\left( \textrm{e}^{m\varepsilon _*t}\psi _{m,\varepsilon _*}^-(y,y_0)-\textrm{e}^{-m\varepsilon _*t}\psi _{m,\varepsilon _*}^+(y,y_0) \right) \textrm{d}y_0\right\| _{L^2_y} \\&\qquad + \left\| \int _0^{\varepsilon _*} \textrm{e}^{-imy_*t}\left( \textrm{e}^{mst}\psi _{m,s}^-(y,y_*)+\textrm{e}^{-mst}\psi _{m,s}^+(y,y_*) \right) \textrm{d}s\right\| _{L^2_y} \\&\qquad + \left\| \int _0^{\varepsilon _*} \left( \textrm{e}^{mst}\psi _{m,s}^-(y,0)+\textrm{e}^{-mst}\psi _{m,s}^+(y,0) \right) \textrm{d}s\right\| _{L^2_y} \\&\qquad +\left\| \int _{y_*}^0 \textrm{e}^{-imy_0t}\left( \textrm{e}^{m\varepsilon _*t}\rho _{m,\varepsilon _*}^-(y,y_0)-\textrm{e}^{-m\varepsilon _*t}\rho _{m,\varepsilon _*}^+(y,y_0) \right) \textrm{d}y_0\right\| _{L^2_y} \\&\qquad + \left\| \int _0^{\varepsilon _*} \textrm{e}^{-imy_*t}\left( \textrm{e}^{mst}\rho _{m,s}^-(y,y_*)+\textrm{e}^{-mst}\rho _{m,s}^+(y,y_*) \right) \textrm{d}s\right\| _{L^2_y} \\&\qquad + \left\| \int _0^{\varepsilon _*} \left( \textrm{e}^{mst}\rho _{m,s}^-(y,0)+\textrm{e}^{-mst}\rho _{m,s}^+(y,0) \right) \textrm{d}s\right\| _{L^2_y}. \end{aligned} \end{aligned}$$

We remark here that this decomposition is valid and will also be used for the cases $\beta ^2 < \frac{1}{4}$ and $\beta ^2=\frac{1}{4}$. In what follows, we obtain suitable estimates for each integral. We begin by obtaining bounds on the Green’s functions ${\mathcal {G}}_{m,\varepsilon }^\pm (y,y_*,z)$ when $y_*=-p_k$ for some $k\ge 0$ small and for $\varepsilon =\varepsilon _*$ small.

##### Lemma 6.5

Let $\varepsilon >0$ and $p_k>0$ given by Proposition 6.3 for some $k\ge 1$. Then,

$$\begin{aligned} |{\mathcal {G}}_{m,\varepsilon }^\pm (y,-p_k,z)|\lesssim _m |y+p_k -i\varepsilon |^\frac{1}{2}, \end{aligned}$$

uniformly for all $y,z\in [0,1]$, for $\frac{\varepsilon }{p_k}$ sufficiently small.

##### Proof

We proceed similarly as in the proof of Lemma 6.15. That is, for $y\le z$,

$$\begin{aligned} {\mathcal {G}}_{m,\varepsilon }^-(y,-p_k,z)=\frac{\phi _{l,m,\varepsilon }^-(y,-p_k)\phi _{u,m,\varepsilon }^-(z,-p_k)}{{{\mathcal {W}}_{m,\varepsilon }^-(-p_k)}} \end{aligned}$$

Due to the explicit solutions of the Taylor–Goldstein equation, we can find that

$$\begin{aligned} \begin{aligned}&{\phi _{l,m,\varepsilon }^-(y,-p_k)}\\&\quad = M_+(p_k- i \varepsilon )M_-(y+p_k- i\varepsilon )-M_-(p_k- i\varepsilon )M_+(y+p_k- i\varepsilon )\\&\quad = M_-(p_k)M_-(y+p_k-i\varepsilon ) -M_-(p_k)M_+(y+p_k-i\varepsilon ) + R_1(p_k,\varepsilon )\\ \end{aligned} \end{aligned}$$

where $R_1(p_k,\varepsilon )\lesssim \frac{\varepsilon }{p_k}|M_+(p_k)|\big ( |M_+(y+p_k-i\varepsilon )| + |M_-(y+p_k-i\varepsilon )|\big )$. In particular,

$$\begin{aligned} |\phi _{l,m,\varepsilon }^-(y,-p_k)| \lesssim \left( 1+\frac{\varepsilon }{p_k}\right) |M_+(p_k)||y+p_k-i\varepsilon |^\frac{1}{2}. \end{aligned}$$

On the other hand,

$$\begin{aligned} \begin{aligned}&\phi _{u,m,\varepsilon }^-(z,-p_k)=M_+(1+p_k)M_-(z+p_k)-M_-(1+p_k)M_+(z+p_k) + R_2(p_k,\varepsilon ) \\&=2i\textrm{Im}\left( M_+(1+p_k)M_-(z+p_k)\right) + R_2(\varepsilon ) \end{aligned} \end{aligned}$$

where $|R_2(\varepsilon )|\lesssim |M_+(1+p_k)|{\frac{\varepsilon }{p_k}}$. In particular,

$$\begin{aligned} |\phi _{u,\varepsilon }^-(z,-p_k)|\lesssim \left( 1+\frac{\varepsilon }{p_k}\right) |M_+(1+p_k)|. \end{aligned}$$

Now, let us now estimate the Wronskian. We trivially have that

$$\begin{aligned} \begin{aligned} {\mathcal {W}}_{m,\varepsilon }^-(-p_k)&= 4i\nu m \Big ( M_+(p_k-i\varepsilon )M_-(1+p_k-i\varepsilon ) - M_+(p_k-i\varepsilon )M_-(1+p_k-i\varepsilon )\Big ) \\&=4i\nu m \Big ( M_+(p_k)M_-(1+p_k) - M_-(p_k)M_+(1+p_k)\Big ) + R_3(p_k,\varepsilon ) \\&=8\nu m \textrm{Im}\Big (M_-(p_k)M_+(1+p_k)\Big ) + R_3(p_k,\varepsilon ) \\&=8\nu m (-1)^k|M_+(p_k)||M_-(1+p_k)|+ R_3(p_k,\varepsilon ), \end{aligned} \end{aligned}$$

where $|R_3(p_k,\varepsilon )|\lesssim \nu m|M_+(p_k)||M_+(1+p_k)|\frac{\varepsilon }{p_k}$. In particular,

$$\begin{aligned} \begin{aligned} |{\mathcal {W}}_{m,\varepsilon }(0)| \ge 8\nu m|M_+(p_k)||M_+(1+p_k)| - |R_3(p_k,\varepsilon )|\gtrsim \nu m|M_+(p_k)||M_+(1+p_k)|, \end{aligned} \end{aligned}$$

for $\frac{\varepsilon }{p_k}$ small enough. The bound on ${\mathcal {G}}_{m,\varepsilon }^-(y,-p_k,z)$ follows directly. $\square$

Once we have the pointwise bounds on the Green’s function, we are able to prove the following.

##### Proposition 6.6

Let $y_*=-p_k$ be given by Proposition 6.3 for some $k\ge 0$ and let $\varepsilon _*>0$ such that $\frac{\varepsilon _*}{|y_*|}$ is small enough. Then,

$$\begin{aligned} \left\| \int _0^{\varepsilon _*} \textrm{e}^{-imp_kt}\left( \textrm{e}^{mst}\psi _{m,s}^-(y,y_*)+\textrm{e}^{-mst}\psi _{m,s}^+(y,y_*) \right) \textrm{d}s\right\| _{L^2_y}\lesssim \varepsilon _* \end{aligned}$$

and

$$\begin{aligned} \left\| \int _0^{\varepsilon _*} \textrm{e}^{-imp_kt}\left( \textrm{e}^{mst}\rho _{m,s}^-(y,y_*)+\textrm{e}^{-mst}\rho _{m,s}^+(y,y_*) \right) \textrm{d}s\right\| _{L^2_y}\lesssim \varepsilon _*^\frac{1}{2}. \end{aligned}$$

##### Proof

Firstly, Minkowski inequality provides

$$\begin{aligned} \begin{aligned}&\left\| \int _0^{\varepsilon _*} \textrm{e}^{-imp_kt}\left( \textrm{e}^{mst}\psi _{m,s}^-(y,y_*)+\textrm{e}^{-mst}\psi _{m,s}^+(y,y_*) \right) \textrm{d}s\right\| _{L^2_y} \\&\quad \lesssim \sum _{\kappa \in \lbrace +, -\rbrace } \int _0^{\varepsilon _*} \Vert \psi _{m,s}^\kappa (y,-p_k)\Vert _{L^2_y}\textrm{d}s \\ \end{aligned} \end{aligned}$$

and we have that

$$\begin{aligned} \begin{aligned} \psi ^\pm _{m,s}(y,y_0)= \frac{1}{\beta ^2}(y-y_0\pm i\varepsilon ){\omega }_m^0(y) -\rho _m^0(y) + \int _0^1 {\mathcal {G}}^\pm _{m,\varepsilon }(y,y_0,z) F_{m,\varepsilon }^\pm (z,y_0) \textrm{d}z. \end{aligned} \end{aligned}$$

Using Cauchy-Schwarz and the uniform estimates from Lemma 6.5, we bound

$$\begin{aligned} \begin{aligned} \sum _{\sigma \in \lbrace +,-\rbrace }\Vert \psi _{m,s}^\sigma (y,-p_k)\Vert _{L^2_y}&\lesssim \Vert {\omega }_m^0\Vert _{L^2_y} + \Vert \rho _m^0\Vert _{L^2_y} +\sum _{\sigma \in \lbrace +,-\rbrace }\Vert {\mathcal {G}}_{m,s}^\sigma (y,-p_k,z)\Vert _{L^2_yL^2_z} \lesssim _m 1 \end{aligned} \end{aligned}$$

and thus integrating in *s* from 0 to $\varepsilon _*$ we get the first part of the Proposition. For the perturbed density, we recall that

$$\begin{aligned} \rho _{m,s}^\pm (y,y_0) = \frac{1}{\beta ^2}{\omega }_m^0(y)+\frac{1}{y-y_0\pm is}\int _0^1{\mathcal {G}}_{m,s}^\pm (y,y_0,z)F_{m,s}^\pm (z,y_0)\textrm{d}z. \end{aligned}$$

In particular, from Lemma 6.5 we have that

$$\begin{aligned} \begin{aligned} |\rho _{m,s}^\pm (y,-p_k)| \lesssim |{\omega }_m(y)| + |y+p_k-is|^{-\frac{1}{2}}\Vert F_{m,s}^\pm (z,y_0)\Vert _{L^2_z}. \end{aligned} \end{aligned}$$

For $\Vert F_{m,s}^\pm (z,y_0)\Vert _{L^2_z}\lesssim 1$ uniformly in $s\in (0,\varepsilon _*)$, we integrate in *s* from 0 to $\varepsilon _*$ to get the desired result. $\square$

We next obtain bounds on the Green’s function when the spectral parameter has non-zero imaginary part. These bounds are shown to depend both on the modulus and on the argument of the complex spectral parameter.

##### Lemma 6.7

Let $y_0<0$ and $\varepsilon >0$. Denote $c=-y_0+i\varepsilon =r\textrm{e}^{i\theta }$, with $r>0$ and $\theta \in \left( 0,\frac{\pi }{2}\right)$. Then,

$$\begin{aligned} |{\mathcal {G}}_{m,\varepsilon }^\pm (y,y_0,z)|\lesssim \frac{|y-y_0\pm i\varepsilon |^\frac{1}{2}}{\sinh ^2(\nu \theta )} \end{aligned}$$

and there exists $K_c>0$ such that

$$\begin{aligned} |{\mathcal {G}}_{m,\varepsilon }^-(y,y_0,z)-{\mathcal {G}}_{m,\varepsilon }^+(y,y_0,z)-K_c\phi _{u,m}(y)\phi _{u,m}(z)|\lesssim \frac{r^\frac{1}{2}}{\sinh ^2(\nu \theta )}, \end{aligned}$$

uniformly for all $y,z\in [0,1]$.

##### Proof

For $y_0<0$ and $\varepsilon >0$, we consider $c=-y_0+i\varepsilon =r\textrm{e}^{i\theta }$, with $r>0$ and $\theta \in \left( 0,\frac{\pi }{2}\right)$. We next study ${\mathcal {G}}_{m,\varepsilon }^+(y,y_0,z)$. For $y\le z$, we write

$$\begin{aligned} {\mathcal {G}}_{m,\varepsilon }^+(y,y_0,z)=\frac{\phi _{l,m,\varepsilon }^+(y,y_0)\phi _{u,m,\varepsilon }^+(z,y_0)}{{\mathcal {W}}_{m,\varepsilon }^+(y_0)} = \frac{\phi _{l,m,\varepsilon }^+(y,y_0)\phi _{u,m,\varepsilon }^+(y,y_0){\mathcal {W}}_{m,\varepsilon }^-(y_0)}{|{\mathcal {W}}_{m,\varepsilon }^+(y_0)|^2} \end{aligned}$$

The main difference with respect to the other estimates we have been carrying out is that now, we control $|{\mathcal {W}}_{m,\varepsilon }^+(y_0)|^2$ as follows:

$$\begin{aligned} \begin{aligned} {\mathcal {W}}_{m,\varepsilon }^+(y_0)&=4i\nu m\big ( M_+(c)M_-(1+c)-M_-(c)M_+(1+c)\big ) \\&=4i\nu m \big (M_+(c)M_-(1)-M_-(c)M_+(1)\big ) + R_1(c), \end{aligned} \end{aligned}$$

with $|R_1(c)|\lesssim r|M_+(c)||M_+(1)|$. For $c=r\textrm{e}^{i\theta }$, a detailed asymptotic analysis of $M_+(c)$ and $M_-(c)$ shows that

$$\begin{aligned} M_+(c)=r^\frac{1}{2}\textrm{e}^{-\nu \theta }\textrm{e}^{i\frac{\theta }{2}}r^{i\nu } + R_2(c), \quad M_-(c)=r^\frac{1}{2}\textrm{e}^{\nu \theta }\textrm{e}^{i\frac{\theta }{2}}r^{-i\nu } + R_3(c), \end{aligned}$$

where $$|R_2(c)|,\,|R_3(c)|\lesssim r^{\frac{5}{2}}$$. Hence,

$$\begin{aligned} {\mathcal {W}}_{m,\varepsilon }^+(y_0) = 4i\nu m r^\frac{1}{2} \textrm{e}^{i\frac{\theta }{2}}\big ( \textrm{e}^{-\nu \theta }r^{i\nu }M_-(1) - \textrm{e}^{\nu \theta }r^{-i\nu }M_+(1)\big ) + R_4(c), \end{aligned}$$

with $|R_4(c)|\le C_4r^{\frac{3}{2}}|M_+(1)|$. In particular, for $r\le \frac{4\nu m\sinh (\nu \theta )}{C_4}$ small enough, we estimate

$$\begin{aligned} |{\mathcal {W}}_{m,\varepsilon }^+(y_0)|\ge 4\nu m r^\frac{1}{2}|M_+(1)|\sinh (\nu \theta ). \end{aligned}$$

As expected, the bound degenerates as $\theta \rightarrow 0^+$. With this lower bound we are able to prove the first part of the proposition, using the asymptotic expansions of $M_+(y-y_0+i\varepsilon )$ and $M_-(y-y_0+i\varepsilon )$, see Lemma A.3. Nevertheless, to obtain the second part of the proposition, we continue by estimating

$$\begin{aligned} \begin{aligned} \phi _{u,m,\varepsilon }^+(z,y_0)&=M_+(1)M_-(z)-M_-(1)M_+(z) + R_5(c)=2i\textrm{Im}\left( M_+(1)M_-(z)\right) + R_5(c), \end{aligned} \end{aligned}$$

where $|R_5(c)|\lesssim r^\frac{1}{2}|M_+(1)|$. Similarly, we have

$$\begin{aligned} \begin{aligned} {\phi _{l,m,\varepsilon }^+(y,0)}{{{\mathcal {W}}_{m,\varepsilon }^-(y_0)}}&= 4i\nu m\Big ( M_+(c)M_-(y)M_+(\overline{c})M_-(1) -M_+(c)M_-(y)M_-(\overline{c})M_+(1)\Big . \\&\quad - \Big . M_-(c)M_+(y)M_+(\overline{c})M_-(1) + M_-(c)M_+(y)M_-(\overline{c})M_+(1)\Big ) + R_6(c), \end{aligned} \end{aligned}$$

with $|R_6(c)|\lesssim r^\frac{1}{2}|M_+(c)|^2|M_+(1)|$. In fact, we can recognize

$$\begin{aligned} \begin{aligned} {\phi _{l,m,\varepsilon }^+(y,0)}{{{\mathcal {W}}_{m,\varepsilon }^-}(y_0)}&= 4i\nu m\Big ( 2\textrm{Re}\big (M_+(c)M_-(y)M_+(\overline{c})M_-(1)\big )\Big . \\&\qquad -\Big .|M_+(c)|^2M_-(y)M_+(1) -|M_-(c)|^2M_+(y)M_-(1) \Big ) + R_6(c). \end{aligned} \end{aligned}$$

Hence, we obtain

$$\begin{aligned} \begin{aligned}&\phi _{l,m,\varepsilon }^+(y,y_0)\phi _{u,m,\varepsilon }^+(y,y_0){\mathcal {W}}_{m,\varepsilon }^-(y_0) \\&\quad = -8\nu m \textrm{Im}\left( M_+(1)M_-(z)\right) \Big ( 2\textrm{Re}\big (M_+(c)M_-(y)M_+(\overline{c})M_-(1)\big )\Big . \\&\qquad -\Big .|M_+(c)|^2M_-(y)M_+(1) -|M_-(c)|^2M_+(y)M_-(1) \Big ) + R_7(c), \end{aligned} \end{aligned}$$

where now $|R_7(c)|\lesssim r^\frac{1}{2}|M_+(c)|^2|M_+(1)|^2$. In particular,

$$\begin{aligned} \textrm{Im}&\left( \phi _{l,m,\varepsilon }^+(y,y_0)\phi _{u,m,\varepsilon }^+(y,y_0){\mathcal {W}}_{m,\varepsilon }^-(y_0)\right) \\&= -2\nu m \phi _{u,m}(z)\phi _{u,m}(y)\left( |M_+(c)|^2-|M_-(c)|^2\right) + \textrm{Im}\left( R_7(c)\right) . \end{aligned}$$

and

$$\begin{aligned} \left| \phi _{l,m,\varepsilon }^+(y,y_0)\phi _{u,m,\varepsilon }^+(y,y_0){\mathcal {W}}_{m,\varepsilon }^-(y_0)\right| \lesssim |M_+(c)|^2|M_+(1)|^2. \end{aligned}$$

Together with the lower bound on the Wronskian, we conclude the proof. $\square$

With the above bounds, we are able to estimate the contribution of the integral along the horizontal boundary.

##### Proposition 6.8

For $y_*<0$ small enough, let $r_*\textrm{e}^{i\theta _*}=y_*+i\varepsilon _*$. We have that

$$\begin{aligned} \left\| \int _0^{y_*} \textrm{e}^{-imy_0t}\big (\textrm{e}^{m\varepsilon _*t}\psi _{m,\varepsilon _*}^-(y,y_0)-\textrm{e}^{-m\varepsilon _*t}\psi _{m,\varepsilon _*}^+(y,y_0) \big )\textrm{d}y_0\right\| _{L^2_y}\lesssim \frac{r_*^\frac{3}{2}}{\sinh ^2(\nu \theta _*)} \end{aligned}$$

and

$$\begin{aligned} \left\| \int _0^{y_*} \textrm{e}^{-imy_0t}\big (\textrm{e}^{m\varepsilon _*t}\rho _{m,\varepsilon _*}^-(y,y_0)-\textrm{e}^{-m\varepsilon _*t}\rho _{m,\varepsilon _*}^+(y,y_0) \big )\textrm{d}y_0\right\| _{L^2_y}\lesssim \frac{r_*^\frac{1}{2}}{\sinh ^2(\nu \theta _*)} \end{aligned}$$

##### Proof

Firstly, note that

$$\begin{aligned} \begin{aligned} \textrm{e}^{m\varepsilon _*t}\psi _{m,\varepsilon _*}^-(y,y_0)-\textrm{e}^{-m\varepsilon _*t}\psi _{m,\varepsilon _*}^+(y,y_0)&= \textrm{e}^{m\varepsilon _*t}\left( \psi _{m,\varepsilon _*}^-(y,y_0)-\psi _{m,\varepsilon _*}^+(y,y_0) \right) \\&\quad +\left( \textrm{e}^{m\varepsilon _*t}-\textrm{e}^{-m\varepsilon _*t} \right) \psi _{m,\varepsilon _*}^+(y,y_0) \end{aligned} \end{aligned}$$

while

$$\begin{aligned} \begin{aligned}&\psi _{m,\varepsilon _*}^-(y,y_0)-\psi _{m,\varepsilon _*}^+(y,y_0)\\&\quad = -\frac{2i\varepsilon _*}{\beta ^2}{\omega }_m^0 +\int _0^1 \left( {\mathcal {G}}_{m,\varepsilon _*}^-(y,y_0,z)-{\mathcal {G}}_{m,\varepsilon _*}^+(y,y_0,z)\right) F_{m}(z,0)\textrm{d}z \\&\qquad +\frac{i\varepsilon _*}{\beta ^2}\int _0^1 \left( {\mathcal {G}}_{m,\varepsilon _*}^-(y,y_0,z)+{\mathcal {G}}_{m,\varepsilon _*}^+(y,y_0,z)\right) {\Delta }_m{\omega }_m^0\textrm{d}z. \end{aligned} \end{aligned}$$

Now, for $r\textrm{e}^{i\theta }=-y_0+i\varepsilon _*$, we use Lemma 6.7 to bound

$$\begin{aligned} \varepsilon _* \left\| \int _0^1 \left( {\mathcal {G}}_{m,\varepsilon _*}^-(y,y_0,z)+{\mathcal {G}}_{m,\varepsilon _*}^+(y,y_0,z)\right) {\Delta }_m{\omega }_m^0\textrm{d}z \right\| _{L^2_y}\lesssim \frac{\varepsilon }{\sinh ^2(\nu \theta )}\lesssim \frac{\varepsilon }{\sinh ^2(\nu \theta _*)} \end{aligned}$$

and, together with the orthogonality condition of the initial data,

$$\begin{aligned} \left\| \int _0^1 \left( {\mathcal {G}}_{m,\varepsilon _*}^-(y,y_0,z)-{\mathcal {G}}_{m,\varepsilon _*}^+(y,y_0,z)\right) F_{m}(z,0)\textrm{d}z \right\| _{L^2_y}\lesssim \frac{r^\frac{1}{2}}{\sinh ^2(\nu \theta )}\lesssim \frac{r_*^\frac{1}{2}}{\sinh ^2(\nu \theta _*)}, \end{aligned}$$

where $r_*\textrm{e}^{i\theta _*}=y_*+i\varepsilon _*$. With this bound uniform in $y_0\in [y_*,0]$, we obtain

$$\begin{aligned}  &   \int _0^{y_*}\Vert \psi _{m,\varepsilon }^-(y,y_0)-\psi _{m,\varepsilon }^+(y,y_0)\Vert _{L^2_y}\textrm{d}y_0 \lesssim \varepsilon |y_*| + \frac{r_*^\frac{1}{2}}{\sinh ^2(\nu \theta _*)}|y_*| + \frac{\varepsilon }{\sinh ^2(\nu \theta _*)}|y_*|\\  &   \quad \lesssim r_*^\frac{3}{2}\frac{\cos (\theta _*)}{\sinh ^2(\nu \theta _*)}. \end{aligned}$$

On the other hand,

$$\begin{aligned} \left\| \int _0^{|y_*|} \left( \textrm{e}^{m\varepsilon _*t}-\textrm{e}^{-m\varepsilon _*t}\right) \psi _{m,\varepsilon _*}^+(y,y_0)\textrm{d}y_0 \right\| _{L^2_y}\lesssim \frac{\varepsilon _*|y_*|}{\sinh ^2(\nu \theta )}\lesssim \frac{r_*^2}{\sinh ^2(\nu \theta _*)}, \end{aligned}$$

For the second part of the proposition, we recall that

$$\begin{aligned} \begin{aligned} \rho _{m,\varepsilon _*}^-(y,y_0)&- \rho _{m,\varepsilon _*}^+(y,y_0) \\&= \frac{1}{y-y_0-i\varepsilon _*}\int _0^1\big ( {\mathcal {G}}_{m,\varepsilon _*}^-(y,y_0,z)-{\mathcal {G}}_{m,\varepsilon _*}^+(y,y_0,z)\big )F_m(z,y_0)\textrm{d}z \\&\quad + \frac{2i\varepsilon _*}{(y-y_0)^2+\varepsilon _*^2}\int _0^1{\mathcal {G}}_{m,\varepsilon _*}^+(y,y_0,z)F_m(z,y_0)\textrm{d}z \\&\quad + \frac{i\varepsilon _*}{\beta }\int _0^1\left( \frac{1}{y-y_0-i\varepsilon _*}{\mathcal {G}}_{m,\varepsilon _*}^-(y,y_0,z)+\frac{1}{y-y_0+i\varepsilon _*}{\mathcal {G}}_{m,\varepsilon _*}^+(y,y_0,z)\right) \\&\quad {\Delta }_m{\omega }_m^0\textrm{d}z. \end{aligned} \end{aligned}$$

Using the bounds of Lemma 6.7 and the orthogonal condition on the initial data, we bound

$$\begin{aligned} \begin{aligned} |\rho _{m,\varepsilon _*}^-(y,y_0) - \rho _{m,\varepsilon _*}^+(y,y_0)|&\lesssim \frac{1}{|y-y_0-i\varepsilon _*|}\frac{|y-y_0+i\varepsilon _*|^\frac{1}{2}}{\sinh ^2(\nu \theta )}\\&\quad + \frac{\varepsilon _*}{|y-y_0+i\varepsilon _*|^2}\frac{|y-y_0+i\varepsilon _*|^\frac{1}{2}}{\sinh ^2(\nu \theta )} \\&\quad + \frac{|y-y_0+i\varepsilon _*|^\frac{1}{2}}{\sinh ^2(\nu \theta )} \\&\lesssim \frac{1}{\sinh ^2(\nu \theta _*)}\frac{1}{|y-y_0+i\varepsilon _*|^\frac{1}{2}}. \end{aligned} \end{aligned}$$

Hence,

$$\begin{aligned} \int _0^{y_*} |\rho _{m,\varepsilon _*}^-(y,y_0) - \rho _{m,\varepsilon _*}^+(y,y_0)| \textrm{d}y_0 \lesssim \frac{1}{\sinh ^2(\nu \theta _*)}\int _0^{|y_*|}\frac{1}{|y_0|^\frac{1}{2}}\textrm{d}y_0 \lesssim \frac{|y_*|^\frac{1}{2}}{\sinh ^2(\nu \theta _*)} \end{aligned}$$

and similarly

$$\begin{aligned} \left\| \int _0^{y_*} \left( \textrm{e}^{m\varepsilon _*t}-\textrm{e}^{-m\varepsilon _*t}\right) |\rho _{m,\varepsilon _*}^+(y,y_0)| \textrm{d}y_0 \right\| _{L^2_y}\lesssim \frac{\varepsilon _*|y_*|^\frac{1}{2}}{\sinh ^2(\nu \theta _*)}\lesssim \frac{r_*^\frac{3}{2}}{\sinh ^2(\nu \theta _*)}. \end{aligned}$$

The proof is concluded. $\square$

We combine the estimates from Proposition 6.6 and Proposition 6.8 to obtain the following result.

##### Proposition 6.9

For all $\delta >0$, there exists $\theta _*\in \left( 0,\frac{\pi }{2}\right)$ such that, for $r_*=\sinh ^8(\nu \theta _*)$, $y_*=-r_*\cos (\theta _*)$ and $\varepsilon _*=r_*\sin (\theta _*)$, there holds

$$\begin{aligned} \left\| \int _{ R_*^0}\textrm{e}^{-imct}\mathcal {R}(c,L_m)\textrm{d}c \right\| _{L^2_y}\le \delta . \end{aligned}$$

##### Proof

We choose $\theta _*>0$ such that $y_*=-r_*\cos (\theta _*)=-\sinh ^8(\nu \theta _*)\cos (\theta _*)=-p_k$, for some $k>0$, where $p_k$ is given by Proposition 6.3. This is possible because for $\theta _*$ small enough, $g(\theta _*):=\sinh ^8(\nu \theta _*)\cos (\theta _*)$ is a continuous strictly monotone increasing function of $\theta _*$ such that $g(0)=0$. Moreover, since $p_k\rightarrow 0^+$ for $k\rightarrow \infty$, we may assume $\theta _*$ is sufficiently small. Hence, $\frac{\varepsilon _*}{y_*}=\tan (\theta _*)$ is sufficiently small and we use Proposition 6.6 to bound

$$\begin{aligned} \left\| \int _0^{\varepsilon _*} \textrm{e}^{-imy_*t}\left( \textrm{e}^{mst}\psi _{m,s}^-(y,y_*)+\textrm{e}^{-mst}\psi _{m,s}^+(y,y_*) \right) \textrm{d}s\right\| _{L^2_y} \lesssim \varepsilon _* \lesssim \sinh ^8(\nu \theta _*), \end{aligned}$$

and

$$\begin{aligned} \left\| \int _0^{\varepsilon _*} \textrm{e}^{-imy_*t}\left( \textrm{e}^{mst}\rho _{m,s}^-(y,y_*)+\textrm{e}^{-mst}\rho _{m,s}^+(y,y_*) \right) \textrm{d}s\right\| _{L^2_y} \lesssim \varepsilon _*^\frac{1}{2}\lesssim \sinh ^4(\nu \theta _*). \end{aligned}$$

Now, we use Proposition 6.8 to bound

$$\begin{aligned} \begin{aligned}&\left\| \int _0^{y_*} \textrm{e}^{-imy_0t}\left( \textrm{e}^{m\varepsilon _*t}\psi _{m,\varepsilon _*}^-(y,y_0)-\textrm{e}^{-m\varepsilon _*t}\psi _{m,\varepsilon _*}^+(y,y_0) \right) \textrm{d}y_0 \right\| _{L^2_y} \\&\quad \lesssim \frac{r_*^\frac{3}{2}}{\sinh ^2(\nu \theta _*)} \lesssim \sinh ^{10}(\nu \theta _*) \end{aligned} \end{aligned}$$

and

$$\begin{aligned}&\left\| \int _0^{y_*} \textrm{e}^{-imy_0t}\big (\textrm{e}^{m\varepsilon _*t}\rho _{m,\varepsilon _*}^-(y,y_0)-\textrm{e}^{-m\varepsilon _*t}\rho _{m,\varepsilon _*}^+(y,y_0)\big )\textrm{d}y_0\right\| _{L^2_y}\\&\quad \lesssim \frac{r_*^\frac{1}{2}}{\sinh ^2(\nu \theta _*)}\lesssim \sinh ^2(\nu \theta _*). \end{aligned}$$

The proposition follows choosing $\theta _*$ small enough (that is, $\theta _*=g^{-1}(p_k)$ for $k>0$ sufficiently large). $\square$

We are finally in position to prove Proposition 6.1 for the case $\beta ^2>1/4$.

##### Proof of Proposition 6.1

We shall see that $\left\| \int _{\partial R_*{\setminus } R_*^{ess}}\textrm{e}^{-imct}\mathcal {R}(c,L_m)\textrm{d}c \right\| _{L^2_y}\le \delta$, for all $\delta >0$. Indeed, given $\delta >0$, from Proposition 6.9 there exists $\theta _*$ such that $y_*=-\sinh ^8(\nu \theta _*)\cos (\theta _*)=-p_k$, for some $k>0$ large enough and such that

$$\begin{aligned} \left\| \int _{R_*^0\cup R_*^1}\textrm{e}^{-imct}\mathcal {R}(c,L_m)\textrm{d}c \right\| _{L^2_y}\le \delta . \end{aligned}$$

Now, there are finitely many isolated eigenvalues in $\Omega \setminus R_*$, they are real and lying in $(-\frac{\beta }{m},y_*)\cup (1-y_*, \frac{\beta }{m})$. Moreover, $\textrm{e}^{-imct}\mathcal {R}(c,L_m)$ is an holomorphic function, for all $c\in \Omega \setminus R_*$ such that $c\ne c_j$, for any of the finitely many discrete eigenvalues $c_j\in (-\frac{\beta }{m},y_*)\cup (1-y_*, \frac{\beta }{m})$. Thus,

$$\begin{aligned} \frac{1}{2\pi i}\int _{\partial \Omega \setminus \partial R_*}\textrm{e}^{-imct}\mathcal {R}(c,L_m)\textrm{d}c = \sum _{j=0}^k \textrm{e}^{-imc_jt} \mathbb {P}_{c_j} \begin{pmatrix} {\omega }_m^0 \\ \rho _m^0 \end{pmatrix}=0, \end{aligned}$$

where $$\mathbb {P}_{c_j} \begin{pmatrix} {\omega }_m^0 \\ \rho _m^0 \end{pmatrix}$$ denotes the spectral projection of $$\begin{pmatrix} {\omega }_m^0 \\ \rho _m^0\end{pmatrix}$$ associated to the eigenvalue $c_j$, see Lemma 6.11. With this, the proof is finished. $\square$

The next proposition shows that the generalized eigenspace associated to any discrete eigenvalues is, in fact, simple.

##### Proposition 6.10

Let $c\in {{\mathbb {R}}}$ be a discrete eigenvalue of $L_m$. Then $\ker \left( L_m -c\right) ^2 = \ker \left( L_m-c\right)$. In particular, *c* is a semi-simple eigenvalue.

##### Proof

Note that the pair $({\omega },\rho )\in \ker \left( L_m-c\right)$ if and only if

$$\begin{aligned} \begin{aligned} (y-c){\omega }+ \beta ^2\rho =0, \qquad -\psi + (y-c)\rho =0, \end{aligned} \end{aligned}$$

where, as usual, we denote $\psi =\Delta _m^{-1}{\omega }$. Hence, $\rho =\frac{\psi }{y-c}$ and the equation

$$\begin{aligned} \Delta _m\psi + \beta ^2\frac{\psi }{(y-c)^2}=0 \end{aligned}$$

characterizes the eigenfunctions of $L_m$ of eigenvalue $c\in {{\mathbb {R}}}$. Now, the pair $({\omega },\rho )\in \ker \left( L_m-c\right) ^2$ if and only if

$$\begin{aligned} \begin{aligned} (y-c)^2{\omega }- \beta ^2\psi + 2(y-c)\beta ^2\rho&= 0, \\ -\Delta _m^{-1}((y-c){\omega }) - (y-c)\psi + (y-c)^2\rho - \beta ^2\Delta _m^{-1}\rho&=0. \end{aligned} \end{aligned}$$

Obtaining $\rho$ in terms of ${\omega }$ from the first equation and plugging it into the second one, we see that ${\omega }$ solves

$$\begin{aligned}  &   0=-\Delta _m^{-1}((y-c){\omega }) -(y-c)\psi - \frac{(y-c)^3}{2\beta ^2}{\omega }+ \frac{y-c}{2}\psi \\  &   \quad +\frac{1}{2}\Delta _m^{-1}\left( \frac{1}{y-c}\left[ (y-c)^2{\omega }-\beta ^2\psi \right] \right) . \end{aligned}$$

Multiplying by $\overline{{\omega }}$ and integrating by parts, we see that

$$\begin{aligned} \begin{aligned} 0&=-\int _0^1\overline{\psi }(y-c){\omega }-\int _0^1 \overline{{\omega }}(y-c)\psi -\int _0^1 \frac{(y-c)^3}{2\beta ^2}|{\omega }|^2 \\&\quad + \int _0^1\overline{{\omega }}\frac{y-c}{2}\psi + \int _0^1 \overline{\psi }\frac{y-c}{2}{\omega }- \int _0^1 \frac{\beta ^2}{2}|\psi |^2 \\&=-\frac{1}{2\beta ^2}\int _0^1 (y-c)^3 \left( |{\omega }|^2 + \beta ^2\frac{\overline{\psi }{\omega }+ \overline{{\omega }}\psi }{(y-c)^2} + \beta ^4 \frac{|\psi |^2}{(y-c)^4}\right) \\&=-\frac{1}{2\beta ^2}\int _0^1 (y-c)^3\left| {\Delta }_m\psi + \beta ^2\frac{\psi }{(y-c)^2}\right| ^2. \end{aligned} \end{aligned}$$

Hence, since either $c<0$ or $c>1$, we conclude that the pair $({\omega },\rho )$ satisfies

$$\begin{aligned} \Delta _m\psi + \beta ^2\frac{\psi }{(y-c)^2}=0, \end{aligned}$$

with ${\Delta }_m\psi ={\omega }$. That is, $({\omega },\rho )\in \ker \left( L_m -c\right)$. $\square$

#### Existence of initial data satisfying the spectral conditions

Here we shall exhibit initial data for which the spectral conditions required on Theorem 1 are satisfied. The proof studies properties of the projection operators and follows a contradiction argument. For the sake of clarity, we drop the subscripts *m* as they play no role in the proof. Let

$$\begin{aligned} D(L_m)&:= \left\{ \begin{pmatrix} \psi \\ \rho \end{pmatrix}\in H^4\times H^2 :\, {\omega }:={\Delta }_m\psi , \quad {\omega }(0) = \rho (0) = {\omega }(1) = \rho (1) = 0, \right. \\&\qquad \left. \int _0^1 \phi _u(z)\Delta _m\left( \rho (z) - \frac{1}{\beta ^2}z{\omega }(z)\right) \textrm{d}z \right. \\&\quad \left. = \int _0^1 \phi _l(z){\Delta }_m\left( \rho (z) - \frac{1}{\beta ^2}(z-1){\omega }(z) \right) \textrm{d}z = 0 \right\} , \end{aligned}$$

and consider $L_m:D(L_m)\subset L^2\times L^2 \rightarrow L^2 \times L^2$, which can be written as

$$\begin{aligned} L_m = \begin{pmatrix} {\Delta }_m^{-1} (y {\Delta }_m) &  0 \\ 0 &  y \end{pmatrix} + \begin{pmatrix} 0 &  \beta ^2{\Delta }_m^{-1} \\ -1 &  0 \end{pmatrix} = L_m^0 + L_m^1. \end{aligned}$$

It is clear that $L_m^0$ has (0, 1) in its continuous spectrum, since for any $c\in (0,1)$, $(L_m^0 -c)$ is injective but, in general, $(L_m^0-c)$ does not admit $L^2$ bounded solutions (it suffices to see that $(y-c)\rho =g\in L^2$ does not have an $L^2$ solution $\rho$ if $g(c) \ne 0$). Moreover, $L_m^1:D(L_m)\subset L^2\times L^2 \rightarrow L^2\times L^2$ is a compact operator and thus (0, 1) belongs to the continuous spectrum of $L_m$.

The next step is to obtain a working formula for the spectral projections associated to each discrete eigenvalue. This is the goal of the following Lemmma.

##### Lemma 6.11

Let $(\psi ^0,\rho ^0)\in D(L_m)$ and $c_k<0$ a discrete eigenvalue given by Corollary 6.4. Then, there exists some constant $\textbf{W}_{c_k}\in {{\mathbb {C}}}$ such that

$$\begin{aligned} \mathbb {P}_{c_k} \begin{pmatrix} \psi ^0 \\ \rho ^0 \end{pmatrix} = -\textbf{W}_{c_k}\left( \int _0^1 \phi _{c_k}(z){\Delta }_m\left( \rho ^0(z) - \frac{1}{\beta ^2}(z-c_k)\omega ^0(z) \right) \textrm{d}z\right) \begin{pmatrix} \phi _{c_k} \\ \rho _{c_k} \end{pmatrix}. \end{aligned}$$

Likewise, for the eigenvalue $d_k=1-c_k$ we have

$$\begin{aligned} \mathbb {P}_{d_k} \begin{pmatrix} \psi ^0 \\ \rho ^0 \end{pmatrix} = -\textbf{W}_{d_k}\left( \int _0^1 \phi _{d_k}(z){\Delta }_m\left( \rho ^0(z) - \frac{1}{\beta ^2}(z-d_k)\omega ^0(z) \right) \textrm{d}z\right) \begin{pmatrix} \phi _{d_k} \\ \rho _{d_k} \end{pmatrix}, \end{aligned}$$

for some $$\textbf{W}_{d_k}\in {{\mathbb {C}}}$$.

##### Proof

We shall argue for the spectral projection associated to $c_k$. Recall that

$$\begin{aligned} \mathbb {P}_{c_k} \begin{pmatrix} \psi ^0 \\ \rho ^0 \end{pmatrix} = -\frac{1}{2\pi i}\int _{\Gamma _k} (L_m-c)^{-1}\begin{pmatrix} \psi ^0 \\ \rho ^0 \end{pmatrix} \textrm{d}c, \end{aligned}$$

where $\Gamma _k$ is any closed contour counter-clockwise oriented, lying on the resolvent of $L_m$ and only enclosing the eigenvalue $c_k$. Denoting $$\begin{pmatrix} \psi (y,c) \\ \rho (y,c) \end{pmatrix} = (L_m-c)^{-1}\begin{pmatrix} \psi ^0 \\ \rho ^0 \end{pmatrix}$$ we see that for $\omega ^0 = {\Delta }_m\psi ^0$, there holds

$$\begin{aligned} {\Delta }_m \psi (y,c) + \beta ^2 \frac{\psi (y,c)}{(y-c)^2} = \frac{\omega ^0(y)}{y-c} - \beta ^2\frac{\rho ^0(y)}{(y-c)^2} \end{aligned}$$

and

$$\begin{aligned} \rho (y,c) = \frac{\rho ^0(y) + \psi (y,c)}{y-c}. \end{aligned}$$

In particular, using Cauchy’s Integral Theorem, since $y\in [0,1]$,

$$\begin{aligned} \mathbb {P}_{c_k} \begin{pmatrix} \psi ^0 \\ \rho ^0 \end{pmatrix} = -\frac{1}{2\pi i }\int _{\Gamma _k} \begin{pmatrix} \psi (y,c)\\ \frac{\rho ^0(y) + \psi (y,c)}{y-c} \end{pmatrix} \textrm{d}c = -\frac{1}{2\pi i }\int _{\Gamma _k} \begin{pmatrix} \psi (y,c) \\ \frac{ \psi (y,c)}{y-c} \end{pmatrix} \textrm{d}c. \end{aligned}$$

On the other hand, using the Green’s function of the Taylor–Goldstein operator, we write

$$\begin{aligned} \begin{aligned} \psi (y,c)&= \int _0^1 {\mathcal {G}}_m(y,c,z) \left( \frac{\omega ^0(z)}{z-c} - \beta ^2\frac{\rho ^0(z)}{(z-c)^2} \right) \textrm{d}z, \\&= \int _0^1 \frac{1}{{\mathcal {W}}(c)}\left( \phi _u(y,c)\phi _l(y,c) \textbf{1}_{y>z} + \phi _l(z,c)\phi _u(z,c)\textbf{1}_{y<z}\right) \\&\quad \left( \frac{\omega ^0(z)}{z-c} - \beta ^2\frac{\rho ^0(z)}{(z-c)^2} \right) \textrm{d}z, \end{aligned} \end{aligned}$$

see Proposition 3.1. Now, $\phi _u$ and $\phi _l$ are holomorphic functions in *c* since both $y,z\in [0,1]$ and $c\in \Gamma _k$ lies strictly away from [0, 1]. Moreover, we note that

$$\begin{aligned} \phi _l(y,c_k)&= M_+(-c_k)M_-(y-c_k) - M_-(-c_k)M_+(y-c_k) \\&= \frac{M_-(-c_k)}{M_-(1-c_k)}\left( M_+(1-c_k)M_-(y-c_k) - M_+(1-c_k)M_-(y-c_k)\right) \\&=\frac{M_-(-c_k)}{M_-(1-c_k)} \phi _u(y,c_k). \end{aligned}$$

On the other hand, ${\mathcal {W}}(c)$ is holomorphic on and inside $\Gamma _k$ and its zeroes are precisely given by the discrete eigenvalues $c_k$ and are of order one. In particular, there is only one such zero in the open set enclosed by $\Gamma _k$. Indeed, since $c,c_k<0$ and $r(c_k) = 0$

$$\begin{aligned} \frac{\textrm{d}}{\textrm{d}c}{\mathcal {W}}(c)\Big |_{c=c_k} = 8\nu m|M_+(-c_k)||M_+(1-c_k)| r'(c_k). \end{aligned}$$

Additionally,

$$\begin{aligned} \begin{aligned} r'(c_k) = -\Theta '(1-c_k) + \Theta '(-c_k)&= \nu \left( \frac{1}{|M_+(-c_k)|^2} - \frac{1}{|M_+(1-c_k)|^2}\right) \\&= \nu \frac{|M_+(1-c_k)|^2 - |M_+(-c_k)|^2}{|M_+(1-c_k)|^2|M_+(-c_k)|^2} > 0, \end{aligned} \end{aligned}$$

due to the monotone properties of $|M_+(x)|$, confer Lemma A.5. Now, thanks to the Residue Theorem, we obtain

$$\begin{aligned} \frac{1}{2\pi i}&\int _{\Gamma _k}\psi (y,c)\textrm{d}c \\&= \int _0^1 \frac{1}{2\pi i}\int _{\Gamma _k}\frac{ \phi _u(y,c)\phi _l(y,c) \textbf{1}_{y>z} + \phi _l(z,c)\phi _u(z,c)\textbf{1}_{y<z}}{{\mathcal {W}}(c)} \\&\quad \left( \frac{\omega ^0(z)}{z-c} - \beta ^2\frac{\rho ^0(z)}{(z-c)^2} \right) \textrm{d}c\, \textrm{d}z \\&= \textbf{W}_{c_k}\phi _u(y,c_k)\int _0^1 \phi _u(z,c_k)\left( \frac{\omega ^0(z)}{z-c_k} - \beta ^2\frac{\rho ^0(z)}{(z-c_k)^2} \right) \textrm{d}z, \end{aligned}$$

where we have defined

$$\begin{aligned} \textbf{W}_{c_k}:=\frac{(-1)^k}{16\nu ^2 m^2}\frac{M_-(-c_k)}{M_-(1-c_k)} \frac{|M_+(1-c_k)||M_+(-c_k)|}{|M_+(1-c_k)|^2 - |M_+(-c_k)|^2}. \end{aligned}$$

A similar computation yields

$$\begin{aligned} \frac{1}{2\pi i}\int _{\Gamma _k}\frac{\psi (y,c)}{y-c} \textrm{d}c =\textbf{W}_{c_k} \frac{\phi _u(y,c_k)}{y-c_k}\int _0^1 \phi _u(z,c_k)\left( \frac{\omega ^0(z)}{z-c_k} - \beta ^2\frac{\rho ^0(z)}{(z-c_k)^2} \right) \textrm{d}z. \end{aligned}$$

Next, owing to the equation satisfied by $\phi _{c_k}=\phi _u(y,c_k)$, integrating by parts and taking into account the vanishing boundary values of both $(\psi ^0,\rho ^0)\in D(L_m)$, there holds

$$\begin{aligned} \int _0^1 \phi _{c_k}(z)\left( \frac{\omega ^0(z)}{z-c_k} - \beta ^2\frac{\rho ^0(z)}{(z-c)^2} \right) \textrm{d}z = \int _0^1 \phi _{c}(z){\Delta }_m\left( \rho ^0(z) - \frac{1}{\beta ^2}(z-c)\omega ^0(z) \right) \textrm{d}z, \end{aligned}$$

for all discrete eigenvalues $c_k$. The Lemma follows once we recall that $\rho _{c_k}=\frac{\phi _{c_k}}{y-c_k}$.

The proof of the statement for the eigenvalue $d_k=1-c_k$, follows the same argument, where now, for $d>1$, the homogeneous solutions to the Taylor–Goldstein equation are given by

$$\begin{aligned} \phi _l(y,d) = M_+(d)M_-(d-y) - M_-(d)M_+(d-y) \end{aligned}$$

and

$$\begin{aligned} \phi _u(y,d) = M_+(d-1)M_-(d-y) - M_-(d-1)M_+(d-y), \end{aligned}$$

together with the Wronskian

$$\begin{aligned} \mathcal {W}_{m,0}^\pm&= 4i\nu m \left( M_+(d)M_-(d-1) - M_-(d)M_+(d-1) \right) \\&= -8m\nu |M_+(d)||M_+(d-1)|\sin \left( \Theta (d) - \Theta (d-1) \right) . \end{aligned}$$

Similarly, for an eigenvalue $d=d_k$, we have the relation

$$\begin{aligned} \phi _u(y,d_k) = \frac{M_-(d_k-1)}{M_-(d_k)}\phi _l(y,d_k). \end{aligned}$$

and

$$\begin{aligned} \textbf{W}_{d_k} = \frac{(-1)^k}{16m^2\nu ^2}\frac{M_-(d_k-1)}{M_-(d_k)}\frac{|M_+(d_k)||M_+(d_k-1)|}{|M_+(d_k)|^2 - |M_+(d_k -1)|^2}, \end{aligned}$$

we omit the details. $\square$

We now show that the sum of the spectral projections give rise to well-defined operators.

##### Lemma 6.12

Let $1\le p < \infty$ and let $c_k$ and $d_k$ be the discrete eigenvalues of $L_m$. Then, $\sum _{k\ge 1} \mathbb {P}_{c_k}: D(L_m)\rightarrow W^{2,p}_0\times W^{1,p}_0$ and $\sum _{k\ge 1} \mathbb {P}_{d_k}: D(L_m)\rightarrow W^{2,p}_0\times W^{1,p}_0$ are well-defined bounded operators.

##### Proof

Let $$\begin{pmatrix} \psi \\ \rho \end{pmatrix}\in D(L_m)$$, we shall see that $$\sum _{k\ge 1} \mathbb {P}_{c_k} \begin{pmatrix} \psi \\ \rho \end{pmatrix}$$ defines a convergent series in *X*. Indeed, from Lemma 6.11, we have

$$\begin{aligned}  &   \left\| \mathbb {P}_{c_k} \begin{pmatrix} \psi \\ \rho \end{pmatrix} \right\| _{W^{2,p}_0\times W^{1,p}_0} \le |\textbf{W}_{c_k}|\left| \int _0^1 \phi _{c_k}(z){\Delta }_m\left( \rho (z) - \frac{1}{\beta ^2}(z-c_k)\omega (z) \right) \textrm{d}z\right| \\  &   \quad \left( \Vert \phi _{c_k}\Vert _{W^{2,p}_0} + \Vert \rho _{c_k}\Vert _{W^{1,p}_0} \right) \end{aligned}$$

Firstly, using Lemma A.5 we note that

$$\begin{aligned} |\textbf{W}_{c_k}| \lesssim |M_+(-c_k)|^2 \lesssim |c_k|. \end{aligned}$$

Secondly, by the explicit expression of the eigenfunction $\phi _{c_k}$ and its associated density, and using again Lemma A.5 we similarly have

$$\begin{aligned} \Vert \phi _{c_k} \Vert _{W^{2,p}_0} + \Vert \rho _{c_k}\Vert _{W^{1,p}_0} \lesssim \Vert |z-c_{k}|^{-\frac{3}{2}}\Vert _{L^p}\lesssim |c_k|^{\frac{1}{p}-\frac{3}{2}}. \end{aligned}$$

Thirdly, using the condition satisfied by functions on $D(L_m)$, we see that

$$\begin{aligned} \int _0^1 \phi _{c_k}(z)&{\Delta }_m\left( \rho (z) - \frac{1}{\beta ^2}(z-c_k)\omega (z) \right) \textrm{d}z \\&=\int _0^1 (\phi _{c_k}(z)-\phi _u(z)){\Delta }_m\left( \rho (z) - \frac{1}{\beta ^2}(z-c_k)\omega (z) \right) \textrm{d}z \\&\quad + \frac{c_k}{\beta ^2}\int _0^1 \phi _u(z){\Delta }_m\omega (z) \textrm{d}z, \end{aligned}$$

and together with the fact that $\Vert \phi _{c_k}\Vert _{L^2}\lesssim 1$, $\omega ,\rho \in H^2$, and $|\phi _{c_k}(z) - \phi _u(z)|\lesssim |c_k|^\frac{1}{2}$, uniformly in $z\in [0,1]$, see Lemma A.3, we conclude that

$$\begin{aligned} \left| \int _0^1 \phi _{c_k}(z){\Delta }_m\left( \rho (z) - \frac{1}{\beta ^2}(z-c_k)\omega (z) \right) \textrm{d}z \right| \lesssim |c_k|^\frac{1}{2}. \end{aligned}$$

Hence,

$$\begin{aligned} \Vert \mathbb {P}_{c_k}\Vert _{D(L_m)\rightarrow W^{2,p}_0 \times W^{1,p}_0} \lesssim |c_k|^\frac{1}{p}\lesssim e^{-\frac{C_0}{p}k} \end{aligned}$$

and, as a result, $\sum _{k\ge 1}\mathbb {P}_{c_k}:D(L_m)\rightarrow W^{2,p}_0 \times W^{1,p}_0$ is a strongly convergent bounded operator. The proof for $\sum _{k\ge 1}\mathbb {P}_{d_k}$ follows the same argument, one can show that $\Vert \mathbb {P}_{d_k}\Vert _{D(L_m)\rightarrow W^{2,p}_0 \times W^{1,p}_0} \lesssim |d_k-1|^\frac{1}{2}$, we omit the details. $\square$

The next lemma shows that there exists data in $D(L_m)$ that has trivial projection onto each eigenspace through a contradiction argument.

##### Lemma 6.13

There exists non-trivial $$\begin{pmatrix} \psi \\ \rho \end{pmatrix}\in D(L_m)$$ such that $$\mathbb {P}_{c_k} \begin{pmatrix} \psi \\ \rho \end{pmatrix} = \mathbb {P}_{d_k} \begin{pmatrix} \psi \\ \rho \end{pmatrix}=0$$, for all eigenvalues $c_k$ and $d_k$.

##### Proof

Assume towards a contradiction that $\textbf{I} = \sum _{k\ge 1}\mathbb {P}_{c_k} + \sum _{k\ge 1}\mathbb {P}_{d_k}$, where $\textbf{I}:D(L_m)\rightarrow W^{2,p}_0 \times W^{1,p}_0$ denotes the identity operator. Given any $$\begin{pmatrix} \psi \\ \rho \end{pmatrix}\in D(L_m)$$, we then have that

$$\begin{aligned} L_m\begin{pmatrix} \psi \\ \rho \end{pmatrix} = L_m \left( \sum _{g\ge 1}\mathbb {P}_{c_k}\begin{pmatrix} \psi \\ \rho \end{pmatrix} + \sum _{k\ge 1}\mathbb {P}_{d_k}\begin{pmatrix} \psi \\ \rho \end{pmatrix}\right) = \left( \sum _{k\ge 1} c_k \mathbb {P}_{c_k} + \sum _{k\ge 1}d_k\mathbb {P}_{d_k}\right) \begin{pmatrix} \psi \\ \rho \end{pmatrix} \end{aligned}$$

and thus we can identify $L_m = \sum _{k\ge 1} c_k \mathbb {P}_{c_k} + \sum _{k\ge 1}d_k\mathbb {P}_{d_k}$ in $D(L_m)$. Let $c\in (0,1)$ and define the operator $\textrm{R}_c = \sum _{k\ge 1}(c-c_k)^{-1}\mathbb {P}_{c_k}+ \sum _{k\ge 1}(c-d_k)^{-1}\mathbb {P}_{d_k}$. Note that

$$\begin{aligned} \textrm{R}_c&= \sum _{k\ge 1}\left( (c-c_k)^{-1} - c^{-1}\right) \mathbb {P}_{c_k} + \sum _{k\ge 1}\left( (c-d_k)^{-1} - c^{-1}\right) \mathbb {P}_{d_k} + c^{-1}\left( \sum _{k\ge 1}\mathbb {P}_{c_k} + \sum _{k\ge 1}\mathbb {P}_{d_k} \right) \end{aligned}$$

and further define $\lambda _k:= (c-c_k)^{-1} - c^{-1}$ and $\mu _k:= (c-d_k)^{-1} - c^{-1}$ for $k\ge 1$. In particular, there holds

$$\begin{aligned} \sum _{k\ge 1}|\lambda _k - \lambda _{k+1}| = \sum _{k\ge 1}\frac{|c_k-c_{k+1}|}{|c-c_k||c-c_{k+1}|}\le \sum _{k\ge 1}\frac{c_{k+1}-c_k}{c^2} = -\frac{c_1}{c^2}>0 \end{aligned}$$

and

$$\begin{aligned} \sum _{k\ge 1}|\mu _k - \mu _{k+1}| = \sum _{k\ge 1} \frac{|d_k-d_{k+1|}}{|d_k - c||d_{k+1}-c|} \le \frac{1}{(1-c)^2}\sum _{k\ge 1}(d_{k+1} - d_k) = \frac{1-d_1}{(1-c)^2} < 0. \end{aligned}$$

Next, define $\textrm{S}_n^0 = \sum _{k=1}^n \mathbb {P}_{c_k}$, $\textrm{S}_n^1 = \sum _{k=1}^n \mathbb {P}_{d_k}$ and observe that by Lemma 6.12 and the uniform bounded principle, the norms of the operators $\textrm{S}_n^0$ acting on $D(L_m)$ are uniformly bounded. Then,

$$\begin{aligned} \sum _{k=1}^K \lambda _k\mathbb {P}_{c_k} = \sum _{k=1}^K (\lambda _k - \lambda _{k+1})\textrm{S}_k^0 + \lambda _K \textrm{S}_{K+1}^0, \quad \sum _{k=1}^K \mu _k\mathbb {P}_{d_k} = \sum _{k=1}^K (\mu _k - \mu _{k+1})\textrm{S}_k^0 + \mu _K \textrm{S}_{K+1}^0 \end{aligned}$$

define bounded operators and both $\sum _{k\ge 1}\lambda _k\mathbb {P}_{c_k}$ and $\sum _{k\ge 1}\mu _k\mathbb {P}_{d_k}$ are well-defined norm convergent operators on $D(L_m)$. In particular,

$$\begin{aligned} \textrm{R}_c = \sum _{k\ge 1}\lambda _k\mathbb {P}_{c_k} + \sum _{k\ge 1}\mu _k\mathbb {P}_{d_k} + c^{-1}\textbf{I} \end{aligned}$$

is a well-defined bounded operator, given by norm convergent sums. On the other hand, owing to Lemma 6.12, we have that $\textrm{V}_c = \sum _{k\ge 1}(c-c_k)\mathbb {P}_{c_k} + \sum _{k\ge 1}(c-d_k)\mathbb {P}_{d_k}$ is given by a strongly convergent sum and, in fact, $\textrm{V}_c = (c-L_m)$. Moreover, since $\mathbb {P}_{\lambda }\mathbb {P}_{\mu }=0$ for any $\lambda ,\mu \in \lbrace c_k, d_k: k\ge 1 \rbrace$ such that $\lambda \ne \mu$ and $\mathbb {P}_\lambda ^2= \mathbb {P}_\lambda$, for all $\lambda \in \lbrace c_k, d_k: k\ge 1 \rbrace$, we have that

$$\begin{aligned} \textrm{R}_c\textrm{V}_c = \textrm{V}_c\textrm{R}_c&= \left( \sum _{k\ge 1}(c-c_k)^{-1}\mathbb {P}_{c_k} + \sum _{k\ge 1}(c-d_k)^{-1}\mathbb {P}_{d_k}\right) \\&\quad \left( \sum _{j\ge 1}(c-c_j)\mathbb {P}_{c_j} + \sum _{j\ge 1}(c-d_j)\mathbb {P}_{d_j}\right) \\&= \sum _{k\ge 1}\mathbb {P}_{c_k} + \sum _{k\ge 1} \mathbb {P}_{d_k} =\textbf{I}, \end{aligned}$$

which shows that $\textrm{R}_c=\textrm{V}_c^{-1}=(c-L_m)^{-1}$ is a bounded operator. Hence, $c\in (0,1)$ is in the resolvent of *L*. We thus reach a contradiction, since the interval (0, 1) belongs to the continuous spectrum of $L_m$. Therefore, it must the case that $\textbf{I} - \sum _{k\ge 1}\mathbb {P}_{c_k} - \sum _{k\ge 1}\mathbb {P}_{d_k}\ne 0$ and there exists $$\begin{pmatrix} \psi \\ \rho \end{pmatrix}\in D(L_m)$$ such that

$$\begin{aligned} \begin{pmatrix} \psi ^0 \\ \rho ^0 \end{pmatrix}:=\left( \textbf{I} - \sum _{k\ge 1}\mathbb {P}_{c_k} - \sum _{k\ge 1}\mathbb {P}_{d_k}\right) \begin{pmatrix} \psi \\ \rho \end{pmatrix}\ne 0, \quad \mathbb {P}_{c_k}\begin{pmatrix} \psi ^0 \\ \rho ^0 \end{pmatrix} = \mathbb {P}_{d_k}\begin{pmatrix} \psi ^0 \\ \rho ^0 \end{pmatrix} = 0, \end{aligned}$$

for all eigenvalues $c_k$ and $d_k$. As such, $$\begin{pmatrix} \psi ^0 \\ \rho ^0 \end{pmatrix}\in W^{2,p}_0 \times W^{1,p}_0$$ constitutes initial data for the linearized initial value problem with trivial projection on all discrete modes. Moreover, for such initial data, we can show that the spectral condition (H) is also satisfied. Indeed,

$$\begin{aligned} 0&= \int _0^1 \phi _{c_k}(z)\left( \frac{\omega ^0(z)}{z-c_k} - \beta ^2 \frac{\rho ^0(z)}{(z-c_k)^2} \right) \textrm{d}z \end{aligned}$$

and since

$$\begin{aligned} \phi _{c_k}(z)\left( \frac{\omega ^0(z)}{z-c_k} - \beta ^2 \frac{\rho ^0(z)}{(z-c_k)^2} \right) \rightarrow \phi _{u}(z)\left( \frac{\omega ^0(z)}{z} - \beta ^2 \frac{\rho ^0(z)}{z^2} \right) \end{aligned}$$

a.e. in (0, 1) for $k\rightarrow \infty$, the spectral condition (H) holds once we apply the Dominated Convergence Theorem. To that purpose,

$$\begin{aligned} |\phi _{c_k}(z)| \left| \frac{\omega ^0(z)}{z-c_k} - \beta ^2 \frac{\rho ^0(z)}{(z-c_k)^2} \right| \lesssim z^{-\frac{1}{2}}|\omega ^0(z)| + z^{-\frac{1}{2}}\frac{|\rho ^0(z)|}{z}, \end{aligned}$$

which belongs to $L^1$, as can be seen using Hölder’s and Hardy’s inequalities. Thus, as $k\rightarrow \infty$ we conclude that

$$\begin{aligned} \int _0^1 \phi _u(z)\left( \frac{\omega ^0(z)}{z} - \beta ^2 \frac{\rho ^0(z)}{z^2} \right) \textrm{d}z = 0, \end{aligned}$$

and the same conclusion holds for the condition associated to $\phi _l(z)$ as $\textrm{d}_k\rightarrow 1$, we omit the details. $\square$

##### Remark 6.14

The space $D(L_m)$ is non-empty. Indeed, let $f,g\in H^2$ such that $\frac{g}{z}\in L^2$ and set

$$\begin{aligned} \omega (z):= \beta ^2(g(z) - f(z)) \quad \rho (z):= zg(z) - (z-1)f(z) \end{aligned}$$

so that $$\begin{pmatrix} \psi \\ \rho \end{pmatrix}\in D(L_m)$$ provided that

$$\begin{aligned} 0&=\int _0^1 \phi _u(z){\Delta }_m\left( \rho (z) - \frac{1}{\beta ^2}z {\omega }(z)\right) \textrm{d}z = \int _0^1 \phi _u(z){\Delta }_m f(z) \textrm{d}z, \\ 0&=\int _0^1 \phi _l(z) {\Delta }_m\left( \rho (z) - \frac{1}{\beta ^2}(z-1){\omega }(z) \right) \textrm{d}z = \int _0^1 \phi _l(z){\Delta }_m g(z) \textrm{d}z. \end{aligned}$$

Let $\eta (z)\in C^\infty _0(0,1)$ it is supported strictly away from 0 and 1 and such that $\eta (z)\ge 0$ for all $z\in (0,1)$. The above integral orthogonality conditions are satisfied for the unique solutions $f(z),g(z)\in H^2\cap H_0^1$ to the problems

$$\begin{aligned} {\Delta }_m f(z)= \eta (z)\left( f_0(z) - \left( \int _0^1 \eta (z)\phi _u(z)f_0(z) \textrm{d}z\right) \frac{\overline{\eta (z)\phi _u(z)}}{\Vert \eta (z)\phi _u\Vert _{L^2}^2}\right) , \\ {\Delta }_mg(z)= \eta (z)\left( g_0(z) - \left( \int _0^1 \eta (z)\phi _l(z)g_0(z) \textrm{d}z\right) \frac{\overline{z\phi _l(z)}}{\Vert \eta (z)\phi _l\Vert _{L^2}^2}\right) , \end{aligned}$$

for any $f_0,g_0\in L^2$. Another (perhaps simpler) instance of an element in $D(L_m)$ is the following: for any $\varpi \in L^2$, we define $\omega (z)\in H^2\cap H_0^1$ as the unique solution to

$$\begin{aligned} {\Delta }_m\omega (z) = \varpi (z)- \left( \int _0^1 \phi _l(z)\varpi (z) \textrm{d}z\right) \frac{\overline{\phi _l(z)}}{\Vert \phi _l\Vert _{L^2}^2}. \end{aligned}$$

Then, it is straightforward to see that $\omega (z)$ and $\rho (z):= \frac{z\omega (z)}{\beta ^2}$ are in $D(L_m)$. $\square$

#### Proof of Theorem 4

We finish the subsection proving Theorem 4 for $\beta ^2> 1/4$. We need the following lemma, which shows that for $y_0\in \lbrace 0, 1\rbrace$, the difference ${\mathcal {G}}_{m,\varepsilon }^-(y,y_0)-{\mathcal {G}}_{m,\varepsilon }^+(y,y_0)$ approaches a varying multiple of a generalized eigenfunction of the linearized operator as $\varepsilon \rightarrow 0$ associated to the “embedded eigenvalue" $c=0$. We state the result for $y_0=0$, since the case $y_0=1$ is analogous.

##### Lemma 6.15

Let $y_0=0$ and $0<\varepsilon \ll 1$ sufficiently small. Then, there exists $C_\varepsilon \in {{\mathbb {R}}}$ such that

$$\begin{aligned} \left| {\mathcal {G}}_{m,\varepsilon }^-(y,0,z)-{\mathcal {G}}_{m,\varepsilon }^+(y,0,z)-C_\varepsilon \phi _{u,m}(y)\phi _{u,m}(z)\right| \lesssim \varepsilon ^\frac{1}{2}, \end{aligned}$$

where $\phi _{u,m}$ is given in (3.7).

##### Proof

The result is trivially true for $y=0$ and $y=1$ because both $\phi _u$ and ${\mathcal {G}}^{\pm }$ vanish there. Therefore, in the sequel we consider $0<y<1$. Due to the complex conjugation property of the Green’s function,

$$\begin{aligned} {\mathcal {G}}_{m,\varepsilon }^-(y,0,z)- {\mathcal {G}}_{m,\varepsilon }^+(y,0,z)=2i\textrm{Im}\left( {\mathcal {G}}_{m,\varepsilon }^-(y,0,z)\right) \end{aligned}$$

Assuming initially that $y\le z$, we have

$$\begin{aligned} 2i\textrm{Im}\left( {\mathcal {G}}_{m,\varepsilon }^-(y,0,z)\right)&=2i\textrm{Im}\left( \frac{\phi _{l,m,\varepsilon }^-(y,0)\phi _{u,m,\varepsilon }^-(z,0)}{{{\mathcal {W}}_{m,\varepsilon }^-}(0)}\right) \\&=\frac{2i}{|{{\mathcal {W}}_{m,\varepsilon }^-}(0)|^2}\textrm{Im}\left( \phi _{l,m,\varepsilon }^-(y,0)\phi _{u,m,\varepsilon }^-(z,0){\mathcal {W}}_{m,\varepsilon }^+(0)\right) . \end{aligned}$$

Due to the explicit solutions of the Taylor–Goldstein equation, we write

$$\begin{aligned} \begin{aligned}&{\phi _{l,m,\varepsilon }^-(y,0)}{{\mathcal {W}}_{m,\varepsilon }^+(0)}\\&\quad = 4i\nu m\Big ( M_+(-i\varepsilon )M_-(y)M_+(i\varepsilon )M_-(1) + M_-(-i\varepsilon )M_+(y)M_-(i\varepsilon )M_+(1) \Big . \\&\qquad - \Big . M_+(-i\varepsilon )M_-(y)M_-(i\varepsilon )M_+(1) - M_-(-i\varepsilon )M_+(y)M_+(i\varepsilon )M_-(1) + R_1(\varepsilon )\Big ), \end{aligned} \end{aligned}$$

where $|R_1(\varepsilon )|\lesssim _{m,\nu } \varepsilon ^\frac{1}{2} |M_+(i\varepsilon )|^2$. Observe also that since $\overline{M_\pm (\zeta )}=M_\mp (\overline{\zeta })$ for all $\zeta \in {{\mathbb {C}}}$, we can write

$$\begin{aligned} \begin{aligned} {\phi _{l,m,\varepsilon }^-(y,0)}{{\mathcal {W}}_{m,\varepsilon }^+(0)}&= 4i\nu m \Big ( 2\textrm{Re}\big (M_+(-i\varepsilon )M_-(y)M_+(i\varepsilon )M_-(1)\big ) \Big . \\&\quad -\Big . |M_+(-i\varepsilon )|^2M_-(y)M_+(1) - |M_+(i\varepsilon )|^2M_+(y)M_-(1)+R_1(\varepsilon )\Big ) \end{aligned} \end{aligned}$$

Since $M_+(-i\varepsilon )=-i\textrm{e}^{\nu \pi }M_+(i\varepsilon )$, we obtain

$$\begin{aligned} \begin{aligned} {\phi _{l,m,\varepsilon }^-(y,0)}{{\mathcal {W}}_{m,\varepsilon }^+(0)}&= 4i\nu m \Big ( 2\textrm{Re}\big (M_+(-i\varepsilon )M_-(y)M_+(i\varepsilon )M_-(1)\big ) \Big .\\&\quad -\Big . \textrm{e}^{2\nu \pi }|M_+(i\varepsilon )|^2M_-(y)M_+(1) - |M_+(i\varepsilon )|^2M_+(y)M_-(1) + R_1(\varepsilon ) \Big ). \end{aligned} \end{aligned}$$

Now, $\overline{M_-(y)M_+(1)}=M_+(y)M_-(1)$ and we further observe that

$$\begin{aligned}  &   \textrm{Im}\big ( M_+(y)M_-(1)\big ) = - \textrm{Im}\big ( M_-(y)M_+(1)\big ) = -\frac{1}{2i}\big ( M_-(y)M_+(1) - M_+(y)M_-(1)\big )\\  &   \quad =-\frac{1}{2i}\phi _{u,m}(y). \end{aligned}$$

On the other hand,

$$\begin{aligned} \begin{aligned} \phi _{u,m,\varepsilon }^-(z,0)&=M_+(1)M_-(z)-M_-(1)M_+(z) + R_2(\varepsilon ) =2i\textrm{Im}\left( M_+(1)M_-(z)\right) + R_2(\varepsilon ) \end{aligned} \end{aligned}$$

with $$|R_2(\varepsilon )|\lesssim \varepsilon ^\frac{1}{2}$$. Thus,

$$\begin{aligned} \begin{aligned}&\phi _{l,m,\varepsilon }^-(y,0)\phi _{u,m,\varepsilon }^-(z,0){\mathcal {W}}_{m,\varepsilon }^+(0) \\&\quad =-8\nu m\textrm{Im}\left( M_+(1)M_-(z)\right) \Big ( 2\textrm{Re}\big (M_+(-i\varepsilon )M_-(y)M_+(i\varepsilon )M_-(1)\big ) \Big .\\&\qquad -\Big . \textrm{e}^{2\nu \pi }|M_+(i\varepsilon )|^2M_-(y)M_+(1) - |M_+(i\varepsilon )|^2M_+(y)M_-(1) \Big ) + R_3(\varepsilon ) \\&\quad =-8\nu m\textrm{Im}\left( M_+(1)M_-(z)\right) \Big ( 2\textrm{Re}\big (M_+(-i\varepsilon )M_-(y)M_+(i\varepsilon )M_-(1)\big )\Big . \\&\qquad -\Big . |M_+(i\varepsilon )|^2\left( \textrm{e}^{2\nu \pi }+1\right) \textrm{Re}\left( M_-(y)M_+(1)\right) \Big ) \\&\qquad -8i\nu m\textrm{Im}\left( M_+(1)M_-(z)\right) |M_+(i\varepsilon )|^2\left( \textrm{e}^{2\nu \pi }-1\right) \textrm{Im}\left( M_-(y)M_+(1)\right) + R_3(\varepsilon ), \end{aligned} \end{aligned}$$

where $|R_3(\varepsilon )|\lesssim \varepsilon ^\frac{1}{2}|M_+(i\varepsilon )|^2$, uniformly in $y,z\in [0,1]$. In particular,

$$\begin{aligned}  &   \textrm{Im}\left( \phi _{l,m,\varepsilon }^-(y,0)\phi _{u,m,\varepsilon }^-(z,0){\mathcal {W}}_{m,\varepsilon }^+(0)\right) \\  &   \quad =2\nu m|M_+(i\varepsilon )|^2\left( \textrm{e}^{2\nu \pi }-1\right) \phi _{u,m}(y)\phi _{u,m}(z) + \textrm{Im}\left( R_3(\varepsilon )\right) . \end{aligned}$$

Moreover, due to the symmetry of the Green’s function with respect to *y* and *z*, we also have that

$$\begin{aligned}  &   \textrm{Im}\left( \phi _{l,m,\varepsilon }^-(z,0)\phi _{u,m,\varepsilon }^-(y,0){\mathcal {W}}_{m,\varepsilon }^+(0)\right) \\  &   \quad =2\nu m|M_+(i\varepsilon )|^2\left( \textrm{e}^{2\nu \pi }-1\right) \phi _{u,m}(y)\phi _{u,m}(z) + \textrm{Im}\left( \widetilde{R}_3(\varepsilon )\right) . \end{aligned}$$

Let us now estimate the modulus squared of the Wronskian, that is, $|{\mathcal {W}}_{m,\varepsilon }(0)|^2$. We trivially have that

$$\begin{aligned} \begin{aligned} |{\mathcal {W}}_{m,\varepsilon }(0)|^2&= 16\nu ^2 m^2 \Big [ |M_+(-i\varepsilon )|^2|M_-(1-i\varepsilon )|^2 + |M_+(i\varepsilon )|^2|M_-(1+i\varepsilon )|^2\Big . \\&\qquad \Big . -2\textrm{Re}\Big (M_+(-i\varepsilon )M_-(1-i\varepsilon )M_+(i\varepsilon )M_-(1+i\varepsilon )\Big )\Big ] \\&=16\nu ^2 m^2 \Big [ \textrm{e}^{2\nu \pi }|M_+(i\varepsilon )|^2|M_-(1-i\varepsilon )|^2+|M_+(i\varepsilon )|^2|M_-(1+i\varepsilon )|^2 \Big . \\&\qquad \Big . -2\textrm{e}^{\nu \pi }|M_+(i\varepsilon )|^2|M_-(1-i\varepsilon )||M_-(1+i\varepsilon )|\cos (\theta _\varepsilon )\Big ] \end{aligned} \end{aligned}$$

where $\theta _\varepsilon = \text {Arg}\big (M_+(-i\varepsilon )M_-(1-i\varepsilon )M_+(i\varepsilon )M_-(1+i\varepsilon )\big )$. As before, since $M_-(\zeta )$ is smooth at $\zeta =1$, we can further write

$$\begin{aligned} \begin{aligned} |{\mathcal {W}}_{m,\varepsilon }(0)|^2&=16\nu ^2m^2|M_+(i\varepsilon )|^2|M_-(1)|^2\left( \textrm{e}^{2\nu \pi }-2\textrm{e}^{\nu \pi }\cos (\theta _\varepsilon )+1\right) +R_4(\varepsilon ) \end{aligned} \end{aligned}$$

where $|R_4(\varepsilon )|\lesssim |\varepsilon |^\frac{1}{2} |M_+(i\varepsilon )|^2$. With this, we are able to write

$$\begin{aligned} \begin{aligned}&{\mathcal {G}}_{m,\varepsilon }^-(y,0,z)-{\mathcal {G}}_{m,\varepsilon }^+(y,0,z)=\frac{2i}{{|{\mathcal {W}}_{m,\varepsilon }^-(0)|^2}}\textrm{Im}\left( \phi _{l,m,\varepsilon }^-(y,0)\phi _{u,m,\varepsilon }^-(z,0){\mathcal {W}}_{m,\varepsilon }^+(0)\right) \\&\quad = -\frac{i}{4\nu m}\frac{|M_+(i\varepsilon )|^2|\left( \textrm{e}^{2\nu \pi }-1\right) \phi _u(y)\phi _u(z)}{|M_+(i\varepsilon )|^2|M_-(1)|^2\left( \textrm{e}^{2\nu \pi }-2\textrm{e}^{\nu \pi }\cos (\theta _\varepsilon )+1\right) +R_4(\varepsilon )}\\&\qquad +\frac{i}{8\nu ^2m^2}\frac{\textrm{Im}\left( R_3(\varepsilon )\right) }{|M_+(i\varepsilon )|^2|M_-(1)|^2\left( \textrm{e}^{2\nu \pi }-2\textrm{e}^{\nu \pi }\cos (\theta _\varepsilon )+1\right) +R_4(\varepsilon )} \end{aligned} \end{aligned}$$

The lemma follows for

$$\begin{aligned} C_\varepsilon :=-\frac{i}{4\nu m}\frac{\textrm{e}^{2\nu \pi }-1}{|M_-(1)|^2\left( \textrm{e}^{2\nu \pi }-2\textrm{e}^{\nu \pi }\cos (\theta _\varepsilon )+1\right) }. \end{aligned}$$

and recalling that $\left| \textrm{Im}\left( R_3(\varepsilon )\right) \right| \lesssim \varepsilon ^\frac{1}{2} |M_+(i\varepsilon )|^2$, $\square$

##### Remark 6.16

Note that $C_\varepsilon$ is bounded but $\lim _{\varepsilon \rightarrow 0}C_\varepsilon$ does not exist. Indeed, as can be seen from the asymptotic expansions of Lemma A.3, we have that $\theta _\varepsilon =2\nu \log (\varepsilon ) + 2\text {Arg}(M_-(1)) + O(\varepsilon )$, as $\varepsilon \rightarrow 0$. Thus, $\theta _\varepsilon$ diverges to $-\infty$ and $\cos (\theta _\varepsilon )$ does not converge. Hence, assumption (H) becomes necessary in order to have a well defined pointwise limiting absorption principle for $y_0=0$.

We are now in position to prove Theorem 4 for $\beta ^2>1/4$.

##### Proof of Theorem 4

For $y_0=0$ we have that

$$\begin{aligned} \begin{aligned} \psi _{m,\varepsilon }^-(y,y_0)-\psi _{m,\varepsilon }^+(y,y_0)&= -\frac{2i\varepsilon }{\beta ^2}{\omega }_m^0 \!+\! \int _0^1 \left( {\mathcal {G}}_{m,\varepsilon }^-(y,0,z)-{\mathcal {G}}_{m,\varepsilon }^+(y,0,z)\right) F_{m}(z,0)\textrm{d}z \\&\quad +\frac{i\varepsilon }{\beta ^2}\int _0^1 \left( {\mathcal {G}}_{m,\varepsilon }^-(y,0,z)+{\mathcal {G}}_{m,\varepsilon }^+(y,0,z)\right) {\Delta }_m{\omega }_m^0\textrm{d}z. \end{aligned} \end{aligned}$$

Since ${\omega }_m^0\in H_y^1$, the first term vanishes easily, while the third term also tends to zero when $\varepsilon \rightarrow 0$, after a direct application of the Cauchy-Schwarz inequality and the facts that $\Vert {\mathcal {G}}_{m,\varepsilon }^\pm \Vert _{L^2_z}$ is uniformly bounded in $\varepsilon$ due to Theorem 2 and ${\omega }_m^0\in H_y^2$.

As for the second term, we invoke Lemma 6.15 to show that

$$\begin{aligned} \begin{aligned} \left| \int _0^1 ({\mathcal {G}}^-_{m,\varepsilon } -{\mathcal {G}}_{m,\varepsilon }^+)F_{m}(z) \textrm{d}z\right|&\le \left| C_\varepsilon \phi _u(y,0)\int _0^1 \phi _u(z,0)F_m(z,0) \textrm{d}z \right| \\&\quad + \left| \int _0^1 ({\mathcal {G}}_{m,\varepsilon }^- -{\mathcal {G}}_{m,\varepsilon }^+ -C_\varepsilon \phi _{u,m}(y)\phi _{u,m}(z))F_m(z) \textrm{d}z \right| \\&\lesssim \varepsilon ^\frac{1}{2}\int _0^1 |F_m(z)|\textrm{d}z \\&\lesssim \varepsilon ^\frac{1}{2} \left( \Vert \rho _m^0 \Vert _{H^{2}_y} + \Vert {\omega }_m^0 \Vert _{H^{2}_y} \right) , \end{aligned} \end{aligned}$$

which vanishes as $\varepsilon \rightarrow 0$. The proof for $y_0=1$ follows similarly from Lemma 6.15. We thus omit the details. $\square$

### Integral reduction for $\beta ^2<1/4$: no discrete eigenvalues

Thanks to the Hardy inequality [15]

6.4 $$\begin{aligned} \int _0^1 \left( \frac{1}{x}\int _0^x f(t)\textrm{d}t\right) ^2 \textrm{d}x\le 4\int _0^1 |f(t)|^2 \textrm{d}t, \qquad f\in L^2(0,1), \end{aligned}$$

we are able to prove $H^1$ bounds for the generalized stream functions $\psi _{m,\varepsilon }^\pm (y,y_0)$ that are uniform in $\varepsilon >0$.

#### Proposition 6.17

Let $y_0\in {{\mathbb {R}}}\setminus (0,1)$ and $0\le \varepsilon \le 1$. Then,

6.5 $$\begin{aligned} \Vert \partial _y \psi _{m,\varepsilon }^\pm (y,y_0)\Vert _{L^2}^2 + m^2 \Vert \psi _{m,\varepsilon }^\pm (y,y_0)\Vert _{L^2}^2 \lesssim \Vert {\omega }_m^0 \Vert _{H^2}^2 + \Vert \rho _m^0 \Vert ^2_{H^2}. \end{aligned}$$

Moreover,

$$\begin{aligned} \begin{aligned} \int _0^1\frac{\varepsilon (y-y_0)}{((y-y_0)^2+\varepsilon ^2)^2}|\varphi _{m,\varepsilon }^\pm |^2 \textrm{d}y \lesssim \Vert {\omega }_m^0 \Vert _{H^2}^2 + \Vert \rho _m^0 \Vert _{H^2}^2, \end{aligned} \end{aligned}$$

and

$$\begin{aligned} \Vert \rho _{m,\varepsilon }^\pm (y,y_0)\Vert _{L^2}^2 \lesssim \Vert {\omega }_m^0 \Vert _{H^2}^2 + \Vert \rho _m^0 \Vert _{H^2}^2. \end{aligned}$$

If we further assume that $|-y_0\pm i\varepsilon |\ge c_0$, for some $c_0>0$, then

$$\begin{aligned} \Vert \partial _y \psi _{m,\varepsilon }^\pm (y,y_0)\Vert _{L^2}^2 + m^2 \Vert \psi _{m,\varepsilon }^\pm (y,y_0)\Vert _{L^2}^2 \lesssim \frac{1}{c_0^2}\Vert {\omega }_m^0 \Vert _{L^2}^2 + \frac{1}{c_0^4}\Vert \rho _m^0 \Vert _{L^2} \end{aligned}$$

and

$$\begin{aligned} \Vert \rho _{m,\varepsilon }^\pm (y,y_0)\Vert _{L^2}^2 \lesssim \frac{1}{c_0^2}\Vert {\omega }_m^0 \Vert _{L^2}^2 + \frac{1}{c_0^4}\Vert \rho _m^0 \Vert _{L^2}^2. \end{aligned}$$

In particular, $c=-y_0\pm i\varepsilon$ belongs to the resolvent set of the operator $L_m$. $\square$

#### Proof

Multiplying (2.11) by $\overline{\varphi _{m,\varepsilon }^\pm (y,y_0)}$ and integrating by parts, we obtain

$$\begin{aligned}  &   \int _0^1 \left( |\partial _y\varphi _{m,\varepsilon }^\pm (y,y_0)|^2 + m^2 |\partial _y\varphi _{m,\varepsilon }^\pm (y,y_0)|^2 -\beta ^2\frac{|\varphi _{m,\varepsilon }^\pm (y,y_0)|^2}{(y-y_0\pm i\varepsilon )^2}\right) \textrm{d}y\\  &   \quad = -\int _0^1 F^\pm _{m,\varepsilon }(y,y_0)\varphi _{m,\varepsilon }^\pm (y,y_0)\textrm{d}y \end{aligned}$$

Assume now that $y_0\le 0$ (the case $y_0\ge 1$ would be done similarly) and observe that, thanks to the Hardy inequality (6.4) and $\varphi _{m,\varepsilon }^\pm (0,y_0)=0$,

$$\begin{aligned} \begin{aligned} \left| \beta ^2\int _0^1 \frac{|\varphi _{m,\varepsilon }^\pm (y,y_0)|^2}{(y-y_0\pm i\varepsilon )^2}\textrm{d}y\right|&\le \beta ^2 \int _0^1 \frac{|\varphi _{m,\varepsilon }^\pm (y,y_0)|^2}{y^2}\textrm{d}y \\&\le \beta ^2 \int _0^1 \left( \frac{1}{y}\int _0^y |\partial _y\varphi _{m,\varepsilon }^\pm (y',y_0)|\textrm{d}y'\right) ^2\textrm{d}y \\&\le 4\beta ^2\int _0^1 |\partial _y\varphi _{m,\varepsilon }^\pm (y,y_0)|^2 \textrm{d}y. \end{aligned} \end{aligned}$$

Therefore, we conclude that

$$\begin{aligned} (1-4\beta ^2)\int _0^1 |\partial _y\varphi _{m,\varepsilon }^\pm (y,y_0)|^2\textrm{d}y + m^2 \int _0^1 |\varphi _{m,\varepsilon }^\pm (y,y_0)|^2\textrm{d}y \lesssim \frac{1}{m^2}\int _0^1 |F_{m,\varepsilon }^\pm (y,y_0)|^2\textrm{d}y. \end{aligned}$$

Thus (6.5) follows from (2.9), the observation that $4\beta ^2<1$ and $\Vert F_{m,\varepsilon }^\pm (y,y_0)\Vert _{L^2_y}^2\lesssim \Vert {\omega }_m^0 \Vert _{H^2}^2 + \Vert \rho _m^0 \Vert _{H^2}$. For the second statement, we take the real and imaginary part of (2.11), for which we get

$$\begin{aligned}  &   \Delta _m \textrm{Re}(\varphi _{m,\varepsilon }^\pm )+\frac{1}{4((y-y_0)^2+\varepsilon ^2)^2}\left( ((y-y_0)^2-\varepsilon ^2)\textrm{Re}(\varphi _{m,\varepsilon }^\pm )\pm 2\varepsilon (y-y_0)\textrm{Im}(\varphi _{m,\varepsilon }^\pm )\right) \\  &   \quad =\textrm{Re}(F_{m,\varepsilon }^\pm ) \end{aligned}$$

and

$$\begin{aligned}  &   \Delta _m\textrm{Im}(\varphi _{m,\varepsilon }^\pm )+\frac{1}{4((y-y_0)^2+\varepsilon ^2)^2}\left( ((y-y_0)^2-\varepsilon ^2)\textrm{Im}(\varphi _{m,\varepsilon }^\pm )\mp 2\varepsilon (y-y_0)\textrm{Re}(\varphi _{m,\varepsilon }^\pm )\right) \\  &   \quad =\textrm{Im}(F_{m,\varepsilon }^\pm ). \end{aligned}$$

Cross multiplying the equations by $\textrm{Im}(\varphi _{m,\varepsilon }^\pm )$ and $\textrm{Re}(\varphi _{m,\varepsilon }^\pm )$, respectively, subtracting them and integrating, we obtain

$$\begin{aligned} \pm \int _0^1\frac{\varepsilon (y-y_0)}{2((y-y_0)^2+\varepsilon ^2)^2}|\varphi _{m,\varepsilon }^\pm |^2 \textrm{d}y = \int _0^1 \textrm{Im}(\varphi _{m,\varepsilon }^\pm )\textrm{Re}(F_{m,\varepsilon }^\pm )-\textrm{Re}(\varphi _{m,\varepsilon }^\pm )\textrm{Im}(F_{m,\varepsilon }^\pm )\textrm{d}y \end{aligned}$$

so that

$$\begin{aligned} \int _0^1\frac{\varepsilon (y-y_0)}{((y-y_0)^2+\varepsilon ^2)^2}|\varphi _{m,\varepsilon }^\pm |^2 \textrm{d}y \lesssim \int _0^1 |\varphi _{m,\varepsilon }^\pm |^2 + |F_{m,\varepsilon }^\pm |^2 \textrm{d}y \lesssim \Vert {\omega }_m^0 \Vert _{H^2}^2 + \Vert \rho _m^0 \Vert ^2_{H^2}. \end{aligned}$$

The third statement of the proposition follows from the density formula (2.10), the Hardy-type inequality and the uniform bounds from the first statement of the proposition. The proof is finished. $\square$

From the arguments of the proof, one can directly obtain the following result.

#### Corollary 6.18

Let $y_0 < 0$ or $y_0 > 1$. Then, $y_0+ic$ is not an eigenvalue of $L_m$, for any $c\in {{\mathbb {R}}}$.

With the $\varepsilon$-uniform $H^1_0$ bounds for $\psi _{m,\varepsilon }^\pm (y,y_0)$ at hand we are now able to prove the following result.

#### Proposition 6.19

We have that

$$\begin{aligned} \lim _{\varepsilon \rightarrow 0}\left\| \int _{-\frac{\beta }{m}}^0 \textrm{e}^{-imy_0t}\big (\textrm{e}^{m\varepsilon _*t}\psi _{m,\varepsilon _*}^-(y,y_0)-\textrm{e}^{-m\varepsilon _*t}\psi _{m,\varepsilon _*}^+(y,y_0) \big )\textrm{d}y_0\right\| _{L^2_y}=0 \end{aligned}$$

and

$$\begin{aligned} \lim _{\varepsilon \rightarrow 0}\left\| \int _{-\frac{\beta }{m}}^0 \textrm{e}^{-imy_0t}\big (\textrm{e}^{m\varepsilon _*t}\rho _{m,\varepsilon _*}^-(y,y_0)-\textrm{e}^{-m\varepsilon _*t}\rho _{m,\varepsilon _*}^+(y,y_0) \big )\textrm{d}y_0\right\| _{L^2_y}=0. \end{aligned}$$

#### Proof

Let us denote $\psi _{m,\varepsilon }(y,y_0) =\psi _{m,\varepsilon }^+(y,y_0) - \psi _{m,\varepsilon }^-(y,y_0)$. Using (2.9), we have

$$\begin{aligned} \psi _{m,\varepsilon }(y,y_0) = \frac{2i\varepsilon }{\beta ^2}{\omega }_m^0(y) +\varphi _{m,\varepsilon }^+(y,y_0) - \varphi _{m,\varepsilon }^-(y,y_0) \end{aligned}$$

and we further denote $\varphi _{m,\varepsilon }(y,y_0)= \varphi _{m,\varepsilon }^+(y,y_0) - \varphi _{m,\varepsilon }^-(y,y_0)$, which solves

$$\begin{aligned}  &   \left( \partial _y^2 -m^2 +\beta ^2\frac{1}{(y-y_0+i\varepsilon )^2}\right) \varphi _{m,\varepsilon }(y,y_0) = \beta ^2\frac{4i\varepsilon (y-y_0)}{((y-y_0)^2 + \varepsilon ^2)^2}\varphi _{m,\varepsilon }^-(y,y_0) \\  &   \quad -\frac{2i\varepsilon }{\beta ^2}{\Delta }_m{\omega }_m^0(y). \end{aligned}$$

Multiplying by $\overline{\varphi _{m,\varepsilon }(y,y_0)}$ integrating by parts and proceeding as before, we see

$$\begin{aligned}  &   (1-4\beta ^2)\int _0^1 |\partial _y\varphi _{m,\varepsilon }|^2 \textrm{d}y + m^2 \int _0^1 |\varphi _{m,\varepsilon }|^2 \textrm{d}y \lesssim \frac{2\varepsilon }{\beta ^2}\Vert {\omega }_m^0\Vert _{H^2}^2 \\  &   \quad + \beta ^2\int _0^1 \frac{4\varepsilon (y-y_0)}{((y-y_0)^2+\varepsilon ^2)^2}|\varphi _{m,\varepsilon }^-| |\varphi _{m,\varepsilon }|\textrm{d}y. \end{aligned}$$

Moreover, using Young’s inequality we can bound

$$\begin{aligned} \begin{aligned}&\beta ^2\int _0^1 \frac{4\varepsilon (y-y_0)}{((y-y_0)^2+\varepsilon ^2)^2}|\varphi _{m,\varepsilon }^-| |\varphi _{m,\varepsilon }|\textrm{d}y\\&\quad \le \beta ^2 \int _0^1 \frac{2\varepsilon (y-y_0)}{((y-y_0)^2+\varepsilon ^2)^2}\left( c_0^2|\varphi _{m,\varepsilon }|^2 +\frac{1}{c_0^2}|\varphi _{m,\varepsilon }^-|^2\right) \textrm{d}y \\&\quad \le c_0^2\beta ^2 \int _0^1 \frac{1}{y^2}||\varphi _{m,\varepsilon }|^2\textrm{d}y +\frac{\beta ^2}{c_0^2}\int _0^1 \frac{2\varepsilon (y-y_0)}{((y-y_0)^2+\varepsilon ^2)^2}|\varphi _{m,\varepsilon }^-|^2\textrm{d}y \\&\quad \le 4c_0^2\beta ^2 \int _0^1 |\partial _y\varphi _{m,\varepsilon }|^2 \textrm{d}y +\frac{\beta ^2}{c_0^2}\int _0^1 \frac{2\varepsilon (y-y_0)}{((y-y_0)^2+\varepsilon ^2)^2}|\varphi _{m,\varepsilon }^-|^2\textrm{d}y. \end{aligned} \end{aligned}$$

Therefore, absorbing the derivative term in the left hand side for some $c_0$ small enough, we obtain

6.6 $$\begin{aligned} \int _0^1 |\partial _y\varphi _{m,\varepsilon }|^2 \textrm{d}y + m^2\int _0^1 |\varphi _{m,\varepsilon }|^2 \textrm{d}y\lesssim \varepsilon \Vert {\omega }_m^0\Vert _{H^2}^2 + \int _0^1 \frac{2\varepsilon (y-y_0)}{((y-y_0)^2+\varepsilon ^2)^2}|\varphi _{m,\varepsilon }^-|^2\textrm{d}y. \nonumber \\ \end{aligned}$$

Given the uniform bounds in $\varepsilon >0$ from Proposition 6.17, we have that

$$\begin{aligned} \begin{aligned} \lim _{\varepsilon \rightarrow 0}&\left\| \int _{-\frac{\beta }{m}}^0 \textrm{e}^{-imy_0t}\big (\textrm{e}^{m\varepsilon t}\psi _{m,\varepsilon }^-(y,y_0)-\textrm{e}^{-m\varepsilon t}\psi _{m,\varepsilon }^+(y,y_0) \big )\textrm{d}y_0\right\| _{L^2_y}\\ \quad&=\lim _{\varepsilon \rightarrow 0}\left\| \int _{-\frac{\beta }{m}}^0 \textrm{e}^{-imy_0t}\textrm{e}^{m\varepsilon t}\big (\psi _{m,\varepsilon }^-(y,y_0)-\psi _{m,\varepsilon }^+(y,y_0) \big )\textrm{d}y_0\right\| _{L^2_y} \\ \quad&\le \lim _{\varepsilon \rightarrow 0} \int _{-\frac{\beta }{m}}^0 \left( \frac{2\varepsilon }{\beta }\Vert {\omega }_m^0\Vert _{L^2_y} + \left\| \varphi _{m,\varepsilon }(y,y_0)\right\| _{L^2_y}\right) \textrm{d}y_0 \\ \quad&= \lim _{\varepsilon \rightarrow 0} \int _{-\frac{\beta }{m}}^0 \left\| \varphi _{m,\varepsilon }(y,y_0)\right\| _{L^2_y}\textrm{d}y_0. \end{aligned} \end{aligned}$$

Now, note that with (6.6) we can estimate

$$\begin{aligned} \begin{aligned} \lim _{\varepsilon \rightarrow 0} \int _{-\frac{\beta }{m}}^0 \left\| \varphi _{m,\varepsilon }(y,y_0)\right\| _{L^2_y}\textrm{d}y_0&\lesssim \lim _{\varepsilon \rightarrow 0} \int _{-\frac{\beta }{m}}^0 \left( \int _0^1 \frac{2\varepsilon (y-y_0)}{((y-y_0)^2+\varepsilon ^2)^2}|\varphi _{m,\varepsilon }^-|^2\textrm{d}y\right) ^\frac{1}{2} \textrm{d}y_0 \\&\lesssim \lim _{\varepsilon \rightarrow 0} \varepsilon ^\frac{1}{2}\int _{-\frac{\beta }{m}}^0 \left( \int _0^1 \frac{1}{(y-y_0)^2}\textrm{d}y\right) ^\frac{1}{2} \textrm{d}y_0, \\ \end{aligned} \end{aligned}$$

where we have used the pointwise bound $|\varphi _{m,\varepsilon }^-(y,y_0)|^2\lesssim y$, obtained from Proposition 6.17. The conclusion follows.

For the density statement, we recall that

$$\begin{aligned} \rho _{m,\varepsilon }^-(y,y_0)-\rho _{m,\varepsilon }^+(y,y_0) = \frac{\varphi _{m,\varepsilon }(y,y_0)}{y-y_0-i\varepsilon } + \frac{2i\varepsilon }{(y-y_0)^2+\varepsilon ^2}\varphi _{m,\varepsilon }^+(y,y_0), \end{aligned}$$

from which, together with Proposition 6.17, we deduce that

$$\begin{aligned} \begin{aligned} \lim _{\varepsilon \rightarrow 0}&\left\| \int _{-\frac{\beta }{m}}^0 \textrm{e}^{-imy_0t}\big (\textrm{e}^{m\varepsilon _*t}\rho _{m,\varepsilon _*}^-(y,y_0)-\textrm{e}^{-m\varepsilon _*t}\rho _{m,\varepsilon _*}^+(y,y_0) \big )\textrm{d}y_0\right\| _{L^2_y}\\ \quad&\le \lim _{\varepsilon \rightarrow 0}\left\| \int _{-\frac{\beta }{m}}^0 \textrm{e}^{-imy_0t}\textrm{e}^{m\varepsilon _*t}\big (\rho _{m,\varepsilon _*}^-(y,y_0)-\rho _{m,\varepsilon _*}^+(y,y_0) \big )\textrm{d}y_0\right\| _{L^2_y} \\ \quad&\lesssim \lim _{\varepsilon \rightarrow 0} \int _{-\frac{\beta }{m}}^0 \left( \left\| \frac{\varphi _{m,\varepsilon }(y,y_0)}{y-y_0-i\varepsilon }\right\| _{L^2_y} + \left\| \frac{2i\varepsilon \varphi _{m,\varepsilon }^+(y,y_0)}{(y-y_0)^2+\varepsilon ^2}\right\| _{L^2_y}\right) \textrm{d}y_0 \\ \end{aligned} \end{aligned}$$

Using the Hardy inequality (6.4), the estimates from (6.6) and the above arguments, we have

$$\begin{aligned} \lim _{\varepsilon \rightarrow 0} \int _{-\frac{\beta }{m}}^0 \left\| \frac{\varphi _{m,\varepsilon }(y,y_0)}{y-y_0-i\varepsilon }\right\| _{L^2_y}\textrm{d}y_0 \lesssim \lim _{\varepsilon \rightarrow 0} \int _{-\frac{\beta }{m}}^0 \left\| \partial _y\varphi _{m,\varepsilon }(y,y_0)\right\| _{L^2_y}\textrm{d}y_0 =0. \end{aligned}$$

On the other hand, thanks to the bounds from Proposition 6.17, we also have

$$\begin{aligned}  &   \lim _{\varepsilon \rightarrow 0} \int _{-\frac{\beta }{m}}^0 \left\| \frac{2i\varepsilon \varphi _{m,\varepsilon }^+(y,y_0)}{(y-y_0)^2+\varepsilon ^2}\right\| _{L^2_y}\textrm{d}y_0\\  &   \quad \le \lim _{\varepsilon \rightarrow 0} \varepsilon ^\frac{1}{2} \int _{-\frac{\beta }{m}}^0 \left\| \frac{2\varepsilon ^\frac{1}{2}(y-y_0)^\frac{1}{2} \varphi _{m,\varepsilon }^+(y,y_0)}{(y-y_0)^2+\varepsilon ^2}\right\| _{L^2_y}\frac{1}{(-y_0)^\frac{1}{2}}\textrm{d}y_0=0. \end{aligned}$$

With this, the proof is finished. $\square$

We next show that the contribution from the vertical boundaries of the contour integral is also negligible.

#### Proposition 6.20

Let $y_0\in \left( -\frac{\beta }{m}, 0\right)$. We have that

$$\begin{aligned} \lim _{\varepsilon \rightarrow 0}\left\| \int _0^\varepsilon \textrm{e}^{-imy_0t}\big (\textrm{e}^{mst}\psi _{m,s}^-(y,y_0)+\textrm{e}^{-mst}\psi _{m,s}^+(y,y_0) \big )\textrm{d}s\right\| _{L^2_y}=0 \end{aligned}$$

and

$$\begin{aligned} \lim _{\varepsilon \rightarrow 0}\left\| \int _0^\varepsilon \textrm{e}^{-imy_0t}\big (\textrm{e}^{mst}\rho _{m,\varepsilon _*}^-(y,y_0)+\textrm{e}^{-mst}\rho _{m,s}^+(y,y_0) \big )\textrm{d}s\right\| _{L^2_y}=0. \end{aligned}$$

#### Proof

The statement follows from Minkowski inequality and the fact that

$$\begin{aligned} \lim _{\varepsilon \rightarrow 0} \int _0^\varepsilon \left\| \psi _{m,s}^-(y,y_0)\right\| _{L^2_y}+ \left\| \psi _{m,s}^+(y,y_0)\right\| _{L^2_y} + \left\| \rho _{m,s}^-(y,y_0)\right\| _{L^2_y} + \left\| \rho _{m,s}^+(y,y_0)\right\| _{L^2_y}\textrm{d}s=0, \end{aligned}$$

due to the uniform bounds in $s\in [0,\varepsilon ]$ of these quantities from Proposition 6.17. $\square$

We are now in position to carry out the proof of Proposition 6.1 for the case $\beta ^2<1/4$.

#### Proof of Proposition 6.1

The resolvent operator $\mathcal {R}(c,L_m)$ is invertible for all $c\in {{\mathbb {C}}}$ with $\textrm{Re}(c)\in {{\mathbb {R}}}{\setminus } (0,1)$ and $|c|\ge c_0$ for some $c_0>0$, confer Proposition 6.17. We can reduce the contour integral from $\partial \Omega$ to the boundary of the set $R_\varepsilon :=\Big \lbrace c=y_0 + is\in {{\mathbb {C}}}: y_0\in \Big [-\beta /m, 1+ \frac{\beta }{m}\Big ], \, s\in [-\varepsilon , \varepsilon ] \Big \rbrace$, after (possibly) collecting finitely many discrete eigenvalues lying on $(0,1)\times \left( (-\frac{\beta }{m}, \frac{\beta }{m}) {\setminus } (-\varepsilon _0, \varepsilon _0) \right)$, for some $\varepsilon _0 >0$. Indeed, Proposition 4.3 shows that the Wronskian is bounded from below in $(0,1)\times (-\varepsilon _0, \varepsilon _0)$, and since the Wronskian is holomorphic in ${{\mathbb {C}}}\setminus [0,1]$, it can only vanish finitely many times in $(0,1)\times \left( (-\frac{\beta }{m}, \frac{\beta }{m}) {\setminus } (-\varepsilon _0, \varepsilon _0) \right)$. Otherwise there would be an accumulation point of zeroes, and thus the Wronskian would necessarily be 0 on the whole open set. We then would have identically zero Wronskian well inside the region where Proposition 4.3 provides lower bounds for the Wronskian, thus reaching a contradiction.

Using the decomposition $\partial R_* = R_0^* \cup R_*^{ess} \cup R_1^*$, and taking any $y_*\in (-\frac{\beta }{m}, 0)$, we see from Proposition 6.19 and Proposition 6.20 that the integrals along $R_*^0$ and $R_*^1$ are negligible as $\varepsilon _*\rightarrow 0$. The result follows. $\square$

We finish the subsection with the proof of Theorem 4 for $\beta ^2<1/4$, which is a direct consequence of the following Lemma.

#### Lemma 6.21

Let $y,z\in [0,1]$. There exists $\varepsilon _0>0$ such that

$$\begin{aligned} \sup _{y,z\in [0,1]}\left| {\mathcal {G}}_{m,\varepsilon }^-(y,y_0,z)-{\mathcal {G}}_{m,\varepsilon }^+(y,y_0,z)\right| \lesssim \varepsilon ^{2\mu } + \varepsilon ^\frac{1}{2}, \qquad y_0\in \{0,1\}, \end{aligned}$$

for all $$\varepsilon \le \varepsilon _0$$.

#### Proof

We take $y_0=0$, the other case is analogous. The argument is similar to the one presented for Lemma 6.15. As before, we need to understand

$$\begin{aligned} {\mathcal {G}}_{m,\varepsilon }^-(y,0,z)- {\mathcal {G}}_{m,\varepsilon }^+(y,0,z)=2i\textrm{Im}\left( {\mathcal {G}}_{m,\varepsilon }^-(y,0,z)\right) \end{aligned}$$

For $y\le z$, we have

$$\begin{aligned} 2i\textrm{Im}\left( {\mathcal {G}}_{m,\varepsilon }^-(y,0,z)\right) =\frac{2i}{|{{\mathcal {W}}_{m,\varepsilon }^-}(0)|^2}\textrm{Im}\left( \phi _{l,m,\varepsilon }^-(y,0)\phi _{u,m\varepsilon }^-(z,0){\mathcal {W}}_{m,\varepsilon }^+(0)\right) . \end{aligned}$$

Due to the explicit solutions of the Taylor–Goldstein equation, we can find that

$$\begin{aligned} \begin{aligned}&{\phi _{l,m,\varepsilon }^-(y,0)}{{\mathcal {W}}_{m,\varepsilon }^+(0)} \\&\quad = 4\mu m\Big ( M_+(-i\varepsilon )M_-(y)M_+(i\varepsilon )M_-(1) + M_-(-i\varepsilon )M_+(y)M_-(i\varepsilon )M_+(1) \Big . \\&\qquad - \Big . M_+(-i\varepsilon )M_-(y)M_-(i\varepsilon )M_+(1) - \Big .M_-(-i\varepsilon )M_+(y)M_+(i\varepsilon )M_-(1) +R_1(\varepsilon )\Big ), \end{aligned} \end{aligned}$$

where $|R_1(\varepsilon )|\lesssim _{m,\mu } \varepsilon ^{\frac{1}{2}-\mu } |M_-(i\varepsilon )|^2$. Moreover, since now $\overline{M_\pm (\zeta )}=M_\pm (\overline{\zeta })$ for all $\zeta \in {{\mathbb {C}}}$, we can write

$$\begin{aligned} \begin{aligned}&{\phi _{l,m,\varepsilon }^-(y,0)}{{\mathcal {W}}_{m,\varepsilon }^+(0)}= 4\mu m \Big ( |M_+(i\varepsilon )|^2M_-(y)M_-(1) + |M_-(i\varepsilon )|^2M_+(y)M_+(1) \Big .\\&\quad - \Big . M_+(-i\varepsilon )M_-(i\varepsilon )M_-(y)M_+(1) - M_+(i\varepsilon )M_-(-i\varepsilon )M_+(y)M_-(1) + R_1(\varepsilon )\Big ). \end{aligned} \end{aligned}$$

On the other hand,

$$\begin{aligned} \begin{aligned} \phi _{u,m,\varepsilon }^-(z,0)&=M_+(1)M_-(z)-M_-(1)M_+(z) + R_2(\varepsilon ) = \phi _u(z) + R_2(\varepsilon )\\ \end{aligned} \end{aligned}$$

with $$|R_2(\varepsilon )|\lesssim \varepsilon ^{\frac{1}{2}-\mu }$$. Thus,

$$\begin{aligned} \begin{aligned}&\phi _{l,m,\varepsilon }^-(y,0)\phi _{u,m,\varepsilon }^-(z,0){\mathcal {W}}_{m,\varepsilon }^+(0) \\&\quad =-4\mu m \phi _u(z) \Big ( M_+(-i\varepsilon )M_-(i\varepsilon )M_-(y)M_+(1) + M_+(i\varepsilon )M_-(-i\varepsilon )M_+(y)M_-(1) \Big ) \\&\qquad +4\mu m \phi _u(z)\Big (|M_+(i\varepsilon )|^2M_-(y)M_-(1) + |M_-(i\varepsilon )|^2M_+(y)M_+(1)\Big ) + R_3(\varepsilon ), \end{aligned} \end{aligned}$$

where $|R_3(\varepsilon )|\lesssim \mu m \varepsilon ^{\frac{1}{2}-\mu }|M_-(i\varepsilon )|^2$, uniformly in $y,z\in [0,1]$. In particular, since $M_\pm (y)\in {{\mathbb {R}}}$, for all $y\in [0,1]$, we have

$$\begin{aligned}  &   \textrm{Im}\left( \phi _{l,m,\varepsilon }^-(y,0)\phi _{u,m,\varepsilon }^-(z,0){\mathcal {W}}_{m,\varepsilon }^+(0)\right) =-4\mu m \phi _{u,m}(z)\phi _{u,m}(y)\textrm{Im}\left( M_+(-i\varepsilon )M_-(i\varepsilon )\right) \\  &   \quad + \textrm{Im}\left( R_3(\varepsilon )\right) . \end{aligned}$$

Moreover, due to the symmetry of the Green’s function with respect to *y* and *z*, we also have that

$$\begin{aligned}  &   \textrm{Im}\left( \phi _{l,m,\varepsilon }^-(z,0)\phi _{u,m,\varepsilon }^-(y,0){\mathcal {W}}_{m,\varepsilon }^+(0)\right) =-4\mu m \phi _{u,m}(z)\phi _{u,m}(y)\textrm{Im}\left( M_+(-i\varepsilon )M_-(i\varepsilon )\right) \\  &   \quad + \textrm{Im}\left( \widetilde{R}_3(\varepsilon )\right) . \end{aligned}$$

For the Wronskian, we have from (3.6) that

$$\begin{aligned} \begin{aligned} \left| {\mathcal {W}}_{m,\varepsilon }^+(0)\right| =4\mu m|M_-(i\varepsilon )||M_+(1)|\left| 1- \frac{M_+(i\varepsilon )}{M_-(i\varepsilon )}\frac{M_-(1)}{M_+(1)} + R_4(\varepsilon )\right| . \end{aligned} \end{aligned}$$

where $|R_4(\varepsilon )|\lesssim \varepsilon ^{\frac{1}{2}-\mu }$. In particular, for $\varepsilon \le \varepsilon _0$ small enough we have from Lemma A.16 that

$$\begin{aligned} \begin{aligned} \left| {\mathcal {W}}_{m,\varepsilon }^+(0)\right| \ge 2\mu m|M_-(i\varepsilon )||M_+(1)|. \end{aligned} \end{aligned}$$

Therefore,

$$\begin{aligned} \begin{aligned} \left| {\mathcal {G}}_{m,\varepsilon }^-(y,0,z)-{\mathcal {G}}_{m,\varepsilon }^+(y,0,z)\right|&=\frac{2}{|{\mathcal {W}}_{m,\varepsilon }^+(0)|^2}\left| \textrm{Im}\left( \phi _{l,m,\varepsilon }^-(y,0)\phi _{u,m,\varepsilon }^-(z,0){\mathcal {W}}_{m,\varepsilon }^+(0)\right) \right| \\&\le \frac{2}{\mu m}\frac{\left| \phi _{u,m}(z)\phi _{u,m}(y)\textrm{Im}\left( M_+(-i\varepsilon )M_-(i\varepsilon )\right) \right| }{|M_-(i\varepsilon )|^2|M_+(1)|^2} + R_5(\varepsilon )\\&\lesssim \varepsilon ^{2\mu } + \varepsilon ^{\frac{1}{2}-\mu }, \end{aligned} \end{aligned}$$

and the lemma follows. $\square$

### Integral reduction for $\beta ^2=1/4$

The special case in which $\beta ^2=1/4$ is critical in the sense that the Hardy inequality (6.4) may saturate and thus the derivative bounds in Proposition 6.17 are no longer uniform in $\varepsilon >0$. Still, we are able to prove the following result.

#### Proposition 6.22

Let $y_0\le 0$ and $0<\varepsilon \le 1$. Then,

$$\begin{aligned} \frac{\varepsilon ^2}{1+\varepsilon ^2}\Vert \partial _y \psi _{m,\varepsilon }^\pm (\cdot ,y_0)\Vert _{L^2}^2 + m^2 \Vert \psi _{m,\varepsilon }^\pm (\cdot ,y_0)\Vert _{L^2}^2 \lesssim \Vert {\omega }_m^0 \Vert _{H^2}^2 + \Vert \rho _m^0 \Vert _{H^2}^2. \end{aligned}$$

Moreover,

$$\begin{aligned} \begin{aligned} \int _0^1\frac{\varepsilon (y-y_0)}{((y-y_0)^2+\varepsilon ^2)^2}|\varphi _{m,\varepsilon }^\pm |^2 \textrm{d}y \lesssim \Vert {\omega }_m^0 \Vert _{H^2}^2 + \Vert \rho _m^0 \Vert _{H^2}^2. \end{aligned} \end{aligned}$$

If we further assume that $|-y_0\pm i\varepsilon |\ge c_0$, for some $c_0>0$, then

$$\begin{aligned} \frac{c_0^2}{1+c_0^2}\Vert \partial _y \psi _{m,\varepsilon }^\pm (y,y_0)\Vert _{L^2}^2 + m^2 \Vert \psi _{m,\varepsilon }^\pm (y,y_0)\Vert _{L^2}^2 \lesssim \frac{1}{c_0^2}\Vert {\omega }_m^0 \Vert _{L^2}^2 + \frac{1}{c_0^4}\Vert \rho _m^0 \Vert _{L^2}^2. \end{aligned}$$

In particular, $c=-y_0\pm i\varepsilon$ belongs to the resolvent set of $L_m$.

#### Proof

The proof is similar to the one for Proposition 6.17. Here, since $\beta ^2=1/4$ we estimate

$$\begin{aligned} \frac{1}{4}\int _0^1\frac{|\psi _{m,\varepsilon }^\pm |^2}{|y-y_0\pm i\varepsilon |^2}\textrm{d}y \le \frac{1}{1+c_0^2}\int _0^1\frac{|\psi _{m,\varepsilon }^\pm |^2}{4y^2}\textrm{d}y \le \frac{1}{1+c_0^2}\int _0^1 |\partial _y\psi _{m,\varepsilon }|^2\textrm{d}y, \end{aligned}$$

which can be absorbed by $\int _0^1 |\partial _y\psi _{m,\varepsilon }|^2\textrm{d}y$, thus producing the desired $H^1$ estimates. $\square$

The estimate on the $L^2$ norm of the derivative degenerates as $\varepsilon$ becomes small. We may lose pointwise bounds on the solution, and for this reason we investigate more thoroughly the Green’s function ${\mathcal {G}}_{m,\varepsilon }^\pm (y,y_0,z)$ when $-1\ll y_0 \le 0$. In particular, we have that

#### Proposition 6.23

Let $y,z\in [0,1]$. There exists $\delta >0$ such that

$$\begin{aligned}  &   |{\mathcal {G}}_{m,\varepsilon }^+(y,y_0,z)|\lesssim |y-y_0+i\varepsilon |^{\frac{1}{2}}|z-y_0+i\varepsilon |^{\frac{1}{2}}\left( 1 + \big |\log m|y-y_0+i\varepsilon |\big |\right) \\  &   \quad \left( 1 + \big |\log m|z-y_0+i\varepsilon |\big |\right) \end{aligned}$$

and

$$\begin{aligned}  &   |\partial _y{\mathcal {G}}_{m,\varepsilon }^+(y,y_0,z)|\lesssim |y-y_0+i\varepsilon |^{-\frac{1}{2}}|z-y_0+i\varepsilon |^{\frac{1}{2}}\left( 1 + \big |\log m|y-y_0+i\varepsilon |\big |\right) \\  &   \quad \left( 1 + \big |\log m|z-y_0+i\varepsilon |\big |\right) \end{aligned}$$

for all $y_0<0$ and $\varepsilon >0$ with $|-y_0\pm i\varepsilon |\le \delta$.

We remark that the hidden implicit constant may depend on *m*, but for our purposes this is unimportant.

#### Proof

The proof follows the same steps as the one for Proposition 5.1. We shall obtain suitable estimates on the Wrosnkian. Now, since $y_0<0$, we recall

$$\begin{aligned} {\mathcal {W}}_{m,\varepsilon }^\pm (y_0):=\frac{2m}{\sqrt{\pi }}\Big (W_0(-y_0\pm i\varepsilon )M_0(1-y_0\pm i\varepsilon )-M_0(-y_0\pm i\varepsilon )W_0(1-y_0\pm i\varepsilon )\Big ). \end{aligned}$$

Using Lemma A.11 and Lemma A.12, there exists $C>0$ and $\delta >0$ such that

$$\begin{aligned} \left| \frac{W_0(1-y_0\pm i\varepsilon )}{M_0(1-y_0\pm i\varepsilon )}\right| \le C, \quad \left| \frac{M_0(-y_0\pm i\varepsilon )}{W_0(-y_0\pm i\varepsilon )}\right| \le \frac{1}{2C}, \end{aligned}$$

for all $|-y_0\pm i\varepsilon |\le \delta$. Hence, we can lower bound

$$\begin{aligned} \left| {\mathcal {W}}_{m,\varepsilon }^{\pm }(y_0)\right| \ge \frac{m}{\sqrt{\pi }}\left| W_0(-y_0\pm i\varepsilon )\right| \left| M_0(1-y_0\pm i\varepsilon )\right| \end{aligned}$$

and the proposition follows from the asymptotic expansions of the homogeneous solutions that conform the Green’s function. $\square$

With the above asymptotics at hand, we are now able to prove the following result.

#### Proposition 6.24

Let $\delta >0$ be given by Proposition 6.23 and let $y_0<0$ such that $|y_0|\le \frac{\delta }{2}$. We have that

$$\begin{aligned} \left\| \int _{-\frac{\delta }{2}}^0 \textrm{e}^{-imy_0t}\big (\textrm{e}^{m\varepsilon t}\psi _{m,\varepsilon }^-(y,y_0)-\textrm{e}^{-m\varepsilon t}\psi _{m,\varepsilon }^+(y,y_0) \big )\textrm{d}y_0\right\| _{L^2_y}\lesssim \varepsilon ^\frac{1}{2} \end{aligned}$$

and also

$$\begin{aligned} \left\| \int _{-\frac{\delta }{2}}^0 \textrm{e}^{-imy_0t}\big (\textrm{e}^{m\varepsilon t}\rho _{m,\varepsilon }^-(y,y_0)-\textrm{e}^{-m\varepsilon t}\rho _{m,\varepsilon }^+(y,y_0) \big )\textrm{d}y_0\right\| _{L^2_y}\lesssim \varepsilon ^\frac{1}{2}, \end{aligned}$$

for all $\varepsilon >0$ such that $|-y_0 +i\varepsilon |\le \delta$.

#### Proof

Following the same strategy as in the proof of Proposition 6.19, we see that $\varphi _{m,\varepsilon }(y,y_0)$ satisfies

$$\begin{aligned} m^2 \Vert \varphi _{m,\varepsilon }\Vert _{L^2}^2 \lesssim \varepsilon \Vert {\omega }_m^0\Vert _{H^2}^2 + \int _0^1 \frac{2\varepsilon (y-y_0)}{((y-y_0)^2 + \varepsilon ^2)^2}\left( |\varphi _{m,\varepsilon }^-|^2 + |\varphi _{m,\varepsilon }^+|^2\right) \textrm{d}y. \end{aligned}$$

In particular, using the asymptotic bounds from Proposition 6.23 we can estimate

$$\begin{aligned} \begin{aligned}&\int _0^1 \frac{2\varepsilon (y-y_0)}{((y-y_0)^2 + \varepsilon ^2)^2}\left( |\varphi _{m,\varepsilon }^-|^2 + |\varphi _{m,\varepsilon }^+|^2\right) \textrm{d}y \\&\quad \lesssim \int _0^1 \frac{\varepsilon (y-y_0)}{|y-y_0 + i\varepsilon |^3}\left( 1 + \left| \log |y-y_0+i\varepsilon |\right| \right) ^2 \textrm{d}y \\&\quad \lesssim \int _0^1 \frac{\varepsilon (y-y_0)}{|y-y_0 + i\varepsilon |^{\frac{7}{2}}} \textrm{d}y \\&\quad \lesssim \varepsilon \int _0^1 \frac{1}{(y-y_0)^\frac{5}{2}} \textrm{d}y \\&\quad \lesssim \varepsilon \left( 1 + (-y_0)^{-\frac{3}{2}}\right) . \end{aligned} \end{aligned}$$

We conclude the first part of the proof upon noting that

$$\begin{aligned} \int _{-\frac{\delta }{2}}^0 \left\| \varphi _{m,\varepsilon }(y,y_0) \right\| _{L^2_y}\textrm{d}y_0\lesssim \varepsilon ^\frac{1}{2}\Vert {\omega }_m^0\Vert _{H^2} + \varepsilon ^\frac{1}{2} \int _{-\frac{\delta }{2}}^0 \left( 1 + (-y_0)^{-\frac{3}{2}}\right) ^\frac{1}{2}\textrm{d}y_0 \lesssim \varepsilon ^\frac{1}{2}. \end{aligned}$$

For the second part of the proposition, from (2.10) we have

$$\begin{aligned} \rho _{m,\varepsilon }^-(y,y_0)-\rho _{m,\varepsilon }^+(y,y_0) = \frac{\varphi _{m,\varepsilon }(y,y_0)}{y-y_0-i\varepsilon } + \frac{2i\varepsilon }{(y-y_0)^2+\varepsilon ^2}\varphi _{m,\varepsilon }^+(y,y_0) \end{aligned}$$

and we write

$$\begin{aligned} \varphi _{m,\varepsilon }(y,y_0)=\int _0^1 {\mathcal {G}}_{m,\varepsilon }^-(y,y_0,z) \left( \frac{i\varepsilon (z-y_0)}{((z-y_0)^2 + \varepsilon ^2)^2}\varphi _{m,\varepsilon }^-(z,y_0)-8i\varepsilon {\Delta }_m{\omega }_m^0(z)\right) \textrm{d}z. \end{aligned}$$

In particular, using Proposition 6.22 and Proposition 6.23 we estimate

$$\begin{aligned} \begin{aligned}&\left| \int _0^1 {\mathcal {G}}_{m,\varepsilon }^-(y,y_0,z) \frac{i\varepsilon (z-y_0)}{((z-y_0)^2 + \varepsilon ^2)^2}\varphi _{m,\varepsilon }^-(z,y_0) \textrm{d}z \right| \\&\quad \lesssim \varepsilon ^\frac{1}{2} \left( \int _0^1 \frac{(z-y_0)}{((z-y_0)^2 + \varepsilon ^2)^2}|{\mathcal {G}}_{m,\varepsilon }^-(y,y_0,z)|^2\textrm{d}z\right) ^\frac{1}{2} \\&\quad \lesssim \varepsilon ^\frac{1}{2}|y-y_0-i\varepsilon |^\frac{1}{2} (1+ \left| \log |y-y_0-i\varepsilon |\right| ) \\&\qquad \left( \int _0^1 \frac{(z-y_0)}{|z-y_0-i\varepsilon |^3}(1+ \left| \log |z-y_0-i\varepsilon |\right| )^2 \textrm{d}z \right) ^\frac{1}{2} \\&\quad \lesssim \varepsilon ^\frac{1}{2}|y-y_0-i\varepsilon |^\frac{1}{2} (1+ \left| \log |y-y_0-i\varepsilon |\right| )\left( \int _0^1 \frac{1}{(z-y_0)^{2+\frac{1}{4}}} \textrm{d}z \right) ^\frac{1}{2} \\&\quad \lesssim \varepsilon ^\frac{1}{2}|y-y_0-i\varepsilon |^\frac{1}{2} (1+ \left| \log |y-y_0-i\varepsilon |\right| )\left( 1+(-y_0)^{-\frac{1}{2}-\frac{1}{8}} \right) . \end{aligned} \end{aligned}$$

With this pointwise bound, we obtain

$$\begin{aligned} \begin{aligned} \left\| \frac{\varphi _{m,\varepsilon }(y,y_0)}{y-y_0-i\varepsilon } \right\| _{L^2_y}&\lesssim \varepsilon ^\frac{1}{2}\left( 1+(-y_0)^{-\frac{1}{2}-\frac{1}{8}} \right) \left( \int _0^1 |y-y_0-i\varepsilon |^{-1} (1+ \left| \log |y-y_0-i\varepsilon |\right| )^2 \textrm{d}y\right) ^\frac{1}{2} \\&\lesssim \varepsilon ^\frac{1}{2}\left( 1+(-y_0)^{-\frac{1}{2}-\frac{1}{8}} \right) \left( \int _0^1 |y-y_0-i\varepsilon |^{-1-\frac{1}{4}} \textrm{d}y\right) ^\frac{1}{2} \\&\lesssim \varepsilon ^\frac{1}{2}\left( 1+(-y_0)^{-\frac{3}{4}} \right) \end{aligned} \end{aligned}$$

and thus

$$\begin{aligned} \int _{-\frac{\delta }{2}}^0 \left\| \frac{\varphi _{m,\varepsilon }(y,y_0)}{y-y_0-i\varepsilon } \right\| _{L^2_y} \textrm{d}y_0 \lesssim \varepsilon ^\frac{1}{2}. \end{aligned}$$

On the other hand, from the bounds obtained in Proposition 6.22, we have

$$\begin{aligned}  &   \int _{-\frac{\delta }{2}}^0 \left\| \frac{2i\varepsilon }{(y-y_0)^2+\varepsilon ^2}\varphi _{m,\varepsilon }^+(y,y_0) \right\| _{L^2_y} \textrm{d}y_0 \\  &   \quad \lesssim \varepsilon ^\frac{1}{2} \int _{-\frac{\delta }{2}}^0 \frac{1}{(-y_0)^\frac{1}{2}}\left\| \frac{\varepsilon ^\frac{1}{2} (y-y_0)^\frac{1}{2}}{(y-y_0)^2+\varepsilon ^2}\varphi _{m,\varepsilon }^+(y,y_0) \right\| _{L^2_y} \textrm{d}y_0\lesssim \varepsilon ^\frac{1}{2}, \end{aligned}$$

and the proof is concluded. $\square$

Similarly, the contribution from the resolvent integral along the vertical boundaries of the contour is also negligible.

#### Proposition 6.25

Let $y_0\in \left( -\beta /m, 0\right)$. We have that

$$\begin{aligned} \left\| \int _0^\varepsilon \textrm{e}^{-imy_0t}\big (\textrm{e}^{mst}\psi _{m,s}^-(y,y_0)+\textrm{e}^{-mst}\psi _{m,s}^+(y,y_0) \big )\textrm{d}s\right\| _{L^2_y}\lesssim \varepsilon \end{aligned}$$

and

$$\begin{aligned} \left\| \int _0^\varepsilon \textrm{e}^{-imy_0t}\big (\textrm{e}^{mst}\rho _{m,s}^-(y,y_0)+\textrm{e}^{-mst}\rho _{m,s}^+(y,y_0) \big )\textrm{d}s\right\| _{L^2_y}\lesssim \varepsilon ^\frac{1}{4}. \end{aligned}$$

#### Proof

The first part concerning the stream-functions $\psi _{m,\varepsilon }^\pm (y,y_0)$ is a direct consequence of the uniform $L^2$ bounds of $\psi _{m,\varepsilon }^\pm (y,y_0)$ obtained in Proposition 6.22. As for the density statement, we use (2.10); thanks to the asymptotic bounds from Proposition 6.23 we further observe that

$$\begin{aligned} \begin{aligned} \int _0^\varepsilon \left\| \frac{\varphi _{m,\varepsilon }^\pm (y,y_0)}{y-y_0\pm is}\right\| _{L^2_y}\textrm{d}s&\lesssim \int _0^\varepsilon \left( \int _0^1 |y-y_0\pm is|^{-1}(1+ \left| \log |y-y_0-i\varepsilon |\right| )^2 \textrm{d}y\right) ^\frac{1}{2} \textrm{d}s \\&\lesssim \int _0^\varepsilon \left( \int _0^1 |y-y_0\pm is|^{-\frac{3}{2}}\textrm{d}y\right) ^\frac{1}{2} \textrm{d}s \lesssim \int _0^\varepsilon |s|^{-\frac{3}{4}}\textrm{d}s. \end{aligned} \end{aligned}$$

With the above estimate, the bound follows swiftly. $\square$

We are now in position to prove Proposition 6.1 for the special case $\beta ^2=1/4$.

#### Proof of Proposition 6.1

Let $\delta >0$ be given by Proposition 6.23. For all $\varepsilon _*<\frac{\delta }{2}$, we introduce the rectangular region $R_*:=\big \lbrace c=y_0 + is\in {{\mathbb {C}}}: y_0\in \left[ -\delta /2,1+\delta /2\right] , \, s\in [-\varepsilon _*, \varepsilon _*] \big \rbrace$ and its associated decomposition into ${{\mathbb {R}}}_*^0$, $R_*^{ess}$ and $R_*^1$. From Proposition 6.24 and Proposition 6.25 we conclude that

$$\begin{aligned} \left\| \int _{R_*^0 \cup R_*^1}\textrm{e}^{-imct}\mathcal {R}(c,L_m)\textrm{d}c \right\| _{L^2_y}\lesssim \varepsilon ^\frac{1}{4}, \qquad \left\| \int _{\partial (\Omega \setminus R_*)}\textrm{e}^{-imct}\mathcal {R}(c,L_m)\textrm{d}c \right\| _{L^2_y}=0, \end{aligned}$$

because any $c\in \Omega \setminus R_*$ belongs to the resolvent set of the operator $L_m$. Indeed, any $c\in \Omega \setminus R_*$ is such that $\textrm{Re}(c)\in {{\mathbb {R}}}{\setminus } [0,1]$ and $|c|\ge \frac{\delta }{2}$, and we can see from Proposition 6.22 that $\mathcal {R}(c,L_m)$ is invertible. $\square$

Finally, in order to prove Theorem 4 for $\beta ^2=1/4$, we state and prove the following key Lemma, from which the Theorem easily follows.

#### Lemma 6.26

Let $y_0=0$ and $y,z\in [0,1]$. Then, there exists $\varepsilon _0>0$ such that

$$\begin{aligned} \sup _{y,z\in [0,1]}\left| {\mathcal {G}}_{m,\varepsilon }^-(y,0,z)-{\mathcal {G}}_{m,\varepsilon }^+(y,0,z)\right| \lesssim \frac{1}{\log \left( \frac{4}{\varepsilon }\right) } + \varepsilon ^\frac{1}{4}, \end{aligned}$$

for all $$\varepsilon \le \varepsilon _0$$.

#### Proof

We have ${\mathcal {G}}_{m,\varepsilon }^-(y,0,z)- {\mathcal {G}}_{m,\varepsilon }^+(y,0,z)=2i\textrm{Im}\left( {\mathcal {G}}_{m,\varepsilon }^-(y,0,z)\right)$ and for $y\le z$,

$$\begin{aligned} 2i\textrm{Im}\left( {\mathcal {G}}_{m,\varepsilon }^-(y,0,z)\right) =\frac{2i}{|{{\mathcal {W}}_{m,\varepsilon }^-}(0)|^2}\textrm{Im}\left( \phi _{l,m,\varepsilon }^-(y,0)\phi _{u,m,\varepsilon }^-(z,0){\mathcal {W}}_{m,\varepsilon }^+(0)\right) . \end{aligned}$$

Now, using Proposition 3.3, Lemma A.4 and Lemma A.11,

$$\begin{aligned} \begin{aligned}&{\phi _{l,m,\varepsilon }^-(y,0)}{{\mathcal {W}}_{m,\varepsilon }^+(0)} \\&\quad = \frac{2m}{\sqrt{\pi }}\Big ( |W_0(i\varepsilon )|^2M_0(y)M_0(1) + |M_0(i\varepsilon )|^2W_0(y)W_0(1)\Big . \\&\qquad - \Big .W_0(i\varepsilon )M_0(-i\varepsilon )W_0(y)M_0(1) - W_0(-i\varepsilon )M_0(i\varepsilon )M_0(y)W_0(1) +R_1(\varepsilon )\Big ), \end{aligned} \end{aligned}$$

where $$|R_1(\varepsilon )|\lesssim _{m,\mu } \varepsilon ^{\frac{1}{4}} |W_0(i\varepsilon )|^2$$. Similarly,

$$\begin{aligned} \begin{aligned} \phi _{u,m,\varepsilon }^-(z,0)&=W_0(1)M_0(z)-M_0(1)W_0(z) + R_2(\varepsilon ) =: \phi _{u,m}(z) + R_2(\varepsilon )\\ \end{aligned} \end{aligned}$$

with $$|R_2(\varepsilon )|\lesssim \varepsilon ^{\frac{1}{4}}$$. Thus,

$$\begin{aligned} \begin{aligned}&\phi _{l,m,\varepsilon }^-(y,0)\phi _{u,m,\varepsilon }^-(z,0){\mathcal {W}}_{m,\varepsilon }^+(0) \\&\quad =-\frac{2m}{\sqrt{\pi }} \phi _{u,m}(z) \Big ( W_0(-i\varepsilon )M_0(i\varepsilon )M_0(y)W_0(1) + W_0(i\varepsilon )M_0(-i\varepsilon )W_0(y)M_0(1) \Big ) \\&\qquad +\frac{2m}{\sqrt{\pi }} \phi _{u,m}(z)\Big ( |W_0(i\varepsilon )|^2M_0(y)M_0(1) + |M_0(i\varepsilon )|^2W_0(y)W_0(1)\Big ) + R_3(\varepsilon ), \end{aligned} \end{aligned}$$

where $|R_3(\varepsilon )|\lesssim m \varepsilon ^{\frac{1}{4}}|W_0(i\varepsilon )|^2$, uniformly in $y,z\in [0,1]$. In particular, since $M_0(y)\in {{\mathbb {R}}}$ and $W_0(y)\in {{\mathbb {R}}}$, for all $y\in [0,1]$, we have

$$\begin{aligned}  &   \textrm{Im}\left( \phi _{l,m,\varepsilon }^-(y,0)\phi _{u,m,\varepsilon }^-(z,0){\mathcal {W}}_{m,\varepsilon }^+(0)\right) =-\frac{2m}{\sqrt{\pi }} \phi _{u,m}(z)\phi _{u,m}(y)\textrm{Im}\left( W_0(-i\varepsilon )M_0(i\varepsilon )\right) \\  &   \quad + \textrm{Im}\left( R_3(\varepsilon )\right) . \end{aligned}$$

Due to symmetry, we also have

$$\begin{aligned}  &   \textrm{Im}\left( \phi _{l,m,\varepsilon }^-(z,0)\phi _{u,m,\varepsilon }^-(y,0){\mathcal {W}}_{m,\varepsilon }^+(0)\right) =-\frac{2m}{\sqrt{\pi }} \phi _{u,m}(z)\phi _{u,m}(y)\textrm{Im}\left( W_0(-i\varepsilon )M_0(i\varepsilon )\right) \\  &   \quad + \textrm{Im}\left( \widetilde{R}_3(\varepsilon )\right) . \end{aligned}$$

For the Wronskian, we have from (3.13) that

$$\begin{aligned} \begin{aligned} \left| {\mathcal {W}}_{m,\varepsilon }^+(0)\right| =\frac{2m}{\sqrt{\pi }}|W_0(i\varepsilon )||M_0(1)|\left| 1- \frac{M_0(i\varepsilon )}{W_0(i\varepsilon )}\frac{W_0(1)}{M_0(1)} + R_4(\varepsilon )\right| . \end{aligned} \end{aligned}$$

where $|R_4(\varepsilon )|\lesssim \varepsilon$. In particular, for $\varepsilon \le \varepsilon _0$ small enough we have from Lemma A.11 that

$$\begin{aligned} \begin{aligned} \left| {\mathcal {W}}_{m,\varepsilon }^+(0)\right| \ge \frac{m}{\sqrt{\pi }}|W_0(i\varepsilon )||M_0(1)|. \end{aligned} \end{aligned}$$

Therefore,

$$\begin{aligned} \begin{aligned} \left| {\mathcal {G}}_{m,\varepsilon }^-(y,0,z)-{\mathcal {G}}_{m,\varepsilon }^+(y,0,z)\right|&=\frac{2}{|{\mathcal {W}}_{m,\varepsilon }^+(0)|^2}\left| \textrm{Im}\left( \phi _{l,m,\varepsilon }^-(y,0)\phi _{u,m,\varepsilon }^-(z,0){\mathcal {W}}_{m,\varepsilon }^+(0)\right) \right| \\&\le \frac{4\sqrt{\pi }}{m}\frac{\left| \phi _{u,m}(z)\phi _{u,m}(y)\textrm{Im}\left( W_0(-i\varepsilon )M_0(i\varepsilon )\right) \right| }{|W_0(i\varepsilon )|^2|M_0(1)|^2} + R_5(\varepsilon )\\&\lesssim \left| \frac{M_0(i\varepsilon )}{W_0(i\varepsilon )}\right| + \varepsilon ^\frac{1}{4}, \end{aligned} \end{aligned}$$

and the conclusion follows from Lemma A.11. $\square$

## Bounds on Solutions to the Inhomogeneous Taylor–Goldstein Equation

This section provides bounds for solutions $\Phi _{m,\varepsilon }$ to the inhomogeneous Taylor–Goldstein equation (TGf) with boundary conditions $\Phi _{m,\varepsilon }(0,y_0)=\Phi _{m,\varepsilon }(1,y_0)=0$. The following lemma relates regions of the interval (0, 1) that are far away from a fixed $y_0\in [0,1]$ to nearby regions of $y_0$.

### Lemma 7.1

Let $y_0\in [0,1]$, $n\ge 1$ and $\Phi _{m,\varepsilon }$ be the solution to TGf). Then, we have that

$$\begin{aligned} \Vert \partial _y \Phi _{m,\varepsilon } \Vert _{L^2_y(J_3^c)}^2 + m^2 \Vert \Phi _{m,\varepsilon } \Vert _{L^2_y(J_3^c)}^2 \lesssim m^2 \Vert \Phi _{m,\varepsilon } \Vert _{L^2_y(J_2^c\cap J_3)}^2 + \frac{1}{m^2}\Vert f \Vert _{L^2_y(J_2^c)}^2. \end{aligned}$$

### Proof

For $y_n=y_0+\frac{n\beta }{m}$, the lemma follows from the energy inequality

$$\begin{aligned} \frac{1}{2}\int _{y_3}^1 \left[ |\partial _y \Phi _{m,\varepsilon }|^2 + m^2|\Phi _{m,\varepsilon }|^2 \right] \textrm{d}y \le \frac{m^2}{\beta ^2}\int _{y_2}^{y_3}|\Phi _{m,\varepsilon }|^2 \textrm{d}y + \int _{y_2}^1 |f||\Phi _{m,\varepsilon }|\textrm{d}y, \end{aligned}$$

and Young’s inequality to absorb the potential term. We omit the details. $\square$

With the above lemma we are in position to provide bounds on the solution to (TGf).

### Proposition 7.2

Let $\Phi _{m,\varepsilon }$ be the solution to TGf). Then

- If $m|y-y_0|\le 3\beta$ and $\beta ^2\ne 1/4$, then
  $$\begin{aligned} |y-y_0+i\varepsilon |^{-\frac{1}{2}+\mu } |\Phi _{m,\varepsilon }(y,y_0)|+ |y-y_0+i\varepsilon |^{\frac{1}{2}+\mu } |\partial _y \Phi _{m,\varepsilon }(y,y_0)|\lesssim \frac{1}{m^{1+\mu }}\Vert f \Vert _{L^2_y}. \end{aligned}$$
- If $m|y-y_0|\le 3\beta$ and $\beta ^2 = 1/4$, then
  $$\begin{aligned}  &   |y-y_0+i\varepsilon |^{-\frac{1}{2}} |\Phi _{m,\varepsilon }(y,y_0)|+ |y-y_0+i\varepsilon |^{\frac{1}{2}} |\partial _y \Phi _{m,\varepsilon }(y,y_0)|\\  &   \quad \lesssim \frac{1}{m} \left( 1 + \big | \log \left( m|y-y_0\pm i\varepsilon |\right) \big | \right) \Vert f \Vert _{L^2_y}. \end{aligned}$$
- If $$m|y-y_0|\ge 3\beta $$ then
  $$\begin{aligned} m\Vert \Phi _{m,\varepsilon }(y,y_0)\Vert _{L^2_y(J_3^c)}+\Vert \partial _y \Phi _{m,\varepsilon }(y,y_0)\Vert _{L^2_y(J_3^c)}\lesssim \frac{1}{m}\Vert f \Vert _{L^2_y} \end{aligned}$$
  and
  $$\begin{aligned} |\partial _y \Phi _{m,\varepsilon }(y,y_0)|\lesssim \Vert f \Vert _{L^2_y}. \end{aligned}$$

### Proof

The first part is a straightforward application of the bounds on the Green’s function from Theorem 2 and the Cauchy-Schwartz inequality, once we write $\Phi _{m,\varepsilon }(y,y_0)=\int _0^1 {\mathcal {G}}_{m,\varepsilon }^+(y,y_0,z)f(z,y_0)\textrm{d}z$. The second part of the proposition follows from the first part, which gives $m \Vert \Phi _{m,\varepsilon } \Vert _{L^2_y(J_2^c\cap J_3)}\lesssim \frac{1}{m}\Vert f \Vert _{L^2_y}$ and Lemma 7.1. For the pointwise bound, assume without loss of generality that $y_0+\frac{3\beta }{m}<y\le 1$. Then, let $y_3=y_0+\frac{3\beta }{m}$ and write

$$\begin{aligned}  &   \partial _y\Phi _{m,\varepsilon }(y,y_0)=\partial _y \Phi _{m,\varepsilon }(y_3,y_0) \\  &   \quad + \int _{y_3}^y \left[ \left( m^2-\beta ^2\frac{1}{(y'-y_0+i\varepsilon )^2}\right) \Phi _{m,\varepsilon }(y',y_0) + f(y')\right] \textrm{d}y'. \end{aligned}$$

Now, $|y_3-y_0|=\frac{3\beta }{m}$ so that we estimate $|\partial _y \Phi _{m,\varepsilon }(y_3,y_0)|\lesssim \frac{1}{m^{1+\mu }}\left| \frac{\beta }{m}\right| ^{-\frac{1}{2}-\mu }\lesssim m^{-\frac{1}{2}}$. Similarly, we use the second part of the proposition to estimate the remaining terms in $L^2_y(J_3^c)$ and obtain the desired conclusion. $\square$

## Boundary Terms Estimates

The purpose of this section is to obtain estimates on the boundary terms that appear in the expressions for $\partial _{y_0}\psi _{m,\varepsilon }^\pm (y,y_0)$ and other related derivatives. We begin by recording the following results, which will be used throughout the entire section.

### Proposition 8.1

Let $\beta ^2\ne 1/4$. There exists $\varepsilon _0>0$ such that for all $y,y_0\in [0,1]$ with $m|y-y_0|\le 3\beta$ there holds

$$\begin{aligned}  &   |y-y_0\pm i \varepsilon |^{-\frac{1}{2}-\mu }|\partial _z{\mathcal {G}}_{m,\varepsilon }^\pm (y,y_0,0)| + |y-y_0\pm i\varepsilon |^{\frac{1}{2}+\mu }|\partial _y\partial _z {\mathcal {G}}_{m,\varepsilon }^\pm (y,y_0,0)|\\  &   \quad \lesssim m^{\frac{1}{2}-\mu }\frac{1}{|M_-(y_0\mp i \varepsilon ))|}, \end{aligned}$$

and

$$\begin{aligned}  &   |y-y_0\pm i \varepsilon |^{-\frac{1}{2}-\mu }|\partial _z{\mathcal {G}}_{m,\varepsilon }^\pm (y,y_0,1)| + |y-y_0\pm i\varepsilon |^{\frac{1}{2}+\mu }|\partial _y\partial _z {\mathcal {G}}_{m,\varepsilon }^\pm (y,y_0,1)|\\  &   \quad \lesssim m^{\frac{1}{2}-\mu }\frac{1}{|M_-(1-y_0\pm i \varepsilon ))|}, \end{aligned}$$

for all $$0\le \varepsilon \le \varepsilon _0$$.

### Proof

For $z=0$, note that we have the explicit expression

$$\begin{aligned}  &   \partial _z{\mathcal {G}}_{m,\varepsilon }^\pm (y,y_0,0)\\  &   \quad =\frac{M_+(1-y_0\pm i\varepsilon )M_-(y-y_0\pm i\varepsilon )-M_-(1-y_0\pm i\varepsilon )M_+(y-y_0\pm i\varepsilon )}{M_+(1-y_0\pm i\varepsilon )M_-(-y_0\pm i\varepsilon )-M_-(1-y_0\pm i\varepsilon )M_+(-y_0\pm i\varepsilon )}, \end{aligned}$$

so that

$$\begin{aligned}  &   \partial _y \partial _z{\mathcal {G}}_{m,\varepsilon }^\pm (y,y_0,0)\\  &   \quad =2m \frac{M_+(1-y_0\pm i\varepsilon )M_-'(y-y_0\pm i\varepsilon )-M_-(1-y_0\pm i\varepsilon )M_+'(y-y_0\pm i\varepsilon )}{M_+(1-y_0\pm i\varepsilon )M_-(-y_0\pm i\varepsilon )-M_-(1-y_0\pm i\varepsilon )M_+(-y_0\pm i\varepsilon )}. \end{aligned}$$

If $m|y-y_0|\le 3\beta$, we use the bounds on the Wronskian from Proposition 4.1. For $\beta ^2>1/4$, the conclusion is straightforward. For $\beta ^2<1/4$, we take a closer look to the Wronskian estimates obtained on the proof of Proposition 4.3. The bounds are a consequence of Lemma A.5, A.15-A.17. The argument for $z=1$ is similar, we omit the details. $\square$

### Proposition 8.2

Let $\beta ^2= 1/4$. There exists $\varepsilon _0>0$ such that for all $y,y_0\in [0,1]$ with $m|y-y_0|\le 3\beta$ there holds

$$\begin{aligned}  &   |y-y_0\pm i \varepsilon |^{-\frac{1}{2}}|\partial _z{\mathcal {G}}_{m,\varepsilon }^\pm (y,y_0,0)| + |y-y_0\pm i\varepsilon |^{\frac{1}{2}}|\partial _y\partial _z {\mathcal {G}}_{m,\varepsilon }^\pm (y,y_0,0)|\\  &   \quad \lesssim m^{\frac{1}{2}}\frac{1+ \big | \log \left( m|y-y_0\pm i\varepsilon |\right) \big |}{|M_0(y_0 \mp i \varepsilon ))|}, \end{aligned}$$

and

$$\begin{aligned}  &   |y-y_0\pm i \varepsilon |^{-\frac{1}{2}}|\partial _z{\mathcal {G}}_{m,\varepsilon }^\pm (y,y_0,1)| + |y-y_0\pm i\varepsilon |^{\frac{1}{2}}|\partial _y\partial _z {\mathcal {G}}_{m,\varepsilon }^\pm (y,y_0,1)|\\  &   \quad \lesssim m^{\frac{1}{2}}\frac{1+ \big | \log \left( m|y-y_0\pm i\varepsilon | \right) \big |}{|M_0(1-y_0\mp i \varepsilon ))|}, \end{aligned}$$

for all $$0\le \varepsilon \le \varepsilon _0$$.

### Proof

Since $m|y-y_0|\le 3\beta$, the proof follows the same ideas to show Proposition 5.1, with the help of Lemma A.5, A.10-A.12, we omit the details. $\square$

### Estimates for first order boundary terms

This subsection is devoted to obtain estimates on

$$\begin{aligned} {\mathcal {B}}_{m,\varepsilon }^\pm (y,y_0,z)=\partial _z{\mathcal {G}}_{m,\varepsilon }^\pm (y,y_0,z)\partial _z\varphi _{m,\varepsilon }^\pm (z,y_0) \end{aligned}$$

for $z=0$ and $z=1$ under the assumption that $m|y-y_0|\le 3\beta$. In what follows, we shall argue for $z=0$, the statements and proofs for $z=1$ are similar and we thus omit them. We begin by providing bounds for $\partial _z\varphi _{m,\varepsilon }^\pm (0,y_0)$.

#### Proposition 8.3

Let $y_0\in [0,1]$, we have the following.

- If $$my_0\le 3\beta $$, then $$ |\partial _y\varphi _{m,\varepsilon }^\pm (0,y_0)|\lesssim m^{-\frac{1}{2}} Q_{0,m}. $$
- If $$my_0\ge 3\beta $$, then $$ |\partial _y\varphi _{m,\varepsilon }^\pm (0,y_0)|\lesssim Q_{0,m} $$.

For the proof, we assume that $y_0<1/2$. Otherwise, the proposition follows from Proposition 7.2. Note that from (2.8) and (2.12), there holds

$$\begin{aligned} \partial _y\varphi _{m,\varepsilon }^\pm (0,y_0)&= \int _0^1 \partial _y{\mathcal {G}}_{m,\varepsilon }^\pm (0,y_0,z) F_{m,\varepsilon }^\pm (z,y_0) \textrm{d}z \\&= \int _0^1 \partial _y{\mathcal {G}}_{m,\varepsilon }^\pm (0,y_0,z)\left( F_{m}(z)+ \frac{y_0\mp i\varepsilon }{\beta ^2}{\Delta }_m{\omega }_m^0(z)\right) \textrm{d}z, \end{aligned}$$

Further observe that, due to (H) we have

$$\begin{aligned} \begin{aligned} \int _0^1 \partial _y{\mathcal {G}}_{m,\varepsilon }^\pm (0,y_0,z) F_{m}^\pm (z,0) \textrm{d}z&= -4(\mu +i\nu )m \int _0^1 \frac{\phi _{u,m,\varepsilon }^\pm (z,y_0)}{{\mathcal {W}}_{m,\varepsilon }^\pm (y_0)}F_{m}(z,0)\textrm{d}z \\&= -4(\mu + i \nu )m\int _0^1 \frac{\phi _{u,m,\varepsilon }^\pm (z,y_0)- \phi _{u,m}(z)}{{\mathcal {W}}_{m,\varepsilon }^\pm (y_0)}F_{m}(z,0)\textrm{d}z \end{aligned} \end{aligned}$$

and we define

$$\begin{aligned} f_{m,\varepsilon }^\pm (z,y_0):=\phi _{u,m,\varepsilon }^\pm (z,y_0) - \phi _{u,m}(z). \end{aligned}$$

#### Estimates on $f_{m,\varepsilon }^\pm$ for $\beta ^2\ne 1/4$

From the explicit formulas (3.4) and (3.7), we have

$$\begin{aligned} \begin{aligned} f_{m,\varepsilon }^\pm (z,y_0)&=M_+(1-y_0\pm i\varepsilon )M_-(z-y_0\pm i\varepsilon ) - M_+(1)M_-(z) \\&\quad - M_-(1-y_0\pm i\varepsilon )M_+(z-y_0\pm i\varepsilon ) + M_-(1)M_+(z) \end{aligned} \end{aligned}$$

and we can obtain the next result.

##### Proposition 8.4

Let $z,y_0\in [0,1]$ such that $my_0\le 3\beta$ and $mz\le 6\beta$. Let $0\le \varepsilon \le \min \left( \frac{\beta }{m},\frac{1}{2m}\right)$. Then,

$$\begin{aligned} |f_{m,\varepsilon }(z,y_0)|\lesssim m^{\frac{1}{2}-\mu }|y_0\pm i\varepsilon |^{\frac{1}{2}-\mu }|M_+(1-y_0\pm i\varepsilon )|. \end{aligned}$$

In particular, $$\Vert f_{m,\varepsilon }\Vert _{L^2_y(J)}\lesssim m^{-\mu }|y_0\pm i\varepsilon |^{\frac{1}{2}-\mu }|M_+(1-y_0\pm i\varepsilon )|$$.

##### Proof

We shall assume $\beta ^2<1/4$, the case $\beta ^2>1/4$ is analogous and easier. We write

$$\begin{aligned} \begin{aligned}&M_+(1-y_0\pm i\varepsilon )M_-(z-y_0\pm i\varepsilon ) - M_+(1)M_-(z) \\&\quad =M_+(1-y_0\pm i\varepsilon )\Big (M_-(z-y_0\pm i\varepsilon ) - M_-(z)\Big ) \\&\qquad + M_-(z)\Big ( M_+(1-y_0\pm i\varepsilon ) - M_+(1)\Big ) \end{aligned} \end{aligned}$$

and

$$\begin{aligned} \begin{aligned}&M_-(1-y_0\pm i\varepsilon )M_+(z-y_0\pm i\varepsilon ) - M_-(1)M_+(z)\\&\quad =M_-(1-y_0\pm i\varepsilon )\Big (M_+(z-y_0\pm i\varepsilon ) - M_+(z)\Big ) \\&\qquad + M_+(z)\Big ( M_-(1-y_0\pm i\varepsilon ) - M_-(1)\Big ). \end{aligned} \end{aligned}$$

Firstly, we estimate

$$\begin{aligned}  &   M_+(1-y_0\pm i\varepsilon ) - M_+(1)=\int _0^1 \frac{\textrm{d}}{\textrm{d}s}M_+(1+s(-y_0\pm i\varepsilon ))\textrm{d}s = (-y_0\pm i\varepsilon )\\  &   \quad \int _0^1 M_+'(1+s(-y_0\pm i\varepsilon ))\textrm{d}s \end{aligned}$$

and we divide our argument as follows. Let $N_{\mu ,0}$ be given as in Lemma A.15.

For $m\le N_{\mu ,0}$, we use Lemma A.3 and the fact that $y_0\le 1/2$ to bound

$$\begin{aligned} \begin{aligned} |M_+(1-y_0\pm i\varepsilon ) - M_+(1)|&\lesssim m^{\frac{1}{2}+\mu }|y_0\pm i\varepsilon |\int _0^1\frac{\textrm{d}s}{|1+s(-y_0\pm i\varepsilon )|^{\frac{1}{2} - \mu }} \\&\lesssim m^{\frac{1}{2}+\mu }|y_0\pm i\varepsilon | \\&\lesssim |y_0\pm i\varepsilon ||M_+(1-y_0 \pm i\varepsilon )|. \end{aligned} \end{aligned}$$

In the last inequality, we have used Lemma A.5, A.17 and A.15. Similarly,

$$\begin{aligned} |M_-(1-y_0\pm i\varepsilon ) - M_-(1)|\lesssim |y_0\pm i\varepsilon ||M_-(1-y_0 \pm i\varepsilon )|\lesssim |y_0\pm i\varepsilon ||M_+(1-y_0 \pm i\varepsilon )|, \end{aligned}$$

where we have used Lemma A.5 and Lemma A.17 to deduce $|M_-(1-y_0 \pm i\varepsilon )|\lesssim |M_+(1-y_0 \pm i\varepsilon )|$.

For $m\ge N_{\mu ,0}$, we claim that

$$\begin{aligned} \left| \frac{M_+'(1+s(-y_0\pm i\varepsilon ))}{M_+(1-y_0\pm i\varepsilon )} \right| \lesssim 1. \end{aligned}$$

Indeed, this follows from

$$\begin{aligned} \left| \frac{M_+'(1+s(-y_0\pm i\varepsilon ))}{M_+(1-y_0\pm i\varepsilon )} \right| = \left| \frac{M_+'(1+s(-y_0\pm i\varepsilon ))}{M_+(1+s(-y_0\pm i\varepsilon ))}\right| \left| \frac{M_+(1+s(-y_0\pm i\varepsilon )) }{M_+(1-y_0\pm i\varepsilon )} \right| \end{aligned}$$

and the corresponding bounds from Lemma A.6 since $2\,m(1-y_0)\ge m\ge N_{\mu ,0}$. Hence, we have

$$\begin{aligned} \begin{aligned} |M_+(1-y_0\pm i\varepsilon ) - M_+(1)|&\le 2m|y_0\pm i\varepsilon |\int _0^1 \left| M_+'(1+s(-y_0\pm i\varepsilon ))\right| \textrm{d}s \\&\lesssim m|y_0\pm i\varepsilon |\left| M_+(1-y_0\pm i\varepsilon ) \right| . \\ \end{aligned} \end{aligned}$$

Similarly, we also have

$$\begin{aligned}  &   |M_-(1-y_0\pm i\varepsilon ) - M_-(1)|\lesssim m|y_0\pm i\varepsilon |\left| M_-(1-y_0\pm i\varepsilon ) \right| \\  &   \quad \lesssim m|y_0\pm i\varepsilon |\left| M_+(1-y_0\pm i\varepsilon ) \right| , \end{aligned}$$

where we have used Lemma A.15 to deduce $\left| M_-(1-y_0\pm i\varepsilon ) \right| \lesssim \left| M_+(1-y_0\pm i\varepsilon ) \right|$. We next turn our attention to the bounds for $M_-(z-y_0\pm i\varepsilon ) - M_-(z)$. As before, we consider two cases.

$\bullet$ **Case 1.** For $2y_0\le z$ we estimate

$$\begin{aligned}  &   M_-(z-y_0\pm i\varepsilon ) - M_-(z)=\int _0^1 \frac{\textrm{d}}{\textrm{d}s}M_-(z+s(-y_0\pm i\varepsilon ))\textrm{d}s = (-y_0\pm i\varepsilon )\\  &   \quad \int _0^1 M_-'(z+s(-y_0\pm i\varepsilon ))\textrm{d}s. \end{aligned}$$

From Lemma A.3, $M_-'(\zeta )\lesssim \zeta ^{-\frac{1}{2}-\mu } m^{\frac{1}{2}-\mu }$, and since $2y_0\le z$, we have that $s|y_0\pm i\varepsilon |\le |z+s(-y_0\pm i\varepsilon )|$, for all $s\in (0,1)$. Thus,

$$\begin{aligned} \begin{aligned} |M_-(z-y_0\pm i\varepsilon ) - M_-(z)|&\lesssim m^{\frac{1}{2}-\mu }|y_0\pm i\varepsilon |\int _0^1\frac{\textrm{d}s}{|z+s(-y_0\pm i\varepsilon )|^{\frac{1}{2} + \mu }} \\&\lesssim m^{\frac{1}{2}-\mu }|y_0\pm i\varepsilon |\int _0^1\frac{\textrm{d}s}{|s(y_0\pm i\varepsilon )|^{\frac{1}{2} + \mu }} \\&\lesssim m^{\frac{1}{2}-\mu }|y_0\pm i\varepsilon |^{\frac{1}{2}-\mu }. \end{aligned} \end{aligned}$$

$\bullet$ **Case 2.** For $z\le 2y_0$, we directly estimate using Lemma A.3, that is,

$$\begin{aligned} \begin{aligned}&|M_-(z-y_0\pm i\varepsilon ) - M_-(z)|\le |M_-(z-y_0\pm i\varepsilon )| + |M_-(z)|\\&\quad \lesssim m^{\frac{1}{2} -\mu } \left( |z-y_0\pm i\varepsilon |^{\frac{1}{2}-\mu } + |z|^{\frac{1}{2}-\mu }\right) \\&\quad \lesssim m^{\frac{1}{2} -\mu }|y_0\pm i\varepsilon |^{\frac{1}{2}-\mu }. \end{aligned} \end{aligned}$$

$$\square $$

From this localised estimates, we are able to obtain bounds on $f_{m,\varepsilon }(z,y_0)$ for $mz\ge 6\beta$. For this, we first deduce useful estimates on $\phi _{u,m}^\pm (z)$.

##### Lemma 8.5

The function $\phi _{u,m}(z)=M_+(1)M_-(z) - M_-(1)M_+(z)$ satisfies

$$\begin{aligned} {\Delta }_m \phi _{u,m}^\pm (z) + \beta ^2 \frac{\phi _{u,m}^\pm (z)}{z^2}=0, \qquad \phi _{u,m}^\pm (1)=0. \end{aligned}$$

For $J_6=\lbrace z\in [0,1]: mz\le 6\beta \rbrace$ and $J_6^c=[0,1]\setminus J_6$, it is such that

$$\begin{aligned} \Vert \phi _{u,m}\Vert _{L^\infty (J_6)}\lesssim m^{\frac{1}{2}-\mu }|z|^{\frac{1}{2}-\mu }|M_+(1)|, \quad \Vert \phi _{u,m}\Vert _{L^2_y(J_6)}\lesssim m^{-\frac{1}{2}}|M_+(1)| \end{aligned}$$

and

$$\begin{aligned} \Vert \partial _z\phi _{u,m}\Vert _{L^2_y(J_6^c)} + m\Vert \phi _{u,m}\Vert _{L^2_y(J_6^c)} \lesssim m^{\frac{1}{2}}|M_+(1)|. \end{aligned}$$

$$\square $$

##### Proof

The statements for $\Vert \phi _{u,m}\Vert _{L^\infty (J_6)}$ and $\Vert \phi _{u,m}\Vert _{L^2_y(J_6)}$ follow from the asymptotic expansions for small argument given by Lemma A.3. The integral estimates follow from the $\Vert \phi _{u,m}\Vert _{L^2_y(J_6)}$ bounds using Lemma 7.1. $\square$

The following proposition obtains $L^2$ bounds on $f_{m,\varepsilon }^\pm (\cdot ,y_0)$ from the localized bounds of Proposition 8.4 and the above lemma.

##### Proposition 8.6

We have that

$$\begin{aligned} \Vert f_{m,\varepsilon }^\pm (\cdot , y_0)\Vert _{L^2}\lesssim m^{-\frac{1}{2}}(m|y_0-i\varepsilon |)^{\frac{1}{2}-\mu }|M_+(1-y_0+i\varepsilon )|. \end{aligned}$$

##### Proof

It is straightforward to see that $f_{m,\varepsilon }^\pm (z,y_0)$ solves

$$\begin{aligned} {\Delta }_m f_{m,\varepsilon }^\pm + \beta ^2 \frac{f_{m,\varepsilon }^\pm }{(z-y_0\pm i\varepsilon )^2}=\beta ^2(-y_0\pm i\varepsilon )\left( \frac{2}{z(z-y_0\pm i\varepsilon )^2} + \frac{-y_0\pm i\varepsilon }{z^2(z-y_0\pm i\varepsilon )^2}\right) \phi _{u,m} \end{aligned}$$

and $f_{m,\varepsilon }^\pm (1,y_0)=0$. Hence, using the same strategy from Lemma 4.4, we have that

8.1 $$\begin{aligned} \begin{aligned}&\frac{1}{2}\int _{\frac{6\beta }{m}}^1 |\partial _z f_{m,\varepsilon }^\pm |^2 + m^2 |f_{m,\varepsilon }^\pm |^2\textrm{d}z \le \frac{m^2}{\beta ^2}\int _{\frac{5\beta }{m}}^{\frac{6\beta }{m}}|f^\pm _{m,\varepsilon }|^2\textrm{d}z \\&\quad +\beta ^2|-y_0\pm i\varepsilon |\int _{\frac{5\beta }{m}}^{1}\left( \frac{2}{z} + \frac{|-y_0\pm i\varepsilon |}{z^2}\right) \frac{|\phi _{u,m}(z)|f_{m,\varepsilon }^\pm (z)|}{|z-y_0\pm i\varepsilon |^2}\textrm{d}z. \end{aligned} \nonumber \\ \end{aligned}$$

Now, from Proposition 8.4, we have

$$\begin{aligned} \frac{m^2}{\beta ^2}\int _{\frac{5\beta }{m}}^{\frac{6\beta }{m}}|f^\pm _{m,\varepsilon }|^2\textrm{d}z \lesssim \frac{m}{\beta } \left( m|y_0-i\varepsilon |\right) ^{1-2\mu }|M_+(1-y_0+i\varepsilon )|^2, \end{aligned}$$

while we write

$$\begin{aligned}  &   \beta ^2|-y_0\pm i\varepsilon |^2\int _{\frac{5\beta }{m}}^{1}\frac{|\phi _{u,m}(z)|f_{m,\varepsilon }^\pm (z)|}{z^2|z-y_0\pm i\varepsilon |^2}\textrm{d}z = \beta ^2|-y_0\pm i\varepsilon |^2\\  &   \quad \left( \int _{\frac{5\beta }{m}}^{\frac{6\beta }{m}} + \int _{\frac{6\beta }{m}}^1 \right) \frac{|\phi _{u,m}(z)|f_{m,\varepsilon }^\pm (z)|}{z^2|z-y_0\pm i\varepsilon |^2}\textrm{d}z. \end{aligned}$$

For example, with the bounds of Proposition 8.4 and Lemma 8.5, and the fact that $z\ge \frac{5\beta }{m}$ and $y_0\le \frac{3\beta }{m}$, we have $|z-y_0\pm i\varepsilon |^{-2}\lesssim m^2$ and

$$\begin{aligned} \begin{aligned}&\beta ^2|-y_0\pm i\varepsilon |^2 \int _{\frac{5\beta }{m}}^{\frac{6\beta }{m}}\frac{|\phi _{u,m}(z)|f_{m,\varepsilon }^\pm (z)|}{z^2|z-y_0\pm i\varepsilon |^2}\textrm{d}z\\&\quad \lesssim {m^2}|y_0-i\varepsilon |^2\Vert f_{m,\varepsilon }^\pm \Vert _{L^\infty (J)}|M_+(1)|^2 \int _{y_2}^{y_2+\frac{\beta }{m}}m^{\frac{1}{2}-\mu }z^{-\frac{3}{2}-\mu } \textrm{d}z \\&\quad \lesssim m^{\frac{7}{2} -\mu }|y_0\pm i\varepsilon |^{\frac{5}{2}-\mu }|M_+(1)||M_+(1-y_0\pm i\varepsilon )|. \end{aligned} \end{aligned}$$

On the other hand, Young’s inequality and Lemma 8.5 gives

$$\begin{aligned} \begin{aligned}&\beta ^2|-y_0\pm i\varepsilon |^2\int _{\frac{6\beta }{m}}^1\frac{|\phi _{u,m}(z)|f_{m,\varepsilon }^\pm (z)|}{z^2|z-y_0\pm i\varepsilon |^2}\textrm{d}z \\&\quad \le \frac{m^2}{8}\int _{y_2+\frac{\beta }{m}}^1|f_{m,\varepsilon }^\pm (z)|^2 \textrm{d}z + C m^5|y_0-i\varepsilon |^4 {|M_+(1)|^2}, \end{aligned} \end{aligned}$$

for some $C>0$ large enough. Similarly, we bound

$$\begin{aligned} \begin{aligned}&\beta ^2|-y_0\pm i\varepsilon |\int _{\frac{5\beta }{m}}^{\frac{6\beta }{m}}\frac{|\phi _{u,m}(z)|f_{m,\varepsilon }^\pm (z)|}{z|z-y_0\pm i\varepsilon |^2}\textrm{d}z \\&\quad \lesssim m^{\frac{5}{2} -\mu }|y_0\pm i\varepsilon |^{\frac{3}{2}-\mu }|M_+(1)||M_+(1-y_0\pm i\varepsilon )| \end{aligned} \end{aligned}$$

and

$$\begin{aligned} \begin{aligned}&\beta ^2|-y_0\pm i\varepsilon |\int _{\frac{6\beta }{m}}^1\frac{|\phi _{u,m}(z)|f_{m,\varepsilon }^\pm (z)|}{z|z-y_0\pm i\varepsilon |^2}\textrm{d}z \\&\quad \le \frac{m^2}{8}\int _{\frac{6\beta }{m}}^1|f_{m,\varepsilon }^\pm (z)|^2 \textrm{d}z + C m^3|y_0-i\varepsilon |^2 {|M_+(1)|^2}, \end{aligned} \end{aligned}$$

for some $C>0$ large enough. Hence, we absorb the potential term on the left hand side of (8.1) and conclude that

$$\begin{aligned} \frac{1}{4}\int _{y_2+\frac{\beta }{m}}^1 |\partial _z f_{m,\varepsilon }^\pm |^2 + m^2 |f_{m,\varepsilon }^\pm |^2\textrm{d}z \lesssim m(m|y_0-i\varepsilon |)^{1-2\mu }|M_+(1-y_0+i\varepsilon )|^2. \end{aligned}$$

and the lemma follows. $\square$

#### Estimates on $f_{m,\varepsilon }^\pm$ for $\beta ^2=1/4$

From the explicit formulas (3.11) and (3.14), we now have

$$\begin{aligned} \begin{aligned} f_{m,\varepsilon }^\pm (z,y_0)&=W_0(1-y_0\pm i\varepsilon )M_0(z-y_0\pm i\varepsilon ) - W_0(1)M_0(z) \\&\quad - M_0(1-y_0\pm i\varepsilon )W_0(z-y_0\pm i\varepsilon ) + M_0(1)W_0(z) \end{aligned} \end{aligned}$$

from which we obtain the following result.

##### Proposition 8.7

Let $z,y_0\in [0,1]$ such that $my_0\le 3\beta$ and $mz\le 6\beta$. Let $0\le \varepsilon \le \min \left( \frac{\beta }{m},\frac{1}{2m}\right)$. Then,

$$\begin{aligned} |f_{m,\varepsilon }(z,y_0)|\lesssim (m|y_0\pm i\varepsilon |)^\frac{1}{2} \left( 1 + \big | \log \left( my_0 \right) \big | \right) |M_0(1-y_0\pm i\varepsilon )|. \end{aligned}$$

In particular, $$\Vert f_{m,\varepsilon }\Vert _{L^2_y(J)}\lesssim |y_0\pm i\varepsilon |^{\frac{1}{2}} \left( 1 + \big | \log \left( m|y_0\pm i\varepsilon |\right) \big | \right) |M_0(1-y_0\pm i\varepsilon )|$$.

##### Proof

We write

$$\begin{aligned} \begin{aligned}&W_0(1-y_0\pm i\varepsilon )M_0(z-y_0\pm i\varepsilon ) - W_0(1)M_0(z)\\&\quad = W_0(1-y_0\pm i\varepsilon )\Big (M_0(z-y_0\pm i\varepsilon ) - M_0(z)\Big ) \\&\qquad + M_0(z)\Big ( W_0(1-y_0\pm i\varepsilon ) - W_0(1)\Big ) \end{aligned} \end{aligned}$$

We shall now estimate the differences involving the Whittaker function $W_0$, the estimates for the differences involving $M_0$ follow similarly as for the case $\beta ^2\ne 1/4$ and they are

$$\begin{aligned}  &   \left| M_0(z-y_0\pm i\varepsilon ) - M_0(z)\right| \lesssim m^\frac{1}{2}|y_0\pm i\varepsilon |^\frac{1}{2}, \quad \left| M_0(1-y_0\pm i\varepsilon ) - M_0(1)\right| \\  &   \quad \lesssim m|y_0\pm i\varepsilon ||M_0(1-y_0\pm i\varepsilon )|. \end{aligned}$$

Firstly, we estimate

$$\begin{aligned}  &   W_0(1-y_0\pm i\varepsilon ) - W_0(1)=\int _0^1 \frac{\textrm{d}}{\textrm{d}s}W_0(1+s(-y_0\pm i\varepsilon ))\textrm{d}s = 2m(-y_0\pm i\varepsilon )\\  &   \quad \int _0^1 W_0'(1+s(-y_0\pm i\varepsilon ))\textrm{d}s \end{aligned}$$

and we divide our argument as follows. Let $N_{\mu ,0}$ be given as in Lemma A.15.

For $m\le N_{\mu ,0}$, we use Lemma A.4 and the fact that $y_0\le \frac{1}{2}$ to bound

$$\begin{aligned} \begin{aligned} |W_0(1-y_0\pm i\varepsilon ) - W_0(1)|&\lesssim m^{\frac{1}{2}}|y_0\pm i\varepsilon |\int _0^1\frac{1+ \big |\log m|1+s(-y_0\pm i\varepsilon )|\big |}{|1+s(-y_0\pm i\varepsilon )|^{\frac{1}{2} }} \textrm{d}s \\&\lesssim m^{\frac{1}{2}}|y_0\pm i\varepsilon | \\&\lesssim |y_0\pm i\varepsilon ||M_0(1-y_0 \pm i\varepsilon )|. \end{aligned} \end{aligned}$$

In the last inequality, we have used Lemma A.5, A.17 and A.15.

For $m\ge N_{\mu ,0}$, we claim that

$$\begin{aligned} \left| \frac{W_0'(1+s(-y_0\pm i\varepsilon ))}{M_0(1-y_0\pm i\varepsilon )} \right| \lesssim 1. \end{aligned}$$

Indeed, this follows from

$$\begin{aligned}  &   \left| \frac{W_0'(1+s(-y_0\pm i\varepsilon ))}{M_0(1-y_0\pm i\varepsilon )} \right| = \left| \frac{W_0'(1+s(-y_0\pm i\varepsilon ))}{W_0(1+s(-y_0\pm i\varepsilon ))}\right| \left| \frac{W_0(1+s(-y_0\pm i\varepsilon )) }{W_0(1-y_0\pm i\varepsilon )} \right| \\  &   \quad \left| \frac{W_0(1-y_0\pm i\varepsilon ) }{M_0(1-y_0\pm i\varepsilon )} \right| \end{aligned}$$

and the corresponding bounds from Lemma A.6 since $2\,m(1-y_0)\ge m\ge N_{\mu ,0}$. Hence, we have

$$\begin{aligned} \begin{aligned} |W_0(1-y_0\pm i\varepsilon ) - W_0(1)|&\le 2m|y_0\pm i\varepsilon |\int _0^1 \left| W_0'(1+s(-y_0\pm i\varepsilon ))\right| \textrm{d}s \\&\lesssim m|y_0\pm i\varepsilon |\left| M_0(1-y_0\pm i\varepsilon ) \right| . \\ \end{aligned} \end{aligned}$$

We next turn our attention to the bounds for $W_0(z-y_0\pm i\varepsilon ) - W_0(z)$. As before, we consider two cases.

$\bullet$ **Case 1.** For $2y_0\le z$ we estimate

$$\begin{aligned}  &   W_0(z-y_0\pm i\varepsilon ) - W_0(z)=\int _0^1 \frac{\textrm{d}}{\textrm{d}s}W_0(z+s(-y_0\pm i\varepsilon ))\textrm{d}s = (-y_0\pm i\varepsilon )\\  &   \quad \int _0^1 W_0'(z+s(-y_0\pm i\varepsilon ))\textrm{d}s. \end{aligned}$$

From Lemma A.4, $W_0'(\zeta )\lesssim m^\frac{1}{2}\zeta ^{-\frac{1}{2}}\left( 1+ \big | \log \left( m|\zeta |\right) \big |\right)$, and since $2y_0\le z$, we have that $s|y_0\pm i\varepsilon |\le |z+s(-y_0\pm i\varepsilon )|$, for all $s\in (0,1)$. Thus,

$$\begin{aligned} \begin{aligned} |W_0(z-y_0\pm i\varepsilon ) - W_0(z)|&\lesssim m^{\frac{1}{2}}|y_0\pm i\varepsilon |\int _0^1\frac{1+\big | \log \left( m|z+s(-y_0\pm i\varepsilon )|\right) \big |}{|z+s(-y_0\pm i\varepsilon )|^{\frac{1}{2}}}\textrm{d}s \\&\lesssim m^{\frac{1}{2}}|y_0\pm i\varepsilon |^\frac{1}{2}\int _0^1\frac{1+\big | \log \left( ms|y_0\pm i\varepsilon |\right) \big |}{s^{\frac{1}{2}}}\textrm{d}s \\&\lesssim (m|y_0\pm i\varepsilon |)^{\frac{1}{2}}\left( 1 + \big | \log \left( m|y_0\pm i\varepsilon |\right) \big |\right) . \end{aligned} \end{aligned}$$

$\bullet$ **Case 2.** For $z\le 2y_0$, we directly estimate using Lemma A.4, that is,

$$\begin{aligned} \begin{aligned} |M_-(z-y_0\pm i\varepsilon ) - M_-(z)|&\le |M_-(z-y_0\pm i\varepsilon )| + |M_-(z)| \\&\lesssim m^\frac{1}{2}|z-y_0\pm i\varepsilon |^\frac{1}{2} \left( 1 + \big | \log \left( m|z-y_0\pm i\varepsilon |\right) \big |\right) \\&\quad + m^\frac{1}{2}z^\frac{1}{2} \left( 1 + \big | \log \left( mz\right) \big |\right) \\&\lesssim (m|y_0\pm i\varepsilon |)^\frac{1}{2} \left( 1 + \big | \log \left( m|y_0\pm i\varepsilon |\right) \big | \right) . \end{aligned} \end{aligned}$$

$$\square $$

From this localised estimates, we are able to obtain bounds on $f_{m,\varepsilon }(z,y_0)$ for $mz\ge 6\beta$. For this, we first deduce useful estimates on $\phi _{u,m}^\pm (z)$.

##### Lemma 8.8

The function $\phi _{u,m}(z)=W_0(1)M_0(z) - M_0(1)W_0(z)$ satisfies

$$\begin{aligned} {\Delta }_m \phi _{u,m}^\pm (z) + \beta ^2 \frac{\phi _{u,m}^\pm (z)}{z^2}=0, \qquad \phi _{u,m}^\pm (1)=0. \end{aligned}$$

For $J_6=\lbrace z\in [0,1]: mz\le 6\beta \rbrace$ and $J_6^c=[0,1]\setminus J_6$, it is such that

$$\begin{aligned} \Vert \phi _{u,m}\Vert _{L^\infty (J)}\lesssim (mz)^\frac{1}{2} \left( 1 + \big | \log \left( mz\right) \big | \right) |M_0(1)|, \quad \Vert \phi _{u,m}\Vert _{L^2_y(J)}\lesssim m^{-\frac{1}{2}}|M_0(1)| \end{aligned}$$

and

$$\begin{aligned} \Vert \partial _z\phi _{u,m}\Vert _{L^2_y(J^c)} + m\Vert \phi _{u,m}\Vert _{L^2_y(J^c)} \lesssim m^{\frac{1}{2}}|M_+(1)| \end{aligned}$$

##### Proof

The statement for $\Vert \phi _{u,m}\Vert _{L^\infty (J_6)}$ follows from the asymptotic expansions for small argument given by Lemma A.3. For the integral estimates estimate, note that the change of variables $u=mz$ provides

$$\begin{aligned} \Vert \phi _{u,m}\Vert _{L^2_y(J_6)}^2&\lesssim \int _0^{\frac{6\beta }{m}}(mz)\left( 1 + \big | \log \left( mz\right) \big | \right) ^2 |M_0(1)|^2 \textrm{d}z \\&= \frac{|M_0(1)|^2}{m}\int _0^{6\beta } \eta \left( 1 + | \log \left( \eta \right) | \right) ^2 \textrm{d}\eta \\&\lesssim \frac{|M_0(1)|^2}{m}. \end{aligned}$$

The result follows using Lemma 7.1. $\square$

The following proposition obtains $L^2(0,1)$ bounds on $f_{m,\varepsilon }^\pm (\cdot ,y_0)$ from the localized bounds of Proposition 8.7 and the above Lemma. We omit its proof due to its similarity to the one for Proposition 8.6.

##### Proposition 8.9

We have that

$$\begin{aligned} \Vert f_{m,\varepsilon }^\pm (\cdot , y_0)\Vert _{L^2(0,1)}\lesssim m^{-\frac{1}{2}}(m|y_0-i\varepsilon |)^{\frac{1}{2}}\left( 1 + \big | \log \left( m|y_0\pm i\varepsilon |\right) \big |\right) |M_0(1-y_0\pm i\varepsilon )| \end{aligned}$$

We are now able to compare $\Vert f_{m,\varepsilon }^\pm (\cdot , y_0)\Vert _{L^2(0,1)}$ and the Wronskian $|{\mathcal {W}}_{m,\varepsilon }^\pm (y_0)|$.

##### Lemma 8.10

Let $y_0\in [0,1]$ such that $my_0\le 3\beta$. There exists $\varepsilon _0>0$ such that

$$\begin{aligned} \frac{\Vert f_{m,\varepsilon }^\pm (\cdot , y_0)\Vert _{L^2(0,1)}}{|{\mathcal {W}}_{m,\varepsilon }^\pm (y_0)|}\lesssim m^{-\frac{3}{2}}. \end{aligned}$$

##### Proof

Let $N_0>0$ be given by Lemma A.10, $\delta _1>0$ be given by Lemma A.11 and $\delta _2>0$ be given by Lemma A.13. From Lemma 5.2, there holds the following,

$\bullet$ **Case 1.** For $m\le N_0$ and $2\,m|y_0\pm i\varepsilon |\le \delta _1$, we have

$$\begin{aligned} |{\mathcal {W}}_{m,\varepsilon }^\pm (y_0)|\gtrsim m|M_0(1-y_0\pm i\varepsilon )||W_0(y_0\mp i\varepsilon )|. \end{aligned}$$

where further

$$\begin{aligned} \left| \frac{M_0(y_0\pm i\varepsilon )}{W_0(y_0\pm i\varepsilon )}\right| \le \frac{1}{2\sqrt{\pi }}. \end{aligned}$$

Now, from Lemma A.13, if $\delta _1\le \delta _2$, then,

$$\begin{aligned} |2m(y_0\pm i\varepsilon )|^\frac{1}{2}\left( 1 + \big | \log \left( 2m|y_0\pm i\varepsilon |\right) \big | \right) \lesssim |W_0(y_0\pm i\varepsilon )|, \end{aligned}$$

and the conclusion follows. On the other hand, for $\delta _2\le 2\,m|y_0\pm i\varepsilon | \le \delta _1$, we have that

$$\begin{aligned} m\frac{\Vert f_{m,\varepsilon }^\pm (\cdot , y_0)\Vert _{L^2}}{|{\mathcal {W}}_{m,\varepsilon }^\pm (y_0)|}\lesssim m^{-\frac{1}{2}}\frac{(m|y_0\pm i\varepsilon |)^\frac{1}{2}}{M_0(y_0\pm i\varepsilon )}\lesssim m^{-\frac{1}{2}}, \end{aligned}$$

due to Lemma A.14.

$\bullet$ **Case 2.** For $m\le N_0$ and $\delta _1\le 2my_0\le N_0$, we have now

$$\begin{aligned} |{\mathcal {W}}_{m,\varepsilon }^\pm (y_0)|\gtrsim m|M_0(1-y_0\pm i\varepsilon )||M_0(y_0\mp i\varepsilon )|, \end{aligned}$$

the conclusion follows using Lemma A.14 and the fact that $\left( 1 + \big | \log |2\,m(y_0\pm i\varepsilon )| \big | \right) \lesssim 1$.

$\bullet$ **Case 3.** For $m\ge N_0$, and $2\,m|y_0\pm i\varepsilon |\le \delta _1$, we have

$$\begin{aligned} |{\mathcal {W}}_{m,\varepsilon }^\pm (y_0)|\gtrsim m|M_0(1-y_0\pm i\varepsilon )||W_0(y_0\mp i\varepsilon )|. \end{aligned}$$

and also

$$\begin{aligned} \left| \frac{M_0(y_0\pm i\varepsilon )}{W_0(y_0\pm i\varepsilon )}\right| \le \frac{1}{2\sqrt{\pi }}, \end{aligned}$$

we proceed as in Case 1, we omit the details.

$\bullet$ **Case 4.** For $m\ge N_0$ and $\delta _1 \le 2my_0\le N_0$, we have

$$\begin{aligned} |{\mathcal {W}}_{m,\varepsilon }^\pm (y_0)|\gtrsim m|M_0(1-y_0\pm i\varepsilon )||M_0(y_0\mp i\varepsilon )|, \end{aligned}$$

we proceed as in Case 2, we omit the details. $\square$

We are now in position to prove Proposition 8.3.

##### Proof of Proposition 8.3

For $my_0\ge 3\beta$ we appeal to Proposition 7.2 to obtain the desired bound. On the other hand, for $my_0\le 3\beta$ let us recall that we can write

$$\begin{aligned} \begin{aligned} \partial _y\varphi _{m,\varepsilon }^\pm (0,y_0)&= \int _0^1 \partial _y{\mathcal {G}}_{m,\varepsilon }^\pm (0,y_0,z)\left( F_{m}(z)+ \frac{y_0\mp i\varepsilon }{\beta ^2}{\Delta }_m{\omega }_m^0(z)\right) \textrm{d}z \end{aligned} \end{aligned}$$

For $\beta ^2\ne 1/4$, it is straightforward to see from Proposition 7.2 that

$$\begin{aligned} \left| \frac{y_0\mp i\varepsilon }{\beta ^2}\int _0^1 \partial _y{\mathcal {G}}_{m,\varepsilon }^\pm (0,y_0,z){\Delta }_m{\omega }_m^0(z) \textrm{d}z\right| \lesssim \frac{1}{m^{1+\mu }}|y_0\pm i\varepsilon |^{\frac{1}{2}-\mu }\Vert {\omega }_m^0\Vert _{H^2_y}, \end{aligned}$$

while, thanks to Proposition 8.6, the lower bounds on the Wronskian from Proposition 4.3 and Lemma A.16 we bound

$$\begin{aligned} \begin{aligned} \left| \int _0^1 \partial _y{\mathcal {G}}_{m,\varepsilon }^\pm (0,y_0,z) F_{m}^\pm (z,0) \textrm{d}z\right|&= \left| 4(\mu +i\nu )m \int _0^1 \frac{f_{m,\varepsilon }^\pm (z,y_0)}{{\mathcal {W}}_{m,\varepsilon }^\pm (y_0)}F_{m}^\pm (z,0)\textrm{d}z\right| \\&\lesssim m \frac{\Vert f_{m,\varepsilon }^\pm (\cdot ,y_0)\Vert _{L^2_z}}{|{\mathcal {W}}_{m,\varepsilon }^\pm (y_0)|}\Vert F_{m}^\pm (z,0)\Vert _{L^2_z} \\&\lesssim m^{-\frac{1}{2}}\Vert F_m \Vert _{L^2}. \end{aligned} \end{aligned}$$

Similarly, for $\beta ^2=1/4$, using again Proposition 7.2,

$$\begin{aligned}&\left| \frac{y_0\mp i\varepsilon }{\beta ^2}\int _0^1 \partial _y{\mathcal {G}}_{m,\varepsilon }^\pm (0,y_0,z){\Delta }_m{\omega }_m^0(z) \textrm{d}z\right| \\&\quad \lesssim \frac{1}{m}|y_0\pm i\varepsilon |^{\frac{1}{2}}\left( 1 + \big | \log \left( m|y_0\pm i\varepsilon |\right) \big | \right) \Vert {\omega }_m^0\Vert _{H^2_y}\\&\quad \lesssim m^{-\frac{3}{2}}\Vert {\omega }_m^0\Vert _{H^2_y}, \end{aligned}$$

while, thanks to Lemma 8.10 we have

$$\begin{aligned} \begin{aligned} \left| \int _0^1 \partial _y{\mathcal {G}}_{m,\varepsilon }^\pm (0,y_0,z) F_{m}^\pm (z,0) \textrm{d}z\right|&= \left| 4(\mu +i\nu )m \int _0^1 \frac{f_{m,\varepsilon }^\pm (z,y_0)}{{\mathcal {W}}_{m,\varepsilon }^\pm (y_0)}F_{m}^\pm (z,0)\textrm{d}z\right| \\&\lesssim m \frac{\Vert f_{m,\varepsilon }^\pm (\cdot ,y_0)\Vert _{L^2_z}}{|{\mathcal {W}}_{m,\varepsilon }^\pm (y_0)|}\Vert F_{m}^\pm (z,0)\Vert _{L^2_z} \\&\lesssim m^{-\frac{1}{2}}\ \Vert F_m \Vert _{L^2}. \end{aligned} \end{aligned}$$

With this, the proof is finished. $\square$

We next provide pointwise localized bounds on ${\mathcal {B}}_{m,\varepsilon }^\pm (y,y_0,0)$.

##### Proposition 8.11

Let $\beta ^2\ne 1/4$ and $0\le \varepsilon \le \varepsilon _0$. Let $y,y_0\in [0,1]$ such that $m|y-y_0|\le 3\beta$. Then,

- If $my_0\le 3\beta$, we have
  $$\begin{aligned} \left| {\mathcal {B}}_{m,\varepsilon }^\pm (y,y_0,0)\right| \lesssim m^{-\frac{1}{2}}y_0^{-\frac{1}{2}+\mu }|y-y_0\pm i\varepsilon |^{\frac{1}{2}-\mu }{Q_{0,m}}. \end{aligned}$$
- If $my_0\ge 3\beta$, we have
  $$\begin{aligned} \left| {\mathcal {B}}_{m,\varepsilon }^\pm (y,y_0,0)\right| \lesssim (m|y-y_0\pm i\varepsilon |)^{\frac{1}{2}-\mu }{Q_{0,m}}. \end{aligned}$$

##### Proposition 8.12

Let $\beta ^2=1/4$ and $0\le \varepsilon \le \varepsilon _0$. Let $y,y_0\in [0,1]$ such that $m|y-y_0|\le 3\beta$. Then,

- If $my_0\le 3\beta$, we have
  $$\begin{aligned} \left| {\mathcal {B}}_{m,\varepsilon }^\pm (y,y_0,0)\right| \lesssim m^{-\frac{1}{2}}y_0^{-\frac{1}{2}}|y-y_0\pm i\varepsilon |^{\frac{1}{2}}\left( 1 + \big | \log \left( m|y-y_0\pm i\varepsilon |\right) \big | \right) {Q_{0,m}}. \end{aligned}$$
- If $my_0\ge 3\beta$, we have
  $$\begin{aligned} \left| {\mathcal {B}}_{m,\varepsilon }^\pm (y,y_0,0)\right| \lesssim (m|y-y_0\pm i\varepsilon |)^{\frac{1}{2}} \left( 1 + \big | \log \left( m|y-y_0\pm i\varepsilon |\right) \big | \right) {Q_{0,m}}. \end{aligned}$$

With the above pointwise bounds, one deduces the following integral estimates for all $\beta ^2>0$.

##### Corollary 8.13

Let $$y_0\in [0,1]$$. Then,

- If $my_0\le 3\beta$, we have
  $$\begin{aligned} \Vert {\mathcal {B}}_{m,\varepsilon }^\pm (\cdot ,y_0,0)\Vert _{L^2_y(J_2^c\cap J_3)} \lesssim m^{-\frac{3}{2}}y_0^{-\frac{1}{2}}{Q_{0,m}}. \end{aligned}$$
- If $my_0\ge 3\beta$, we have
  $$\begin{aligned} \Vert {\mathcal {B}}_{m,\varepsilon }^\pm (\cdot ,y_0,0)\Vert _{L^2_y(J_2^c\cap J_3)}\lesssim m^{-\frac{1}{2}}{Q_{0,m}}. \end{aligned}$$

The two propositions are a consequence of Propositions 8.1, 8.2, the lower bounds from Lemma A.9, A.14, A.18 and the pointwise estimates on $\partial _y\varphi _{m,\varepsilon }^\pm (0,y_0)$ from Proposition 8.3.

### Boundary pointwise estimates on Green’s Function’s derivatives

This subsection estimates derivatives of the Green’s function ${\mathcal {G}}_{m,\varepsilon }^\pm (y,y_0,z)$ evaluated at the boundary values $y,z\in \lbrace 0,1\rbrace$.

#### Lemma 8.14

We have that for $my_0\ge 3\beta$,

$$\begin{aligned} |\partial _y\partial _z{\mathcal {G}}_{m,\varepsilon }^{\pm }(0,y_0,0)|\lesssim m \end{aligned}$$

while for $$my_0\le 3\beta $$,

$$\begin{aligned} |\partial _y\partial _z{\mathcal {G}}_{m,\varepsilon }^{\pm }(0,y_0,0)|\lesssim \frac{1}{y_0}. \end{aligned}$$

#### Proof

For $\beta ^2>1/4$, it follows from the proof of Proposition 4.1 and Lemma A.9. For $\beta ^2=1/4$, it follows from Lemma 5.2, Lemma A.13 and Lemma A.14. For $b^2<1/4$, it follows from the proof of Proposition 4.3 and Lemma A.18. $\square$

#### Lemma 8.15

For $m\ge 6\beta$, we have

- If $my_0\le 3\beta$, then $m(1-y_0)\ge 3\beta$ and
  $$\begin{aligned} |\partial _y\partial _z{\mathcal {G}}_{m,\varepsilon }^\pm (1,y_0,0)|\lesssim {m^{\frac{1}{2}+\mu }}{y_0^{-\frac{1}{2}+\mu }}. \end{aligned}$$
- If $m(1-y_0)\le 3\beta$, then $my_0\ge 3\beta$ and
  $$\begin{aligned} |\partial _y\partial _z{\mathcal {G}}_{m,\varepsilon }^\pm (1,y_0,0)|\lesssim {m^{\frac{1}{2}+\mu }}{(1-y_0)^{-\frac{1}{2}+\mu }}. \end{aligned}$$
- If $my_0\ge 3\beta$ and $m(1-y_0)\ge 3\beta$, then
  $$\begin{aligned} |\partial _y\partial _z{\mathcal {G}}_{m,\varepsilon }^\pm (1,y_0,0)|\lesssim m. \end{aligned}$$

On the other hand, for $m\le 6\beta$, we have that

- If $$y_0\le \frac{1}{2}$$, then
  $$\begin{aligned} |\partial _y\partial _z{\mathcal {G}}_{m,\varepsilon }^\pm (1,y_0,0)|\lesssim {y_0^{-\frac{1}{2}+\mu }}. \end{aligned}$$
- If $$1-y_0\le \frac{1}{2}$$, then
  $$\begin{aligned} |\partial _y\partial _z{\mathcal {G}}_{m,\varepsilon }^\pm (1,y_0,0)|\lesssim {(1-y_0)^{-\frac{1}{2}+\mu }}. \end{aligned}$$

#### Proof

It is straightforward the lower bounds on the Wronskian and Lemmas A.9, A.18, A.13, for $\beta ^2>1/4$, $\beta ^2=1/4$ and $\beta ^2<1/4$, respectively. $\square$

#### Lemma 8.16

The same bounds as in Lemma 8.15 hold for $|\partial _y\partial _z{\mathcal {G}}_{m,\varepsilon }^\pm (0,y_0,1)|$.

#### Lemma 8.17

We have that for $m(1-y_0)\ge 3\beta$,

$$\begin{aligned} |\partial _y\partial _z{\mathcal {G}}_{m,\varepsilon }^{\pm }(1,y_0,1)|\lesssim m \end{aligned}$$

while for $$m(1-y_0)\le 3\beta $$,

$$\begin{aligned} |\partial _y\partial _z{\mathcal {G}}_{m,\varepsilon }^{\pm }(1,y_0,1)|\lesssim \frac{1}{1-y_0}. \end{aligned}$$

### Estimates for second order boundary terms

In what follows, we shall consider only the case $m\ge 6\beta$, since the setting $m\le 6\beta$ is analogous and easier. With the pointwise derivatives bounds obtained in the four previous lemmas, we are now in position to estimate

$$\begin{aligned} \begin{aligned} \left( \partial _y + \partial _{y_0}\right) ^2\varphi _{m,\varepsilon }^\pm (y,y_0)&=-\frac{2}{\beta ^2}\partial _y{\omega }_m^0(y) -F_{m,\varepsilon }^\pm (y,y_0) +2\partial _y\Big . {\mathcal {B}}_{m,\varepsilon }^\pm (y,y_0,z)\Big ]_{z=0}^{z=1} \\&\quad +2 \int _0^1\partial _y{\mathcal {G}}_{m,\varepsilon }^\pm (y,y_0,z)\left( \partial _zF_{m,\varepsilon }^\pm (z,y_0)+\partial _{y_0}F_{m,\varepsilon }^\pm (z,y_0)\right) \textrm{d}z \end{aligned} \end{aligned}$$

for both $y=0$ and $y=1$. For simplicity we only discuss the case $y=0$; the results and proofs are the same for the case $y=1$.

#### Proposition 8.18

Let $m\ge 6\beta$ and $y_0\in [0,1]$. Then, we have that

- For $$my_0\le 3\beta $$ and $$\beta ^2\ne 1/4$$,
  $$\begin{aligned} |\left( \partial _y + \partial _{y_0}\right) ^2\varphi _{m,\varepsilon }^\pm (y,y_0)|\lesssim \left( 1+\frac{1}{m^{1+\mu }}y_0^{-\frac{1}{2}-\mu } +m^{-\frac{1}{2}}y_0^{-1} + m^\frac{1}{2}y_0^{-\frac{1}{2}}\right) Q_{1,m} \end{aligned}$$
- For $$my_0\le 3\beta $$ and $$\beta ^2=1/4$$,
  $$\begin{aligned} |\left( \partial _y + \partial _{y_0}\right) ^2\varphi _{m,\varepsilon }^\pm (y,y_0)|\lesssim \left( 1+\frac{1}{m}y_0^{-\frac{1}{2}}\left( 1+ \big | \log \left( my_0\right) \big | \right) +m^{-\frac{1}{2}}y_0^{-1} + m^\frac{1}{2}y_0^{-\frac{1}{2}}\right) Q_{1,m} \end{aligned}$$
- For $$my_0\ge 3\beta $$ and $$m(1-y_0)\le 3\beta $$,
  $$\begin{aligned} |\left( \partial _y + \partial _{y_0}\right) ^2\varphi _{m,\varepsilon }^\pm (y,y_0)|\lesssim \left( m + (1-y_0)^{-\frac{1}{2}}\right) Q_{1,m}, \end{aligned}$$
- For $$my_0\ge 3\beta $$ and $$m(1-y_0)\ge 3\beta $$,
  $$\begin{aligned} |\left( \partial _y + \partial _{y_0}\right) ^2\varphi _{m,\varepsilon }^\pm (y,y_0)|\lesssim mQ_{1,m}. \end{aligned}$$

#### Proof

For $y=0$, we can estimate

$$\begin{aligned} |\partial _y{\omega }_m^0(0)|+ |F_{m,\varepsilon }^\pm (0,y_0)|\lesssim Q_{1,m} \end{aligned}$$

thanks to the Sobolev embedding. On the other hand, from Proposition 7.2, for $my_0\le 3\beta$ and $\beta ^2\ne 1/4$ we have

$$\begin{aligned} \left| \int _0^1\partial _y{\mathcal {G}}_{m,\varepsilon }^\pm (0,y_0,z)\left( \partial _zF_{m,\varepsilon }^\pm (z,y_0)+\partial _{y_0}F_{m,\varepsilon }^\pm (z,y_0)\right) \textrm{d}z\right| \lesssim \frac{1}{m^{1+\mu }}|y_0\pm i\varepsilon |^{-\frac{1}{2}-\mu }Q_{1,m}, \end{aligned}$$

while for $my_0\le 3\beta$ and $\beta ^2=1/4$ we have

$$\begin{aligned}&\left| \int _0^1\partial _y{\mathcal {G}}_{m,\varepsilon }^\pm (0,y_0,z) \left( \partial _z+\partial _{y_0}\right) F_{m,\varepsilon }^\pm (z,y_0)\textrm{d}z\right| \\  &\quad \lesssim \frac{1}{m}|y_0\pm i\varepsilon |^{-\frac{1}{2}} \left( 1 + \big | \log \left( m|y_0\pm i\varepsilon |\right) \big | \right) Q_{1,m}, \end{aligned}$$

whereas for $my_0\ge 3\beta$, we have

$$\begin{aligned} \left| \int _0^1\partial _y{\mathcal {G}}_{m,\varepsilon }^\pm (0,y_0,z)\left( \partial _z+\partial _{y_0}\right) F_{m,\varepsilon }^\pm (z,y_0)\textrm{d}z\right| \lesssim Q_{1,m}. \end{aligned}$$

Now, for the solid boundary terms $\partial _y\Big . {\mathcal {B}}_{m,\varepsilon }^\pm (y,y_0,z)\Big ]_{z=0}^{z=1}$, we shall use Proposition 8.3 as well as Lemmas 8.14-8.17. Indeed, for $\partial _y{\mathcal {B}}_{m,\varepsilon }^\pm (0,y_0,0)=\partial _y\partial _z{\mathcal {G}}_{m,\varepsilon }^\pm (0,y_0,0)\partial _y\varphi _{m,\varepsilon }^\pm (0,y_0)$, Lemma 8.14 provides

- For $my_0\le 3\beta$, we have that $|\partial _y{\mathcal {B}}_{m,\varepsilon }^\pm (0,y_0,0)|\lesssim m^{-\frac{1}{2}}y_0^{-1}Q_{0,m}$.
- For $my_0\ge 3\beta$, we have that $|\partial _y{\mathcal {B}}_{m,\varepsilon }^\pm (0,y_0,0)|\lesssim mQ_{0,m}$.

Similarly, for $\partial _y{\mathcal {B}}_{m,\varepsilon }^\pm (0,y_0,1)=\partial _y\partial _z{\mathcal {G}}_{m,\varepsilon }^\pm (0,y_0,1)\partial _y\varphi _{m,\varepsilon }^\pm (1,y_0)$, we have from Lemma 8.16 that

- For $$my_0\le 3\beta $$, then $$|\partial _y{\mathcal {B}}_{m,\varepsilon }^\pm (0,y_0,1)|\lesssim m^{\frac{1}{2}+\mu }y_0^{-\frac{1}{2}+\mu }Q_{0,m}$$.
- For $my_0\ge 3\beta$, we further distinguish
  - For $$m(1-y_0)\le 3\beta $$, then $$|\partial _y{\mathcal {B}}_{m,\varepsilon }^\pm (0,y_0,1)|\lesssim m^\mu (1-y_0)^{-\frac{1}{2}+\mu }Q_{0,m}$$.
  - For $$m(1-y_0)\ge 3\beta $$, then $$|\partial _y{\mathcal {B}}_{m,\varepsilon }^\pm (0,y_0,1)|\lesssim mQ_{0,m}$$.

As a result, for $my_0\le 3\beta$ and $\beta ^2\ne 1/4$ we have that

$$\begin{aligned} \begin{aligned}&|\left( \partial _y + \partial _{y_0}\right) ^2\varphi _{m,\varepsilon }^\pm (y,y_0)|\\&\quad \lesssim Q_{1,m}+\frac{1}{m^{1+\mu }}y_0^{-\frac{1}{2}-\mu }Q_{1,m} + m^{-\frac{1}{2}}y_0^{-1}Q_{0,m} + m^{\frac{1}{2}+\mu }y_0^{-\frac{1}{2}+\mu }Q_{0,m} \\&\quad \lesssim \left( 1+\frac{1}{m^{1+\mu }}y_0^{-\frac{1}{2}-\mu } +m^{-\frac{1}{2}}y_0^{-1} + m^\frac{1}{2}y_0^{-\frac{1}{2}}\right) Q_{1,m}, \end{aligned} \end{aligned}$$

while for $my_0\le 3\beta$ and $\beta ^2=1/4$ we have that

$$\begin{aligned} \begin{aligned}&|\left( \partial _y + \partial _{y_0}\right) ^2\varphi _{m,\varepsilon }^\pm (y,y_0)|\\&\quad \lesssim Q_{1,m}+\frac{1}{m}y_0^{-\frac{1}{2}} \left( 1 + \big | \log \left( my_0\right) \big | \right) Q_{1,m} + m^{-\frac{1}{2}}y_0^{-1}Q_{0,m} + m^{\frac{1}{2}}y_0^{-\frac{1}{2}}Q_{0,m} \\&\quad \lesssim \left( 1+\frac{1}{m}y_0^{-\frac{1}{2}} \left( 1 + \big | \log \left( my_0\right) \big | \right) +m^{-\frac{1}{2}}y_0^{-1} + m^\frac{1}{2}y_0^{-\frac{1}{2}}\right) Q_{1,m}. \end{aligned} \end{aligned}$$

Similarly, for $my_0\ge 3\beta$ and $m(1-y_0)\le 3\beta$ we conclude

$$\begin{aligned} \begin{aligned} |\left( \partial _y + \partial _{y_0}\right) ^2\varphi _{m,\varepsilon }^\pm (y,y_0)|&\lesssim Q_{1,m} + m Q_{0,m} + (1-y_0)^{-\frac{1}{2}}Q_{0,m} \lesssim \left( m+(1-y_0)^{-\frac{1}{2}}\right) Q_{1,m}, \end{aligned} \end{aligned}$$

whereas for $my_0\ge 3\beta$ and $m(1-y_0)\ge 3\beta$ we obtain

$$\begin{aligned} \begin{aligned} |\left( \partial _y + \partial _{y_0}\right) ^2\varphi _{m,\varepsilon }^\pm (y,y_0)|&\lesssim Q_{1,m} + m Q_{0,m} \lesssim mQ_{1,m} \end{aligned} \end{aligned}$$

and the proof is finished. $\square$

We next present estimates for

$$\begin{aligned} \widetilde{{{\mathcal {B}}_{m,\varepsilon }^\pm }}(y,y_0,z)=\partial _z{\mathcal {G}}_{m,\varepsilon }^\pm (y,y_0,z)\left( \partial _y + \partial _{y_0}\right) ^2\varphi _{m,\varepsilon }^\pm (y,y_0), \end{aligned}$$

at $z=0$. As before, we only obtain these bounds under the assumption that $m|y-y_0|\le 3\beta$. We state them for $z=0$; the result for $z=1$ is the same and thus we omit the details. The next two Propositions are a direct consequence of Propositions 8.1, 8.2, 8.18, as well as Lemma A.9A.13, A.14, A.18, depending on $\beta ^2$.

#### Proposition 8.19

Let $\beta ^2\ne 1/4$ and $y,y_0\in [0,1]$ such that $m|y-y_0|\le 3\beta$. Then,

- For $$my_0\le 3\beta $$,
  $$\begin{aligned} |\widetilde{{{\mathcal {B}}_{m,\varepsilon }^\pm }}(y,y_0,0)|\lesssim m^{-\frac{1}{2}}y_0^{-\frac{3}{2}}(m|y-y_0\pm i\varepsilon |)^{\frac{1}{2}-\mu }Q_{1,m} \end{aligned}$$
- For $$my_0\ge 3\beta $$ and $$m(1-y_0)\le 3\beta $$,
  $$\begin{aligned} |\widetilde{{{\mathcal {B}}_{m,\varepsilon }^\pm }}(y,y_0,0)|\lesssim \left( m + (1-y_0)^{-\frac{1}{2}}\right) (m|y-y_0\pm i\varepsilon |)^{\frac{1}{2}-\mu }Q_{1,m}, \end{aligned}$$
- For $$my_0\ge 3\beta $$ and $$m(1-y_0)\ge 3\beta $$,
  $$\begin{aligned} |\widetilde{{{\mathcal {B}}_{m,\varepsilon }^\pm }}(y,y_0,0)|\lesssim m(m|y-y_0\pm i\varepsilon |)^{\frac{1}{2}-\mu }Q_{1,m}. \end{aligned}$$

#### Proposition 8.20

Let $\beta ^2=1/4$ and $y,y_0\in [0,1]$ such that $m|y-y_0|\le 3\beta$. Then,

- For $$my_0\le 3\beta $$,
  $$\begin{aligned} |\widetilde{{{\mathcal {B}}_{m,\varepsilon }^\pm }}(y,y_0,0)|\lesssim m^{-\frac{1}{2}}y_0^{-\frac{3}{2}}(m|y-y_0\pm i\varepsilon |)^{\frac{1}{2}}\left( 1 + \big | \log \left( m|y-y_0\pm i\varepsilon |\right) \big | \right) Q_{1,m} \end{aligned}$$
- For $$my_0\ge 3\beta $$ and $$m(1-y_0)\le 3\beta $$,
  $$\begin{aligned}  &   |\widetilde{{{\mathcal {B}}_{m,\varepsilon }^\pm }}(y,y_0,0)|\lesssim \left( m + (1-y_0)^{-\frac{1}{2}}\right) (m|y-y_0\pm i\varepsilon |)^{\frac{1}{2}} \\  &   \quad \left( 1 + \big | \log \left( m|y-y_0\pm i\varepsilon |\right) \big | \right) Q_{1,m}, \end{aligned}$$
- For $$my_0\ge 3\beta $$ and $$m(1-y_0)\ge 3\beta $$,
  $$\begin{aligned} |\widetilde{{{\mathcal {B}}_{m,\varepsilon }^\pm }}(y,y_0,0)|\lesssim m(m|y-y_0\pm i\varepsilon |)^{\frac{1}{2}} \left( 1 + \big | \log \left( m|y-y_0\pm i\varepsilon |\right) \big | \right) Q_{1,m}. \end{aligned}$$

We upgrade the above pointwise estimates to integral bounds for $y\in [0,1]$ such that $2\beta \le m|y-y_0|\le 3\beta$, which will be useful later on.

#### Corollary 8.21

Let $$0\le \varepsilon \le \varepsilon _0\le \frac{\beta }{m}$$. Then,

- For $$my_0\le 3\beta $$,
  $$\begin{aligned} \Vert \widetilde{{{\mathcal {B}}_{m,\varepsilon }^\pm }}(\cdot ,y_0,0)\Vert _{L^2_y(J_2^c\cap J_3)}\lesssim m^{-1}y_0^{-\frac{3}{2}}Q_{1,m} \end{aligned}$$
- For $$my_0\ge 3\beta $$ and $$m(1-y_0)\le 3\beta $$,
  $$\begin{aligned} \Vert \widetilde{{{\mathcal {B}}_{m,\varepsilon }^\pm }}(\cdot ,y_0,0)\Vert _{L^2_y(J_2^c\cap J_3)}\lesssim m^{-\frac{1}{2}}\left( m + (1-y_0)^{-\frac{1}{2}}\right) Q_{1,m}, \end{aligned}$$
- For $$my_0\ge 3\beta $$ and $$m(1-y_0)\ge 3\beta $$,
  $$\begin{aligned} \Vert \widetilde{{{\mathcal {B}}_{m,\varepsilon }^\pm }}(\cdot ,y_0,0)\Vert _{L^2_y(J_2^c\cap J_3)}\lesssim m^{\frac{1}{2}}Q_{1,m}. \end{aligned}$$

We finish the section with estimates for

$$\begin{aligned} \partial _y{\mathcal {B}}_{m,\varepsilon }^\pm (y,y_0,z)=\partial _y\partial _z{\mathcal {G}}_{m,\varepsilon }^\pm (y,y_0,z)\partial _z\varphi _{m,\varepsilon }^\pm (z,y_0) \end{aligned}$$

for $z=0$ and $z=1$ under the localizing assumption that $m|y-y_0|\le 3\beta$. The next two results follows directly from Proposition 8.1, Proposition 8.2 and Proposition 8.3.

#### Proposition 8.22

Let $\beta ^2\ne 1/4$. Let $y,y_0\in [0,1]$ such that $m|y-y_0|\le 3\beta$. Then,

- For $my_0\le 3\beta$, we have that
  $$\begin{aligned} |\partial _y{\mathcal {B}}_{m,\varepsilon }^\pm (y,y_0,0)|\lesssim m^{-\frac{1}{2}}y_0^{-\frac{1}{2}+\mu }|y-y_0\pm i\varepsilon |^{-\frac{1}{2}-\mu }Q_{0,m}. \end{aligned}$$
- For $my_0\ge 3\beta$, we have that
  $$\begin{aligned} |\partial _y{\mathcal {B}}_{m,\varepsilon }^\pm (y,y_0,0)|\lesssim m^{\frac{1}{2}-\mu }|y-y_0\pm i\varepsilon |^{-\frac{1}{2}-\mu }Q_{0,m}. \end{aligned}$$

#### Proposition 8.23

Let $b^2=1/4$ and $y,y_0\in [0,1]$ such that $m|y-y_0|\le 3\beta$. Then,

- For $my_0\le 3\beta$, we have that
  $$\begin{aligned} |\partial _y{\mathcal {B}}_{m,\varepsilon }^\pm (y,y_0,0)|\lesssim m^{-\frac{1}{2}}y_0^{-\frac{1}{2}}|y-y_0\pm i\varepsilon |^{-\frac{1}{2}} \left( 1 + \big | \log \left( m|y-y_0\pm i\varepsilon |\right) \big | \right) Q_{0,m}. \end{aligned}$$
- For $my_0\ge 3\beta$, we have that
  $$\begin{aligned} |\partial _y{\mathcal {B}}_{m,\varepsilon }^\pm (y,y_0,0)|\lesssim m^{\frac{1}{2}}|y-y_0\pm i\varepsilon |^{-\frac{1}{2}} \left( 1 + \big | \log \left( m|y-y_0\pm i\varepsilon |\right) \big | \right) Q_{0,m}. \end{aligned}$$

Finally, we state the integral bounds that are deduced from the above estimates.

#### Corollary 8.24

Let $$0\le \varepsilon \le \varepsilon _0$$. Then,

- For $my_0\le 3\beta$, we have that
  $$\begin{aligned} \Vert \partial _y{\mathcal {B}}_{m,\varepsilon }^\pm (\cdot ,y_0,0) \Vert _{L^2_y(J_2^c\cap J_3)}\lesssim (my_0)^{-\frac{1}{2}}Q_{0,m}. \end{aligned}$$
- For $my_0\ge 3\beta$, we have that
  $$\begin{aligned} \Vert \partial _y{\mathcal {B}}_{m,\varepsilon }^\pm (\cdot ,y_0,0) \Vert _{L^2_y(J_2^c\cap J_3)}\lesssim m^{\frac{1}{2}}Q_{0,m}. \end{aligned}$$

## Estimates for the Generalized Stream-Functions

This section is devoted to obtaining estimates for the generalized stream-functions $\psi _{m,\varepsilon }^\pm (y,y_0)$ and densities $\rho _{m,\varepsilon }^{\pm }(y,y_0,z)$, as well as for some of their derivatives. Moreover, we define

$$\begin{aligned} \widetilde{\psi _m}(y,y_0):=\lim _{\varepsilon \rightarrow 0}\psi _{m,\varepsilon }^-(y,y_0) - \psi _{m,\varepsilon }^+(y,y_0), \end{aligned}$$

and similarly

$$\begin{aligned} \widetilde{\rho _m}(y,y_0):=\lim _{\varepsilon \rightarrow 0}\rho _{m,\varepsilon }^-(y,y_0) - \rho _{m,\varepsilon }^+(y,y_0). \end{aligned}$$

We state the following proposition regarding estimates for $\partial _{y_0}\varphi _{m,\varepsilon }^\pm (y,y_0)$ and $\partial _{y,y_0}^2\varphi _{m,\varepsilon }^\pm (y,y_0)$, from which one obtains the corresponding estimates for $\partial _{y_0}\widetilde{\psi _m}(y,y_0)$ and $\partial _{y,y_0}^2\widetilde{\psi _m}(y,y_0)$, respectively.

### Proposition 9.1

The following holds true.

(i) For $m|y-y_0|\le 3\beta$ and $\beta ^2\ne 1/4$, we have that
  $$\begin{aligned} |\partial _{y_0}\varphi _{m,\varepsilon }^\pm (y,y_0)|\lesssim \frac{1}{m^{1+\mu }}|y-y_0|^{-\frac{1}{2}-\mu }Q_{1,m} + \sum _{\sigma =0,1}|{\mathcal {B}}_{m,\varepsilon }^\pm (y,y_0,\sigma )|, \end{aligned}$$
  and
  $$\begin{aligned} |\partial _{y,y_0}^2\varphi _{m,\varepsilon }^\pm (y,y_0)|\lesssim \frac{1}{m^{1+\mu }}|y-y_0|^{-\frac{3}{2}-\mu }Q_{1,m} + \sum _{\sigma =0,1}|\partial _y{\mathcal {B}}_{m,\varepsilon }^\pm (y,y_0,\sigma )|, \end{aligned}$$
  where the bounds for $|{\mathcal {B}}_{m,\varepsilon }^\pm (y,y_0,\sigma )|$ and $|\partial _y{\mathcal {B}}_{m,\varepsilon }^\pm (y,y_0,\sigma )|$ for $\sigma =0,1$ are given in Propositions 8.11 and 8.22, respectively.
(ii) For $m|y-y_0|\le 3\beta$ and $\beta ^2=1/4$, we have that
  $$\begin{aligned}  &   |\partial _{y_0}\varphi _{m,\varepsilon }^\pm (y,y_0)|\lesssim \frac{1}{m} |y-y_0|^{-\frac{1}{2}} \left( 1 + \big | \log \left( m|y-y_0 \pm i\varepsilon |\right) \big | \right) Q_{1,m} \\  &   \quad + \sum _{\sigma =0,1}|{\mathcal {B}}_{m,\varepsilon }^\pm (y,y_0,\sigma )|, \end{aligned}$$
  and
  $$\begin{aligned}  &   |\partial _{y,y_0}^2\varphi _{m,\varepsilon }^\pm (y,y_0)|\lesssim \frac{1}{m}|y-y_0|^{-\frac{3}{2}} \left( 1 + \big | \log \left( m|y-y_0\pm i\varepsilon |\right) \big | \right) Q_{1,m} \\  &   \quad + \sum _{\sigma =0,1}|\partial _y{\mathcal {B}}_{m,\varepsilon }^\pm (y,y_0,\sigma )|, \end{aligned}$$
  where the bounds for $|{\mathcal {B}}_{m,\varepsilon }^\pm (y,y_0,\sigma )|$ and $|\partial _y{\mathcal {B}}_{m,\varepsilon }^\pm (y,y_0,\sigma )|$ for $\sigma =0,1$ are given in Propositions 8.12 and 8.23, respectively.
(iii) For $m|y-y_0|\ge 3\beta$, we have that
  $$\begin{aligned} \Vert \partial _{y}\partial _{y_0} \varphi _{m,\varepsilon }^\pm \Vert _{L^2_y(J_3^c)}^2 + m^2 \Vert \partial _{y_0} \varphi _{m,\varepsilon }^\pm \Vert _{L^2_y(J_3^c)}^2 \lesssim Q_{1,m}^2 + m^2 \sum _{\sigma =0,1}\Vert {\mathcal {B}}_{m,\varepsilon }^\pm (\cdot ,y_0,\sigma )\Vert _{L^2_y(J_2^c\cap J_3)}^2, \end{aligned}$$
  where the bounds for $\Vert {\mathcal {B}}_{m,\varepsilon }^\pm (\cdot ,y_0,\sigma )\Vert _{L^2_y(J_2^c\cap J_3)}$ are given in Corollary 8.13.

In particular, these bounds also apply to $\partial _{y_0}\widetilde{\psi _{m}}(y,y_0)$ and $\partial _{y,y_0}^2\widetilde{\psi _{m}}(y,y_0)$.

### Proof

Both (*i*) and (*ii*) follows from Proposition 3.5 and Proposition 7.2. As for (*iii*), we argue assuming that $\beta ^2\ne 1/4$. Taking a $\partial _{y_0}$ derivative in (2.11), we see that

$$\begin{aligned} {\textsc {TG}}_{m,\varepsilon }^\pm \partial _{y_0}\varphi _{m,\varepsilon }^\pm = \partial _{y_0}F_{m,\varepsilon }^\pm -2\beta ^2\frac{1}{(y-y_0\pm i\varepsilon )^3}\varphi _{m,\varepsilon }^\pm . \end{aligned}$$

In order to use Lemma 7.1, we need to control $\left\| \partial _{y_0}\varphi _{m,\varepsilon }^\pm \right\| _{L^2_y(J_2^c\cap J_3)}$ and $\left\| \frac{1}{(y-y_0\pm i\varepsilon )^3}\varphi _{m,\varepsilon }^\pm \right\| _{L^2_y(J_2^c)}$. We begin by estimating

$$\begin{aligned}  &   \int _{y_0+\frac{2\beta }{m}}^{y_0+\frac{3\beta }{m}}|\partial _{y_0}\varphi _{m,\varepsilon }^\pm (y,y_0)|^2\textrm{d}y \lesssim \sum _{\sigma =0,1}\int _{y_0+\frac{2\beta }{m}}^{y_0+\frac{3\beta }{m}}|{\mathcal {B}}_{m,\varepsilon }^\pm (y,y_0,\sigma )|^2\textrm{d}y + Q_{1,m}^2 \\  &   \quad \int _{y_0+\frac{2\beta }{m}}^{y_0+\frac{3\beta }{m}} \frac{1}{m^{2+2\mu }}|y-y_0|^{-1-2\mu }\textrm{d}y \end{aligned}$$

Now, for $\beta ^2< 1/4$ we have $\mu \ne 0$ and

$$\begin{aligned} Q_{1,m}^2 \int _{y_0+2\frac{\beta }{m}}^{y_0+3\frac{\beta }{m}} \frac{1}{m^{2+2\mu }}|y-y_0|^{-1-2\mu }\textrm{d}y \lesssim \frac{1}{m^2}Q_{1,m}^2, \end{aligned}$$

while for $\beta ^2>1/4$, we have $\mu =0$ and therefore the bound still becomes

$$\begin{aligned} Q_{1,m}^2 \int _{y_0+2\frac{\beta }{m}}^{y_0+3\frac{\beta }{m}} \frac{1}{m^{2}}|y-y_0|^{-1}\textrm{d}y \lesssim \frac{1}{m^2}Q_{1,m}^2\left( \log \left( \frac{3\beta }{m}\right) - \log \left( \frac{2\beta }{m}\right) \right) \lesssim \frac{1}{m^2}Q_{1,m}^2. \end{aligned}$$

Therefore, we conclude that

$$\begin{aligned} \left\| \partial _{y_0}\varphi _{m,\varepsilon }^\pm \right\| _{L^2_y(J_2^c\cap J_3)}\lesssim \frac{1}{m}Q_{1,m} + \sum _{\sigma =0,1}\Vert {\mathcal {B}}_{m,\varepsilon }^\pm (\cdot ,y_0,\sigma )\Vert _{L^2_y(J_2^c\cap J_3)} \end{aligned}$$

On the other hand, we use Proposition 7.2 applied to $\varphi _{m,\varepsilon }^\pm (y,y_0)$ to estimate

$$\begin{aligned}  &   \left\| \frac{1}{(y-y_0\pm i\varepsilon )^3}\varphi _{m,\varepsilon }^\pm \right\| _{L^2_y(J_2^c\cap J_3)}^2\lesssim \int _{2\beta \le m|y-y_0|\le 3\beta } \frac{1}{m^{2+2\mu }}|y-y_0\pm i\varepsilon |^{-5-2\mu }Q_{0,m} \textrm{d}y \\  &   \quad \lesssim m^2Q_{0,m} \end{aligned}$$

and

$$\begin{aligned} \left\| \frac{1}{(y-y_0\pm i\varepsilon )^3}\varphi _{m,\varepsilon }^\pm \right\| _{L^2_y(J_3^c)}^2\lesssim m^6\Vert \varphi _{m,\varepsilon }^\pm \Vert _{L^2_y(J_3^c)}\lesssim m^2Q_{0,m}. \end{aligned}$$

The result follows from applying Lemma 7.1. $\square$

The next proposition gives bounds on $\partial _{y_0}^2\varphi _{m,\varepsilon }^\pm$ and therefore also on $\partial _{y_0}^2\widetilde{\psi _{m}}(y,y_0)$.

### Proposition 9.2

The following holds true.

- For $m|y-y_0|\le 3\beta$ and $\beta ^2\ne 1/4$, we have that
  $$\begin{aligned}  &   |\partial _{y_0}^2\varphi _{m,\varepsilon }^\pm (y,y_0)|\lesssim \frac{1}{m^{1+\mu }}|y-y_0|^{-\frac{3}{2}-\mu }Q_{2,m} \\  &   \quad + \sum _{\sigma =0,1}\Big ( |\partial _y{\mathcal {B}}_{m,\varepsilon }^\pm (y,y_0,\sigma )| + |\widetilde{{\mathcal {B}}_{m,\varepsilon }^\pm }(y,y_0,\sigma )|\Big ), \end{aligned}$$
  where the bounds for $|\partial _y{\mathcal {B}}_{m,\varepsilon }^\pm (y,y_0,\sigma )|$ and $|\widetilde{{\mathcal {B}}_{m,\varepsilon }^\pm }(y,y_0,\sigma )|$ are given in Propositions 8.22 and 8.19, respectively.
- For $m|y-y_0|\le 3\beta$ and $\beta ^2=1/4$, we have that
  $$\begin{aligned} |\partial _{y_0}^2\varphi _{m,\varepsilon }^\pm (y,y_0)|&\lesssim \frac{1}{m}|y-y_0|^{-\frac{3}{2}} \left( 1 + \big | \log \left( m|y-y_0\pm i\varepsilon |\right) \big | \right) Q_{2,m} \\&\quad + \sum _{\sigma =0,1}\Big ( |\partial _y{\mathcal {B}}_{m,\varepsilon }^\pm (y,y_0,\sigma )| + |\widetilde{{\mathcal {B}}_{m,\varepsilon }^\pm }(y,y_0,\sigma )|\Big ), \end{aligned}$$
  where the bounds for $|\partial _y{\mathcal {B}}_{m,\varepsilon }^\pm (y,y_0,\sigma )|$ and $|\widetilde{{\mathcal {B}}_{m,\varepsilon }^\pm }(y,y_0,\sigma )|$ are given in Propositions 8.23 and 8.20, respectively.
- For $m|y-y_0|\ge 3\beta$, we have that
  $$\begin{aligned}&\Vert \partial _{y_0}^2\varphi _{m,\varepsilon }^\pm \Vert _{L^2_y(J_3^c)} \lesssim Q_{2,m} \\&\quad + \sum _{\sigma =0,1}\left( \Vert \partial _y{\mathcal {B}}_{m,\varepsilon }^\pm (\cdot ,y_0,\sigma )\Vert _{L^2_y(J_2^c\cap J_3)} + \Vert \widetilde{{\mathcal {B}}_{m,\varepsilon }^\pm }(\cdot ,y_0,\sigma )\Vert _{L^2_y(J_2^c\cap J_3)}\right) \\&\quad +m \sum _{\sigma =0,1} \Vert B_{m,\varepsilon }^\pm (\cdot ,y_0,\sigma )\Vert _{L^2_y(J_2^c\cap J_3)}, \end{aligned}$$
  where the estimates for $\Vert {\mathcal {B}}_{m,\varepsilon }^\pm (\cdot ,y_0,\sigma )\Vert _{L^2_y(J_2^c\cap J_3)}$, $\Vert \partial _y{\mathcal {B}}_{m,\varepsilon }^\pm (\cdot ,y_0,\sigma )\Vert _{L^2_y(J_2^c\cap J_3)}$ and $\Vert \widetilde{{\mathcal {B}}_{m,\varepsilon }^\pm }(\cdot ,y_0,\sigma )\Vert _{L^2_y(J_2^c\cap J_3)}$ are given in Corollaries 8.13, 8.21 and 8.24, respectively.

In particular, these bounds also apply to $\partial _{y_0}^2\widetilde{\psi _{m}}(y,y_0)$.

### Proof

The first two statements of the proposition follow from Proposition 3.5 and Proposition 7.2. For the third part of the proposition, we argue for $\beta ^2\ne 1/4$. Taking $\partial _{y_0}^2$ derivatives to (2.11), we see that $\partial _{y_0}^2\varphi _{m,\varepsilon }^\pm (y,y_0)$ solves

$$\begin{aligned} {\textsc {TG}}_{m,\varepsilon }^\pm \partial _{y_0}^2\varphi _{m,\varepsilon }^\pm = \partial _{y_0}^2F_{m,\varepsilon }^\pm -4\beta ^2\frac{\partial _{y_0}\varphi _{m,\varepsilon }^\pm }{(y-y_0\pm i\varepsilon )^3} - 6\beta ^2 \frac{\varphi _{m,\varepsilon }^\pm }{(y-y_0\pm i\varepsilon )^4}. \end{aligned}$$

In order to use Lemma 7.1, we need to bound $\Vert \partial _{y_0}^2\varphi _{m,\varepsilon }^\pm \Vert _{L^2_y(J_2^c\cap J_3)}$, as well as $\Vert \frac{\partial _{y_0}\varphi _{m,\varepsilon }^\pm }{(y-y_0\pm i\varepsilon )^3}\Vert _{L^2_y(J_2^c\cap J_3)}$ and $\Vert \frac{\varphi _{m,\varepsilon }^\pm }{(y-y_0\pm i\varepsilon )^4}\Vert _{L^2_y(J_2^c\cap J_3)}$. We estimate

$$\begin{aligned} \int _{2\beta \le m|y-y_0|\le 3\beta }&|\partial _{y_0}^2\varphi _{m,\varepsilon }^\pm (y,y_0)|^2\textrm{d}y \\&\lesssim Q_{2,m}\int _{2\beta \le |y-y_0|\le 3\beta } \frac{1}{m^{2+2\mu }}|y-y_0|^{-3-2\mu }\textrm{d}y \\&\qquad + \sum _{\sigma =0,1}\Big ( \Vert \partial _y{\mathcal {B}}_{m,\varepsilon }^\pm (\cdot ,y_0,\sigma )\Vert _{L^2_y(J_2^c\cap J_3)} + \Vert \widetilde{{\mathcal {B}}_{m,\varepsilon }^\pm }(\cdot ,y_0,\sigma )\Vert _{L^2_y(J_2^c\cap J_3)}\Big ) \\&\lesssim Q_{2,m} + \sum _{\sigma =0,1}\Big ( \Vert \partial _y{\mathcal {B}}_{m,\varepsilon }^\pm (\cdot ,y_0,\sigma )\Vert _{L^2_y(J_2^c\cap J_3)} + \Vert \widetilde{{\mathcal {B}}_{m,\varepsilon }^\pm }(\cdot ,y_0,\sigma )\Vert _{L^2_y(J_2^c\cap J_3)}\Big ). \end{aligned}$$

Similarly, from Proposition 9.1 we have that

$$\begin{aligned}  &   \left\| \frac{\partial _{y_0}\varphi _{m,\varepsilon }^\pm }{(y-y_0\pm i\varepsilon )^3}\right\| _{L^2_y(J_2^c)}\lesssim m^3\Vert {\partial _{y_0}\varphi _{m,\varepsilon }^\pm }\Vert _{L^2_y(J_2^c\cap J_3)}\\  &   \quad \lesssim m^2Q_{1,m} + m^3\sum _{\sigma =0,1}\Vert {\mathcal {B}}_{m,\varepsilon }^\pm (\cdot ,y_0,\sigma )\Vert _{L^2_y(J_2^c\cap J_3)}, \end{aligned}$$

while using Proposition 7.2 we obtain

$$\begin{aligned} \left\| \frac{\varphi _{m,\varepsilon }^\pm }{(y-y_0\pm i\varepsilon )^4}\right\| _{L^2_y(J_2^c)}\lesssim m^4\Vert {\varphi _{m,\varepsilon }^\pm }\Vert _{L^2_y(J_2^c\cap J_3)}\lesssim m^2Q_{0,m}. \end{aligned}$$

With this, the proof is complete. $\square$

We finish the subsection by providing the estimates for $\widetilde{\rho _m}$ and $\partial _{y_0}\widetilde{\rho _m}$.

### Proposition 9.3

The following holds true.

- For $m|y-y_0|\le 3\beta$ and $\beta ^2\ne 1/4$, we have that
  $$\begin{aligned} |\widetilde{\rho _{m}}(y,y_0)|\lesssim \frac{1}{m^{1+\mu }}|y-y_0|^{-\frac{1}{2}-\mu }Q_{0,m}. \end{aligned}$$
  and
  $$\begin{aligned}  &   |\partial _{y_0}\widetilde{\rho _{m}}(y,y_0)|\lesssim \frac{1}{m^{1+\mu }}|y-y_0|^{-\frac{3}{2}-\mu }Q_{1,m} \\  &   \quad + \sup _{0\le \varepsilon \le \varepsilon _0} \sum _{\sigma =0,1}\sum _{\kappa \in \lbrace +,-\rbrace } |y-y_0 +\kappa i\varepsilon |^{-1} |{\mathcal {B}}_{m,\varepsilon }^\kappa (y,y_0,\sigma )|, \end{aligned}$$
  where the bounds for $|{\mathcal {B}}_{m,\varepsilon }^\pm (y,y_0,\sigma )|$ for $\sigma =0,1$ are given in Proposition 8.11.
- For $m|y-y_0|\le 3\beta$ and $\beta ^2=1/4$, we have that
  $$\begin{aligned} |\widetilde{\rho _{m}}(y,y_0)|\lesssim \frac{1}{m}|y-y_0|^{-\frac{1}{2}} \left( 1 + \big | \log \left( m|y-y_0\pm i\varepsilon |\right) \big | \right) Q_{0,m}. \end{aligned}$$
  and
  $$\begin{aligned} |\partial _{y_0}\widetilde{\rho _{m}}(y,y_0)|&\lesssim \frac{1}{m}|y-y_0|^{-\frac{3}{2}} \left( 1 + \big | \log \left( m|y-y_0\pm i\varepsilon |\right) \big | \right) Q_{1,m} \\&\quad + \sup _{0\le \varepsilon \le \varepsilon _0} \sum _{\sigma =0,1}\sum _{\kappa \in \lbrace +,-\rbrace } |y-y_0 +\kappa i\varepsilon |^{-1} |{\mathcal {B}}_{m,\varepsilon }^\kappa (y,y_0,\sigma )|, \end{aligned}$$
  where the bounds for $|{\mathcal {B}}_{m,\varepsilon }^\pm (y,y_0,\sigma )|$ for $\sigma =0,1$ are given in Proposition 8.12.
- For $m|y-y_0|\ge 3\beta$, we have that
  $$\begin{aligned} \Vert \widetilde{\rho _m}\Vert _{L^2_y(J_3^c)}\lesssim \frac{1}{m}Q_{0,m} \end{aligned}$$
  and
  $$\begin{aligned} \Vert \partial _{y_0}\widetilde{\rho _m}\Vert _{L^2_y(J_3^c)}\lesssim Q_{1,m} + m\sum _{\sigma =0,1}\sum _{\kappa \in \lbrace +,-\rbrace } \Vert {\mathcal {B}}_{m,\varepsilon }^\kappa (y,y_0,\sigma )\Vert _{L^2_y(J_2^c\cap J_3)} \end{aligned}$$

### Proof

The bounds follow directly from Proposition 3.6, Proposition 7.2 and Proposition 9.1. $\square$

## Time-Decay Estimates

This section is devoted to the proof of the time decay estimate rates of the stream function $\psi _{m}(t,y)$, its $\partial _y\psi _m(t,y)$ derivative and the density $\rho _m(t,y)$. Let us recall that we can write

$$\begin{aligned} \psi _m(t,y)=\frac{1}{2\pi i}\lim _{\varepsilon \rightarrow 0} \int _0^1 \textrm{e}^{-imy_0t}\left( \psi ^-_m(y,y_0) - \psi _m^+(y,y_0)\right) \textrm{d}y_0, \end{aligned}$$

and

$$\begin{aligned} \rho _m(t,y)=\frac{1}{2\pi i}\lim _{\varepsilon \rightarrow 0} \int _0^1 \textrm{e}^{-imy_0t}\left( \rho ^-_m(y,y_0) - \rho _m^+(y,y_0)\right) \textrm{d}y_0. \end{aligned}$$

A simple integration by parts provides

$$\begin{aligned} \begin{aligned} \psi _m(t,y)&=-\frac{1}{2\pi i}\frac{1}{imt}\lim _{\varepsilon \rightarrow 0}\big [ \textrm{e}^{-imy_0t}\left( \psi _{m,\varepsilon }^-(y,y_0)-\psi _{m,\varepsilon }^+(y,y_0)\right) \big ]_{y_0=0}^{y_0=1} \\&\quad +\frac{1}{2\pi i}\frac{1}{imt}\lim _{\varepsilon \rightarrow 0}\int _0^1 \textrm{e}^{-imy_0t}\left( \partial _{y_0}\psi _{m,\varepsilon }^-(y,y_0)- \partial _{y_0}\psi _{m,\varepsilon }^+(y,y_0)\right) \textrm{d}y_0 \\&=\frac{1}{2\pi i}\frac{1}{imt}\lim _{\varepsilon \rightarrow 0}\int _0^1 \textrm{e}^{-imy_0t}\left( \partial _{y_0}\psi _{m,\varepsilon }^-(y,y_0)- \partial _{y_0}\psi _{m,\varepsilon }^+(y,y_0)\right) \textrm{d}y_0 \end{aligned} \end{aligned}$$

where we use Theorem 4 to show that the solid terms associated to the spectral boundary vanish. Throughout the entire section, let us consider $\beta ^2\ne 1/4$, unless we state otherwise.

We begin proving the following result.

### Proposition 10.1

Let $$t\ge 1$$. Then,

$$\begin{aligned} \begin{aligned} \Vert \psi _m(t) \Vert _{L^2_y}&\lesssim m^{-\frac{3}{2}}t^{-\frac{3}{2}+\mu } Q_{2,m} \\ \end{aligned} \end{aligned}$$

### Proof

We write

$$\begin{aligned} \begin{aligned} \psi _m(t,y)&=\frac{1}{2\pi i}\frac{1}{imt}\lim _{\varepsilon \rightarrow 0}\int _0^1 \textrm{e}^{-imy_0t}\left( \partial _{y_0}\psi _{m,\varepsilon }^-(y,y_0)- \partial _{y_0}\psi _{m,\varepsilon }^+(y,y_0)\right) \textrm{d}y_0. \end{aligned} \end{aligned}$$

Let us denote $\delta _0:=\min \left( \frac{3\beta }{m},\frac{1}{2}\right)$ and let $\delta \in \left( 0,\frac{\delta _0}{2}\right)$. In particular, we note that $m\delta \le 3\beta$, it is bounded. We shall first show the decay rates for $\Vert \psi _m(t) \Vert _{L^2_y(\delta ,1-\delta )}$ and then for $\Vert \psi _m(t) \Vert _{L^2_y(0,\delta )}$ and $\Vert \psi _m(t) \Vert _{L^2_y(1-\delta ,1)}$.

$\bullet$ **Step 1.** For $y\in (\delta , 1-\delta )$, we write

$$\begin{aligned} \begin{aligned} \psi _m(t,y)&=\frac{1}{2\pi i}\frac{1}{imt}\lim _{\varepsilon \rightarrow 0}\left( \int _0^{y-\frac{\delta }{2}} +\int _{y-\frac{\delta }{2}}^{y+\frac{\delta }{2}} + \int _{y+\frac{\delta }{2}}^1\right) \textrm{e}^{-imy_0t}\\  &\quad \left( \partial _{y_0}\psi _{m,\varepsilon }^-(y,y_0)- \partial _{y_0}\psi _{m,\varepsilon }^+(y,y_0)\right) \textrm{d}y_0 \\&=\mathcal {T}_1 + \mathcal {T}_2 + \mathcal {T}_3. \end{aligned} \end{aligned}$$

and we begin with estimating $\mathcal {T}_2$. There, we have that $|y-y_0|\le \frac{\delta }{2}\le \frac{\delta _0}{4}$ and we can use the bounds from Proposition 9.1 to bound

$$\begin{aligned} |\mathcal {T}_2|&\lesssim \frac{1}{mt}\int _{y-\frac{\delta }{2}}^{y+\frac{\delta }{2}}\frac{1}{m^{1+\mu }}|y-y_0|^{-\frac{1}{2}+\mu }Q_{1,m}\textrm{d}y_0 + \frac{1}{mt}\int _{y-\frac{\delta }{2}}^{y+\frac{\delta }{2}} \sum _{\sigma =0,1}\sum _{\kappa \in \lbrace +,-\rbrace }|{\mathcal {B}}_{m,\varepsilon }^\kappa (y,y_0,\sigma )| \textrm{d}y_0 \\&=\mathcal {T}_{2,1} + \mathcal {T}_{2,2}. \end{aligned}$$

We can integrate directly to obtain

$$\begin{aligned} |\mathcal {T}_{2,1}|\lesssim \frac{1}{m^{2+\mu }t}\delta ^{\frac{1}{2}-\mu }Q_{1,m}. \end{aligned}$$

For $\mathcal {T}_{2,2}$, for $\sigma =0$ we decompose

$$\begin{aligned}  &   \frac{1}{mt}\int _{y-\frac{\delta }{2}}^{y+\frac{\delta }{2}} |{\mathcal {B}}_{m,\varepsilon }^\pm (y,y_0,\sigma )| \textrm{d}y_0 = \frac{1}{mt}\int _{y-\frac{\delta }{2}}^{y+\frac{\delta }{2}} \left( \chi _{y_0\le \frac{3\beta }{m}} + \chi _{y_0> \frac{3\beta }{m}}\right) |{\mathcal {B}}_{m,\varepsilon }^\pm (y,y_0,\sigma )| \textrm{d}y_0 \\  &   \quad = \mathcal {T}_{2,2,1} + \mathcal {T}_{2,2,2}. \end{aligned}$$

We use Proposition 8.11 to compute

$$\begin{aligned} \mathcal {T}_{2,2,1}\lesssim \frac{1}{mt}\int _{y-\frac{\delta }{2}}^{y+\frac{\delta }{2}}m^{-1+\mu }y_0^{-\frac{1}{2}+\mu }Q_{0,m}\textrm{d}y_0\lesssim \frac{1}{m^{2-\mu }t}\delta ^{\frac{1}{2}+\mu }Q_{0,m} \end{aligned}$$

and

$$\begin{aligned} \mathcal {T}_{2,2,2}\lesssim \frac{1}{mt}\int _{y-\frac{\delta }{2}}^{y+\frac{\delta }{2}}Q_{0,m}\textrm{d}y_0\lesssim \frac{1}{mt}\delta Q_{0,m}. \end{aligned}$$

The bounds for the terms of $T_{2,2}$ for $\sigma =1$ are the same, we omit the details. We summarize these estimates into

$$\begin{aligned} \Vert {{\mathcal {T}}}_2 \Vert _{L^2_y(\delta ,1-\delta )}\lesssim \frac{1}{mt}\left( m^{-\frac{3}{2}}(m\delta )^{\frac{1}{2}-\mu } + m^{-\frac{3}{2}}(m\delta )^{\frac{1}{2}+\mu } + \delta \right) Q_{1,m} \end{aligned}$$

We shall next estimate $\mathcal {T}_1$, the bounds of $\mathcal {T}_3$ are the same and the arguments to prove them are identical. For $\mathcal {T}_1$, note that we can further integrate by parts,

$$\begin{aligned} \begin{aligned} \mathcal {T}_1&=-\frac{1}{2\pi i}\frac{1}{m^2t^2}\lim _{\varepsilon \rightarrow 0}\big [ \textrm{e}^{-imy_0t}\partial _{y_0}\left( \psi _{m,\varepsilon }^-(y,y_0)-\psi _{m,\varepsilon }^+(y,y_0)\right) \big ]_{y_0=\frac{\delta }{2}}^{y_0=y-\frac{\delta }{2}} \\&\quad + \frac{1}{2\pi i}\frac{1}{imt}\lim _{\varepsilon \rightarrow 0}\int _0^{\frac{\delta }{2}} \textrm{e}^{-imy_0t}\partial _{y_0}\left( \psi _{m,\varepsilon }^-(y,y_0)- \psi _{m,\varepsilon }^+(y,y_0)\right) \textrm{d}y_0 \\&\quad + \frac{1}{2\pi i}\frac{1}{m^2t^2}\lim _{\varepsilon \rightarrow 0}\int _{\frac{\delta }{2}}^{y-\frac{\delta }{2}} \textrm{e}^{-imy_0t}\partial _{y_0}^2\left( \psi _{m,\varepsilon }^-(y,y_0)- \psi _{m,\varepsilon }^+(y,y_0)\right) \textrm{d}y_0 \\&=\mathcal {T}_{1,1}+ \mathcal {T}_{1,2}+\mathcal {T}_{1,3}. \end{aligned} \end{aligned}$$

We shall treat each $\mathcal {T}_{1,i}$, for $i=1,2,3$ separately.

$\diamond$ *Estimates for* $\mathcal {T}_{1,1}.$ For the boundary terms of $\mathcal {T}_{1,1}$, consider first $y_0=y-\frac{\delta }{2}$. Then, $|y-y_0|=\frac{\delta }{2}\le \frac{\delta _0}{4}$, so that from Proposition 9.1 we have

$$\begin{aligned}  &   \frac{1}{m^2t^2}\left| \partial _{y_0}\widetilde{\psi _m}\left( y,y-\tfrac{\delta }{2}\right) \right| \lesssim \frac{1}{m^2t^2}\\  &   \quad \left( \frac{1}{m^{1+\mu }}\delta ^{-\frac{1}{2}-\mu }Q_{1,m} + \sum _{\sigma =0,1}\sum _{\kappa \in \lbrace +,- \rbrace } \left| {\mathcal {B}}_{m,\varepsilon }^\kappa \left( y,y-\tfrac{\delta }{2},\sigma \right) \right| \right) . \end{aligned}$$

Now, from Proposition 8.11 we have

$$\begin{aligned} \sum _{\sigma =0,1}\sum _{\kappa \in \lbrace +,- \rbrace }\left| {\mathcal {B}}_{m,\varepsilon }^\kappa \left( y,y-\tfrac{\delta }{2},\sigma \right) \right| \lesssim \left( 1 + m^{-\frac{1}{2}}(m\delta )^{-\frac{1}{2}+\mu }\right) Q_{0,m}, \end{aligned}$$

since $$y\in (\delta ,1-\delta )$$ ensures $$y-\frac{\delta }{2}>\frac{\delta }{2}$$.

For the boundary term $\mathcal {T}_{1,1}$ associated to $y_0=\frac{\delta }{2}$, since $y\in (\delta , 1-\delta )$, we have that $1-\frac{\delta }{2}\ge y-y_0\ge \frac{\delta }{2}$. Hence, for those $y\in (\delta ,1-\delta )$ such that $m|y-y_0|\le 3\beta$, we use Proposition 9.1 to pointwise estimate

$$\begin{aligned} \frac{1}{m^2t^2}\left| \partial _{y_0}\widetilde{\psi _m}\left( y,\tfrac{\delta }{2}\right) \right| \lesssim \frac{1}{m^2t^2}\left( \frac{1}{m^{1+\mu }}\delta ^{-\frac{1}{2}-\mu }Q_{1,m} + \sum _{\sigma =0,1}\sum _{\kappa \in \lbrace +,- \rbrace } \left| {\mathcal {B}}_{m,\varepsilon }^\kappa \left( y,\tfrac{\delta }{2},\sigma \right) \right| \right) , \end{aligned}$$

where we further have from Proposition 8.11 that

$$\begin{aligned} \sum _{\sigma =0,1}\sum _{\kappa \in \lbrace +,- \rbrace }\Vert {\mathcal {B}}_{m,\varepsilon }^\pm \left( y,\tfrac{\delta }{2},0 \right) \Vert _{L^2_y(J_3)}\lesssim \left( m^{-\frac{3}{2}}\delta ^{-\frac{1}{2}} + m^{-\frac{1}{2}}\right) Q_{0,m}. \end{aligned}$$

Next, for those $y\in (\delta ,1-\delta )$ such that $m|y-y_0|\ge 3\beta$ we can directly estimate in $L^2_y$ using Proposition 9.1 to deduce that

$$\begin{aligned} \frac{1}{m^2t^2}\left\| \partial _{y_0}\widetilde{\psi _m}\left( y,\tfrac{\delta }{2}\right) \right\| _{L^2_y(J_3^c)}\lesssim \frac{1}{m^2t^2}\left( \frac{1}{m}Q_{1,m} + \sum _{\sigma =0,1}\sum _{\kappa \in \lbrace +,-\rbrace }\Vert {\mathcal {B}}_{m,\varepsilon }^\kappa \left( y, \tfrac{\delta }{2}, \sigma \right) \Vert _{L^2_y(J_2^c\cap J_3)}\right) , \end{aligned}$$

while from Corollary 8.13 we are able to bound

$$\begin{aligned} \Vert {\mathcal {B}}_{m,\varepsilon }^\pm \left( y, \tfrac{\delta }{2}, 0 \right) \Vert _{L^2_y(J_2^c\cap J_3)}\lesssim m^{-\frac{3}{2}}\delta ^{-\frac{1}{2}}Q_{0,m}, \quad \Vert {\mathcal {B}}_{m,\varepsilon }^\pm \left( y, \tfrac{\delta }{2}, 1 \right) \Vert _{L^2_y(J_2^c\cap J_3)}\lesssim m^{-\frac{1}{2}}Q_{0,m}. \end{aligned}$$

Therefore, we have

$$\begin{aligned} \Vert {{\mathcal {T}}}_{1,1}\Vert _{L^2_y(\delta ,1-\delta )}\lesssim \frac{1}{m^2t^2}\left( 1 + m^{-\frac{1}{2}}(m\delta )^{-\frac{1}{2}-\mu } + m^{-\frac{1}{2}}(m\delta )^{-\frac{1}{2}+\mu } + m^{-\frac{3}{2}}\delta ^{-\frac{1}{2}} + m^{-\frac{1}{2}} \right) Q_{0,m} \end{aligned}$$

This concludes the analysis of $\mathcal {T}_{1,1}$.

$\diamond$ *Estimates for* $\mathcal {T}_{1,2}.$ We begin by splitting

$$\begin{aligned} |\mathcal {T}_{1,2}|\lesssim \frac{1}{mt}\int _0^{\frac{\delta }{2}}\left( \chi _{m|y-y_0|\le 3\beta } + \chi _{m|y-y_0|>3\beta } \right) |\partial _{y_0}\widetilde{\psi _m}(y,y_0)|\textrm{d}y_0 = \mathcal {T}_{1,2,1} + \mathcal {T}_{1,2,2} \end{aligned}$$

We use Proposition 9.1 to estimate

$$\begin{aligned} |{{\mathcal {T}}}_{1,2,1}|\lesssim \frac{1}{mt}\int _0^{\frac{\delta }{2}} \left( \frac{1}{m^{1+\mu }}|y-y_0|^{-\frac{1}{2}-\mu }Q_{1,m} + \chi _{m|y-y_0|\le 3\beta }\sum _{\sigma =0,1}\sum _{\kappa \in \lbrace +,- \rbrace }|{\mathcal {B}}_{m,\varepsilon }^\pm (y,y_0,\sigma )|\right) \textrm{d}y_0 \end{aligned}$$

Now, since $y\in (\delta ,1-\delta )$ and $y_0\le \frac{\delta }{2}$, we have $|y-y_0|\ge \frac{\delta }{2}$. Hence,

$$\begin{aligned} \int _0^{\frac{\delta }{2}} \frac{1}{m^{1+\mu }}|y-y_0|^{-\frac{1}{2}-\mu }Q_{1,m}\textrm{d}y_0 \lesssim \frac{1}{m^{1+\mu }}\delta ^{\frac{1}{2}-\mu }Q_{1,m}. \end{aligned}$$

Moreover, Proposition 8.11 provides

$$\begin{aligned} \sum _{\sigma =0,1}\sum _{\kappa \in \lbrace +,- \rbrace }&\left\| \int _0^{\frac{\delta }{2}} \chi _{m|y-y_0|\le 3\beta } |{\mathcal {B}}_{m,\varepsilon }^\pm (y,y_0,\sigma )| \textrm{d}y_0 \right\| _{L^2_y(\delta ,1-\delta )} \\&\lesssim \sum _{\sigma =0,1}\sum _{\kappa \in \lbrace +,- \rbrace } \int _0^{\frac{\delta }{2}} \left\| {\mathcal {B}}_{m,\varepsilon }^\pm (y,y_0,\sigma )\right\| _{L^2_y(J_3)} \textrm{d}y_0 \\&\lesssim m^{-\frac{1}{2}}\delta ^{\frac{1}{2}}\left( m^{-1} + \delta ^\frac{1}{2}\right) Q_{0,m}. \end{aligned}$$

As a result, we are able to bound

$$\begin{aligned} \Vert {{\mathcal {T}}}_{1,2,1}\Vert _{L^2_y(\delta ,1-\delta )}\lesssim \frac{1}{mt}\left( m^{-1-\mu }\delta ^{\frac{1}{2}-\mu } + m^{-\frac{3}{2}}\delta ^\frac{1}{2} + m^{-\frac{1}{2}}\delta \right) Q_{1,m}. \end{aligned}$$

We again use Proposition 9.1 and Corollary 8.13 to estimate

$$\begin{aligned} \Vert {{\mathcal {T}}}_{1,2,2}\Vert _{L^2_y(\delta ,1-\delta )}&\lesssim \frac{1}{mt}\int _0^{\frac{\delta }{2}}\Vert \partial _{y_0}\widetilde{\psi _m}(y,y_0)\Vert _{L^2_y(J_3^c)}\textrm{d}y_0 \\&\lesssim \frac{1}{mt}\int _0^{\frac{\delta }{2}} \left( \frac{1}{m}Q_{1,m} + \sum _{\sigma =0,1}\sum _{\kappa \in \lbrace +,-\rbrace }\Vert {\mathcal {B}}_{m,\varepsilon }^\kappa \left( y, \tfrac{\delta }{2}, \sigma \right) \Vert _{L^2_y(J_2^c\cap J_3)}\right) \textrm{d}y_0 \\&\lesssim \frac{1}{mt}\left( m^{-1}\delta + m^{-\frac{3}{2}}\delta ^{\frac{1}{2}} + m^{-\frac{1}{2}}\delta \right) Q_{1,m} \end{aligned}$$

so that we can conclude

$$\begin{aligned} \Vert {{\mathcal {T}}}_{1,2} \Vert _{L^2_y(\delta ,1-\delta )}\lesssim \frac{1}{mt}\left( m^{-1-\mu }\delta ^{\frac{1}{2}-\mu } + m^{-1}\delta + m^{-\frac{3}{2}}\delta ^{\frac{1}{2}} + m^{-\frac{1}{2}}\delta \right) Q_{1,m}. \end{aligned}$$

$\diamond$ *Estimates for* $\mathcal {T}_{1,3}.$ We shall split again

$$\begin{aligned} |{{\mathcal {T}}}_{1,3}|\lesssim \frac{1}{m^2t^2}\int _{\frac{\delta }{2}}^{y-\frac{\delta }{2}}\left( \chi _{m|y-y_0|\le 3\beta } + \chi _{m|y-y_0|>3\beta } \right) |\partial _{y_0}^2\widetilde{\psi _m}(y,y_0)|\textrm{d}y_0 = \mathcal {T}_{1,3,1} + \mathcal {T}_{1,3,2}. \end{aligned}$$

Now, we use Proposition 9.2 to estimate

$$\begin{aligned} |{{\mathcal {T}}}_{1,3,1}|&\lesssim \frac{1}{m^2t^2}\int _{\frac{\delta }{2}}^{y-\frac{\delta }{2}} \frac{1}{m^{1+\mu }}|y-y_0|^{-\frac{3}{2}-\mu }Q_{2,m}\textrm{d}y_0 \\&\quad +\frac{1}{m^2t^2} \sum _{\sigma =0,1} \sum _{\kappa \in \lbrace +,- \rbrace } \int _{\frac{\delta }{2}}^{y-\frac{\delta }{2}} \chi _{m|y-y_0|\le 3\beta }\Big ( |\partial _y{\mathcal {B}}_{m,\varepsilon }^\kappa (y,y_0,\sigma )| + |\widetilde{{\mathcal {B}}_{m,\varepsilon }^\kappa }(y,y_0,\sigma )|\Big ) \textrm{d}y_0. \end{aligned}$$

Clearly, since $y\in (\delta ,1-\delta )$, we have that

$$\begin{aligned}  &   \int _{\frac{\delta }{2}}^{y-\frac{\delta }{2}} \frac{1}{m^{1+\mu }}|y-y_0|^{-\frac{3}{2}-\mu }Q_{2,m}\textrm{d}y_0\\  &   \quad \lesssim \frac{1}{m^{1+\mu }}\left[ (y-y_0)^{-\frac{1}{2}-\mu }\right] _{y_0=\frac{\delta }{2}}^{y_0=y-\frac{\delta }{2}}Q_{2,m}\lesssim \frac{1}{m^{1+\mu }} \delta ^{-\frac{1}{2}-\mu }Q_{2,m}. \end{aligned}$$

Similarly, Proposition 8.19 provides

$$\begin{aligned} \sum _{\sigma =0,1}\sum _{\kappa \in \lbrace +,- \rbrace }&\left\| \int _{\frac{\delta }{2}}^{y-\frac{\delta }{2}} \chi _{m|y-y_0|\le 3\beta } |\widetilde{{\mathcal {B}}_{m,\varepsilon }^\kappa }(y,y_0,\sigma )| \textrm{d}y_0 \right\| _{L^2_y(\delta ,1-\delta )} \\&\lesssim \sum _{\sigma =0,1}\sum _{\kappa \in \lbrace +,- \rbrace } \int _{\frac{\delta }{2}}^{1-\frac{\delta }{2}} \left\| \widetilde{{\mathcal {B}}_{m,\varepsilon }^\kappa }(y,y_0,\sigma )\right\| _{L^2_y(J_3)} \textrm{d}y_0 \\&\lesssim \left( m^{-1}\delta ^{-\frac{1}{2}} +m^{-\frac{1}{2}}\delta ^\frac{1}{2}+ m^\frac{1}{2}\right) Q_{1,m}, \end{aligned}$$

while Proposition 8.22 gives

$$\begin{aligned} \sum _{\sigma =0,1}\sum _{\kappa \in \lbrace +,- \rbrace } \int _{\frac{\delta }{2}}^{y-\frac{\delta }{2}} \left| \partial _y{{\mathcal {B}}_{m,\varepsilon }^\kappa }(y,y_0,\sigma )\right| \textrm{d}y_0 \lesssim \left( m^{\frac{1}{2}-\mu } + (m\delta )^{\frac{1}{2}-\mu } + m^{-\frac{1}{2}}\delta ^{-\frac{1}{2}+ \mu }\right) Q_{0,m}. \end{aligned}$$

Therefore, we have

$$\begin{aligned} \Vert {{\mathcal {T}}}_{1,3,1}\Vert _{L^2_y(\delta ,1-\delta )}\lesssim \frac{1}{m^2t^2}\left( m^\frac{1}{2} + m^{-\frac{1}{2}}(m\delta )^{-\frac{1}{2}-\mu } + m(m\delta )^{\frac{1}{2}-\mu } + (m\delta )^{-\frac{1}{2}}\right) Q_{2,m}. \end{aligned}$$

For ${{\mathcal {T}}}_{1,3,2}$, we use Minkowski inequality, Proposition 9.2 and Corollaries 8.13, 8.21 and 8.24 to estimate

$$\begin{aligned} \Vert {{\mathcal {T}}}_{1,3,2}\Vert _{L^2_y(\delta ,1-\delta )}&\lesssim \frac{1}{m^2t^2}\int _{\frac{\delta }{2}}^{1-\frac{\delta }{2}} \left( Q_{2,m} + m \sum _{\sigma =0,1}\sum _{\kappa \in \lbrace +, - \rbrace } \Vert {\mathcal {B}}_{m,\varepsilon }^\kappa (y,y_0,\sigma )\Vert _{L^2_y(J_2^c\cap J_3)} \right) \textrm{d}y_0 \\&\quad + \frac{1}{m^2t^2} \sum _{\sigma =0,1}\sum _{\kappa \in \lbrace +, - \rbrace } \int _{\frac{\delta }{2}}^{1-\frac{\delta }{2}} \Vert \partial _y{\mathcal {B}}_{m,\varepsilon }^\kappa (y,y_0,\sigma )\Vert _{L^2_y(J_2^c\cap J_3)} \textrm{d}y_0 \\&\quad + \frac{1}{m^2t^2} \sum _{\sigma =0,1}\sum _{\kappa \in \lbrace +, - \rbrace } \int _{\frac{\delta }{2}}^{1-\frac{\delta }{2}} \Vert \widetilde{B_{m,\varepsilon }^\kappa }(y,y_0,\sigma )\Vert _{L^2_y(J_2^c\cap J_3)} \textrm{d}y_0 \\&\lesssim \frac{1}{m^2t^2}\left( m^\frac{1}{2} + m^{-\frac{1}{2}}\delta ^\frac{1}{2} + m^{-1}\delta ^{-\frac{1}{2}}\right) Q_{2,m}. \end{aligned}$$

Hence, we conclude that

$$\begin{aligned} \Vert {{\mathcal {T}}}_{1,3}\Vert _{L^2_y(\delta ,1-\delta )} \lesssim \frac{1}{m^2 t^2}\left( m^\frac{1}{2} + m^{-\frac{1}{2}}(m\delta )^{-\frac{1}{2}-\mu } + m(m\delta )^{\frac{1}{2}-\mu } + m^{-\frac{1}{2}}\delta ^\frac{1}{2}\right) Q_{2,m} \end{aligned}$$

and thus

$$\begin{aligned} \Vert {{\mathcal {T}}}_1 \Vert _{L^2_y(\delta ,1-\delta )}\lesssim \frac{1}{mt}m^{-\frac{3}{2}}(m\delta )^{\frac{1}{2}}Q_{2,m} + \frac{1}{m^2 t^2}\left( m^\frac{1}{2} + m^{-\frac{1}{2}}(m\delta )^{-\frac{1}{2}-\mu }\right) Q_{2,m}. \end{aligned}$$

In particular, gathering the estimates for ${{\mathcal {T}}}_{2}$ and ${{\mathcal {T}}}_{1,i}$, for $i=1,2,3$, we obtain

$$\begin{aligned} \Vert \psi _m(t,y)\Vert _{L^2_y(\delta ,1-\delta )}\lesssim \frac{1}{m^2 t}(m\delta )^{\frac{1}{2}-\mu }Q_{2,m} + \frac{1}{m^2t^2}\left( m^\frac{1}{2} + m^{-\frac{1}{2}}(m\delta )^{-\frac{1}{2}-\mu }\right) Q_{2,m} \end{aligned}$$

$\bullet$ **Step 2.** For $y\in (0,\delta )$, we have that

$$\begin{aligned} \psi _m(t,y)=\frac{1}{2\pi i}\frac{1}{imt}\lim _{\varepsilon \rightarrow 0}\left( \int _0^{y+\frac{\delta }{2}} + \int _{y+\frac{\delta }{2}}^1 \right) e^{imy_0t}\left( \partial _{y_0}\psi _m^-(y,y_0) - \psi _m^+(y,y_0) \right) \textrm{d}y_0 = \widetilde{{{\mathcal {T}}}}_1 + \widetilde{{{\mathcal {T}}}}_2. \end{aligned}$$

One can see that the bounds for $\widetilde{{{\mathcal {T}}}}_2$ here are the same as the ones for ${{\mathcal {T}}}_3$, the procedure to obtain them is the same, we thus omit the details. On the other hand, for $\widetilde{{{\mathcal {T}}}}_1$ we argue as follows. Note that for $0\le y_0\le y+\frac{\delta }{2}$, we have that $|y-y_0|\le \delta \le \frac{3\beta }{m}$ and therefore we have from Proposition 9.1,

$$\begin{aligned} |\widetilde{{{\mathcal {T}}}}_1|\lesssim \frac{1}{mt}\int _0^{y+\frac{\delta }{2}}\left( \frac{1}{m^{1+\mu }}|y-y_0|^{-\frac{1}{2}-\mu }Q_{1,m} + \sum _{\sigma =0,1}\sum _{\kappa \in \lbrace +,- \rbrace }\left| {\mathcal {B}}_{m,\varepsilon }^\kappa (y,y_0,\sigma )\right| \right) \textrm{d}y_0. \end{aligned}$$

Since $y\in (0,\delta )$, we trivially have that

$$\begin{aligned} \frac{1}{mt}\int _0^{y+\frac{\delta }{2}} \frac{1}{m^{1+\mu }}|y-y_0|^{-\frac{1}{2}-\mu }Q_{1,m}\textrm{d}y_0\lesssim \frac{1}{mt}m^{-\frac{3}{2}}(m\delta )^{\frac{1}{2}-\mu }Q_{1,m}. \end{aligned}$$

Similarly, using the bounds from Proposition 8.11,

$$\begin{aligned} \sum _{\sigma =0,1}\sum _{\kappa \in \lbrace +,- \rbrace } \int _0^{y+\frac{\delta }{2}} \left| {\mathcal {B}}_{m,\varepsilon }^\kappa (y,y_0,\sigma )\right| \textrm{d}y_0&\lesssim \int _0^{y+\frac{\delta }{2}}\left( 1+ m^{-1+\mu }y_0^{-\frac{1}{2} + \mu }\right) \textrm{d}y_0 \\&\lesssim y+\tfrac{\delta }{2} + m^{-1+\mu }\left( y+\tfrac{\delta }{2}\right) ^{\frac{1}{2}+\mu }. \end{aligned}$$

As a result, we compute

$$\begin{aligned} \Vert \widetilde{{{\mathcal {T}}}}_1\Vert _{L^2_y(0,\delta )}\lesssim \frac{1}{mt}\left( m^{-2}(m\delta )^{1-\mu } + \delta ^\frac{3}{2} + m^{-2}(m\delta )^{1+\mu }\right) Q_{1,m} \lesssim \frac{1}{mt}m^{-\frac{3}{2}}(m\delta )^{1-\mu }Q_{1,m}, \end{aligned}$$

and thus we obtain

$$\begin{aligned} \Vert \psi _m(t)\Vert _{L^2_y(0,\delta )}\lesssim \frac{1}{mt}m^{-\frac{3}{2}}(m\delta )^\frac{1}{2}Q_{2,m} + \frac{1}{m^2t^2}\left( m^\frac{1}{2} + m^{-\frac{1}{2}}(m\delta )^{-\frac{1}{2}-\mu }\right) Q_{2,m}. \end{aligned}$$

The same bounds can be achieved for $\Vert \psi _m(t)\Vert _{L^2_y(1-\delta ,1)}$, the proof of which follows along the same ideas, we omit the details. With all these bounds, we are now able to estimate

$$\begin{aligned} \Vert \psi _m(t)\Vert _{L^2_y}&\lesssim \frac{1}{mt}m^{-1}(m\delta )^{\frac{1}{2}-\mu }Q_{2,m} + \frac{1}{m^2t^2}\left( m^\frac{1}{2} + m^{-\frac{1}{2}}(m\delta )^{-\frac{1}{2}-\mu }\right) Q_{2,m} \\&\lesssim m^{-\frac{3}{2}}t^{-\frac{3}{2}+\mu }Q_{2,m} \end{aligned}$$

once we choose $\delta =\frac{c_0}{mt}$, for $c_0=\frac{1}{1000}\min (\beta ,1)$. The proof is complete. $\square$

### Proposition 10.2

Let $$t\ge 1$$. Then,

$$\begin{aligned} \Vert \partial _y\psi _m(t)\Vert _{L^2_y}\lesssim m^{-\frac{1}{2}}t^{-\frac{1}{2} + \mu } Q_{1,m}. \end{aligned}$$

### Proof

The argument follows the same lines as the proof for $\Vert \psi _m(t,y)\Vert _{L^2_y}$. Hence, we shall only give the bounds for the terms involved in the computation. Their proof have already been carried out in the proof of Proposition 10.1.

$\bullet$ **Step 1.** For $y\in (\delta ,1-\delta )$ we shall write

$$\begin{aligned} \partial _y\psi _m(t,y)&=\frac{1}{2\pi i}\lim _{\varepsilon \rightarrow 0} \left( \int _0^{y-\frac{\delta }{2}} + \int _{y-\frac{\delta }{2}}^{y+\frac{\delta }{2}} + \int _{y+\frac{\delta }{2}}^1 \right) e^{-imy_0t}\left( \partial _y\psi _{m,\varepsilon }^-(y,y_0) - \partial _y\psi _{m,\varepsilon }^+(y,y_0)\right) \textrm{d}y_0 \\&={{\mathcal {I}}}_1 + {{\mathcal {I}}}_2 + {{\mathcal {I}}}_3 \end{aligned}$$

We begin by using Proposition 7.2 to bound

$$\begin{aligned} \left\| {{\mathcal {I}}}_2 \right\| _{L^2_y(\delta ,1-\delta )}\lesssim \left\| \int _{y-\frac{\delta }{2}}^{y+\frac{\delta }{2}} \frac{1}{m^{1+\mu }}|y-y_0|^{-\frac{1}{2}-\mu } \textrm{d}y_0 \right\| _{L^2_y(\delta ,1-\delta )}\lesssim m^{-\frac{3}{2}}(m\delta )^{\frac{1}{2}-\mu }Q_{0,m}. \end{aligned}$$

As before, for ${{\mathcal {I}}}_1$ we split it into

$$\begin{aligned} {{\mathcal {I}}}_1&= -\frac{1}{2\pi i} \frac{1}{imt}\lim _{\varepsilon \rightarrow 0} \left[ e^{-imy_0t}\left( \partial _y\psi _{m,\varepsilon }^-(y,y_0) - \partial _y\psi _{m,\varepsilon }^+(y,y_0)\right) \right] _{y_0=\frac{\delta }{2}}^{y_0=y-\frac{\delta }{2}} \\&\quad + \frac{1}{2\pi i}\lim _{\varepsilon \rightarrow 0}\int _0^{\frac{\delta }{2}} \textrm{e}^{-imy_0t}\partial _{y}\left( \psi _{m,\varepsilon }^-(y,y_0)- \psi _{m,\varepsilon }^+(y,y_0)\right) \textrm{d}y_0 \\&\quad + \frac{1}{2\pi i}\frac{1}{imt}\lim _{\varepsilon \rightarrow 0}\int _{\frac{\delta }{2}}^{y-\frac{\delta }{2}} \textrm{e}^{-imy_0t}\partial _{y,y_0}^2\left( \psi _{m,\varepsilon }^-(y,y_0)- \psi _{m,\varepsilon }^+(y,y_0)\right) \textrm{d}y_0 \\&=\mathcal {I}_{1,1}+ \mathcal {I}_{1,2}+\mathcal {I}_{1,3}. \end{aligned}$$

From Proposition 7.2 we see that

$$\begin{aligned} \Vert {{\mathcal {I}}}_{1,1}\Vert _{L^2_y(\delta ,1-\delta )}\lesssim \frac{1}{mt} m^{-\frac{1}{2}}(m\delta )^{-\frac{1}{2}-\mu }Q_{0,m}, \quad \Vert {{\mathcal {I}}}_{1,2}\Vert _{L^2_y(\delta ,1-\delta )}\lesssim m^{-\frac{3}{2}}(m\delta )^{\frac{1}{2}-\mu }Q_{0,m}. \end{aligned}$$

Similarly, from Proposition 9.1, we obtain

$$\begin{aligned} \Vert {{\mathcal {I}}}_{1,3}\Vert _{L^2_y(\delta ,1-\delta )}\lesssim \frac{1}{mt}\left( m^\frac{1}{2} + (m\delta )^{-\frac{1}{2}-\mu }\right) Q_{1,m}. \end{aligned}$$

The bounds for ${{\mathcal {I}}}_3$ are the same as the ones for ${{\mathcal {I}}}_1$, we omit the details. Recovering all terms, we conclude that

$$\begin{aligned} \Vert \partial _y\psi _m(t) \Vert _{L^2_y(\delta ,1-\delta )} \lesssim m^{-\frac{3}{2}}(m\delta )^{\frac{1}{2}-\mu }Q_{0,m} + \frac{1}{mt}\left( m^\frac{1}{2} + (m\delta )^{-\frac{1}{2}-\mu }\right) Q_{1,m}. \end{aligned}$$

$\bullet$ **Step 2.** For $y\in (0,\delta )$, we shall split now

$$\begin{aligned} \partial _y\psi _m(t,y)=\frac{1}{2\pi i}\lim _{\varepsilon \rightarrow 0}\left( \int _0^{y+\frac{\delta }{2}} + \int _{y+\frac{\delta }{2}}^1 \right) e^{-imy_0t}\left( \partial _{y}\psi _m^-(y,y_0) - \partial _y\psi _m^+(y,y_0) \right) \textrm{d}y_0 = \widetilde{{{\mathcal {I}}}}_1 + \widetilde{{{\mathcal {I}}}}_2. \end{aligned}$$

As before, the bound for $\widetilde{{{\mathcal {I}}}}_2$ is the same as the bound for ${{\mathcal {I}}}_3$. For $\widetilde{{{\mathcal {I}}}}_1$, note that $|y-y_0|\le \delta \le \frac{3\beta }{m}$ so that we shall use Proposition 7.2 to find that

$$\begin{aligned} \Vert \widetilde{{{\mathcal {I}}}}_1 \Vert _{L^2_y(0,\delta )}\lesssim m^{-2}(m\delta )^{1-\mu }Q_{0,m}. \end{aligned}$$

Gathering the previous bound, we obtain

$$\begin{aligned} \Vert \partial _y\psi _m(t) \Vert _{L^2_y}\lesssim m^{-\frac{3}{2}}(m\delta )^{\frac{1}{2}-\mu }Q_{0,m} + \frac{1}{mt}\left( m^\frac{1}{2} + (m\delta )^{-\frac{1}{2}-\mu }\right) Q_{1,m}. \end{aligned}$$

As before, the conclusion follows for $\delta =\frac{c_0}{mt}$, with $c_0=\frac{1}{1000}\min \left( \beta ,1\right)$. $\square$

We next obtain the decay rates for the perturbed density.

### Proposition 10.3

Let $$t\ge 1$$. Then,

$$\begin{aligned} \Vert \rho _m(t)\Vert _{L^2_y}\lesssim m^{-\frac{1}{2}}t^{-\frac{1}{2} + \mu } Q_{1,m}. \end{aligned}$$

### Proof

The demonstration also follows the same strategy as the proof for Proposition 10.1, we just present the main ideas and bounds.

$\bullet$ **Step 1.** For $y\in (\delta ,1-\delta )$ we write

$$\begin{aligned} \rho _m(t,y)&=\frac{1}{2\pi i}\lim _{\varepsilon \rightarrow 0} \left( \int _0^{y-\frac{\delta }{2}} + \int _{y-\frac{\delta }{2}}^{y+\frac{\delta }{2}} + \int _{y+\frac{\delta }{2}}^1 \right) e^{-imy_0t}\left( \rho _{m,\varepsilon }^-(y,y_0) - \rho _{m,\varepsilon }^+(y,y_0)\right) \textrm{d}y_0 \\&={{\mathcal {S}}}_1 + {{\mathcal {S}}}_2 + {{\mathcal {S}}}_3 \end{aligned}$$

We use Proposition 9.3 to bound

$$\begin{aligned} \Vert {{\mathcal {S}}}_2\Vert _{L^2_y(\delta ,1-\delta )}\lesssim m^{-\frac{3}{2}}(m\delta )^{\frac{1}{2}-\mu }. \end{aligned}$$

As before, both the bounds for ${{\mathcal {S}}}_3$ and ${{\mathcal {S}}}_1$ and the manner to show them are the same, we just comment on ${{\mathcal {S}}}_1$, which we split as follows.

$$\begin{aligned} {{\mathcal {S}}}_1&= -\frac{1}{2\pi i} \frac{1}{imt}\lim _{\varepsilon \rightarrow 0} \left[ \textrm{e}^{-imy_0t}\left( \rho _{m,\varepsilon }^-(y,y_0) - \rho _{m,\varepsilon }^+(y,y_0)\right) \right] _{y_0=\frac{\delta }{2}}^{y_0=y-\frac{\delta }{2}} \\&\quad + \frac{1}{2\pi i}\lim _{\varepsilon \rightarrow 0}\int _0^{\frac{\delta }{2}} \textrm{e}^{-imy_0t}\left( \rho _{m,\varepsilon }^-(y,y_0)- \rho _{m,\varepsilon }^+(y,y_0)\right) \textrm{d}y_0 \\&\quad + \frac{1}{2\pi i}\frac{1}{imt}\lim _{\varepsilon \rightarrow 0}\int _{\frac{\delta }{2}}^{y-\frac{\delta }{2}} \textrm{e}^{-imy_0t}\partial _{y_0}\left( \rho _{m,\varepsilon }^-(y,y_0)- \rho _{m,\varepsilon }^+(y,y_0)\right) \textrm{d}y_0 \\&=\mathcal {S}_{1,1}+ \mathcal {S}_{1,2}+\mathcal {S}_{1,3}. \end{aligned}$$

From Proposition 9.3 we easily deduce

$$\begin{aligned} \Vert {{\mathcal {S}}}_{1,1} \Vert _{L^2_y(\delta ,1-\delta )}\lesssim \frac{1}{mt} m^{-\frac{1}{2}}(m\delta )^{-\frac{1}{2}-\mu }Q_{0,m}, \quad \Vert {{\mathcal {S}}}_{1,2} \Vert _{L^2_y(\delta ,1-\delta )}\lesssim m^{-\frac{3}{2}}(m\delta )^{\frac{1}{2}-\mu }Q_{0,m}. \end{aligned}$$

On the other hand, Proposition 9.3 also yields

$$\begin{aligned} \Vert {{\mathcal {S}}}_{1,3} \Vert _{L^2_y(\delta ,1-\delta )}\lesssim \frac{1}{mt}\left( m^{\frac{1}{2}} + (m\delta )^{-\frac{1}{2}-\mu }\right) Q_{1,m}. \end{aligned}$$

Gathering the bounds we get

$$\begin{aligned} \Vert \rho _m(t) \Vert _{L^2_y(\delta ,1-\delta )}\lesssim m^{-\frac{3}{2}}(m\delta )^{\frac{1}{2}-\mu }Q_{0,m} + \frac{1}{mt}\left( m^{\frac{1}{2}} + (m\delta )^{-\frac{1}{2}-\mu }\right) Q_{1,m}. \end{aligned}$$

$\bullet$ **Step 2.** For $y\in (0,\delta )$ we shall now consider

$$\begin{aligned} \rho _m(t,y)=\frac{1}{2\pi i}\lim _{\varepsilon \rightarrow 0}\left( \int _0^{y+\frac{\delta }{2}} + \int _{y+\frac{\delta }{2}}^1 \right) e^{-imy_0t}\left( \rho _m^-(y,y_0) - \rho _m^+(y,y_0) \right) \textrm{d}y_0 = \widetilde{{{\mathcal {S}}}}_1 + \widetilde{{{\mathcal {S}}}}_2. \end{aligned}$$

The bounds for $\widetilde{{{\mathcal {S}}}_2}$ are the same as the ones for ${{\mathcal {S}}}_3$, we just focus on $\widetilde{{{\mathcal {S}}}_1}$. From Proposition 9.3, we see that

$$\begin{aligned} \Vert \widetilde{{{\mathcal {S}}}_1} \Vert _{L^2_y(0,\delta )}\lesssim m^{-2}(m\delta )^{1-\mu }Q_{0,m}. \end{aligned}$$

With this, it follows that

$$\begin{aligned} \Vert \rho _m(t)\Vert _{L^2_y(0,1)}\lesssim m^{-\frac{3}{2}}(m\delta )^{\frac{1}{2}-\mu }Q_{0,m} + \frac{1}{mt}\left( m^{\frac{1}{2}} + (m\delta )^{-\frac{1}{2}-\mu }\right) Q_{1,m} \end{aligned}$$

and thus the Proposition is proved once we choose $\delta =\frac{c_0}{mt}$, with $c_0=\frac{1}{1000}\min \left( \beta ,1\right)$.

$$\square $$

We next prove the inviscid damping decay estimates for the case $\beta ^2=1/4$. The precise bounds are recorded in the following proposition.

### Proposition 10.4

Let $$t\ge 1$$. Then,

$$\begin{aligned} \begin{aligned} \Vert \psi _m(t) \Vert _{L^2_y}&\lesssim m^{-\frac{3}{2}}t^{-\frac{3}{2}}(1 + \log (t) )\left( \Vert \rho _m^0 \Vert _{H^{4}_y} + \Vert {\omega }_m^0 \Vert _{H^{4}_y}\right) \\ \Vert \partial _y\psi _m(t)\Vert _{L^2_y}&\lesssim m^{-\frac{1}{2}}t^{-\frac{1}{2}}(1 + \log (t) )\left( \Vert \rho _m^0 \Vert _{H^{3}_y} + \Vert {\omega }_m^0 \Vert _{H^{3}_y}\right) \\ \Vert \rho _m(t)\Vert _{L^2_y}&\lesssim m^{-\frac{1}{2}}t^{-\frac{1}{2}}(1 + \log (t) )\left( \Vert \rho _m^0 \Vert _{H^{3}_y} + \Vert {\omega }_m^0 \Vert _{H^{3}_y}\right) . \end{aligned} \end{aligned}$$

### Proof

The proof follows along the same lines for the case $\beta ^2\ne 1/4$, the only difference is the logarithmic singularity present in the bounds of several quantities. For this, we note that for $m\delta < 1$,

$$\begin{aligned} \int _{y-\frac{\delta }{2}}^{y+\frac{\delta }{2}}\frac{1}{m}|y-y_0|^{-\frac{1}{2}}&\left( 1+ \big | \log \left( m|y-y_0|\right) \big | \right) \textrm{d}y_0 \\&\lesssim m^{-\frac{1}{2}}\int _{y-\frac{\delta }{2}}^{y+\frac{\delta }{2}}(m|y-y_0|)^{-\frac{1}{2}}\left( 1+ \big | \log \left( m|y-y_0|\right) \big | \right) \textrm{d}y_0 \\&\lesssim m^{-\frac{3}{2}} \int _{-m\frac{\delta }{2}}^{m\frac{\delta }{2}} |\eta |^{-\frac{1}{2}} (1 - \log |\eta |)\textrm{d}\eta \\&\lesssim m^{-\frac{3}{2}}(m\delta )^{\frac{1}{2}}\left( 1 + \big | \log \left( m\delta \right) \big | \right) . \end{aligned}$$

Here, we have used that, for $0<m\delta \le 1$,

$$\begin{aligned} -\int _0^{m\delta } \eta ^{-\frac{1}{2}}\log (\eta ) \textrm{d}\eta&= -\int _0^{m\delta } 2\partial _\eta (\eta ^\frac{1}{2})\log (\eta )\textrm{d}\eta = \left[ -2\eta ^\frac{1}{2}\log (\eta )\right] _{\eta =0}^{\eta =m\delta }+ 2\int _0^{m\delta } \eta ^{-\frac{1}{2}}\textrm{d}\eta \\&\lesssim (m\delta )^\frac{1}{2}\left( 1 + \big | \log \left( m\delta \right) \big |\right) . \end{aligned}$$

The same argument also yields

$$\begin{aligned} \int _{y-\frac{\delta }{2}}^{y+\frac{\delta }{2}}\frac{1}{m}|y-y_0|^{-\frac{3}{2}} \left( 1+ \big | \log \left( m|y-y_0|\right) \big | \right) \textrm{d}y_0\lesssim m^{-\frac{1}{2}}(m\delta )^{-\frac{1}{2}}\left( 1+ \big | \log \left( m\delta \right) \big | \right) . \end{aligned}$$

With this, the result follows thanks to the estimates obtained in Propositions 9.1-9.3, we omit the details. $\square$

Finally, Theorem 1 is a direct consequence of Propositions 10.1-10.4 together with Parseval identity.

## Properties of the Whittaker Functions

Here we state and prove some properties of the Whittaker functions that are used throughout the paper, we refer to [26] for a complete description of the Whittaker functions.

### Basic definitions and asymptotic expansions

For $\gamma , \zeta \in {{\mathbb {C}}}$, the Whittaker function $M_{0,\gamma }(\zeta )$ is given by

$$\begin{aligned} M_{0,\gamma }(\zeta )=e^{-\frac{1}{2}\zeta }\zeta ^{\frac{1}{2} + \gamma }M\left( \tfrac{1}{2} + \gamma , 1+2\gamma , \zeta \right) , \quad M(a,b,\zeta ) = \sum _{s=0}^\infty \frac{(a)_s}{(b)_s s!}\zeta ^s, \end{aligned}$$

where $(a)_s=a(a+1)(a+2)\dots (a+s-1)$ denotes the Pochhammer symbol. For $\gamma =0$, we also introduce

$$\begin{aligned} W_{0,0}(\zeta )=e^{-\frac{1}{2}\zeta }\sqrt{\frac{\zeta }{\pi }}\sum _{s=0}^\infty \frac{\left( \tfrac{1}{2} \right) _s}{(s!)^2}\zeta ^s \left( 2\frac{\Gamma '(1+s)}{\Gamma (1+s)} - \frac{\Gamma '(\tfrac{1}{2}+s)}{\Gamma (\tfrac{1}{2}+s)} - \log (\zeta )\right) , \end{aligned}$$

where $\Gamma (x)$ denotes the Gamma function.

We recall that $\mu =\textrm{Re}\left( \sqrt{1/4-\beta ^2}\right)$ and $\nu =\textrm{Im}\left( \sqrt{1/4-\beta ^2}\right)$, and set $\gamma =\mu +i\nu$. We begin by recording some basic properties regarding complex conjugation for $M_{0,\gamma }(\zeta )$, which can be deduce from the series definition of $M_{0,\gamma }$ and $W_{0,0}$.

#### Lemma A.1

We have the following

- For $$\beta ^2>1/4$$, then $$M_{0,i\nu }(\zeta )=\overline{M_{0,-i\nu }\left( \overline{\zeta }\right) }$$.
- For $\beta ^2\le 1/4$, then $M_{0,\mu }(\zeta ) = \overline{M_{0,\mu }\left( \overline{\zeta }\right) }$. Additionally, for $x\in {{\mathbb {R}}}$ then $M_{0,\mu }(x),W_{0,0}(x)\in {{\mathbb {R}}}$.

We next state an analytic continuation property, which is key in studying the Wronskian of the Green’s function and is directly determined by the analytic continuation of the nonentire term of $M_{0,\gamma }(\zeta )$, which is $\zeta ^{\frac{1}{2}+\gamma }$, for $\zeta \in {{\mathbb {C}}}$.

#### Lemma A.2

[26] Let $$\beta ^2>0$$. Then

$$\begin{aligned} M_{0,\gamma }(\zeta \textrm{e}^{\pm \pi i}) = \pm i \textrm{e}^{\pm \gamma \pi i}M_{0,\gamma }(\zeta ), \quad W_{0,0}(\zeta e^{\pm i\pi })=\sqrt{\pi }M_{0,0}(\zeta ) \pm iW_{0,0}(\zeta ) \end{aligned}$$

for all $$\zeta \in {{\mathbb {C}}}$$.

The next result gives a precise description of the asymptotic expansion of $M_\pm (\zeta )$ and its derivatives, for $\zeta$ in a bounded domain.

#### Lemma A.3

Let $\zeta \in {{\mathbb {C}}}$. Let $B_R\subset {{\mathbb {C}}}$ denote the closed unit ball of radius $R>0$ centered in the origin. Then,

$$\begin{aligned} \begin{aligned} M_{0,\pm \gamma }(\zeta )&=\zeta ^{\frac{1}{2} \pm \gamma }\mathcal {E}_{0,\pm \gamma }(\zeta ),\quad M_{0,\pm \gamma }'(\zeta )=\zeta ^{-\frac{1}{2} \pm \gamma }\mathcal {E}_{1,\pm \gamma }(\zeta ), \end{aligned} \end{aligned}$$

where $\mathcal {E}_{j,\pm \gamma }\in L^\infty (B_R)$ and $\Vert \mathcal {E}_{j,\pm \gamma }\Vert _{L^\infty (B_R)} \lesssim _{\gamma ,R} 1$, for all $j\in \left\{ 0,1,2 \right\}$.

Moreover, for $R_m:=\frac{R}{2m}$ and $M_{\pm }(\zeta )=M_{0,\pm \gamma }(2m\zeta )$, let $B_{R_m}\subset {{\mathbb {C}}}$ denote the closed ball centered in the origin of radius $R_m$. We have that

$$\begin{aligned} \begin{aligned} M_\pm (\zeta )&=\zeta ^{\frac{1}{2} \pm \gamma }\mathcal {E}_{m,0,\pm \gamma }(\zeta ),\quad M_\pm '(\zeta )=\zeta ^{-\frac{1}{2} \pm \gamma }\mathcal {E}_{m,1,\pm \gamma }(\zeta ), \end{aligned} \end{aligned}$$

where $\mathcal {E}_{m,j,\pm \gamma }\in L^\infty (B_{R_m})$ and $\Vert \mathcal {E}_{m,j,\pm \gamma }\Vert _{L^\infty (B_{R_m})} \lesssim _\gamma (2\,m)^{\frac{1}{2}\pm \mu }$, for all $j\in \left\{ 0,1,2 \right\}$.

#### Proof

Firstly, from [26] we know that

$$\begin{aligned} \begin{aligned} M_{0,\pm \gamma }(\zeta )&=\textrm{e}^{-\frac{1}{2}\zeta } \zeta ^{\frac{1}{2} \pm \gamma } M\left( \frac{1}{2} \pm \gamma , 1\pm 2\gamma ,\zeta \right) =\zeta ^{\frac{1}{2} \pm \gamma }\mathcal {E}_{0,\pm \gamma }(\zeta ), \end{aligned} \end{aligned}$$

where $\mathcal {E}_{0,\pm \gamma }(\zeta )$ is entire and $\Vert \mathcal {E}_{0,\pm \gamma }\Vert _{L^\infty (B_R)} \lesssim _{\gamma ,R} 1$. On the other hand, note that

A.1 $$\begin{aligned} M_{0,\pm \gamma }'(\zeta )=-\frac{1}{2}M_{0,\pm \gamma }(\zeta )+\left( \frac{1}{2}\pm \gamma \right) \frac{M_{0,\pm \gamma }(\zeta )}{\zeta }+\frac{1}{2}\zeta ^{-\frac{1}{2}}M_{-\frac{1}{2},\frac{1}{2}\pm \gamma }(\zeta ), \end{aligned}$$

where further

$$\begin{aligned} M_{-\frac{1}{2},\frac{1}{2}\pm \gamma }(\zeta )=\textrm{e}^{-\frac{1}{2}\zeta }\zeta ^{1\pm \gamma }M\left( \frac{3}{2}\pm \gamma ,2\pm 2\gamma ,\zeta \right) =\zeta ^{1\pm \gamma }\mathcal {H}_{\pm \gamma }(\zeta ), \end{aligned}$$

with $\mathcal {H}_{\pm \gamma }(\zeta )$ entire and thus uniformly bounded in $B_R$. Hence,

$$\begin{aligned} M_{0,\pm \gamma }'(\zeta )=\zeta ^{-\frac{1}{2}-\gamma }\left( \left( \frac{1}{2}\pm \gamma \right) \mathcal {E}_{0,\pm \gamma }(\zeta )+ \frac{1}{2} \zeta \left( \mathcal {H}_{\pm \gamma }(\zeta ) - \mathcal {E}_{0,\pm \gamma }(\zeta )\right) \right) = \zeta ^{-\frac{1}{2}-\gamma }\mathcal {E}_{1,\pm \gamma }(\zeta ), \end{aligned}$$

with $\Vert \mathcal {E}_{1,\pm \gamma }(\zeta ) \Vert _{L^\infty (B_R)}\lesssim _{\gamma ,R} 1$. The formulas and bounds for $M_\pm (\zeta )=M_{0,\pm \gamma }(2m\zeta )$ and its derivatives follow from those for $M_{0,\pm \gamma }$, the chain rule and the observation that $2m\zeta \in B_R$ provided that $\zeta \in B_{R_m}$. $\square$

#### Lemma A.4

Let $\beta ^2=1/4$ and $\zeta \in {{\mathbb {C}}}$. Let $B_R\subset {{\mathbb {C}}}$ denote the closed ball of radius $R>0$ centered at the origin. Then,

$$\begin{aligned} W_{0,0}(\zeta ) = \zeta ^\frac{1}{2} \big ( {{\mathcal {E}}}_{0,1}(\zeta ) - \log (\zeta ) {{\mathcal {E}}}_{0,2}(\zeta )\big ), \quad W_{0,0}'(\zeta ) = \zeta ^{-\frac{1}{2}} \big ( {{\mathcal {E}}}_{1,1}(\zeta ) - \log (\zeta ) {{\mathcal {E}}}_{1,2}(\zeta )\big ), \end{aligned}$$

where ${{\mathcal {E}}}_{j,k}(\zeta )$ are entire functions in ${{\mathbb {C}}}$ and $\Vert {{\mathcal {E}}}_{j,k}\Vert _{L^\infty (B_R)}\lesssim 1$, for $j=0,1$ and $k=1,2$.

#### Proof

We begin with noting that $W_{0,0}(2\zeta )=\sqrt{\frac{2\zeta }{\pi }}K_0(\zeta )$, where $K_0(\cdot )$ is the modified Bessel function of second kind of order 0. Moreover, we have that

$$\begin{aligned} K_0(\zeta )=-\left( \ln \left( \frac{\zeta }{2}\right) + \varsigma \right) I_0(\zeta ) +2\sum _{k=1}^\infty \frac{I_{2k}(\zeta )}{k}, \end{aligned}$$

where

$$\begin{aligned} I_{2k}(\zeta )=\left( \frac{\zeta }{2}\right) ^{2k}\sum _{j\ge 0}\frac{\left( \frac{\zeta ^2}{4}\right) ^j}{j!(2k+j)!}. \end{aligned}$$

Here, $I_j(\zeta )$ denotes the modified Bessel function of first kind of order $j\in {{\mathbb {N}}}$. In particular, one observes that $|I_{2k}(\zeta )|\le I_{2k}(|\zeta |)$. Additionally, since $\cosh (|\zeta |) = I_0(|\zeta |) + 2\sum _{k=1}^\infty I_{2k}(|\zeta |)$, see [26], we can bound

$$\begin{aligned} \left| 2\sum _{k=1}^\infty \frac{I_{2k}(z)}{k} \right| \le 2 \sum _{k=1}^\infty I_{2k}(|\zeta |)= \cosh (|\zeta |) -I_0(|\zeta |)< \cosh (|\zeta |). \end{aligned}$$

Therefore, since $I_j(\zeta )$ is analytic in ${{\mathbb {C}}}$ for all $j\in {{\mathbb {N}}}$ and $\frac{1}{2}\zeta \in B_R$, we can write

$$\begin{aligned} W_{0,0}(\zeta ) = \zeta ^\frac{1}{2} \big ( {{\mathcal {E}}}_{0,1}(\zeta ) - \log (\zeta ) {{\mathcal {E}}}_{0,2}(\zeta )\big ), \end{aligned}$$

where

$$\begin{aligned} {{\mathcal {E}}}_{0,1}(\zeta )=\left( \log (2) - \varsigma \right) I_0(\zeta ) + 2\sum _{k=1}^{+\infty }\frac{I_{2k}(\zeta )}{k}, \quad {{\mathcal {E}}}_{0,2}(\zeta ) = I_0(\zeta ) \end{aligned}$$

and they are such that $\Vert {{\mathcal {E}}}_{0,j}(\zeta )\Vert _{L^\infty (B_R)}\lesssim 1$, for $j=1,2$.

For $W_{0,0}'(\zeta )$, note that $W_{0,0}'(\zeta )=\frac{1}{2\sqrt{\pi \zeta }}\left( K_0(\zeta /2)+\zeta K_0'(\zeta /2)\right)$. As before, we can write

$$\begin{aligned} K_0'(\zeta )=-K_1(\zeta )= -\left[ \frac{1}{\zeta }I_0(\zeta ) + \left( \log \left( \frac{1}{2}\zeta \right) + \varsigma -1 \right) I_1(\zeta ) - \sum _{k\ge 1}\frac{(1+2k)}{k(1+k)}I_{1+2k}(\zeta )\right] . \end{aligned}$$

Since $\sinh (\zeta ) = I_1(\zeta ) + 2\sum _{k\ge 1}I_{1+2k}(\zeta )$, confer [26], we bound

$$\begin{aligned} \left| \sum _{k\ge 1}\frac{(1+2k)}{k(1+k)}I_{1+2k}(\zeta )\right| \le \sinh (|\zeta |)-I_1(|\zeta |)\le \sinh (|\zeta |). \end{aligned}$$

and we conclude the existence of two entire functions ${{\mathcal {E}}}_{1,1}(\zeta )$ and ${{\mathcal {E}}}_{1,2}(\zeta )$ such that $\Vert {{\mathcal {E}}}_{1,j}(\zeta )\Vert _{L^\infty (B_R)}\lesssim 1$, for $j=1,2$ and for which

$$\begin{aligned} W_{0,0}'(\zeta )=\zeta ^{-\frac{1}{2}}\big ( {{\mathcal {E}}}_{1,1}(\zeta ) -\log (\zeta ){{\mathcal {E}}}_{0,2}(\zeta )\big ). \end{aligned}$$

$$\square $$

### Lower bounds for Whittaker functions

The next lemma shows that there are no zeroes of $M_+(x)$, for any $x\in (0,\infty )$.

#### Lemma A.5

Let $x>0$. We have the following.

- For $\beta ^2\le 1/4$, then $M_{0,\mu }(x)$ is monotone increasing and
  $$\begin{aligned} M_{0,\mu }(x)>x^{\frac{1}{2}+\mu }, \quad M\left( \tfrac{1}{2} + \mu ,1+2\mu ,x \right) \ge e^{\frac{1}{2} x}. \end{aligned}$$
- For $\beta ^2>1/4$, then $|M_{0,i\nu }(x)|$ is monotone increasing and
  $$\begin{aligned} x|\Gamma (1+i\nu )|^2\frac{\sinh (\nu \pi )}{\nu \pi }\le |M_{0,i\nu }(x)|^2 \le x\cosh (x)|\Gamma (1+i\nu )|^2\frac{\sinh (\nu \pi )}{\nu \pi }, \end{aligned}$$
  with also
  $$\begin{aligned} \left| M\left( \tfrac{1}{2} +i\nu , 1+ 2i\nu , x \right) \right| \ge \textrm{e}^{\frac{1}{2}x}|\Gamma (1+i\nu )|\sqrt{\frac{\sinh (\nu \pi )}{\nu \pi }}. \end{aligned}$$

#### Proof

From [26], we have

$$\begin{aligned} M_{0,\gamma }\left( 2x\right) =2^{2\gamma +\frac{1}{2}}\Gamma \left( 1+\gamma \right) \sqrt{x}I_{\gamma }\left( x\right) . \end{aligned}$$

For $\beta ^2\le 1/4$, we have $\gamma =\mu$ and the conclusion is straightforward, since we can use the power series representation of $I_\mu (x)$ to obtain

$$\begin{aligned} M_{0,\mu }(2x)>(2x)^{\frac{1}{2}+\mu }. \end{aligned}$$

On the other hand, for $\beta ^2> 1/4$, we have $\gamma =i\nu$ and

$$\begin{aligned} M_{0,i\nu }\left( 2x\right) =2^{2i\nu +\frac{1}{2}}\Gamma \left( 1+i\nu \right) \sqrt{x}I_{i\nu }\left( x\right) . \end{aligned}$$

Therefore,

$$\begin{aligned} |M_{0,i\nu }(2x)|^2= &   2x|\Gamma (1+i\nu )|^2I_{i\nu }(x)I_{-i\nu }(x)=2x|\Gamma (1+i\nu )|^2 \frac{2}{\pi }\\  &   \int _0^{\frac{\pi }{2}} I_0(2x\cos \theta )\cosh (2\nu \theta )\textrm{d}\theta \end{aligned}$$

The upper and lower bound follow from the fact that $1 \le I_0(x) \le \cosh (x)$, for all $x\ge 0$. See [26] for the product formula for $I_{i\nu }(x)I_{-i\nu }(x)$. $\square$

### Growth bounds and comparison estimates for $\beta ^2>1/4$

In this subsection we treat the case $\beta ^2>1/4$, so that $\mu =0$ and $\nu =\sqrt{\beta ^2-1/4}$.

#### Lemma A.6

Denote $a:=\frac{1}{2} + i\nu$ and $b:=2a$. Then, there exists $C>0$ and $N_{\nu ,0}>0$ such that

$$\begin{aligned} \textrm{e}^{-\frac{1}{8}\nu \pi }\textrm{e}^{\frac{1}{2}\textrm{Re}\zeta }\le \left| \frac{\Gamma (a)}{\Gamma (b)}M_+(\zeta )\right| \le \textrm{e}^{\frac{1}{8}\nu \pi }\textrm{e}^{\frac{1}{2}\textrm{Re}\zeta }, \end{aligned}$$

and

$$\begin{aligned} \left| \frac{M_\pm '(\zeta )}{M_\pm (\zeta )}-\frac{1}{2}\right| \le \frac{1}{4}, \end{aligned}$$

for all $$\textrm{Re}\zeta \ge N_{\nu ,0}$$.

#### Proof

Let $\zeta \in {{\mathbb {C}}}$. We recall that

$$\begin{aligned} \begin{aligned} M_+(\zeta )&=\textrm{e}^{-\frac{1}{2}\zeta }\zeta ^aM(a,b,\zeta ) =\textrm{e}^{-\frac{1}{2}\zeta }\zeta ^a\frac{\Gamma (b)}{\Gamma (a)}\left( \textrm{e}^{-i\pi a}U(a,b,\zeta ) + \textrm{e}^{i\pi a}\textrm{e}^\zeta U(a,b,\textrm{e}^{i\pi } \zeta )\right) . \end{aligned} \end{aligned}$$

Moreover, we have that $U(a,b,\zeta ) = \zeta ^{-a}+ \mathcal {E}_1(\zeta )$, where further

$$\begin{aligned} |\zeta ^a{{\mathcal {E}}}_1(\zeta )|\le \frac{2\beta ^2}{|\zeta |}\textrm{e}^{\frac{2\beta ^2}{|\zeta |}}. \end{aligned}$$

In the sequel, we write $x:=\frac{2\beta ^2}{|\zeta |}$. Therefore, we can write

A.2 $$\begin{aligned} M_+(\zeta )=\frac{\Gamma (b)}{\Gamma (a)}\textrm{e}^{\frac{1}{2}\zeta }\left( \left[ 1 + (\textrm{e}^{i\pi }\zeta )^a{{\mathcal {E}}}_1(\textrm{e}^{i\pi }\zeta )\right] + \textrm{e}^{-\zeta }\textrm{e}^{-i\pi a}\left[ 1+ \zeta ^a{{\mathcal {E}}}_1(\zeta )\right] \right) \end{aligned}$$

We shall focus on obtaining upper and lower bound estimates for

$$\begin{aligned} \left| \left[ 1 + (\textrm{e}^{i\pi }\zeta )^a{{\mathcal {E}}}_1(\textrm{e}^{i\pi }\zeta )\right] + \textrm{e}^{-\zeta }\textrm{e}^{-i\pi a}\left[ 1+ \zeta ^a{{\mathcal {E}}}_1(\zeta )\right] \right| \end{aligned}$$

when $\textrm{Re}\zeta$ is large. To this end, we note that $|\zeta ^a{{\mathcal {E}}}_1(\zeta )|\le 2x$, for $x\le \frac{1}{2}$. Moreover,

$$\begin{aligned} |1+\zeta ^a{{\mathcal {E}}}_1(\zeta )| \le \textrm{e}^{\nu \frac{\pi }{16}}, \end{aligned}$$

provided that $x\le \frac{1}{2}\min \left\{ 1, \textrm{e}^{\nu \frac{\pi }{16}}-1\right\}$. Similarly, we also have that

$$\begin{aligned} 1+\textrm{e}^{-\zeta } \textrm{e}^{\nu \pi }\le \textrm{e}^{\nu \frac{\pi }{16}}, \end{aligned}$$

for all $\textrm{Re}\zeta > \nu \pi -\log (\textrm{e}^{\frac{\nu \pi }{16}}-1)$. Hence,

$$\begin{aligned} \left| \left[ 1 + (\textrm{e}^{i\pi }\zeta )^a{{\mathcal {E}}}_1(\textrm{e}^{i\pi }\zeta )\right] + \textrm{e}^{-\zeta }\textrm{e}^{-i\pi a}\left[ 1+ \zeta ^a{{\mathcal {E}}}_1(\zeta )\right] \right| \le \textrm{e}^{\frac{\nu \pi }{8}}. \end{aligned}$$

On the other hand, for $x\le \min \left\{ \frac{1}{4}\left( 1- \textrm{e}^{-\frac{1}{8}\nu \pi }\right) , \frac{1}{2}\right\}$, we have that

$$\begin{aligned} |\zeta ^a{{\mathcal {E}}}_1(\zeta )| \le \frac{1}{2}\left( 1-\textrm{e}^{-\nu \frac{\pi }{8}}\right) , \end{aligned}$$

and also

$$\begin{aligned} \left| \textrm{e}^{-\zeta }\textrm{e}^{\nu \pi }\left( 1+ \zeta ^a{{\mathcal {E}}}_1(\zeta )\right) \right| \le \frac{1}{2}\left( 1-\textrm{e}^{-\nu \frac{\pi }{8}}\right) , \end{aligned}$$

provided that $\textrm{Re}\zeta >\nu \pi +\log \left( \frac{4}{1-\textrm{e}^{-\frac{1}{8}\nu \pi }}\right)$. Therefore, we can lower bound

$$\begin{aligned} \left| \left[ 1 + (\textrm{e}^{i\pi }\zeta )^a{{\mathcal {E}}}_1(\textrm{e}^{i\pi }\zeta )\right] + \textrm{e}^{-\zeta }\textrm{e}^{-i\pi a}\left[ 1+ \zeta ^a{{\mathcal {E}}}_1(\zeta )\right] \right| \ge \textrm{e}^{-\frac{\nu \pi }{8}}. \end{aligned}$$

We choose $N_\nu >0$ so that all the above conditions are satisfied when $\textrm{Re}\zeta \ge N_\nu$. For the second part of the lemma, we take a $\frac{\textrm{d}}{\textrm{d}\zeta }$ derivative in (A.2) to obtain

$$\begin{aligned} \begin{aligned} M_+'(\zeta )&=\frac{1}{2}M_+(\zeta ) + \frac{\Gamma (b)}{\Gamma (a)}\textrm{e}^{\frac{1}{2}\zeta }\left( \frac{a}{\zeta }(\textrm{e}^{i\pi }\zeta )^a{{\mathcal {E}}}_1(\textrm{e}^{i\pi }\zeta ) + \textrm{e}^{i\pi }(\textrm{e}^{i\pi }\zeta )^a{{\mathcal {E}}}_1'(\textrm{e}^{i\pi }\zeta )\right) \\&\quad + \frac{\Gamma (b)}{\Gamma (a)}\textrm{e}^{-\frac{1}{2}\zeta }\textrm{e}^{i\pi a}\left( \frac{a}{\zeta }\zeta ^a{{\mathcal {E}}}_1(\zeta ) + \zeta ^a{{\mathcal {E}}}_1'\zeta ) -1 -\zeta ^a{{\mathcal {E}}}_1(\zeta )\right) . \end{aligned} \end{aligned}$$

Since $|\zeta ^a{{\mathcal {E}}}_1(\zeta )|\le \frac{2\beta ^2}{|\zeta |}\textrm{e}^{\frac{2\beta ^2}{|\zeta |}}$ and $|\zeta ^a{{\mathcal {E}}}_1'(\zeta )|\le \frac{4\beta ^2}{|\zeta |}\textrm{e}^{\frac{2\beta ^2}{|\zeta |}}$, confer [26], we find that

$$\begin{aligned} \left| \frac{a}{\zeta }(\textrm{e}^{i\pi }\zeta )^a{{\mathcal {E}}}_1(\textrm{e}^{i\pi }\zeta ) + \textrm{e}^{i\pi }(\textrm{e}^{i\pi }\zeta )^a{{\mathcal {E}}}_1'(\textrm{e}^{i\pi }\zeta ) \right| \le \left( \frac{|a|}{|\zeta |}+2\right) \frac{2\beta ^2}{|\zeta |}\textrm{e}^{\frac{2\beta ^2}{|\zeta |}}\le 6x, \end{aligned}$$

and

$$\begin{aligned} \left| \textrm{e}^{i\pi a}\left( \frac{a}{\zeta }\zeta ^a{{\mathcal {E}}}_1(\zeta ) + \zeta ^a{{\mathcal {E}}}_1'\zeta ) -1 -\zeta ^a{{\mathcal {E}}}_1(\zeta )\right) \right| \le \left( 1 + \left( \frac{|a|}{|\zeta |}+3\right) \frac{2\beta ^2}{|\zeta |}\textrm{e}^{\frac{2\beta ^2}{|\zeta |}} \right) \le 5, \end{aligned}$$

for $|\zeta |\ge |a|$ and $x\le \frac{1}{2}$. Therefore,

$$\begin{aligned} \begin{aligned} \left| \frac{M_+'(\zeta )}{M_+(\zeta )}-\frac{1}{2}\right|&\le \left| \frac{\frac{a}{\zeta }(\textrm{e}^{i\pi }\zeta )^a{{\mathcal {E}}}_1(\textrm{e}^{i\pi }\zeta ) + \textrm{e}^{i\pi }(\textrm{e}^{i\pi }\zeta )^a{{\mathcal {E}}}_1'(\textrm{e}^{i\pi }\zeta )}{1 + (\textrm{e}^{i\pi }\zeta )^a{{\mathcal {E}}}_1(\textrm{e}^{i\pi }\zeta ) + \textrm{e}^{-\zeta }\textrm{e}^{-i\pi a}\left( 1+ \zeta ^a{{\mathcal {E}}}_1(\zeta )\right) }\right| \\&+ \textrm{e}^{-\textrm{Re}(\zeta )}\left| \frac{\textrm{e}^{i\pi a}\left( \frac{a}{\zeta }\zeta ^a{{\mathcal {E}}}_1(\zeta ) + \zeta ^a{{\mathcal {E}}}_1'\zeta ) -1 -\zeta ^a{{\mathcal {E}}}_1(\zeta )\right) }{1 + (\textrm{e}^{i\pi }\zeta )^a{{\mathcal {E}}}_1(\textrm{e}^{i\pi }\zeta ) + \textrm{e}^{-\zeta }\textrm{e}^{-i\pi a}\left( 1+ \zeta ^a{{\mathcal {E}}}_1(\zeta )\right) }\right| , \end{aligned} \end{aligned}$$

which can be made arbitrarily small due to the previous bounds for $\textrm{Re}(\zeta )$ sufficiently large. $\square$

#### Lemma A.7

Let $y_0\in [0,1]$ such that $2my_0\le N_{\nu ,0}$. Then, there exists $\varepsilon _0>0$ such that

$$\begin{aligned} \left| \frac{M_+(y_0-i\varepsilon )}{M_+(y_0+i\varepsilon )}\right| \le \textrm{e}^{\frac{5}{4}\nu \pi }, \end{aligned}$$

for all $$\varepsilon \le \varepsilon _0$$.

#### Proof

Let $\theta =\arg (y_0-i\varepsilon )\in \left[ -\frac{\pi }{2}, 0\right]$. Recall that for $a=\frac{1}{2}+i\nu$ and $b=2a$, for $\zeta \in {{\mathbb {C}}}$,

$$\begin{aligned} \begin{aligned} M_+(\zeta )&=\textrm{e}^{-\frac{1}{2}\zeta }\zeta ^aM(a,b,\zeta ). \end{aligned} \end{aligned}$$

Therefore, we can estimate

$$\begin{aligned} \left| \frac{M_+(y_0-i\varepsilon )}{M_+(y_0+i\varepsilon )}\right| = \left| \textrm{e}^{-i\varepsilon }\textrm{e}^{i\theta }\textrm{e}^{-2\nu \theta } \frac{M(a,b,2m(y_0-i\varepsilon ))}{M(a,b,2m(y_0+i\varepsilon ))}\right| \le \textrm{e}^{\nu \pi }\left| \frac{M(a,b,2m(y_0-i\varepsilon ))}{M(a,b,2m(y_0+i\varepsilon ))}\right| . \end{aligned}$$

Now, since $\frac{\textrm{d}}{\textrm{d}\zeta }M(a,b,\zeta )=\frac{1}{2}M(a+1,b+1,\zeta )$, which is entire in $\zeta \in {{\mathbb {C}}}$, we have that

$$\begin{aligned} M(a,b,2m(y_0+i\varepsilon ))=M(a,b,2my_0) + \int _{0}^{\varepsilon }imM(a+1,b+1,2m(y_0+is))\textrm{d}s. \end{aligned}$$

We can further bound the error term by noting that $|2\,m(y_0+is)|\le N_\nu +10\beta ^2$, for all $|s|\le |\varepsilon |$. As a result, there exists $C_\nu$ such that $|M(a+1,b+1,2m(y_0+is))|\le C_\nu$, for all $|s|\le |\varepsilon |$. Therefore,

$$\begin{aligned} \begin{aligned}&\left| \int _{0}^{\varepsilon }imM(a+1,b+1,2m(y_0+is))\textrm{d}s\right| \\&\quad \le |M(a,b,2my_0)|\frac{C_\nu m|\varepsilon |}{|M(a,b,2my_0)|} \\&\quad \le C_\nu |M(a,b,2my_0)|\textrm{e}^{-my_0}\sqrt{\frac{\nu \pi \cosh \nu \pi }{\sinh \nu \pi }}m|\varepsilon | \\&\quad \le (1-\textrm{e}^{-\frac{1}{8}\nu \pi })|M(a,b,2my_0)|, \end{aligned} \end{aligned}$$

for all $0\le |\varepsilon |\le \varepsilon _0=\frac{1-\textrm{e}^{-\frac{1}{8}\nu \pi }}{m}\sqrt{\frac{\sinh \nu \pi }{\nu \pi \cosh \nu \pi }} C_\nu$. Consequently, we have that

$$\begin{aligned} \left| \frac{M(a,b,2m(y_0-i\varepsilon ))}{M(a,b,2m(y_0+i\varepsilon ))}\right| \le \textrm{e}^{\frac{1}{4}\nu \pi }. \end{aligned}$$

$$\square $$

#### Lemma A.8

Let $N_{\nu ,0}$ be given as above and $N_{\nu ,1}>0$. Let $\sigma \in \lbrace +,-\rbrace$. If $N_{\nu ,1}< N_{\nu ,0}$, then, there exists $\varepsilon _0>0$ such that

$$\begin{aligned} |M_\sigma (y_0)|\textrm{e}^{-\frac{1}{8}\nu \pi } \le |M_\sigma (y_0+i\varepsilon )|\le |M_\sigma (y_0)|\textrm{e}^{\frac{1}{8}\nu \pi }, \end{aligned}$$

for all $y_0\in [0,1]$ such that $N_{\nu ,1}\le 2my_0 \le N_{\nu ,0}$, and all $0\le |\varepsilon |\le \varepsilon _0$.

#### Proof

The result follows from the Fundamental Theorem of Calculus, the asymptotic expansions of $M_\sigma$ and $M_\sigma '$ for small arguments from Lemma tal and the lower bounds on $|M_\sigma |$ from Lemma Qual. More precisely, assume without loss of generality that $0\le \varepsilon$ and note that

$$\begin{aligned} M_\sigma (y_0+i\varepsilon )=M_\sigma (y_0) + \int _0^\varepsilon \frac{\textrm{d}}{\textrm{d}s}M_\sigma (y_0+is)\textrm{d}s. \end{aligned}$$

Thanks to the asymptotic expansions for small arguments we next estimate

$$\begin{aligned} \left| \int _0^\varepsilon \frac{\textrm{d}}{\textrm{d}s}M_\sigma (y_0+is)\textrm{d}s \right| \le \int _0^\varepsilon |M_\sigma '(y_0+is)| \textrm{d}s \le C_\nu \int _0^\varepsilon \frac{(2m)^\frac{1}{2}}{|y_0+is|^\frac{1}{2}}\textrm{d}s \le C_\nu (2m\varepsilon )(2my_0)^{-\frac{1}{2}}. \end{aligned}$$

Using the lower bound $(2my_0)^{\frac{1}{2}}\le \sqrt{\frac{\nu \pi \cosh \nu \pi }{\sinh \nu \pi }}|M_\sigma (y_0)|$ we have that

$$\begin{aligned} \left| \int _0^\varepsilon \frac{\textrm{d}}{\textrm{d}s}M_\sigma (y_0+is)\textrm{d}s \right| \le C_\nu |M_\sigma (y_0)|\frac{\varepsilon }{y_0}\le C_\nu N_{\nu ,2}^{-1}2m\varepsilon . \end{aligned}$$

We now choose $\varepsilon _0=\frac{N_{\nu ,2}}{2mC_\nu }(1-\textrm{e}^{-\frac{1}{8}\nu \pi })$. The conclusion of the lemma follows swiftly for all $\varepsilon \le \varepsilon _0$. $\square$

#### Lemma A.9

Let $y_0\in [0,1]$ and $0\le \varepsilon \le \frac{\beta }{m}$. Then,

- If $my_0\le 3\beta$, there exists $\varepsilon _0>0$ such that
  $$\begin{aligned} \left( m|y_0+ i\varepsilon |\right) ^\frac{1}{2}\lesssim |M_\pm (y_0+ i\varepsilon )| \end{aligned}$$
  for all $$0\le \varepsilon \le \varepsilon _0$$.
- If $$my_0\ge 3\beta $$,
  $$\begin{aligned} 1\lesssim {|M_\pm (y_0+i\varepsilon )|}. \end{aligned}$$

#### Proof

For the first part of the Lemma, recall $M_+(\zeta )=e^{-\frac{1}{2}\zeta }\zeta ^{a}M\left( a, b,\zeta \right)$, the lemma follows once we obtain lower bounds on $e^{-\frac{1}{2}\zeta }M\left( a,b,\zeta \right)$. For this, note that since $\frac{\textrm{d}}{\textrm{d}\zeta }M(a,b,\zeta )=\frac{1}{2}M(a+1,b+1,\zeta )$, which is entire in $\zeta \in {{\mathbb {C}}}$, we have that

$$\begin{aligned} M(a,b,2m(y_0+i\varepsilon ))=M(a,b,2my_0) + \int _{0}^{\varepsilon }imM(a+1,b+1,2m(y_0+is))\textrm{d}s. \end{aligned}$$

We further bound the error term by noting that $|2\,m(y_0+is)|\le 10\beta$, for all $|s|\le |\varepsilon |$. As a result, there exists $C>0$ such that $|M(a+1,b+1,2\,m(y_0+is))|\le C$, for all $|s|\le |\varepsilon |$. Therefore, using the lower bounds on $|M(a,b,2my_0)|$ from Lemma A.5,

$$\begin{aligned} \begin{aligned} \left| \int _{0}^{\varepsilon }imM(a+1,b+1,2m(y_0+is))\textrm{d}s\right|&\le |M(a,b,2my_0)|\frac{C m|\varepsilon |}{|M(a,b,2my_0)|} \\&\le C|M(a,b,2my_0)||\Gamma (1+i\nu )|\sqrt{\frac{\nu \pi }{\sinh \nu \pi }}m|\varepsilon |. \\ \end{aligned} \end{aligned}$$

In particular, there exists $\varepsilon _0>0$ such that for all $0\le \varepsilon \le \varepsilon _0$,

$$\begin{aligned} \begin{aligned} e^{-my_0}|M(a,b,2m(y_0+i\varepsilon ))|&\ge e^{-my_0}|M(a,b,2my_0)|\left( 1-Cm\frac{\varepsilon }{|M(a,b,2my_0)|}\right) \\&\ge \frac{1}{2}\frac{1}{|\Gamma (1+i\nu )|}\sqrt{\frac{\sinh (\nu \pi )}{\nu \pi }}, \end{aligned} \end{aligned}$$

and the first part of the lemma follows. As for the second statement, it is a direct consequence of Lemma A.6 and the fact that $|M_\pm (\cdot )|$ is bounded in compact domains (it is entire). $\square$

### Growth bounds and comparison estimates for $\beta ^2=1/4$

#### Lemma A.10

Let $\beta ^2 = 1/4$ and let $\mu =\sqrt{1/4-\beta ^2}$. Denote $a:=\frac{1}{2}$ and $b:=2a=1$. Then, there exists $N_0>0$ such that

$$\begin{aligned} \left| \frac{W_{0,0}(\zeta )}{M_{0,0}(\zeta )}\right| \le 2\sqrt{\pi }\textrm{e}^{-\textrm{Re}\zeta }, \quad \left| \frac{W_{0,0}'(\zeta )}{W_{0,0}(\zeta )} + \frac{1}{2} \right| \le \frac{1}{4}, \quad \frac{1}{2}e^{-\frac{1}{2}\textrm{Re}\zeta }\le |W_{0,0}(\zeta )| \le \frac{3}{2}\textrm{e}^{-\frac{1}{2}\textrm{Re}\zeta } \end{aligned}$$

for all $$\textrm{Re}\zeta \ge N_0$$.

#### Proof

Let $\zeta \in {{\mathbb {C}}}$ and $\delta >0$. We recall that

$$\begin{aligned} \begin{aligned} M_{0,0}(\zeta )&=\textrm{e}^{-\frac{1}{2}\zeta }\zeta ^{\frac{1}{2}} M(1/2,1,\zeta ) \\&=\textrm{e}^{-\frac{1}{2}\zeta }\zeta ^{1/2}\frac{\Gamma (1)}{\Gamma (1/2)}\left( -iU(1/2,1,\zeta ) + i\textrm{e}^\zeta U(1/2,1,\textrm{e}^{i\pi } \zeta )\right) , \end{aligned} \end{aligned}$$

while also

$$\begin{aligned} W_{0,0}(\zeta )=\textrm{e}^{-\frac{1}{2}\zeta }\zeta ^\frac{1}{2}U(1/2,1,\zeta ). \end{aligned}$$

Thus, we have that

$$\begin{aligned} \frac{W_{0,0}(\zeta )}{M_{0,0}(\zeta )}=-i\sqrt{\pi }\frac{U(1/2,1,\zeta )}{\textrm{e}^\zeta U(1/2,1,\textrm{e}^{i\pi }\zeta ) - U(1/2,1,\zeta )}=-i\sqrt{\pi }\frac{1}{\textrm{e}^\zeta \frac{U(1/2,1,\textrm{e}^{i\pi }\zeta )}{U(1/2,1,\zeta )}-1}. \end{aligned}$$

Now, we also recall that $U(1/2,1,\zeta )=\zeta ^{-\frac{1}{2}}\left( 1 + \zeta ^\frac{1}{2}{{\mathcal {E}}}_1(\zeta )\right)$, with $|\zeta ^\frac{1}{2}{{\mathcal {E}}}_1(\zeta )|\le \frac{1}{2|\zeta |}\textrm{e}^{\frac{1}{2|\zeta |}}$. Therefore, we have the lower bound

$$\begin{aligned} \left| \frac{U(1/2,1,\textrm{e}^{i\pi }\zeta )}{U(1/2,1,\zeta )} \right| \ge \frac{3}{4}, \end{aligned}$$

for $|\zeta |$ sufficiently large. Moreover, $\frac{3}{4} \textrm{e}^{\textrm{Re}\zeta }-1\ge \frac{1}{2} \textrm{e}^{\textrm{Re}\zeta }$, for all $\textrm{Re}\zeta \ge 2\ln 2$. The desired conclusion follows.

For the second part of the Lemma, since $W_{0,0}(\zeta )=e^{-\frac{1}{2}\zeta }\zeta ^\frac{1}{2}\left( \zeta ^{-\frac{1}{2}} + {{\mathcal {E}}}_1(\zeta )\right)$, we note that

$$\begin{aligned} W_{0,0}'(\zeta )= -\frac{1}{2} W_0(\zeta ) +\frac{1}{2}\frac{W_0(\zeta )}{\zeta } + e^{-\frac{1}{2}\zeta }\left( -\frac{1}{2\zeta } + \zeta ^\frac{1}{2}{{\mathcal {E}}}_1'(\zeta )\right) , \end{aligned}$$

where we recall that $\left| \zeta ^\frac{1}{2}{{\mathcal {E}}}_1'(\zeta )\right| \le \frac{1}{4|\zeta |}e^{\frac{1}{2|\zeta |}}\le x$, for $x=\frac{1}{2|\zeta |}\le \frac{1}{2}$. Hence,

$$\begin{aligned} \left| \frac{W_{0,0}'(\zeta )}{W_{0,0}(\zeta )} + \frac{1}{2}\right| \le x + 2x\left| \frac{1}{1+\zeta ^\frac{1}{2}{{\mathcal {E}}}_1(\zeta )}\right| \le 2x\le \frac{1}{4}, \end{aligned}$$

for all $$x\le \frac{1}{8}$$.

For the third statement of the Lemma, note that $W_{0,0}(\zeta )=e^{-\frac{1}{2}\zeta }\left( 1+ \zeta ^\frac{1}{2}{{\mathcal {E}}}_1(\zeta )\right)$, the conclusion follows for $|\zeta |$ large enough so that $\left| \zeta ^\frac{1}{2}{{\mathcal {E}}}_1(\zeta ) \right| \le \frac{1}{2}$. $\square$

#### Lemma A.11

Let $\beta ^2 = 1/4$. Denote $a:=\frac{1}{2} \pm \mu$ and $b:=2a=1$. Then, for all $\epsilon >0$ there exists $\delta _0>0$ such that

$$\begin{aligned} \left| \frac{M_{0,0}(\zeta )}{W_{0,0}(\zeta )}\right| \le \epsilon , \end{aligned}$$

for all $\zeta \in {{\mathbb {C}}}$ such that $|\zeta |\le \delta$.

#### Proof

We use the functional relation between the Whittaker functions and the modified Bessel functions in order to extract the correct asymptotic behaviour of the functions near the origin and estimate the quotient precisely. In this direction, recall that

$$\begin{aligned} M_{0,0}(2\zeta )=\sqrt{2\zeta }I_0(\zeta ), \quad W_{0,0}(2\zeta ) = \sqrt{\frac{2\zeta }{\pi }}K_0(\zeta ), \end{aligned}$$

where $I_0(\zeta )$ and $K_0(\zeta )$ denote the modified Bessel functions of order 0. Moreover, we have that

$$\begin{aligned} K_0(\zeta )=-\left( \ln \left( \frac{\zeta }{2}\right) + \varsigma \right) I_0(\zeta ) +2\sum _{k=1}^\infty \frac{I_{2k}(\zeta )}{k}, \end{aligned}$$

where

$$\begin{aligned} I_{2k}(\zeta )=\left( \frac{\zeta }{2}\right) ^{2k}\sum _{j\ge 0}\frac{\left( \frac{\zeta ^2}{4}\right) ^j}{j!(2k+j)!}. \end{aligned}$$

In particular, one observes that $|I_{2k}(\zeta )|\le I_{2k}(|\zeta |)$. Moreover, under the observation that $(2k+j)!\ge (2k)!j!$, we can bound

$$\begin{aligned} \left| 2\sum _{k=1}^\infty \frac{I_{2k}(z)}{k} \right| \le 2 \sum _{k=1}^\infty I_0(|\zeta |)\frac{\left( \frac{1}{2} |\zeta |\right) ^{2k}}{k(2k)!}\le 2I_0(|\zeta |)\left( \cosh \frac{1}{2}|\zeta | -1\right) . \end{aligned}$$

With this, together with the fact that $I_0(\cdot )$ is analytic in ${{\mathbb {C}}}$ and $I_0(\zeta )\rightarrow 1$ when $\zeta \rightarrow 0$, we have that

$$\begin{aligned} |K_0(\zeta )|\ge -\frac{1}{2}\ln \left( \frac{1}{2}|\zeta |\right) , \end{aligned}$$

for $|\zeta |$ sufficiently small. The conclusion follows, since for $|\zeta |$ sufficiently small we have

$$\begin{aligned} \left| \frac{M_{0,0}(\zeta )}{W_{0,0}(\zeta )}\right| \le -\frac{3}{\ln \left( \frac{1}{4} |\zeta | \right) } \end{aligned}$$

$$\square $$

#### Lemma A.12

Let $\beta ^2=1/4$ and let $y_0\in [0,1]$ such that $N_2\le 2my_0\le N_1$. Then, for all $\epsilon >0$ there exists $\varepsilon _0>$ such that

$$\begin{aligned} \left| \frac{W_0(y_0-i\varepsilon )}{M_0(y_0-i\varepsilon )}-\frac{W_0(y_0)}{M_0(y_0)}\right| \le \epsilon , \quad \left| \frac{W_0(y_0-i\varepsilon )}{M_0(y_0-i\varepsilon )}\right| \le C \end{aligned}$$

for all $\varepsilon \le \varepsilon _0$ and some $C>0$. In particular,

$$\begin{aligned} \left| \textrm{Im}\left( \frac{W_0(y_0-i\varepsilon )}{M_0(y_0-i\varepsilon )}\right) \right| \le \epsilon , \end{aligned}$$

#### Proof

It follows from the continuity of the functions involved, plus the fact that $M_{0,0}(x)$ does not vanish and $W_{0,0}(x)$ is bounded, for any $x>0$ such that $0<N_2\le x \le N_1< \infty$. $\square$

#### Lemma A.13

There exists $\delta _2>0$ such that

$$\begin{aligned} |\zeta |^\frac{1}{2} \left( 1 + \big | \log |\zeta | \big | \right) \lesssim |W_{0,0}(\zeta )|, \end{aligned}$$

for all $$|\zeta |\le \delta _2$$.

#### Proof

Recall that $W_{0,0}(\zeta )= \sqrt{\frac{\zeta }{\pi }}K_0(\zeta /2)$ and the fact that $|K_0(\zeta )|\ge -\frac{1}{2}\log \left( \frac{|\zeta |}{2}\right)$ for $\zeta \rightarrow 0$. Then,

$$\begin{aligned} |W_{0,0}(\zeta )|\ge \frac{1}{2\sqrt{\pi }}|\zeta |^\frac{1}{2} \big | \log |\zeta | - \log 4 \big |\ge \frac{1}{20\sqrt{\pi }}|\zeta |^\frac{1}{2}\big | \log |\zeta | \big | \ge \frac{1}{40\sqrt{\pi }}|\zeta |^\frac{1}{2}\left( 1 + \big | \log |\zeta | \big | \right) , \end{aligned}$$

for $|\zeta |$ sufficiently small. $\square$

#### Lemma A.14

Let $y_0\in [0,1]$ and $0\le \varepsilon \le \frac{\beta }{m}$. Then,

- If $my_0\le 3\beta$, there exists $\varepsilon _0>0$ such that
  $$\begin{aligned} \left( m|y_0+ i\varepsilon |\right) ^{\frac{1}{2}}\lesssim |M_0(y_0+ i\varepsilon )| \end{aligned}$$
  for all $$0\le \varepsilon \le \varepsilon _0$$.
- If $$my_0\ge 3\beta $$,
  $$\begin{aligned} 1\lesssim {|M_\pm (y_0+i\varepsilon )|}. \end{aligned}$$

#### Proof

The proof uses the ideas from Lemma A.9 together with the bounds from Lemma A.15. We omit the details. $\square$

### Growth bounds and comparison estimates for $\beta ^2<1/4$

In this subsection we consider the case $\beta ^2 < 1/4$, for which $\mu =\sqrt{1/4-\beta ^2}$ with $\mu \in \left( 0,\frac{1}{2}\right)$ and $\nu =0$.

#### Lemma A.15

Denote $a_\pm :=\frac{1}{2} \pm \mu$ and $b_\pm :=2a_\pm$. Then,

$$\begin{aligned} \lim _{\textrm{Re}(\zeta )\rightarrow +\infty }\left| \frac{\Gamma (a)}{\Gamma (b)}\textrm{e}^{-\frac{1}{2}\zeta }M_+(\zeta )\right| =1. \end{aligned}$$

Moreover, let $C_\mu =2^{-4\mu }\frac{\Gamma (1-\mu )}{\Gamma (1+\mu )}$. There exists $N_{\mu ,0}>0$ such that

$$\begin{aligned} \left| \frac{M_-(\zeta )}{M_+(\zeta )} - 2^{-4\mu }\frac{\Gamma (1-\mu )}{\Gamma (1+\mu )} \right| \le \min \left( 5, \frac{\tan \mu \pi }{1+\tan \mu \pi }\right) \frac{C_\mu }{4}. \end{aligned}$$

for all $$\textrm{Re}\zeta \ge N_{\mu ,0}$$. $$\square $$

#### Proof

Let $\zeta \in {{\mathbb {C}}}$ and $\delta >0$. We recall that

$$\begin{aligned} \begin{aligned} M_\pm (\zeta )&=\textrm{e}^{-\frac{1}{2}\zeta }\zeta ^{a_\pm } M(a_\pm ,b_\pm ,\zeta ) \\&=\textrm{e}^{-\frac{1}{2}\zeta }\zeta ^{a_\pm }\frac{\Gamma (b_\pm )}{\Gamma (a_\pm )}\left( \textrm{e}^{-i\pi a_\pm }U(a_\pm ,b_\pm ,\zeta ) + \textrm{e}^{i\pi a_\pm }\textrm{e}^\zeta U(a_\pm ,b_\pm ,\textrm{e}^{i\pi } \zeta )\right) . \end{aligned} \end{aligned}$$

Moreover, we have that $U(a_\pm ,b_\pm ,\zeta ) = \zeta ^{-a_\pm }+ \mathcal {E}_{\pm }(\zeta )$, where further

$$\begin{aligned} |\zeta ^{a_\pm }{{\mathcal {E}}}_\pm (\zeta )|\le \frac{2\beta ^2}{|\zeta |}\textrm{e}^{\frac{2\beta ^2}{|\zeta |}}. \end{aligned}$$

In the sequel, we write $x:=\frac{2\beta ^2}{|\zeta |}$. Therefore, we can write

$$\begin{aligned} M_\pm (\zeta )=\frac{\Gamma (b_\pm )}{\Gamma (a_\pm )}\textrm{e}^{\frac{1}{2}\zeta }\left( \left[ 1 + (\textrm{e}^{i\pi }\zeta )^{a_\pm }{{\mathcal {E}}}_\pm (\textrm{e}^{i\pi }\zeta )\right] + \textrm{e}^{-\zeta }\textrm{e}^{-i\pi a_\pm }\left[ 1+ \zeta ^{a_\pm }{{\mathcal {E}}}_\pm (\zeta )\right] \right) \end{aligned}$$

Now, since $b_\pm =2a_\pm$, we have that

$$\begin{aligned} \frac{\Gamma (b_\pm )}{\Gamma (a_\pm )}=\pi ^{-\frac{1}{2}}2^{2a_\pm -1}\Gamma \left( a_\pm +\frac{1}{2}\right) , \end{aligned}$$

confer, [26]. Therefore,

$$\begin{aligned} \frac{\displaystyle {\Gamma (b_-)}/{\Gamma (a_-)}}{\displaystyle {\Gamma (b_+)}/{\Gamma (a_+)}}=2^{-4\mu }\frac{\Gamma (1-\mu )}{\Gamma (1+\mu )} \end{aligned}$$

and we note that

$$\begin{aligned} \frac{M_-(\zeta )}{M_+(\zeta )}=2^{-4\mu }\frac{\Gamma (1-\mu )}{\Gamma (1+\mu )}\frac{ 1 + (\textrm{e}^{i\pi }\zeta )^{a_-}{{\mathcal {E}}}_-(\textrm{e}^{i\pi }\zeta ) + \textrm{e}^{-\zeta }\textrm{e}^{-i\pi a_-}\left[ 1+ \zeta ^{a_-}{{\mathcal {E}}}_-(\zeta )\right] }{ 1 + (\textrm{e}^{i\pi }\zeta )^{a_+}{{\mathcal {E}}}_+(\textrm{e}^{i\pi }\zeta ) + \textrm{e}^{-\zeta }\textrm{e}^{-i\pi a_+}\left[ 1+ \zeta ^{a_+}{{\mathcal {E}}}_+(\zeta )\right] }. \end{aligned}$$

Moreover, we observe that that $|\zeta ^a{{\mathcal {E}}}_1(\zeta )|\le \frac{4\beta ^2}{|\zeta |}$, for $|\zeta |\ge 4\beta ^2$. Hence, for any $\delta >0$,

$$\begin{aligned} \left| (\textrm{e}^{i\pi }\zeta )^{a_\pm }{{\mathcal {E}}}_\pm (\textrm{e}^{i\pi }\zeta ) + \textrm{e}^{-\zeta }\textrm{e}^{-i\pi a_\pm }\left( 1+\zeta ^{a_\pm }{{\mathcal {E}}}_\pm (\zeta )\right) \right| \le \delta \end{aligned}$$

provided that $\textrm{Re}\zeta > N_{\mu ,0}$ for some $N_{\mu ,0}>0$. $\square$

#### Lemma A.16

Denote $a_\pm :=\frac{1}{2} \pm \mu$ and $b_\pm :=2a_\pm$. Then,

$$\begin{aligned} \lim _{\zeta \rightarrow 0}\frac{M_+(\zeta )}{M_-(\zeta )}=0. \end{aligned}$$

Therefore, there exists $\delta _{\mu ,1}>0$ such that

$$\begin{aligned} \left| \frac{M_+(\zeta )}{M_-(\zeta )}\right| \le \min \left( \frac{1}{5M(a_-,b_-,2N_{\mu ,0})}, \frac{1}{3C_\mu }\right) \end{aligned}$$

for all $\zeta \in {{\mathbb {C}}}$ such that $|\zeta |\le \delta _{\mu ,1}$.

#### Proof

We recall once again that

$$\begin{aligned} M_\pm (\zeta )=\textrm{e}^{-\frac{1}{2}\zeta }\zeta ^{a_\pm } M(a_\pm ,b_\pm ,\zeta ). \end{aligned}$$

Hence, we directly compute

$$\begin{aligned} \frac{M_+(\zeta )}{M_-(\zeta )}=\zeta ^{2\mu }\frac{M(a_+,b_+,\zeta )}{M(a_-,b_-,\zeta )}. \end{aligned}$$

Since $M(a_\pm ,b_\pm ,\zeta )\rightarrow 1$ for $\zeta \rightarrow 0$, and $2\mu >0$, the conclusion follows for $|\zeta |$ small enough.

#### Lemma A.17

Denote $a_\pm :=\frac{1}{2} \pm \mu$ and $b_\pm :=2a_\pm$. Let $y_0\in [0,1]$ such that $N_{\mu ,1}\le 2my_0\le N_{\mu ,0}$, for some $N_{\mu ,1}\in {{\mathbb {R}}}$. Then, there exists $\varepsilon _0>0$ such that

$$\begin{aligned} \left| \frac{M_\pm (y_0+i\varepsilon )}{M_\pm (y_0)} -1 \right| \le \frac{\sin \mu \pi }{5}, \end{aligned}$$

for all $$0<|\varepsilon |\le \varepsilon _0$$.

#### Proof

Assume without loss of generality that $\varepsilon >0$. Then,

$$\begin{aligned} M_\pm (y_0+i\varepsilon )=M_\pm (y_0) + \int _0^\varepsilon \frac{\textrm{d}}{\textrm{d}s}M_\pm (y_0+is)\textrm{d}s. \end{aligned}$$

Thanks to the asymptotic expansions for small arguments we next estimate

$$\begin{aligned}  &   \left| \int _0^\varepsilon \frac{\textrm{d}}{\textrm{d}s}M_\pm (y_0+is)\textrm{d}s \right| \le \int _0^\varepsilon |M_\pm '(y_0+is)| \textrm{d}s \le C_\mu \int _0^\varepsilon \frac{(2m)^{\frac{1}{2} \pm \mu }}{|y_0+is|^{\frac{1}{2} \mp \mu }}\textrm{d}s \\  &   \quad \le C_\mu (2m\varepsilon )(2my_0)^{-\frac{1}{2}}. \end{aligned}$$

Using the lower bound $(2my_0)^{\frac{1}{2}\pm \mu }\le M_\pm (y_0)$ we have that

$$\begin{aligned} \left| \int _0^\varepsilon \frac{\textrm{d}}{\textrm{d}s}M_\pm (y_0+is)\textrm{d}s \right| \le C_\mu M_\pm (y_0)\frac{\varepsilon }{y_0}\le C_\mu M_\pm (y_0)N_{2}^{-1}2m\varepsilon . \end{aligned}$$

Hence,

$$\begin{aligned} M_\pm (y_0)\left( 1-C_\mu \frac{\varepsilon }{N_2}\right) \le |M_\pm (y_0+ i\varepsilon )|\le M_\pm (y_0)\left( 1+C_\mu \frac{\varepsilon }{N_2}\right) \end{aligned}$$

and now choose $\varepsilon _0>0$ sufficiently small, so that the conclusion of the lemma follows swiftly for all $\varepsilon \le \varepsilon _0$. $\square$

#### Lemma A.18

Let $y_0\in [0,1]$ and $0\le \varepsilon \le \frac{\beta }{m}$. Then,

- If $my_0\le 3\beta$, there exists $\varepsilon _0>0$ such that
  $$\begin{aligned} \left( m|y_0+ i\varepsilon |\right) ^{\frac{1}{2}\pm \mu }\lesssim |M_\pm (y_0+ i\varepsilon )| \end{aligned}$$
  for all $$0\le \varepsilon \le \varepsilon _0$$.
- If $$my_0\ge 3\beta $$,
  $$\begin{aligned} 1\lesssim {|M_\pm (y_0+i\varepsilon )|}. \end{aligned}$$

#### Proof

The proof uses the ideas from Lemma A.9 together with the bounds from Lemma A.15. We omit the details. $\square$
